# Integrative taxonomy of cryptic *Pachypus* chafers using museomics, morphometrics, barcoding, and genomic DNA analysis (Coleoptera: Scarabaeidae: Pachypodinae)

**DOI:** 10.1038/s41598-026-47761-7

**Published:** 2026-05-20

**Authors:** Dirk Ahrens, Erika Bazzato, Ana Cristina Castro Lopez, Marcel Krause, Cesare Ancona, Christina Blume, Lia Botjes, Giuseppe Carpaneto, Davide Cillo, Jonas Eberle, Christoph Mayer, Thaynara L. Pacheco, Guido Sabatinelli, Ignazio Sparacio, Marco Uliana, Oliver Niehuis, Lars Podsiadlowski, Lars Dietz

**Affiliations:** 1 Museum A. Koenig, Leibniz Institute of Biodiversity Change, Adenauerallee 127, 53113 Bonn, Germany; 2 Department of Life and Environmental Sciences, Botany Division, University of Cagliari, viale Sant’Ignazio da Laconi 13, Cagliari, 09123 CA Italy; 3 Via P. Mascagni 3, Ussana, 09020 SU Italy; 4 Dipartimento di Scienze, Università Roma Tre, Viale Marconi 446, Roma, 00146 Italy; 5 Via Zeffiro 8, Cagliari, 09130 Italy; 6 University of Salzburg, Environment & Biodiversity, Salzburg, Austria; 7 Muséum d’Histoire Naturelle, Route de Malagnou 1, Geneva, 1208 Switzerland; 8 via Principe di Paternò 3, Palermo, 90143 Italy; 9 Museo di Storia Naturale, Santa Croce 1730, Venezia, 30135 Italy; 10 Abteilung Evolutionsbiologie und Ökologie, Institut für Biologie I, Albert-Ludwigs- Universität Freiburg, Freiburg, Germany

**Keywords:** Museomics, Mzl-USCOs, Integrative taxonomy, Morphometrics, Chafer beetles, Ecology, Ecology, Evolution, Genetics, Molecular biology, Zoology

## Abstract

The taxonomy of the endemic Mediterranean genus *Pachypus* Dejean, 1821 has long been complicated by cryptic diversity and limited morphological differentiation. Based on extensive results from previous studies and new genomic data analyses, we perform here an integrative taxonomic revision of the genus. Using the morphological examination of the type material of most species and *de novo* sequencing of 978 metazoan-level universal single-copy orthologous genes (mzl-USCOs) of the almost 200-year-old lectotype of *Pachypus impressus* Erichson, 1840, we proposed refined species hypotheses within the genus. Resulting species entities from a recent study using mzl-USCOs were aligned within the framework of existing nomenclatorially available names inferred from Museomics and comparative morphological analyses. We additionally removed two existing taxon names from synonymy with *Pachypus candidae* and raised them to valid species: *Pachypus cornutus* (Olivier, 1789) and *P. impressus* Erichson, 1840. *Pachypus excavatus* Fabricius, 1792 is confirmed here as a valid species. This study also revealed one new synonymy: *Pachypus impressus* Erichson, 1840 (= *P. melonii* Sparacio, 2008, syn. n.). Furthermore, we described seven new species which had been already delineated in our earlier study: *Pachypus baroniensis* sp. n., *P. franginii* sp. n., *P. gallurensis* sp. n., *P. matzaccara* sp. n., *P. occidentalis* sp. n., *P. pelegrinus* sp. n., and *P. sulcis* sp. n. Extensive morphometric analyses of five datasets (one based on distance measurements and four on body outlines), including about 1,900 specimens, confirmed the cryptic character of most *Pachypus* species, as morphospace plots of all traits largely overlapped among most of the species. This made the assignment of analyzed type specimens to retrieved morphometric clusters a poor predictor of the true identity of mzl-USCO-defined species. We designated a lectotype of *Pachypus impressus* Erichson, 1840 and a neotype of *Pachypus cornutus* (Olivier, 1789). Finally, for the purpose of enhancing applied DNA-based studies and metabarcoding, we extracted the 5’ COI DNA sequences from the raw DNA reads of our target enrichment nucleotide data as well as from the newly sequenced lectotype of *P. impressus*. The phylogenetic tree inferred from these data was compared with that obtained from the previously produced 3’ COI data generated by Sanger sequencing. The habitus and male genitalia of all species are illustrated, an identification key is provided, and the distribution of the species is shown. As a result of our long-term studies of the genus, we also provide new insights into the ecology and distribution of the group, which may be relevant to conservation across the entire circum-Tyrrhenian region. Zoobank link: urn:lsid:zoobank.org:pub:34F3F1E1-2A40-4450-95B3-0336E6E2CF52.

**Supplementary Information:**

The online version contains supplementary material available at 10.1038/s41598-026-47761-7.

## Introduction

Insects in drier regions often exhibit a subterranean lifestyle, at least temporarily. This is often connected with the loss of flight capability^1,2^. The resulting reduced dispersal capacity has a major impact on the evolution and diversification of these dry-adapted faunas^3,4^ as gene flow between populations can be affected. In some cases, wing and flight reduction affects only one sex^2^, namely females. This, in turn, results not only in strong sexual dimorphism but also in asymmetric gene flow between sexes. The latter, in particular, may affect current approaches and protocols for recognizing and delimiting species using DNA sequence data^5^.

One such prominent example is the genus *Pachypus* Dejean, 1821. The genus appears to be a phylogenetically isolated lineage within the phytophagous Scarabaeidae (Pleurosticti), with no known close relatives^6–8^. Accordingly, it is often placed in its own subfamily, Pachypodinae^9^. Adults of both sexes of *Pachypus* do not feed and have almost entirely reduced mouthparts. The saprophagous, endogeous larva is the only feeding stage. *Pachypus* is unique among the Scarabaeoidea for its female brachyelytry (i.e., females lack both pairs of wings^10^. Females live almost entirely underground in burrows, while males are very good fliers^11,12^.

For a long time, only three *Pachypus* species had been recognized^13^, to which two additional species were later added, originating both from Sardinia^14,15^. Several species recognized earlier^16–18^ were thought to be synonymous with *Pachypus candidae* Petagna, 1787^13,19,20^.

**Fig. 1:**
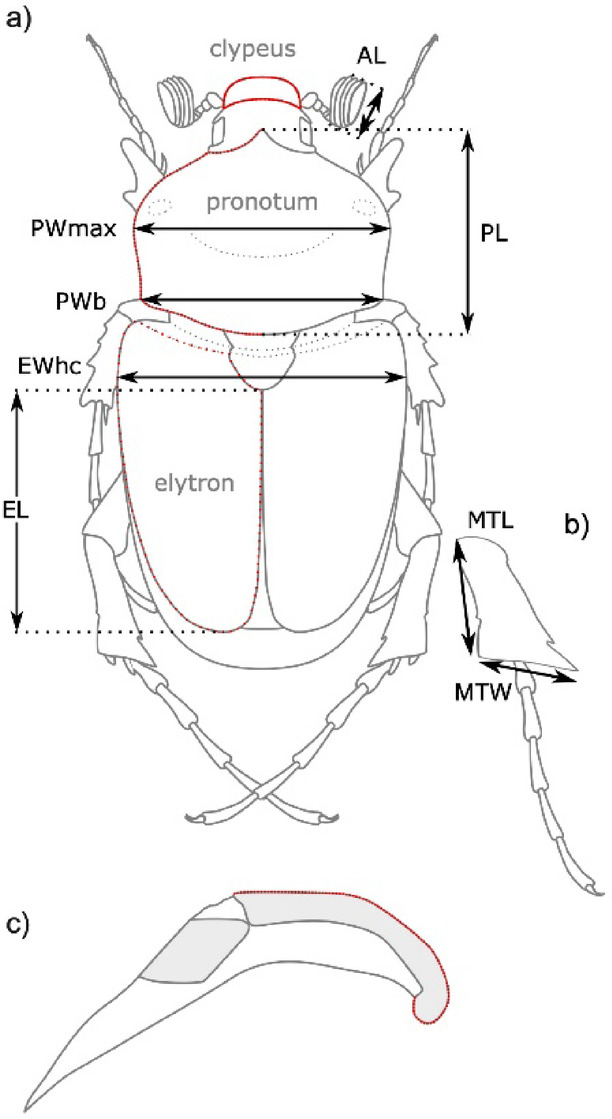
Linear measurements and outlines used in morphometric analyses of (**a**) adult males (habitus dorsal view; clypeus, pronotum [left half]), elytron), (**b**) metatibia (lateral view), and (**c**) their copulation organ (paramere, lateral view). MTL and MTW were measured in lateral view of the leg. Outlines were digitized as 100 equidistant semilandmarks (red dots). AL = length of antennal club, EWhc = elytron width at humeral callus, EL = elytron length, PWmax = maximum pronotum width, PWb = width of pronotum at base, PL = medial pronotum length, MTL = length of metatibia, MTW = maximum width of metatibia.

Eberle et al.^5^ used an integrative taxonomic approach to study *Pachypus* using two mitochondrial and two nuclear genes, as well as five morphometric datasets (Fig. 1). They found large discrepancies between different species delimitation algorithms, with over-splitting almost twofold higher when using mitochondrial data compared to nuclear data. The highest number of inferred species entities was 111. In contrast, trees based on nuclear DNA showed low resolution and branch support. Eberle et al.^5^ confirmed the use of mtDNA as the main cause of over-splitting. Based on their integrative analysis, Eberle et al.^5^ concluded that the genus comprises at least twelve species, most of them previously assigned to *P. candidae* Petagna, 1787. The variation in their morphometric data was consistent with these hypothesized species entities, but also with metapopulations inferred by GMYC analysis^21^ on mtDNA.

**Fig. 2:**
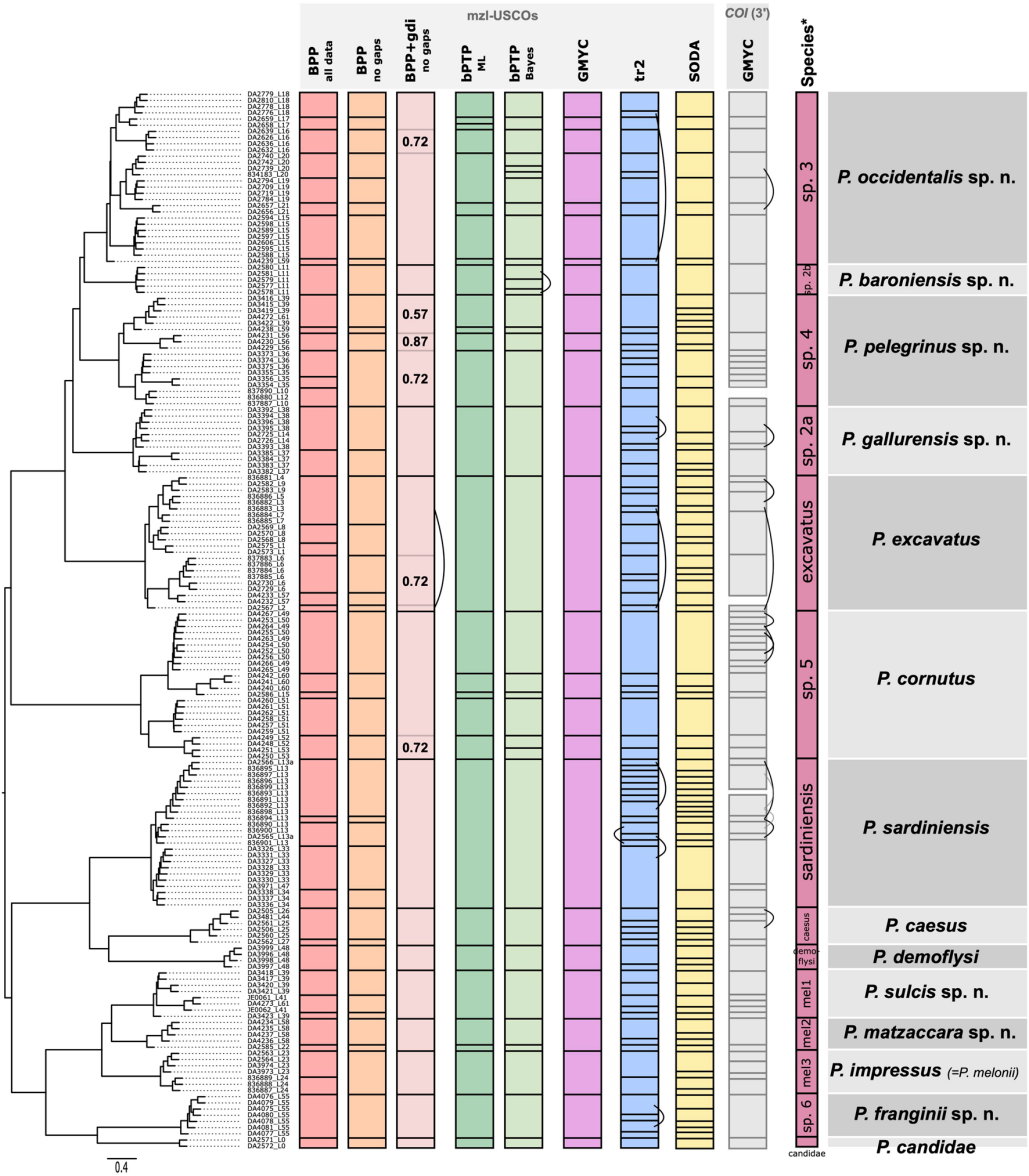
Synoptic overview of species delimitation results from mzl-USCOs (Dietz et al^22^.) from the various applied delimitation approaches, compared to the final species hypothesis and the valid associated species name based on the conclusion of this study.

Dietz et al.^22^ revisited the phylogeny, taxonomy, and population genetics of *Pachypus* using single copy orthologous genes (USCOs), particularly those specific for the metazoan level (mzl-USCOs). USCOs are defined as gene loci that are present in single copy in at least 90% of all known genomes of a given taxonomic group, in this case Metazoa^23,24^. USCOs were originally developed to benchmark the quality of genome and transcriptome assemblies^23,25^ but have also been proposed as a universal marker set for animal taxonomy^24^. Using this approach, closely related species can be discriminated successfully, including species that could not be distinguished by mitochondrial data alone^26^. Dietz et al.^22^ inferred for the first time the timing of evolution, the population structure and hybridization of *Pachypus* species. Based on their thorough integrative analysis, Dietz et al.^22^ confirmed the existence of 14 species within *Pachypus*, including several undescribed species which we describe in the present study (Fig. 2). Using newly sequenced genomic and mitochondrial DNA data from historic specimens as well as extensive morphometric analyses, we refine and revise the taxonomy of the genus.

## Results

### De novo genomic sequencing and phylogenetic placement of the lectotype of *P. impressus*

mRNA sequencing of a freshly preserved specimen^8^ recovered 948 of the 978 mzl-USCOs. 940 of these were also found in the low-coverage genome data of the *P. impressus* lectotype. The latter had 33.42% missing data, significantly more than in data from other specimens obtained on average with targeted enrichment (average 14.83% +/- 2.18% s.d.). In addition, in the DNA sequences from the lectotype, a large proportion of nucleotides had lower sequence quality, as 41.15% of all recovered nucleotides were masked as lowercase by the software.

**Fig. 3:**
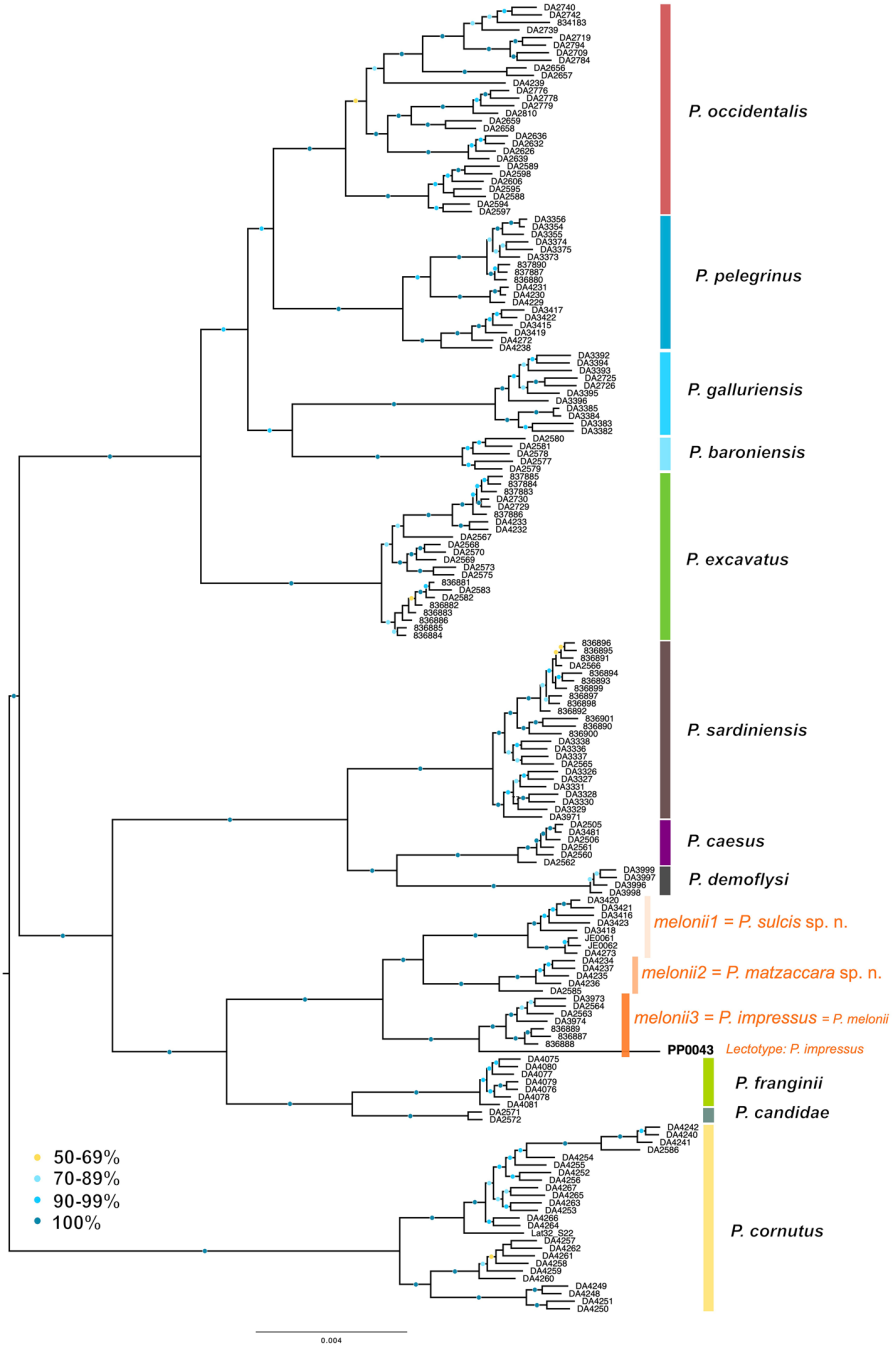
Maximum likelihood tree from IQ-TREE analysis of concatenated USCO data, with the phylogenetic position of the lectotype of *Pachypus impressus* within the lineages of the former *P. melonii* highlighted.

We reanalyzed the USCO dataset published by Dietz et al.^22^ with addition of the data from the lectotype of *Pachypus impressus* to determine to which lineage this name, which has been so far in synonymy with *P. candidae*, might belong. Among all available species names within *Pachypus*, only the identity of *Pachypus impressus* was uncertain, due to the ambiguous type locality indication (“Corsica and Sardinia”^18^) and the species richness in Sardinia. The ML tree based on the concatenated data (Fig. 3) as well as the tree from ASTRAL (Fig. S1) confirmed the association of the lectotype of *P. impressus* with the clade *P. melonii3*, i.e., actually the true *P. melonii* as sequenced specimens of this clade originate from the type locality of *P. melonii* (Assemini; Sardinia). This means that the two names are synonymous, with the older name, *Pachypus impressus*, being the valid one. In both analyses the lectotype of *Pachypus impressus* branched at the ancestral node of the *P. melonii*3 clade. In the MSC tree (Fig. S1) the branch leading to the remaining specimens of the clade was slightly longer than the branch to the common ancestor of them with *P. impressus*, very likely due to the lower data quality of the lectotype (see above). For the remaining lineages, species and specimens, the topology remained unchanged compared to that proposed by Dietz et al.^22^.

### COI barcoding

**Fig. 4:**
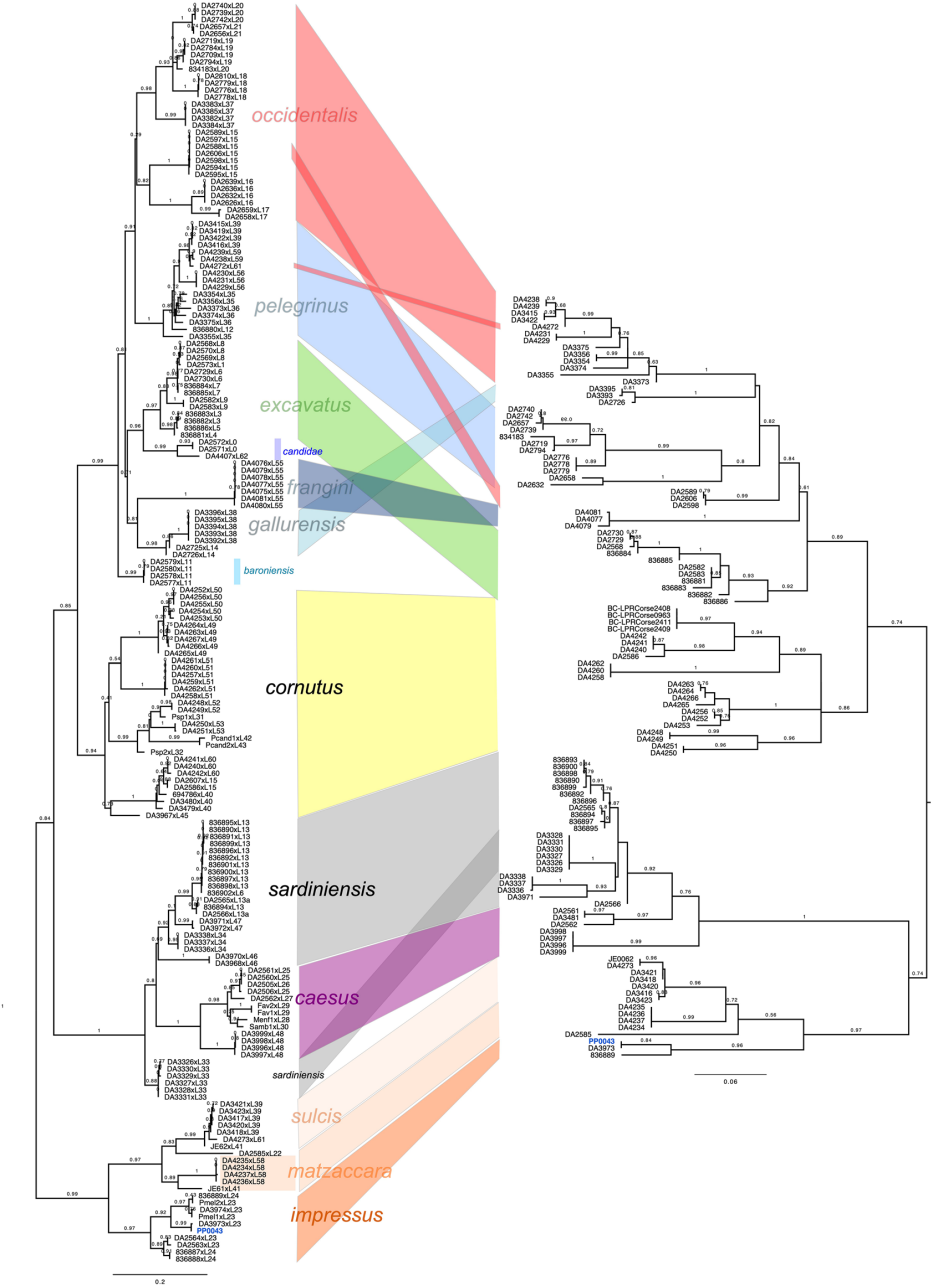
Maximum likelihood tree for the COI gene fragments and the phylogenetic placement of the lectotype of *Pachypus impressus* (PP0043): Left side: 3’ end (data from Eberle et al. 2019, reanalyzed); right side: 5’ end extracted from raw reads of genomic data (this study). For the latter, for two species, no sufficiently complete fragments could be extracted: *P. candidae* and *P. baroniensis*.

We analyzed the COI sequence data of the lectotype of *P. impressus*, which was extracted from the raw data of shallow genome sequencing, analyzing the 3’ and 5’ ends of COI separately. The 3’ dataset was produced with Sanger sequencing^5^, the rest of the 5’ set was produced by extracting the nucleotide sequences from raw data from USCO targeted enrichment sequencing. However, the 5’ set had incomplete data for many specimens, so we included in our analysis here only 107 specimens. The 5’ set therefore did not include sp2b (i.e., *P. baroniensis* sp. n.) and *P. candidae*. The mean nucleotide completeness of the 107 included specimens was only about 87% in the 5’ set. A maximum likelihood tree based on each of the two fragments of COI (Fig. 4) showed the lectotype of *P. impressus* to be closely related with the specimen DA3973, whose sequence was almost identical to that of the lectotype and which originated from Assemini (Sardinia), the type locality of *P. melonii*. The COI data of the lectotype were 100% complete for the COI 3’ data as well as for the 5’ data. The specimens retrieved from the BOLD database were identified as *P. cornutus*. We refrained from rerunning species delimitation analyses on the newly generated COI data, given the expected vast over-splitting which was already reported by Eberle et al.^5^ for the COI 3’ data.

### Morphometric analysis

**Fig. 5:**
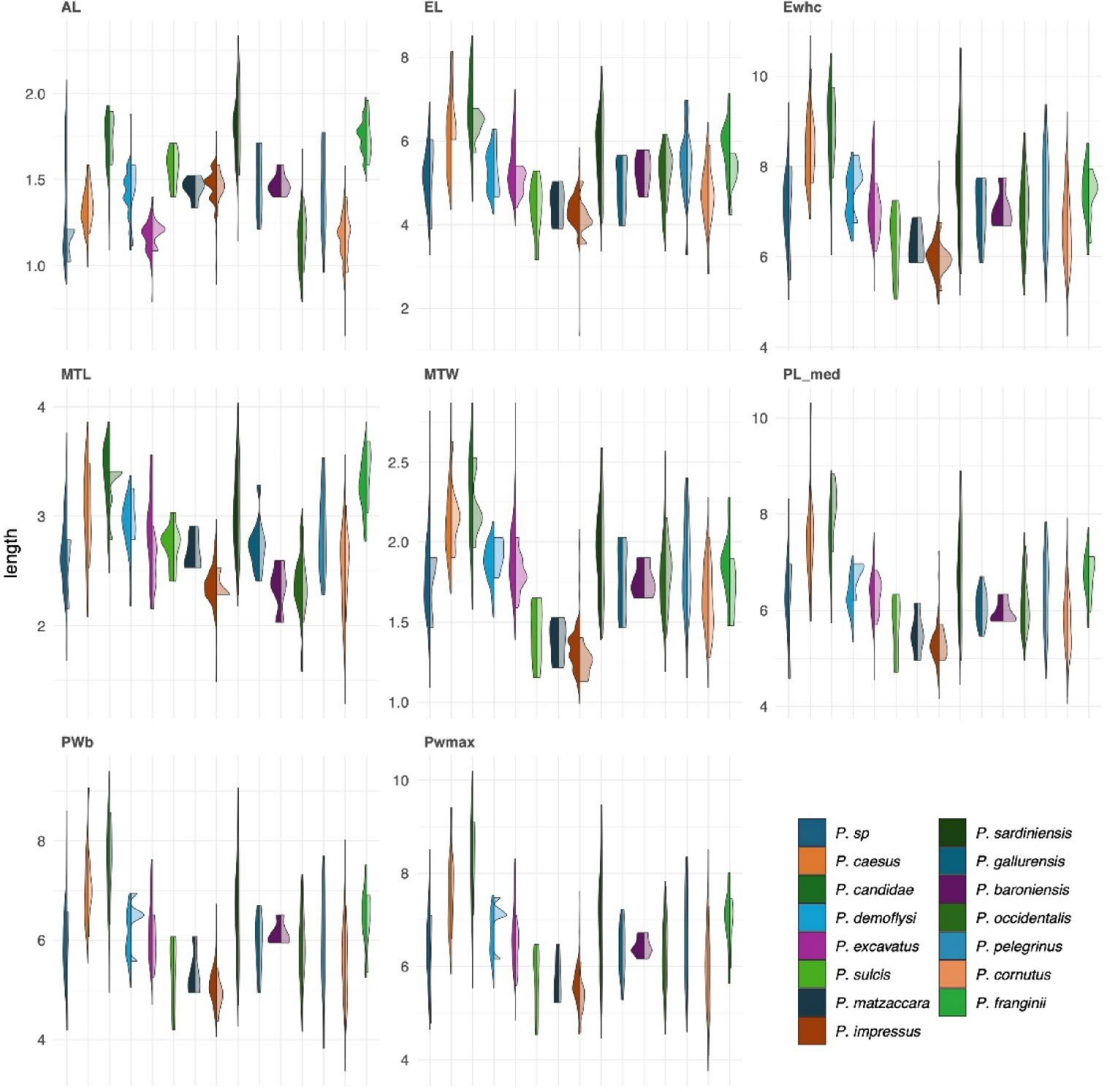
Bar plots showing the single trait variation of performed length measurements of the *Pachypus* species (left side: all identified specimens, this paper; right side - dataset of Eberle et al. 2019).

**Fig. 6:**
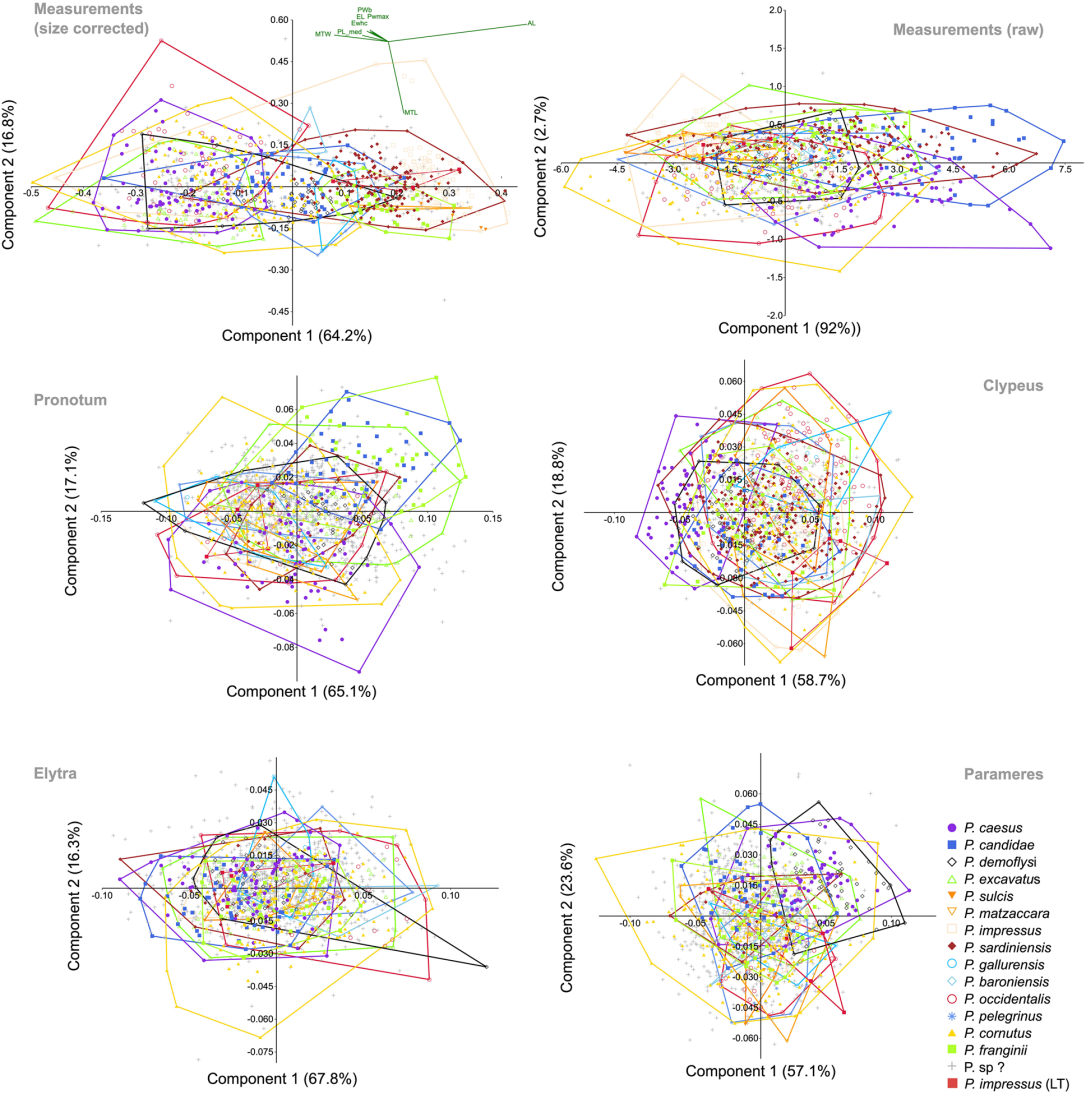
Morphospace plots (axis 1 and 2) from PCA and Procrustes analysis of the various traits: linear measurements (raw data and size-corrected); outlines of pronotum, clypeus, elytra, and parameres (see Fig. 1). The percentage of each axis to the total variation is given along the axes.

The morphometric analyses comprised five trait sets: linear measurements of body parts (which included 1,237 specimens), shape analysis of elytra (1,615 specimens), shape analysis of the pronotum (1,611 specimens), shape analysis of clypeus (1,432 specimens), and shape analysis of parameres (1,501 specimens). The total number of specimens that were represented in all five sets was 1,075. In all sets, the first two PC axes summarized more than 75% of the cumulative variation, 95% were reached at three axes for linear measurements and at five axes for shape-outlines, except in case of the pronotum and clypeus, where seven axes were needed (Supplementary file 9). In order to investigate putative diagnostic features between pairs of species in more detail, we plotted the variation of linear raw measurements for each species separately (Fig. 5). This comparison showed considerable overlap of all trait measurements for most species. Some species had very large trait variation (e.g., *P. sardiniensis*), which often reflected the number of examined specimens. In cases where only a few specimens were available, variation was often narrower. However, this pattern was not seen in *P. impressus* (i.e., the former *P. melonii* sensu stricto/*melonii* clade3), which showed little variation, particularly in the length of the antennal club, despite the considerably large number of examined specimens. This could be seen as an indication for a robust diagnostic character, at least for some species. Most other trait measurements were often quite variable within the species (Fig. 5). The same was also partly true for the metatibia width. This was reflected also by the plots of PC1 and PC2 for the size-corrected data (Fig. 6), in which according to the vector loadings, antennal length and metatibial width had the largest influence on the horizontal differentiation between the taxa. The vertical differentiation along axis 2 was less expressed and mainly triggered by the length of the metatibia. The raw measurements showed much less distinction between the species, as did the majority of remaining shape analyses based on the outline of pronotum, clypeus, elytra, and parameres (Fig. 6). Given the considerable amount of overlap between the hypothesized species, we refrained here from a combined analysis of morphometric traits. We also projected the data from the type series, including the lectotype of *P. impressus*, into the morphospace in an effort to associate the name with some recently sampled specimens and assign it to the obtained DNA-based species entities (Fig. 6). Given that in most analyses the morphospace of the 3 clades of the *P. melonii* group overlapped, including with the plots of the syntype series of *P. impressus*, no robust resolution of the question was possible, to which of the three clades^22^ the name should refer. Therefore, the question about the identity of this species could only be resolved based on DNA-based evidence from the lectotype (see above).

**Fig. 7:**
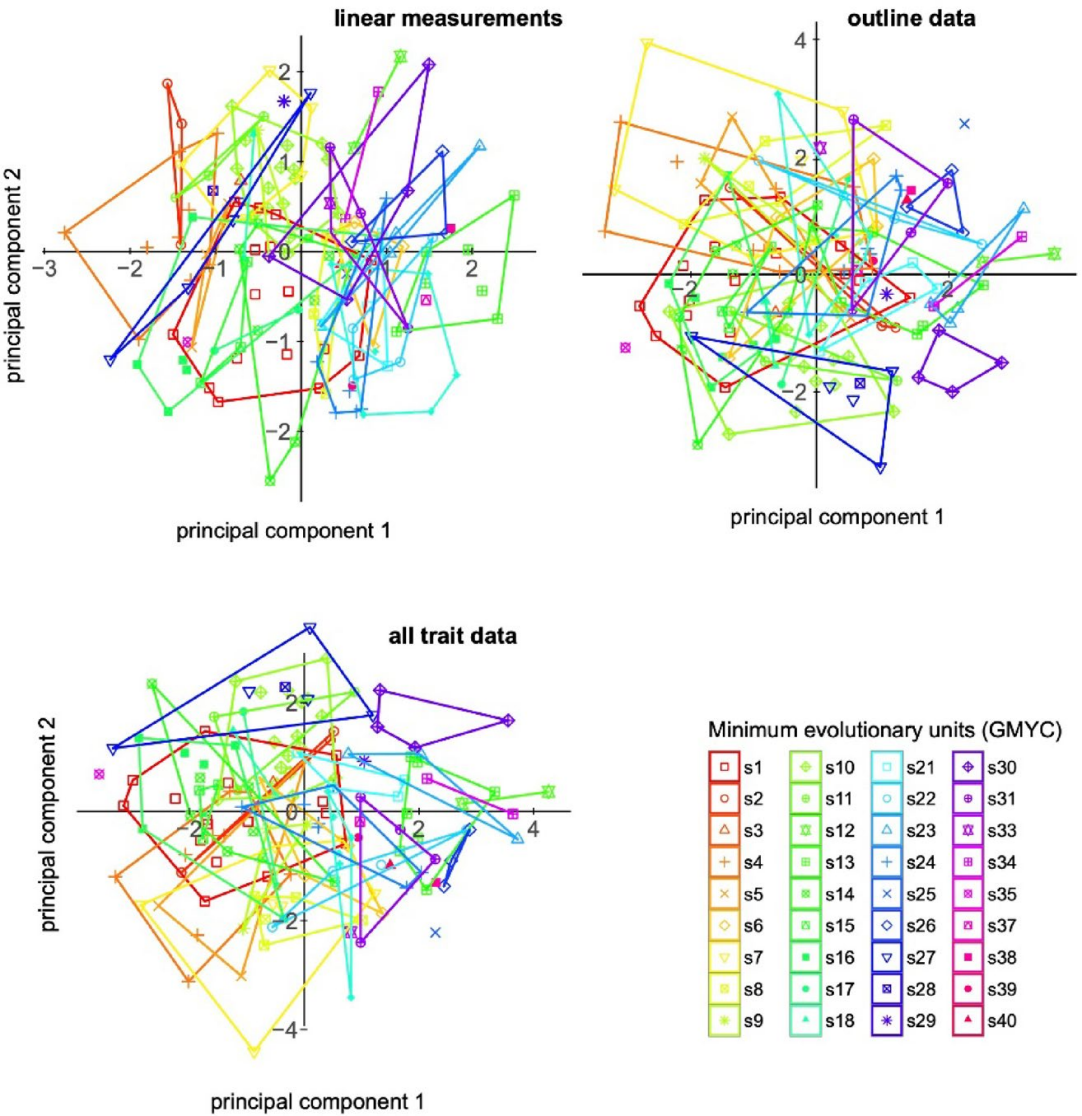
Major metapopulations (i.e., minimum evolutionary units) of *Pachypus* identified by Eberle et al. (2019) based on GMYC analysis of mainly mitochondrial data projected on the morphospace (plots showing axis 1 and 2) from linear size-corrected measurements combined outlines analysis, and all morphological trait data combined. The plots show the strong morphological divergence also between local populations, when specimen sampling is reduced.

How complicated the crypticity in *Pachypus* is, is not only shown by its taxonomic history, with most of the putative species being synonymized at some point with *P. candidae*^20^, but also by the morphometric study of Eberle et al.^5^. Their data revealed a distinctive morphological divergence between metapopulations (Fig. 7) exemplified by the minimum evolutionary units inferred from mitochondrial DNA and GMYC analysis. For a more comprehensive understanding of the taxonomy in *Pachypus*, we have re-illustrated these patterns here.

### Taxonomy

Based on species-boundary hypotheses derived from integrative analysis of mzl-USCO data^22^, we present here the formal descriptions of the newly discovered *Pachypus* species entities. Although morphology alone is insufficient to discriminate all species, we consider it essential to complement the primary diagnostic evidence (mzl-USCOs) with a morphological diagnosis (following^27^. A comprehensive morphological re-examination of the newly proposed *Pachypus* species entities revealed additional morphological traits that can help distinguish some species, at least in a limited geographical context.

### *Pachypus* Dejean, 1821

*Pachypus* Dejean, 1821: 57^28^ (type species by monotypy: *Scarabaeus excavatus* Fabricius, 1792)^30^; (nec *Pachypus* Billberg, 1820; nomen oblitum^29^.

*Caelodera* Dejean, 1833: 159^31^ (type species by monotypy: *Scarabaeus excavatus* Fabricius, 1792); unnecessary replacement name for *Pachypus* Dejean, 1821^30^.

**Diagnosis**. Antenna composed of eight antennomeres, antennal club composed of five, almost subequal antennomeres. Mentum narrow, strongly convex, with robust long setae. Labrum not visible, apparently absent. Mandibles reduced. Elytra and hind wings present and functional in males; scutellum, elytra, and hind wings entirely reduced in females. Pronotum little wider than long, widest shortly anterior to the middle, surface deeply concave in anterior two thirds of central pronotum; anterior margin strongly horn-like produced medially, without marginal line; basal margin weakly but convexly bent medially, with fine marginal line; posterior angles blunt; anterior angles little produced and sharp, their distance little smaller than maximum width of head; lateral margins strongly convex and densely covered with fine long yellow setae; surface very sparsely and finely punctate, glabrous. Scutellum semicircular, short, little wider than long, sparsely superficially punctate on lateral base. Venter with six ventrites, densely and finely punctate, sutures apically broadly membranous; ventrites and tergites including propygidium separated by membranous sutures; spiracles located in membranes, distal pair of spiracles enlarged. Legs robust, moderately long. Procoxae conical, contiguous, with a transverse ridge. Protibia short, bidentate, apical spur robust and long. Mesocoxae contiguous. Mesotibia and metatibia each with one transverse carina behind the middle, each with two apical spurs. Metatibial spurs at metatibial apex widely separated; apex of metatibia on mesal face concavely excavated between them. Pygidium subtriangular, longer than wide, weakly convex, with rounded apex; position directed 45° to main dorso-ventral body axis; surface smooth and shiny, basally with fine, moderately dense punctures and long setae, on apical half almost impunctate and glabrous. All tarsal claws symmetrical and simple, not cleft and without a basal tooth.

**Remarks**. Reversal of precedence (Article 23.9^32^) was proposed by Bouchard et al^29^. for *Pachypus* Dejean, 1821 nomen protectum over *Pachypus*Billberg, 1820 (nomen oblitum). Bouchard et al^29^. provided 25 supporting references for the conservation of *Pachypus* Dejean, 1821 over *Pachypus* Billberg, 1820 (Art. 23.9.2^32^). The genus name *Pachypus* Billberg, 1820 has not been used as valid after 1899^29^. The type species is defined by monotypy^30^. The genus was established based on indication without description or diagnosis^28^. Dejean^28^ included in the first note on *Pachypus* two other names, of which one was a nomen nudum (*Pachypus truncatifrons*, see^33^ and the second, *Melolontha cornuta* Olivier, apparently considered as conspecific with *Pachypus excavatus.*

### *Pachypus caesus Erichson*, 1840

*Pachypus caesus* Erichson, 1840: 35^18^; Luigioni 1923: 62^34^; Bezdek 2016: 249^20^.

*Pachypus caesus* var. *intermedia* Ragusa, 1893: 205^35^.

*Pachypus caesus* var. *intermedius*: Luigioni 1923: 62^34^.

*Pachypus intermedius*: Bezdek 2016: 249^20^.

*Pachypus siculus* Laporte, 1840: 129^36^.

**Additional material examined**. *(ID based on mzl-USCOs)*: 1 ♂ “X-DA2505 Italy, Sicilia: Catania Piazza S. Maria di Gesu, 6.x.2009, leg. A. Marletta Pachypus caesus” (ZFMK), 1 ♂ “X-DA2506 Italy, Sicilia: Catania Villa Bellini, 4.x.2006, leg. M.T. Spena Pachypus caesus” (ZFMK), 1 ♂ “X-DA2560 Italy Catania (CT): Giardino Bellini 8.VIII.2010 M.T. Spena” (ZFMK), 1 ♂ “X-DA2561 Italy Catania (CT): Giardino Bellini 9.VIII.2010 A. Marletta” (ZFMK), 1 ♂ “X-DA2562 Italy Sicilia (PA) Cefalù 30.IX.2003 I. Sparacio” (ZFMK), 1 ♂ “X-DA3481/Sicilia, Manfredia, 17.ix.1997, leg. L. Marsik Pachypus caesus” (ZFMK).

**Additional material examined**. *(identification based on IUMG (= i*ntegrative analysis of *U*SCOs and *m*orphological evidence considering also occurrences of *g*eographically exclusively occurring species inferred by USCOs, see Methods for criteria): see Supplement File 1.

**Redescription of male** (DA2561): Maximum body length: 15.0 mm, elytral length: 8.5 mm, maximum width: 8.0 mm.

**Color**: Head, pronotum and scutellum black, shiny, elytra black, with dull tomentum, humeri weakly shiny; ventral surface black, shiny. Dorsal surface glabrous except on densely and erectly setose frons; ventral surface with dense and long, yellowish pale setae; abdomen and pygidium black, sparsely punctate and setose.

**Head**: Clypeus semicircular, surface strongly concave and densely, finely punctate, glabrous. Antenna completely black, antennal club short, straight, club length distinctly shorter than remaining antennomeres combined. Eyes small, ratio maximal diameter/interocular width: 0.6. Ocular canthus moderately wide and short, slightly evenly narrowed towards apex, apex rounded, densely punctate and setose; 1/4 of maximal ocular diameter; ocular canthus lower than frons and separated from it by a supraocular carina. Area behind frontoclypeal suture deeply impressed, behind this impression with a long, weakly curved frontal carina, which is distinctly raised and complete; frons otherwise coarsely and densely punctate and covered with long, erect, dark brown setae.

**Elytra** elongate, subtriangular, strongly narrowed apically; surface with fine striae, intervals flat, with very sparse indistinct punctures and glabrous, second interval distinctly wider than the other ones. Epipleural margin present until apical rounding of elytra, lateral setae long (as long as metatarsal claw length), black. Ratio of length of metepisternum/metacoxa: 1/0.9.

**Legs**: Setae on metafemur pale yellowish, dense. Metatibia evenly widened towards apex; ratio maximal width/maximal length at dorsal margin: 1/1.4. Mesal face of metatibia in dorsoapical portion impunctate and glabrous. Meso- and metatarsomeres elongate, weakly widened posteriorly, impunctate dorsally, sparsely to densely setose ventrally, circular in cross section, metatarsomere 1 distinctly longer than following tarsomere.

**Fig. 8:**
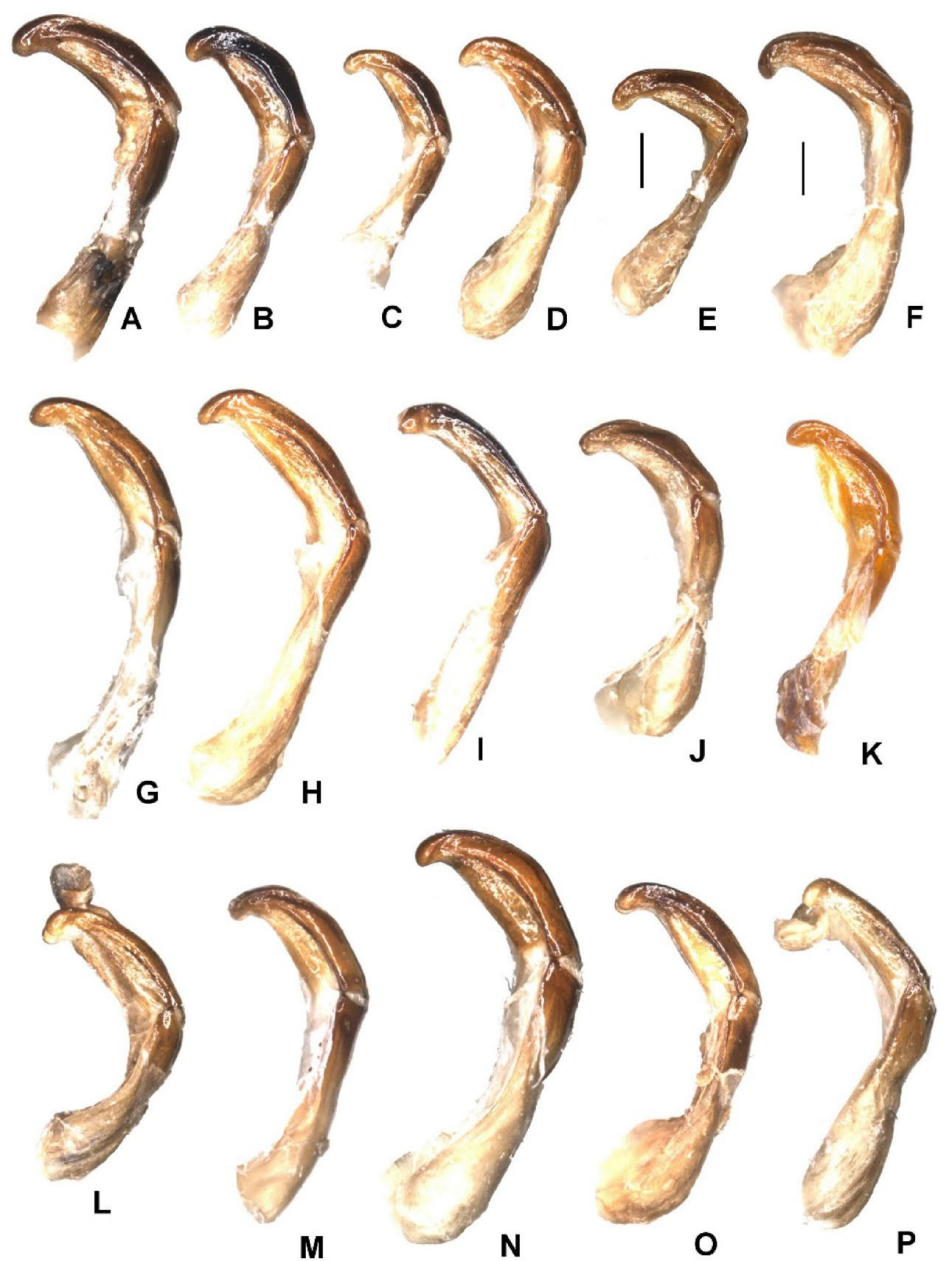
Aedeagus, lateral view. (**A**) - *Pachypus candidae* (DA2572), (**B**) - *P. franginii* sp. n. (holotype), (**C**) - *P. impressus* (836889), (**D**) - *P. matzaccara* sp. n. (holotype), (**E**) - *P. impressus* (lectotype), (**F**) - *P. sulcis* sp. n. (holotype), (**G**) - *P. demoflysi* (DA3999), (**H**) - *P. caesus* (DA2561), (**I**) - *P. sardiniensis* (paratype, DA3968), (**J**) - *P. cornutus* (neotype), (**K**) - *P. excavatus* (DA2573), (**L**) - *P. excavatus* (holotype), (**M**) - *P. gallurensis* sp. n. (holotype), (**N**) - *P. pelegrinus* sp. n. (holotype), (**O**) - *P. baroniensis* sp. n. (holotype), (**P**) - *P. occidentalis* sp. n. (holotype).

**Fig. 9:**
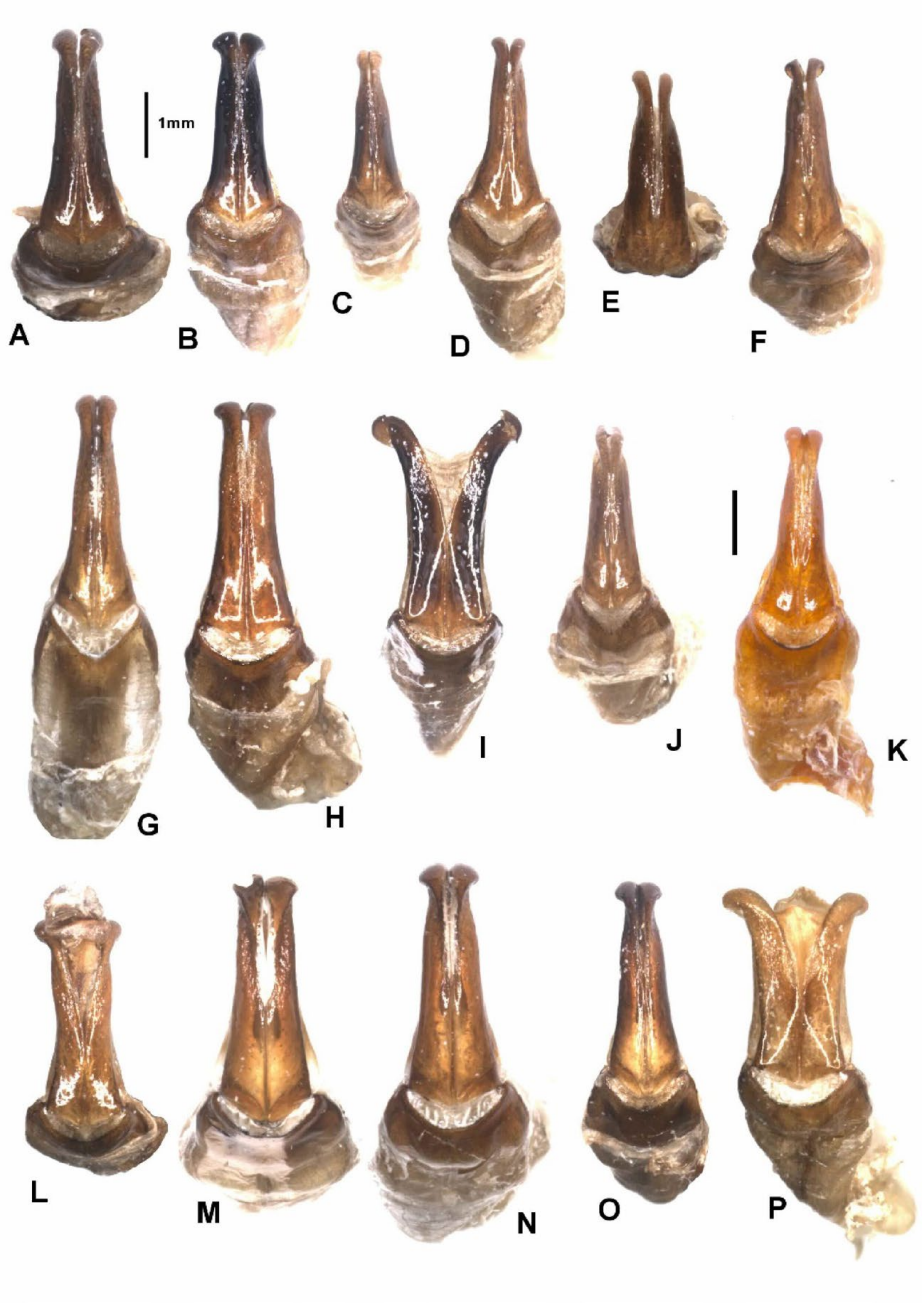
Parameres, dorsal view. (**A**) - *Pachypus candidae* (DA2572), (**B**) - *P. franginii* sp. n. (holotype), (**C**) - *P. impressus* (836889), (**D**) - *P. matzaccara* sp. n. (holotype), (**E**) - *P. impressus* (lectotype), (**F**) - *P. sulcis* sp. n. (holotype), (**G**) - *P. demoflysi* (DA3999), (**H**) - *P. caesus* (DA2561), (**I**) - *P. sardiniensis* (paratype, DA3968), (**J**) - *P. cornutus* (neotype), (**K**) - *P. excavatus* (DA2573), (**L**) - *P. excavatus* (holotype), (**M**) - *P. gallurensis* sp. n. (holotype), (**N**) - *P. pelegrinus* sp. n. (holotype), (**O**) - *P. baroniensis* sp. n. (holotype), (**P**) - *P. occidentalis* sp. n. (holotype).

**Fig. 10:**
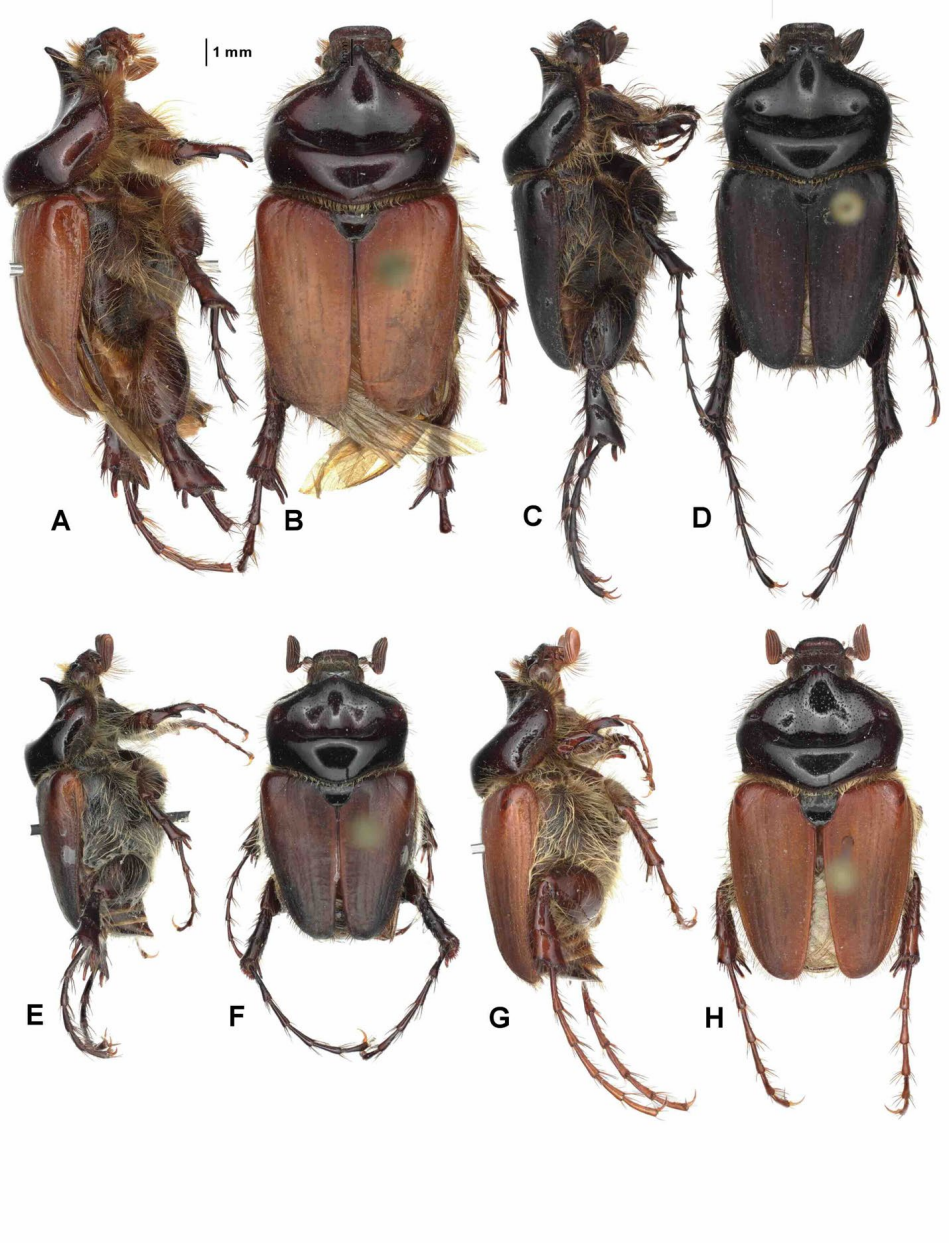
Habitus in dorsal (**B**,** D**,** F**,** H**) and lateral view (**A**,** C**,** E**,** G**).** A**,** B** – *Pachypus impressus* (lectotype),** C**,** D** – *P. sulcis* sp. n. (holotype),** E**,** F** – *P. demoflysi* (DA3999),** G**,** H** – *P. caesus* (DA2561).

Aedeagus: Figures 8H and 9H. Habitus: Fig. 10G, H.

**Variation**. The species is known only in its entirely black form.

### *Pachypus candidae* (Petagna, 1787)

*Scarabaeus candidae* Petagna, 1787: 3^37^.

*Pachypus candidae*: Luigioni 1923: 62^34^; Bezdek 2016: 249^20^.

**Additional material examined**. *(ID based on mzl-USCOs)*: 1 ♂ “X-DA2571 Italy Puglia: Fiume Lato 15.VI.2010 P. Fontana” (ZFMK), 1 ♂ “X-DA2572 Italy Puglia: Fiume Lato 15.VI.2010 P. Fontana” (ZFMK).

**Additional material examined**. *(identification based on IUMG*): see Supplement File 1.

**Redescription of male** (DA2572): Maximum body length: 15.0 mm, maximum width: 7.8 mm; elytral length: 7.8 mm.

**Color**: Head, pronotum, and scutellum dark reddish-brown and shiny, elytra reddish brown, with dull tomentum, humeri weakly shiny; ventral surface dark brown, shiny. Dorsal surface glabrous except on densely and erectly setose frons; ventral surface with dense and long, yellowish setae; abdomen and pygidium yellowish-brown and densely punctate and setose.

**Head**: Clypeus semicircular, surface strongly concave and densely punctate with fine and coarser punctures intermixed, glabrous. Antenna completely red-brown, antennal club little longer than remaining antennomeres combined. Eyes small, ratio maximal diameter/interocular width: 0.5. Ocular canthus long and evenly narrow, rounded at apex, densely punctate and setose; 1/3 of maximal ocular diameter; lower than frons and separated from it by a supraocular carina. Area behind frontoclypeal suture deeply impressed, behind this impression with a strongly curved but indistinct frontal carina; frons otherwise coarsely and densely punctate and covered with long erect brown setae.

**Elytra** elongate, subtriangular, strongly narrowed apically; surface with fine striae, intervals weakly convex, with very sparse indistinct punctures and glabrous, second interval wider than remaining ones. Epipleural margin present until apical rounding of elytra, lateral setae very short, longer only at apex, yellow or dark brown, laterally partly abraded. Ratio of length of metepisternum/metacoxa: 1/0.9.

**Legs**: Setae on metafemur pale yellowish, dense. Metatibia evenly widened towards apex; ratio maximal width/maximal length at dorsal margin: 1/1.2, mesal face along the middle impunctate, otherwise punctures large and ocellate, not granulose. Meso- and metatarsomeres elongate, weakly widened posteriorly, metatarsomeres sparsely but robustly punctate dorsally, each puncture bearing a robust short seta, sparsely to densely setose ventrally, circular in cross section, metatarsomere 1 distinctly longer than following tarsomere.

**Fig. 11:**
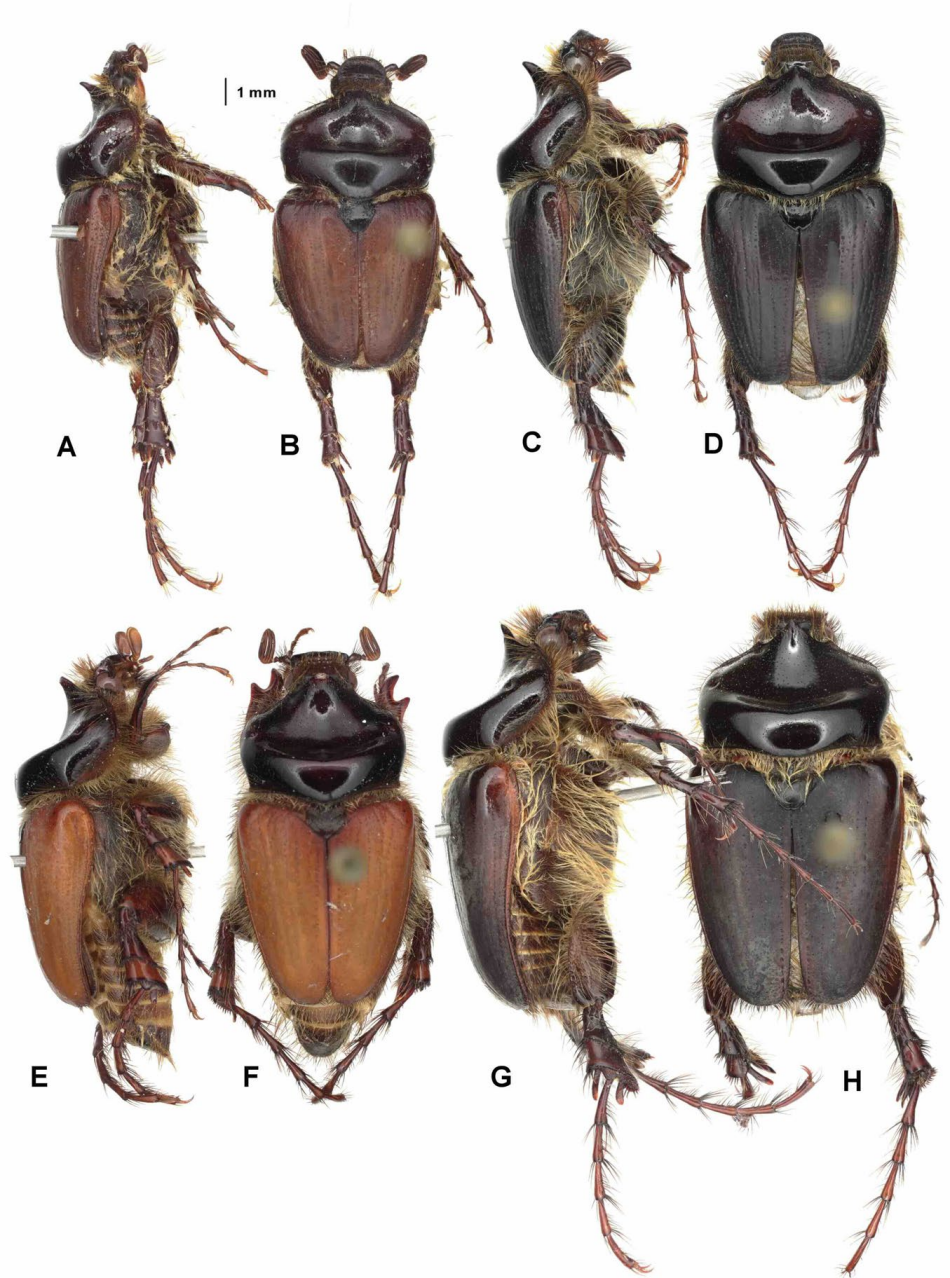
Habitus in dorsal (**B**,** D**,** F**,** H**) and lateral view (**A**,** C**,** E**,** G**).** A**,** B** - *Pachypus candidae* (DA2572),** C**,** D** – *P. franginii* sp. n. (holotype),** E**,** F** - *P. impressus* (836889),** G**,** H** – *P. matzaccara* sp. n. (holotype).

Aedeagus: Figures 8A and 9A. Habitus: Fig. 11A, B.

**Variation**. The color of elytra may vary from lighter yellowish brown to darker brown, but specimens are never uniformly black; the dark portion of the elytra is always indistinct, sometimes entirely absent.

**Remarks**. The infrasubspecific name *Pachypus candidae* ab. *caesicolor* Luigioni, 1923 is not available. The name was established to cover all black-colored specimens of “*P. candidae”* (without precise geographic indication) to distinguish them from black “*P. caesus”*. While on the first mentioning page (p. 56) there was no clear statement about the nature of the new form, at page 63 it is clearly stated “ab. *caesicolor*”. According to Article 45.6.2^32^. the name is therefore not available as its rank was clearly stated to be infrasubspecific. Subsequently also Crovetti^11^ used the name as “ab. *caesicolor*” in the majority of quotes in the text nominations, and the rare omissions of “ab.” are not to be interpretated as use as a subspecies.

### *Pachypus demoflysi* Normand, 1936

*Pachypus demoflysi* Normand, 1936: 376^38^; Bezdek 2016: 249^20^.

**Type material examined**. Holotype: ♂ “Cap Serrat (Tunisie) R. Demoflys 10.35/*Demoflysi* Norm./Type/*Pachypus Demoflysi* Normand/Prep. Sec interne n° P.5/Museum Paris 1993 Coll. J. Baraud” (MNHN).

**Additional material examined**. *(ID based on mzl-USCOs)*: 1 ♂ “X-DA3996/Tunisia, Gov. Beja Cap Serrat, 50 km E. Tabarka, 17.VI.2013, leg. G. Sabatinelli” (ZFMK), 1 ♂ “X-DA3997/Tunisia, Gov. Beja Cap Serrat, 50 km E. Tabarka, 17.VI.2013, leg. G. Sabatinelli” (ZFMK), 1 ♂ “X-DA3998/Tunisia, Gov. Beja Cap Serrat, 50 km E. Tabarka, 17.VI.2013, leg. G. Sabatinelli” (ZFMK), 1 ♂ “X-DA3999/Tunisia, Gov. Beja Cap Serrat, 50 km E. Tabarka, 17.VI.2013, leg. G. Sabatinelli” (ZFMK).

**Additional material examined**. *(identification based on IUMG*): 1 ♂ “Annaba Lac des Oiseaux A.L.+ 8.VII.1979/J.M. Meldes leg/Prép. Sac interne n° P.2/*Pachypus demoflysi* Norm. ab. *maldesi* nov. Baraud/Museum Paris 1993 Coll. J. Baraud” (MNHN), 1 ♂ “ALGERIE, Annaba est, Lac des Oisseaux 8.7.1979 S. Doguet/Prep sac interne N° P. 3/piege lumineux/*Pachypus demoflysi* Norm. ab. *maldesi* nov. Baraud/Museum Paris 1993 Coll. J. Baraud” (MNHN); see other material also Supplement File 1.

**Redescription of male** (DA3999): Maximum body length: 15.0 mm, maximum width: 6.9 mm; elytral length: 6.5 mm.

**Color**: Head, pronotum, and scutellum dark reddish-brown and shiny, elytra reddish brown, with dull tomentum, humeri shiny; ventral surface dark brown, shiny. Dorsal surface glabrous except on densely and erectly setose frons; ventral surface with dense and long, yellowish setae; abdomen and pygidium yellowish-brown and densely punctate and setose.

**Head**: Clypeus semicircular, surface strongly concave, surface densely punctate, fine and coarser punctures are mixed with each other, glabrous. Antenna completely brown, antennal club as long as remaining antennomeres combined. Eyes small, ratio maximal diameter/interocular width: 0.5. Ocular canthus long and evenly narrow, rounded at apex, densely punctate and setose; 1/3 of maximal ocular diameter; lower than frons and separated from it by a supraocular carina. Area behind frontoclypeal suture deeply impressed, behind this impression with a sharp and strongly curved frontal carina; frons otherwise coarsely and densely punctate and covered with long, erect, brown setae.

**Elytra** elongate, subtriangular, strongly narrowed apically; surface with fine striae, intervals weakly convex, with very sparse indistinct punctures and glabrous, second interval wider than remaining ones. Epipleural margin present until apical rounding of elytra, lateral setae short, posteriorly slightly longer, black. Ratio of length of metepisternum/metacoxa: 1/0.9.

**Legs**: Setae on metafemur pale yellowish, dense. Metatibia evenly widened towards apex; ratio maximal width/maximal length at dorsal margin: 1/1.5, dorsoapical portion of mesal face impunctate and glabrous, otherwise punctures large and ocellate, not granulose and with long setae. Meso- and metatarsomeres elongate, weakly widened posteriorly, metatarsomeres sparsely but robustly punctate dorsally, each puncture bearing a robust short seta, sparsely to densely setose ventrally, circular in cross section, metatarsomere 1 distinctly longer than following tarsomere.

Aedeagus: Figures 8G and 9G. Habitus: Fig. 10E, F.

**Variation**. The color of elytra may vary from reddish brown (without dark apical spot or margin) to black, pronotum dark brown to black.

**Remarks**. The name *Pachypus demoflysi* ab. *maldesi*, which Baraud^39^introduced for an entirely black specimen from northern Algeria, is not available according to Art. 45.5 and 45.6^32^. (see also^20^.

### *Pachypus excavatus* (Fabricius, 1792)

*Scarabaeus excavatus* Fabricius, 1792: 31^16^ (type locality: “regno neapolitano”, i.e., southern Italy except Sicily).

*Pachypus excavatus*: Feisthamel 1836: 159^40^; Erichson 1840: 34^18^; Eberle et al. 2019: 15^5^.

*Pachypus candidae* v. *erichsoni *Reitter, 1899: 41^41^.

**Type material examined**. Lectotype (*excavatus*, here designated): ♂ “Type//Italia Schlanbusch Mus. S. & T. L. Geotrupes excavatus. F./Type/Pachypus project PP0250” (ZMUK). Lectotype (*erichsoni*, here designated): ♂ “Italien Gaeta//v. Erichsoni m.//Holotypus Pachypus candidae v. Erichsoni Reitter 1898//coll. Reitter//Pachypus project PP0820” (HNHM).

**Additional material examined**. *(ID based on mzl-USCOs)*: 1 ♂ “836881 Pachypus candidae Italy, Lazio: Selva del Circeo, 5 km N of Sabaudia, 41°19’50,5’’N, 13°03’46,5’’E, 54 m, 15.07.2009, leg. D. Ahrens, S. Fabrizi” (ZFMK), 1 ♂ “836882 Pachypus candidae Italy, Lazio: Fonte di Lucullo (W Sabaudia), 41°15’26,0’’N, 13°03’41,2’’E, 0 m, 15.07.2009, leg. D. Ahrens, S. Fabrizi” (ZFMK), 1 ♂ “836883 Pachypus candidae Italy, Lazio: Fonte di Lucullo (W Sabaudia), 41°15’26,0’’N, 13°03’41,2’’E, 0 m, 15.07.2009, leg. D. Ahrens, S. Fabrizi” (ZFMK), 1 ♂ “836884 Pachypus candidae Italy, Lazio: Pineta di Torre Astura (S Nettuno), 41°24’49,7’’N, 12°45’30,6’’E, 10 m, 15.07.2009, leg. D. Ahrens, S. Fabrizi” (ZFMK), 1 ♂ “836885 Pachypus candidae Italy, Lazio: Pineta di Torre Astura (S Nettuno), 41°24’49,7’’N, 12°45’30,6’’E, 10 m, 15.07.2009, leg. D. Ahrens, S. Fabrizi” (ZFMK), 1 ♂ “836886 Pachypus candidae Italy, Lazio: W of Lago di Sabaudia, 41°17’05,3’’N, 13°03’42,0’’E, 21 m, 15.07.2009, leg. D. Ahrens, S. Fabrizi” (ZFMK), 1 ♂ “DNA voucher BMNH 837883/Italia: Lazio: Castel di Guido, vi.2000, leg. D. Ahrens” (ZFMK), 1 ♂ “DNA voucher BMNH 837884/Italia: Lazio: Castel di Guido, vi.2000, leg. D. Ahrens” (ZFMK), 1 ♂ “DNA voucher BMNH 837885/Italia: Lazio: Castel di Guido, vi.2000, leg. D. Ahrens” (ZFMK), 1 ♂ “DNA voucher BMNH 837886/Italia: Lazio: Castel di Guido, vi.2000, leg. D. Ahrens” (ZFMK), 1 ♂ “X-DA2567 Italy Lazio: (Latina Prov.) Circeo National Park, Mt. Circeo, Quarto Freddo, Peretto (CONECOFOR SITE) 120 m 14.VII.2010 C. Cocciufa” (ZFMK), 1 ♂ “X-DA2568 Italy Lazio: (Latina Prov.) Monti Ausoni, Sugherete di San Vito e Valle Marina, San Vito 70 m, 13.VII.2010 G. Carpaneto” (ZFMK), 1 ♂ “X-DA2569 Italy Lazio: (Latina Prov.) Monti Ausoni, Sugherete di San Vito e Valle Marina, San Vito 70 m, 13.VII.2010 G. Carpaneto” (ZFMK), 1 ♂ “X-DA2570 Italy Lazio: (Latina Prov.) Monti Ausoni, Sugherete di San Vito e Valle Marina, San Vito 70 m, 13.VII.2010 G. Carpaneto” (ZFMK), 1 ♂ “X-DA2573 Italy Lazio: (Frosinone Prov.) Bosco Polverino 33 T 348990E 4588774 N 40 m 11.VIII.2010 G. Carpaneto” (ZFMK), 1 ♂ “X-DA2574 Italy Lazio: (Frosinone Prov.) Bosco Polverino 33 T 348990E 4588774 N 40 m 11.VIII.2010 G. Carpaneto” (ZFMK), 1 ♂ “X-DA2575 Italy Lazio: (Frosinone Prov.) Bosco Polverino 33 T 348990E 4588774 N 40 m 11.VIII.2010 G. Carpaneto” (ZFMK), 1 ♂ “X-DA2576 Italy Lazio: (Frosinone Prov.) Bosco Polverino 33 T 348990E 4588774 N 40 m 11.VIII.2010 G. Carpaneto” (ZFMK), 1 ♂ “X-DA2582 Italy Torre Gianola Scauri, 6 km E Formia 6.VII.2010 D. Ahrens” (ZFMK), 1 ♂ “X-DA2583 Italy Torre Gianola Scauri, 6 km E Formia 6.VII.2010 D. Ahrens” (ZFMK), 1 ♂ “X-DA2729 Italy Lazio: Castel di Guido 41.902788°N; 12.306747°E 21.VII.2011 S. Fabrizi & D. Ahrens” (ZFMK), 1 ♂ “X-DA2730 Italy Lazio: Castel di Guido 41.902788°N; 12.306747°E 21.VII.2011 S. Fabrizi & D. Ahrens” (ZFMK), 1 ♂ “X-DA4232 - Italy, Lazio: (RM), Sughereta di Pomezia, 21.VI.2013, leg. F. Turchetti leg.” (ZFMK), 1 ♂ “X-DA4233 - Italy, Lazio: (RM), Sughereta di Pomezia, 21.VI.2013, leg. F. Turchetti leg.” (ZFMK).

**Additional material examined**. *(ID based on IUMG*): see Supplement File 1.

**Redescription of the lectotype **(*excavatus*): Maximum body length: 14.3 mm, maximum width: 6.5 mm; elytral length: 6 mm.

**Color**: Dorsum dark reddish-brown and shiny, elytra lighter, with dull tomentum, humeri weakly shiny; ventral surface reddish brown, shiny. Dorsal surface glabrous except on densely and erectly setose frons; ventral surface with dense and long yellow to brownish setae; abdomen and pygidium yellowish-brown and densely punctate and setose.

**Head**: Clypeus semicircular, surface strongly concave, surface densely punctate, fine and coarser punctures are mixed with each other, glabrous. Antenna completely yellow, composed of 8 antennomeres, antennal club composed of 5, almost subequal antennomeres, short, club length little shorter than remaining antennomeres combined. Eyes moderately large, ratio maximal diameter/interocular width: 0.6. Ocular canthus long and evenly narrow, rounded at apex, densely punctate and setose; 1/3 of maximal ocular diameter; lower than frons and separated from it by a supraocular carina. Area behind frontoclypeal suture deeply impressed, behind this impression with a long, weakly curved frontal carina, which is more strongly raised medially; frons otherwise coarsely and densely punctate and covered with long erect yellow setae. Mentum narrow, strongly convex, with robust long setae. Labrum not externally visible (apparently absent). Mandibles reduced.

**Elytra** elongate, subtriangular, strongly narrowed apically; surface with fine striae, intervals weakly convex, with very sparse indistinct punctures and glabrous, even intervals distinctly wider than odd ones. Epipleural margin present until apical rounding of elytra, lateral setae only present at apex, long and dark brown, laterally abraded. Hind wings present. Ratio of length of metepisternum/metacoxa: 1.02/1.

**Legs**: Robust, moderately long. Procoxae conical, contiguous, with a transverse ridge. Protibia short, bidentate, apical spur robust and long. Mesocoxae contiguous. Mesotibia and metatibia each with 1 transverse carina behind the middle, each with two apical spurs. Metatibia evenly widened towards apex; ratio maximal width/maximal length at dorsal margin: 1/1.63. Metatibial spurs at metatibial apex widely separated; metatibia concavely excavated between them. Meso- and metatarsomeres lacking in lectotype, distal tarsomeres of protarsomeres as well.

**Fig. 12:**
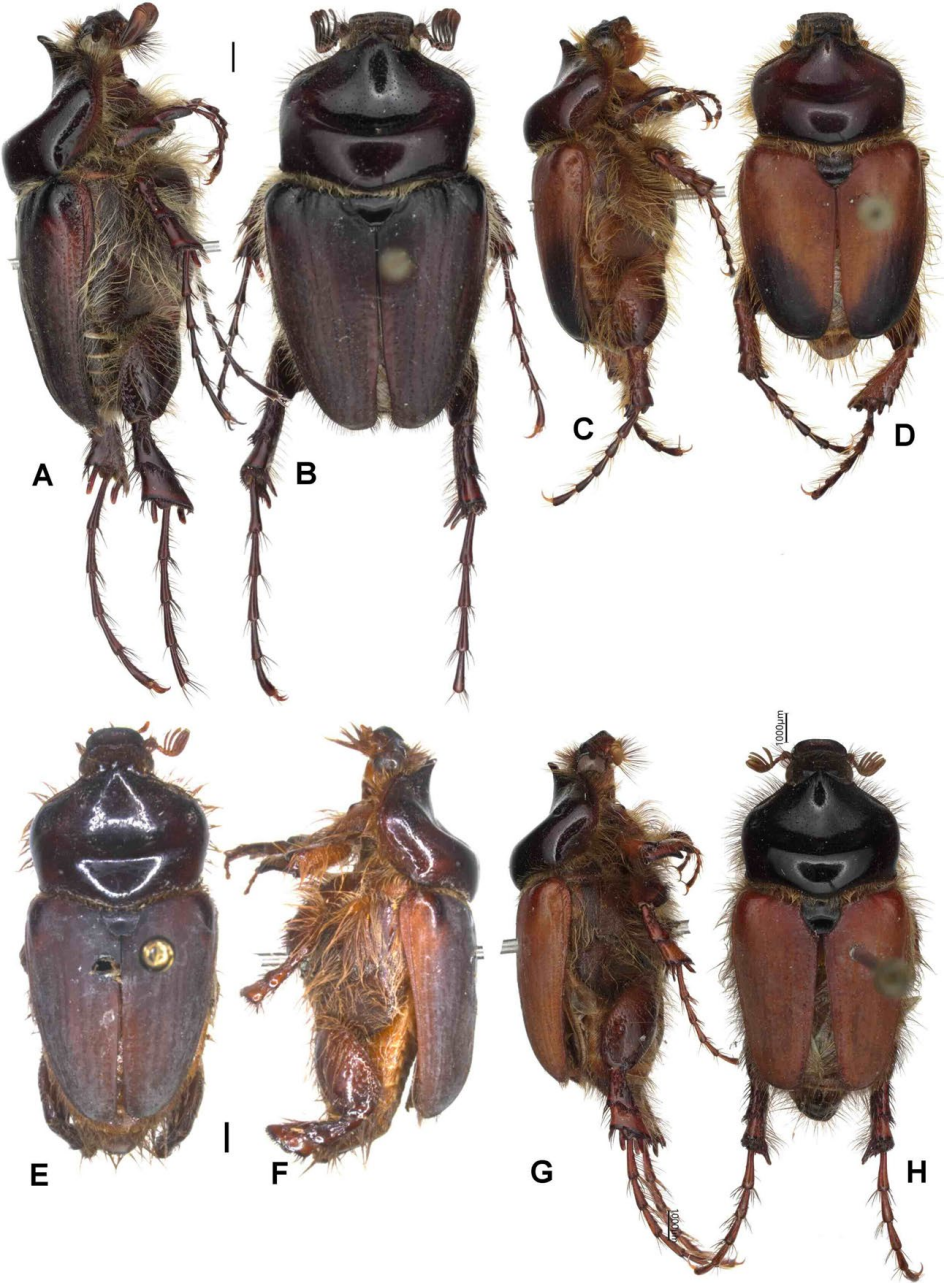
Habitus in dorsal (**B**,** D**,** E**,** H**) and lateral view (**A**,** C**,** F**,** G**).** A**,** B** - *Pachypus sardiniensis* (paratype, DA3968),** C**,** D** – *P. cornutus* (neotype),** E**,** F** – *P. excavatus* (holotype),** G**,** H** - *P. excavatus* (DA2573).

Aedeagus: Figures 8L and 9L. Habitus: Fig. 12E, F.

**Variation**. The color of elytra may vary from reddish brown (without dark apical spot or margin) to black, pronotum dark brown to black.

**Remarks**. Since no specimen number was given in the original descriptions of *Scarabaeus excavatus* Fabricius, 1792 and *Pachypus candidae* v. *erichsoni* Reitter, 1899, were designated here lectotypes for both names.

*Pachypus candidae* v. *erichsoni* was explicitly named as “*candidae* v. *erichsoni*”; in this case the name is potentially available according to Article 45.6.4^32^., since the content of the work did not unambiguously indicate that the name was proposed for an infrasubspecific entity.

Since Reitter^41^, *Pachypus excavatus* was considered to be a junior synonym of *Pachypus candidae* Petagna, 1787. This opinion was followed by all subsequent authors. However, already Erichson^18^ highlighted the taxonomic uncertainty regarding this species which was according to his notes apparently misinterpreted by several authors^40,42^. After first intensive morphometric and molecular investigations as well as based on the morphological examination of the type specimen, Eberle et al.^5^ raised the taxon to a valid species again.

We were able to identify the specimens from Campania (southern Appennine, Italy) to belong to *P. excavatus* using RADseq data, which we examined in an additional methodological study to compare mzl-USCOs with RADseq data (Becher et al., in preparation).

### *Pachypus sulcis* sp. n

LSID urn: lsid: zoobank.org: act:6A0589C0-F35F-43CC-877E-700C68361DB3.

**Type material examined**. *(ID based on mzl-USCOs)*: Holotype: ♂ “X-DA4273 - Italy, Sardegna: S. Margherita di Pula, 15.VI.2013, leg. Alamanni” (ZFMK). Paratypes: 1 ♂ “X-DA3417 - Italy, Sardegna: Torre Chia (Camping), 38°53’57.5’’N, 08°53’10.0’’E, 28.6.−1.7.2012, leg. D. Ahrens & S. Fabrizi” (ZFMK), 1 ♂ “X-DA3418 - Italy, Sardegna: Torre Chia (Camping), 38°53’57.5’’N, 08°53’10.0’’E, 28.6.−1.7.2012, leg. D. Ahrens & S. Fabrizi” (ZFMK), 1 ♂ “X-DA3420 - Italy, Sardegna: Torre Chia (Camping), 38°53’57.5’’N, 08°53’10.0’’E, 28.6.−1.7.2012, leg. D. Ahrens & S. Fabrizi” (ZFMK), 1 ♂ “X-DA3421 - Italy, Sardegna: Torre Chia (Camping), 38°53’57.5’’N, 08°53’10.0’’E, 28.6.−1.7.2012, leg. D. Ahrens & S. Fabrizi” (ZFMK), 1 ♂ “X-DA3423 - Italy, Sardegna: Torre Chia (Camping), 38°53’57.5’’N, 08°53’10.0’’E, 28.6.−1.7.2012, leg. D. Ahrens & S. Fabrizi” (ZFMK), 1 ♂ “X-JE0061/Sardinia, Pula, 01.06.87, leg. Hadulla” (ZFMK), 1 ♂ “X-JE0062/Sardinia, Pula, 23.05.1987, leg. Hadulla” (ZFMK).

Paratypes *(ID based on IUMG*): see Supplement File 1.

**Description of holotype**: Maximum body length: 12.9 mm, elytral length: 6.9 mm, maximum width: 7.3 mm.

**Color**: Head and pronotum black, shiny, some portions of pronotum dark brown, elytra black, with dull tomentum, humeri weakly shiny; ventral surface black to dark brown, shiny. Dorsal surface glabrous except on densely and erectly setose frons; ventral surface with dense and long yellow greyish setae; abdomen and pygidium black, sparsely punctate and setose.

**Head**: Clypeus semicircular, surface strongly concave, surface densely finely punctate, glabrous. Antenna completely black, antennal club moderately long, slightly reflexed, club length distinctly longer than remaining antennomeres combined. Eyes small, ratio maximal diameter/interocular width: 0.5. Ocular canthus wide and moderately long, slightly evenly narrowed towards apex, apex rounded, densely punctate and setose; 1/3 of maximal ocular diameter; ocular canthus lower than frons and separated from it by a supraocular carina. Area behind frontoclypeal suture deeply impressed, behind this impression with a long, weakly curved frontal carina, which is evenly raised; frons otherwise coarsely and densely punctate and covered with long erect yellow setae.

**Elytra** elongate, subtriangular, strongly narrowed apically; surface with fine striae, intervals flat, with very sparse indistinct punctures and glabrous, even intervals distinctly wider than odd ones. Epipleural margin present until apical rounding of elytra, lateral setae short or long (subequal to metatarsal claw length), light brown, posteriorly also often dark. Ratio of length of metepisternum/metacoxa: 1.

**Legs**: Setae on metafemur light grey to yellowish brown, dense. Metatibia evenly widened towards apex; ratio maximal width/maximal length at dorsal margin: 1/1.72. Meso- and metatarsomeres elongate, weakly widened posteriorly, impunctate dorsally, sparsely to densely setose ventrally, circular in cross section, metatarsomere 1 distinctly longer than following tarsomere.

Aedeagus: Figures 8F and 9F. Habitus: Fig. 10C, D.

**Diagnosis**. *Pachypus sulcis* sp. n. is very similar to *P. impressus*. It differs from the latter only by the entirely black elytra (in most *impressus* uniformly dark brown), and the slightly more distally extended coarse punctation on mesal and lateral face of the metatibia.

**Etymology**. The new species was named after the Sardinian geographical region Sulcis, where the species occurs (adjective in nominative case singular).

**Variation**. Maximum body length: 9.8–12.9 mm, elytral length: 5.3–6.9 mm, maximum width: 5.0–7.3 mm. The color of pronotum may vary from black to dark reddish brown, and the elytra can be dark brown. Female unknown.

**Remarks.** Dietz et al.^22^ referred to this species as *Pachypus melonii* (clade 1) or *P. melonii1*.

### *Pachypus matzaccara* sp. n

LSID urn: lsid: zoobank.org: act: B3E7AEEA-2E5B-4799-99BE-25EBFAF8292E.

**Type material examined**. *(ID based on mzl-USCOs)*: Holotype: ♂ “X-DA4234 - Italy, Sardinia: Matzaccara, 23.VI.2014, leg. D. Cillo & E. Bazzato” (ZFMK). Paratypes: 1 ♂ “X-DA2585 Italy Sardegna: Porto Pino env. (3 km S) 19.VII.2010 D. Ahrens” (ZFMK), 1 ♂ “X-DA4235 - Italy, Sardinia: Matzaccara, 23.VI.2014, leg. D. Cillo & E. Bazzato” (ZFMK), 1 ♂ “X-DA4236 - Italy, Sardinia: Matzaccara, 23.VI.2014, leg. D. Cillo & E. Bazzato” (ZFMK), 1 ♂ “X-DA4237 - Italy, Sardinia: Matzaccara, 23.VI.2014, leg. D. Cillo & E. Bazzato” (ZFMK).

Paratypes *(ID based on IUMG*): see Supplement File 1.

**Description of holotype**: Maximum body length: 12.6 mm, elytral length: 7.0 mm, maximum width: 6.8 mm.

**Color**: Pronotum and scutellum black, shiny, head and some portions of pronotum dark brown, elytra entirely reddish brown, with dull tomentum, humeri weakly shiny; ventral surface brown, shiny. Dorsal surface glabrous except on densely and erectly setose frons; ventral surface with dense and long yellow-greyish setae; abdomen and pygidium black, sparsely punctate and setose.

**Head**: Clypeus semicircular, surface strongly concave, surface densely finely punctate, glabrous. Antenna dark brown, antennal club moderately long, slightly reflexed, club length distinctly longer than remaining antennomeres combined. Eyes small, ratio maximal diameter/interocular width: 0.58. Ocular canthus wide and moderately long, slightly evenly narrowed towards apex, apex rounded, densely punctate and setose; 1/3 of maximal ocular diameter; ocular canthus lower than frons and separated from it by a supraocular carina. Area behind frontoclypeal suture deeply impressed, behind this impression with a long, weakly curved frontal carina, which is evenly raised; frons otherwise coarsely and densely punctate and covered with long erect yellow setae.

**Elytra** elongate, subtriangular, strongly narrowed apically; surface with fine striae, intervals flat, with very sparse indistinct punctures and glabrous, even intervals distinctly wider than odd ones. Epipleural margin present until apical rounding of elytra, lateral setae short or long (subequal to metatarsal claw length), light brown, posteriorly also some often a little darker. Ratio of length of metepisternum/metacoxa: 1/0.87.

**Legs**: Setae on metafemur light grey to yellowish brown, dense. Metatibia evenly widened towards apex; ratio maximal width/maximal length at dorsal margin: 1/1.61. Meso- and metatarsomeres elongate, weakly widened posteriorly, impunctate dorsally, sparsely to densely setose ventrally, circular in cross section, metatarsomere 1 distinctly longer than following tarsomere.

Aedeagus: Figures 8D and 9D. Habitus: Fig. 11G, H.

**Diagnosis**. *Pachypus matzaccara* sp. n. is very similar to *P. impressus* and cannot be reliably distinguished by morphology. The holotype differs from the lectotype of the latter only by the slightly more distally extended coarse punctation on mesal and lateral face of the metatibia.

**Etymology**. The new species name is derived from the name of its type locality, Matzaccara (noun in apposition).

**Variation**. Maximum body length: 11.9–12.6 mm, elytral length: 5.9–7.0 mm, maximum width: 5.7–6.8 mm. The color of elytra is in some paratypes lighter reddish brown (always without dark apical spot or margin), in some dark brown. Female unknown.

**Remarks.** Dietz et al.^22^ referred to this species as *Pachypus melonii* (clade 2) or *P. melonii2*.

### *Pachypus impressus* Erichson, 1840

*Pachypus impressus* Erichson, 1840: 33^18^.

*Pachypus candidae* ab. *impressus*: Luigioni 1923: 63^34^.

*Pachypus melonii* Sparacio, 2008: 5^14^; Bezdek 2016: 249^20^; syn. n.

**Type material examined**. Lectotype (here designated, *P. impressus*): ♂ “Syntypus Pachypus impressus Erichson, 1840 labelled by MNHUB 2013/Hist.-Coll (Coleoptera) Nr. 11213 Pachypus impressus Er. Sardinia. Dahl. Zool. Mus. Berlin/Lectotypus Pachypus impressus Erichson, 1840 D. Cillo designed/Pachypus project Bazzato et al. PP0043” (ZMHB)/PP0043” (ZMHB). Paralectotypes (here designated, *P. impressus*): 1 ♂ “Syntypus Pachypus impressus Erichson, 1840 labelled by MNHUB 2013/impressus Erichs. excavatus Guir. Sardin. Dahl./11,213/Paralectotypus Pachypus impressus Erichson, 1840 D. Cillo designed/Pachypus project Bazzato et al. PP0042” (ZMHB)/PP0042” (ZMHB), 1 ♂ “Pachypus impressus Erichson, 1840 MNHUB Berlin/Hist.-Coll (Coleoptera) Nr. 11213 Pachypus impressus Er. Sardinia. Dahl. Zool. Mus. Berlin/Paralectotypus Pachypus impressus Erichson, 1840 D. Cillo designed/Pachypus project Bazzato et al. PP0044” (ZMHB)/PP0044” (ZMHB), 1 ♂ “Pachypus impressus Erichson, 1840 MNHUB Berlin/Hist.-Coll (Coleoptera) Nr. 11,213 Pachypus impressus Er. Sardinia. Dahl. Zool. Mus. Berlin/Paralectotypus Pachypus impressus Erichson, 1840 D. Cillo designed/Pachypus project Bazzato et al. PP0045” (ZMHB)/PP0045” (ZMHB), 1 ♂ “Syntypus Pachypus impressus Erichson, 1840 labelled by MNHUB 2013/Sardigné/Hist.-Coll (Coleoptera) Nr. 11213 Pachypus impressus Er. Sardinia. Dahl. Zool. Mus. Berlin/gehört möglicherweise zur historischen Serie Nr. 11214 von P. cornutus Er./PP0046” (ZMHB). Paratypes: (*P. melonii*) 1 ♂ “Paratypus Pachypus melonii Sparacio, 2008/Sardegna (CA) Assemini Flumini Mannu 21.VI.2007 lg. D. Cillo/Coll. M. Uliana/PP0324” (CMUC), 1 ♂ “Paratypus Pachypus melonii Sparacio, 2008/Italia Sardegna (CA) Assemini Flumini Mannu 16/VI/2001 legit D. Sechi/Coll. M. Uliana/PP0325” (CMUC), 1 ♂ “Paratypus Pachypus melonii Sparacio, 2008/Sardinia Assemini (CA) Rio Cixerri 8.VI.96 leg. D. Sechi/Coll. M. Uliana/PP0326” (CMUC), 1 ♂ “Paratypus Pachypus melonii Sparacio, 2008/Sardegna (CA) Assémini - Flúmini Mannu/10.VI.1993 legit Meloni C./Coll. M. Uliana/PP0327” (CMUC). (see Supplement file 1 for further material).

**Additional material examined**. *(ID based on mzl-USCOs)*: 1 ♂ “836887 - Italy, Sardinia: betw. Uta & Assemini (fiume Mannu), 39°16’30,2’’N, 8°59’26,1’’E, 3 m, 4.07.2009, leg. D. Ahrens, S. Fabrizi” (ZFMK), 1 ♂ “836888 - Italy, Sardinia: betw. Uta & Assemini (fiume Mannu), 39°16’30,2’’N, 8°59’26,1’’E, 3 m, 4.07.2009, leg. D. Ahrens, S. Fabrizi” (ZFMK), 1 ♂ “836889 - Italy, Sardinia: betw. Uta & Assemini (fiume Mannu), 39°16’30,2’’N, 8°59’26,1’’E, 3 m, 4.07.2009, leg. D. Ahrens, S. Fabrizi” (ZFMK), 1 ♂ “X-DA2563 Italy SARDEGNA (CA) Assemini 20.VI.2003 C. Meloni” (ZFMK), 1 ♂ “X-DA2564 Italy SARDEGNA (CA) Assemini 20.VI.2003 C. Meloni” (ZFMK), 1 ♂ “X-DA3973 - Italia, Sardinia: Assemini- CA, 20.v.2011, leg. D. Cillo” (ZFMK), 1 ♂ “X-DA3974 - Italia, Sardinia: Assemini- CA, 20.v.2011, leg. D. Cillo” (ZFMK).

**Additional material examined**. *(ID based on IUMG*): see Supplement File 1.

**Redescription of lectotype**: Maximum body length: 11.6 mm, elytral length: 6.0 mm, maximum width: 6.0 mm.

**Color**: Pronotum and scutellum black, shiny, head and some portions of pronotum dark brown, elytra entirely reddish brown, with dull tomentum, humeri weakly shiny; ventral surface brown, shiny. Dorsal surface glabrous except on densely and erectly setose frons; ventral surface with dense and long yellow greyish setae; abdomen and pygidium black, sparsely punctate and setose.

**Head**: Clypeus semicircular, surface strongly concave, surface densely finely punctate, glabrous. Antenna dark brown, antennal club moderately long, almost straight, club length distinctly longer than remaining antennomeres combined. Eyes small, ratio maximal diameter/interocular width: 0.52. Ocular canthus wide and moderately long, slightly evenly narrowed towards apex, apex rounded, densely punctate and setose; 1/3 of maximal ocular diameter; ocular canthus lower than frons and separated from it by a supraocular carina. Area behind frontoclypeal suture deeply impressed, behind this impression with a long, weakly curved frontal carina, which is evenly raised; frons otherwise coarsely and densely punctate and covered with long erect yellow setae.

**Elytra** elongate, subtriangular, strongly narrowed apically; surface with fine striae, intervals flat, with very sparse indistinct punctures and glabrous, even intervals distinctly wider than odd ones. Epipleural margin present until apical rounding of elytra, lateral setae short or long (subequal to metatarsal claw length), but particularly in lateral portion mostly abraded, light brown. Ratio of length of metepisternum/metacoxa: 1/0.89.

**Legs**: Setae on metafemur light yellowish brown, dense. Metatibia evenly widened towards apex; ratio maximal width/maximal length at dorsal margin: 1/1.48. Meso- and metatarsomeres elongate, weakly widened posteriorly, impunctate dorsally, sparsely to densely setose ventrally, circular in cross section, metatarsomere 1 distinctly longer than following tarsomere.

Aedeagus: Figures 8E and 9E. Habitus: Fig. 10A, B.

**Remarks**. Dietz et al^22^. referred to this species as *Pachypus melonii* (clade 3) or *P. melonii3*. According to Erichson^18^ the original specimens of *P. impressus* came from Corsica and Sardinia. However, we could retrieve only specimens from Sardinia from the ZMHB, where the collection of Erichson is preserved (with the catalogue number 11213; only these were listed in the ZMHB’s old collection’s catalogue in association with the name *P. impressus*). Of these, one specimen was selected here as lectotype. The lectotype was sequenced for mzl-USCO genes in order to correctly identify the species identity, given the cryptic character of most species in the genus and the lack of other evidence to reveal the type’s identity to one of the three *P. melonii* lineages^22^. The ML tree with the mzl-USCO genes confirmed its placement within clade *P. melonii3*, of which all investigated specimens originated from the type locality of the actual *P. melonii*. Consequently, *P. impressus* Erichson, 1840 and *P. melonii* Sparacio, 2008 have to be considered synonymous. Also, the extracted COI data confirmed its identity with the locus typicus populations (clade “*melonii3*”^22^) of *P. melonii* Sparacio, 2008.

### *Pachypus sardiniensis* Guerlach, Bazzato & Cillo, 2013

*Pachypus sardiniensis* Guerlach, Bazzato & Cillo, 2013: 74^15^; Bezdek 2016: 249^20^.

**Type material examined**. Paratypes: 1 ♂ “Paratypus Pachypus sardiniensis n. sp. G. Guerlach, E. Bazzato & D. Cillo/Sardegna Flumini - Quartu S.E. 6.04 leg. E. Bazzato/PP0079” (E. Bazzato, Quartu S.E., Italy); for additional type material see Supplement File 1.

**Additional material examined**. *(ID based on mzl-USCOs)*: 1 ♂ “836890 - Italy, Sardinia: Foce del Fiumendosa (Muravera), 39°23’42,9’’N, 9°26’38,5’’E, 0-78m, 4.07.2009, leg. D. Ahrens, S. Fabrizi” (ZFMK), 1 ♂ “836891 - Italy, Sardinia: Foce del Fiumendosa (Muravera), 39°23’42,9’’N, 9°26’38,5’’E, 0-78m, 4.07.2009, leg. D. Ahrens, S. Fabrizi” (ZFMK), 1 ♂ “836892 - Italy, Sardinia: Foce del Fiumendosa (Muravera), 39°23’42,9’’N, 9°26’38,5’’E, 0-78m, 4.07.2009, leg. D. Ahrens, S. Fabrizi” (ZFMK), 1 ♂ “836893 - Italy, Sardinia: Foce del Fiumendosa (Muravera), 39°23’42,9’’N, 9°26’38,5’’E, 0-78m, 4.07.2009, leg. D. Ahrens, S. Fabrizi” (ZFMK), 1 ♂ “836894 - Italy, Sardinia: Foce del Fiumendosa (Muravera), 39°23’42,9’’N, 9°26’38,5’’E, 0-78m, 4.07.2009, leg. D. Ahrens, S. Fabrizi” (ZFMK), 1 ♂ “836895 - Italy, Sardinia: Foce del Fiumendosa (Muravera), 39°23’42,9’’N, 9°26’38,5’’E, 0-78m, 4.07.2009, leg. D. Ahrens, S. Fabrizi” (ZFMK), 1 ♂ “836896 - Italy, Sardinia: Foce del Fiumendosa (Muravera), 39°23’42,9’’N, 9°26’38,5’’E, 0-78m, 4.07.2009, leg. D. Ahrens, S. Fabrizi” (ZFMK), 1 ♂ “836897 - Italy, Sardinia: Foce del Fiumendosa (Muravera), 39°23’42,9’’N, 9°26’38,5’’E, 0-78m, 4.07.2009, leg. D. Ahrens, S. Fabrizi” (ZFMK), 1 ♂ “836898 - Italy, Sardinia: Foce del Fiumendosa (Muravera), 39°23’42,9’’N, 9°26’38,5’’E, 0-78m, 4.07.2009, leg. D. Ahrens, S. Fabrizi” (ZFMK), 1 ♂ “836899 - Italy, Sardinia: Foce del Fiumendosa (Muravera), 39°23’42,9’’N, 9°26’38,5’’E, 0-78m, 4.07.2009, leg. D. Ahrens, S. Fabrizi” (ZFMK), 1 ♂ “836900 - Italy, Sardinia: Foce del Fiumendosa (Muravera), 39°23’42,9’’N, 9°26’38,5’’E, 0-78m, 4.07.2009, leg. D. Ahrens, S. Fabrizi” (ZFMK), 1 ♂ “836901 - Italy, Sardinia: Foce del Fiumendosa (Muravera), 39°23’42,9’’N, 9°26’38,5’’E, 0-78m, 4.07.2009, leg. D. Ahrens, S. Fabrizi” (ZFMK), 1 ♂ “X-DA2565 Italy SARDEGNA (CA) Muravera 2.VII.2003 I. Sparacio” (ZFMK), 1 ♂ “X-DA2566 Italy SARDEGNA (CA) Muravera 2.VII.2003 I. Sparacio” (ZFMK), 1 ♂ “X-DA3326 - Italy, Sardegna: Villasimius (Camping Spiaggia del Riso), 29°07’23.1’N [sic!; correct: 39°07’23.1’’N], 09°30’40.6’’E, 27.6.2012, leg. D. Ahrens & S. Fabrizi” (ZFMK), 1 ♂ “X-DA3327 - Italy, Sardegna: Villasimius (Camping Spiaggia del Riso), 29°07’23.1’N [sic!; correct: 39°07’23.1’’N], 09°30’40.6’’E, 27.6.2012, leg. D. Ahrens & S. Fabrizi” (ZFMK), 1 ♂ “X-DA3328 - Italy, Sardegna: Villasimius (Camping Spiaggia del Riso), 29°07’23.1’N [sic!; correct: 39°07’23.1’’N], 09°30’40.6’’E, 27.6.2012, leg. D. Ahrens & S. Fabrizi” (ZFMK), 1 ♂ “X-DA3329 - Italy, Sardegna: Villasimius (Camping Spiaggia del Riso), 29°07’23.1’N [sic!; correct: 39°07’23.1’’N], 09°30’40.6’’E, 27.6.2012, leg. D. Ahrens & S. Fabrizi” (ZFMK), 1 ♂ “X-DA3330 - Italy, Sardegna: Villasimius (Camping Spiaggia del Riso), 29°07’23.1’N [sic!; correct: 39°07’23.1’’N], 09°30’40.6’’E, 27.6.2012, leg. D. Ahrens & S. Fabrizi” (ZFMK), 1 ♂ “X-DA3331 - Italy, Sardegna: Villasimius (Camping Spiaggia del Riso), 29°07’23.1’N [sic!; correct: 39°07’23.1’’N], 09°30’40.6’’E, 27.6.2012, leg. D. Ahrens & S. Fabrizi” (ZFMK), 1 ♂ “X-DA3336 - Italy, Sardegna: Costa Rei, Piscina Rei env.: Camping “le Dune”, 39°16’29.7’’N, 09°35’08.2’’E, 2.7.−4.7.2012, leg. D. Ahrens & S. Fabrizi” (ZFMK), 1 ♂ “X-DA3337 - Italy, Sardegna: Costa Rei, Piscina Rei env.: Camping “le Dune”, 39°16’29.7’’N, 09°35’08.2’’E, 2.7.−4.7.2012, leg. D. Ahrens & S. Fabrizi” (ZFMK), 1 ♂ “X-DA3338 - Italy, Sardegna: Costa Rei, Piscina Rei env.: Camping “le Dune”, 39°16’29.7’’N, 09°35’08.2’’E, 2.7.−4.7.2012, leg. D. Ahrens & S. Fabrizi” (ZFMK), 1 ♂ “X-DA3971 - Italia, Sardinia: Torre delle Stelle, 4.vii.2012, leg. D. Cillo” (ZFMK).

**Additional material examined**. *(ID based on IUMG*): see Supplement File 1.

**Redescription of paratype**: Maximum body length: 14.4 mm, elytral length: 8.0 mm, maximum width: 7.5 mm.

**Color**: Head, pronotum and scutellum black, partly reddish dark brown, shiny, elytra black, with dull tomentum, humeri weakly shiny; ventral surface black, shiny. Dorsal surface glabrous except on densely and erectly setose frons; ventral surface with dense and long, yellowish pale setae; abdomen and pygidium black, sparsely punctate and setose.

**Head**: Clypeus semicircular, surface strongly concave, surface densely finely punctate, glabrous. Antenna completely black, antennal club long, reflexed, club length distinctly longer than remaining antennomeres combined. Eyes small, ratio maximal diameter/interocular width: 0.48. Ocular canthus wide and moderately long, slightly evenly narrowed towards apex, apex rounded, densely punctate and setose; 1/3 of maximal ocular diameter; ocular canthus lower than frons and separated from it by a supraocular carina. Area behind frontoclypeal suture deeply impressed, behind this impression with a long, weakly curved frontal carina, which is weakly raised and medially narrowly interrupted; frons otherwise coarsely and densely punctate and covered with long, erect, dark brown setae.

**Elytra** elongate, subtriangular, strongly narrowed apically; surface with fine striae, intervals flat, with very sparse indistinct punctures and glabrous, second interval distinctly wider than the other ones. Epipleural margin present until apical rounding of elytra, lateral setae apically shorter (shorter than metatarsal claw length), black. Ratio of length of metepisternum/metacoxa: 1/0.93.

**Legs**: Setae on metafemur pale yellowish, dense. Metatibia evenly widened towards apex; ratio maximal width/maximal length at dorsal margin: 1/1.48. Mesal face of metatibia in dorsoapical portion impunctate and glabrous. Meso- and metatarsomeres elongate, weakly widened posteriorly, impunctate dorsally, sparsely to densely setose ventrally, circular in cross section, metatarsomere 1 distinctly longer than following tarsomere.

Aedeagus: Figures 8I and 9I. Habitus: Fig. 12A, B.

**Variation**. Some specimens are entirely black (e.g., the holotype^15^).

**Remarks.** As the original description of the species was in French, we provided here an English redescription of one of the available paratype specimens.

### *Pachypus gallurensis* sp. n

LSID urn: lsid: zoobank.org: act:32A4D011-C3D3-4052-A521-519DDCBB3F9C.

**Type material examined. ***(ID based on mzl-USCOs)*: Holotype: ♂ “X-DA3394 - Italy, Sardegna: Cala Ginepro (Cala Liberotto), N. Orosei, 40°26’29.1’’N, 09°47’42.6’’E (Pachypus, lehmiger Boden), 17.−21.6.2012, leg. D. Ahrens & S. Fabrizi” (ZFMK). Paratypes: 1 ♂ “X-DA2725 Italy Sardinia: Cala Pineta, near St. Lucia (Nuoro), 12 m 40°34’07,7’’N; 09°47’17,1’’E 14.−14.VII.2011 S. Fabrizi & D. Ahrens” (ZFMK), 1 ♂ “X-DA2726 Italy Sardinia: Cala Pineta, near St. Lucia (Nuoro), 12 m 40°34’07,7’’N; 09°47’17,1’’E 14.−14.VII.2011 S. Fabrizi & D. Ahrens” (ZFMK), 1 ♂ “X-DA3382 - Italy, Sardegna: Caletta di Osalla (10 km S Orosei), on sand, 40°19’48.2’’N, 09°40’31.6’’E, 20.6.2012, leg. D. Ahrens & S. Fabrizi” (ZFMK), 1 ♂ “X-DA3383 - Italy, Sardegna: Caletta di Osalla (10 km S Orosei), on sand, 40°19’48.2’’N, 09°40’31.6’’E, 20.6.2012, leg. D. Ahrens & S. Fabrizi” (ZFMK), 1 ♂ “X-DA3384 - Italy, Sardegna: Caletta di Osalla (10 km S Orosei), on sand, 40°19’48.2’’N, 09°40’31.6’’E, 20.6.2012, leg. D. Ahrens & S. Fabrizi” (ZFMK), 1 ♂ “X-DA3385 - Italy, Sardegna: Caletta di Osalla (10 km S Orosei), on sand, 40°19’48.2’’N, 09°40’31.6’’E, 20.6.2012, leg. D. Ahrens & S. Fabrizi” (ZFMK), 1 ♂ “X-DA3392 - Italy, Sardegna: Cala Ginepro (Cala Liberotto), N. Orosei, 40°26’29.1’’N, 09°47’42.6’’E (Pachypus, lehmiger Boden), 17.−21.6.2012, leg. D. Ahrens & S. Fabrizi” (ZFMK), 1 ♂ “X-DA3393 - Italy, Sardegna: Cala Ginepro (Cala Liberotto), N. Orosei, 40°26’29.1’’N, 09°47’42.6’’E (Pachypus, lehmiger Boden), 17.−21.6.2012, leg. D. Ahrens & S. Fabrizi” (ZFMK), 1 ♂ “X-DA3395 - Italy, Sardegna: Cala Ginepro (Cala Liberotto), N. Orosei, 40°26’29.1’’N, 09°47’42.6’’E (Pachypus, lehmiger Boden), 17.−21.6.2012, leg. D. Ahrens & S. Fabrizi” (ZFMK), 1 ♂ “X-DA3396 - Italy, Sardegna: Cala Ginepro (Cala Liberotto), N. Orosei, 40°26’29.1’’N, 09°47’42.6’’E (Pachypus, lehmiger Boden), 17.−21.6.2012, leg. D. Ahrens & S. Fabrizi” (ZFMK).

**Additional type material examined.** *(ID based on IUMG*): see Supplement File 1.

**Description of holotype**: Maximum body length: 14.4 mm, elytral length: 7.5 mm, maximum width: 7.8 mm.

**Color**: Head and pronotum black, shiny, elytra black, with dull tomentum, humeri weakly shiny; ventral surface black to dark brown, shiny. Dorsal surface glabrous except on densely and erectly setose frons; ventral surface with dense and long brown setae which are often lighter at apex; abdomen and pygidium black, sparsely punctate and setose.

**Head**: Clypeus semicircular, surface strongly concave, surface densely finely punctate, glabrous. Antenna dark brown, antennal club moderately long, almost straight, club length as long as remaining antennomeres combined. Eyes small, ratio maximal diameter/interocular width: 0.53. Ocular canthus wide and moderately long, slightly evenly narrowed towards apex, apex rounded, densely punctate and setose; 1/3 of maximal ocular diameter; ocular canthus lower than frons and separated from it by a supraocular carina. Area behind frontoclypeal suture deeply impressed, behind this impression with a long, weakly curved frontal carina, which is evenly raised; frons otherwise coarsely and densely punctate and covered with long erect black setae.

**Elytra** elongate, subtriangular, strongly narrowed apically; surface with fine striae, intervals flat, with very sparse indistinct punctures and glabrous, even intervals distinctly wider than odd ones. Epipleural margin present until apical rounding of elytra, lateral setae short or long (subequal to metatarsal claw length), black, at apex brown. Ratio of length of metepisternum/metacoxa: 1/1.05.

**Legs**: Setae on metafemur black, at apex brown, dense. Metatibia evenly widened towards apex; ratio maximal width/maximal length at dorsal margin: 1/1.28. Meso- and metatarsomeres elongate, weakly widened posteriorly, impunctate dorsally, except a few robust punctures on metatarsomere 1, sparsely to densely setose ventrally, circular in cross section, metatarsomere 1 distinctly longer than following tarsomere.

**Fig. 13:**
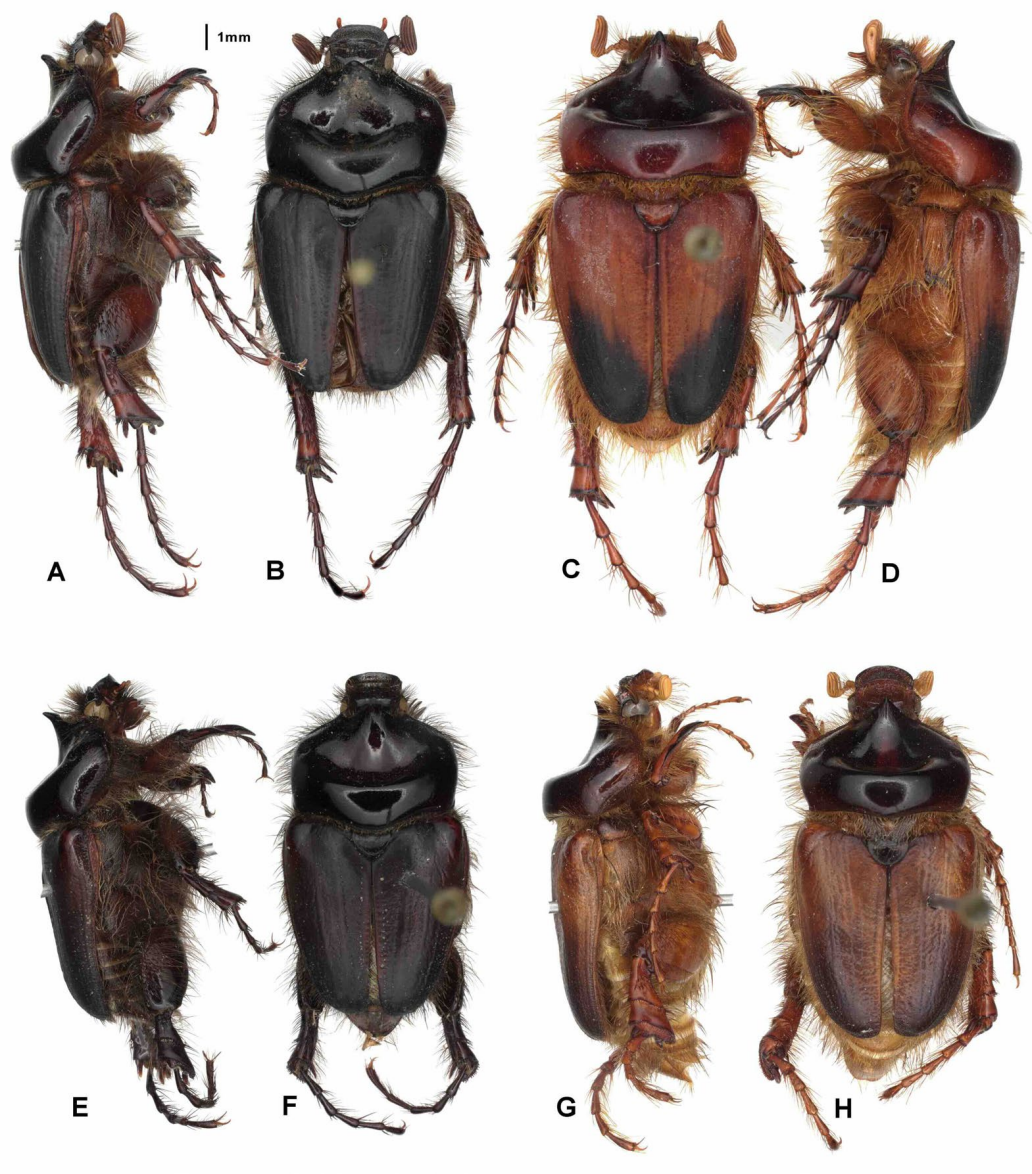
Habitus in dorsal (**B**,** D**,** F**,** H**) and lateral view (**A**,** C**,** E**,** G**).** A**,** B** - *Pachypus gallurensis* sp. n. (holotype),** C**,** D** – *P. pelegrinus* sp. n. (holotype),** E**,** F** – *P. baroniensis* sp. n. (holotype),** G**,** H** - *P. occidentalis* sp. n. (holotype).

Aedeagus: Figures 8M and 9M. Habitus: Fig. 13A, B.

**Diagnosis**. *Pachypus gallurensis* sp. n. is very similar to *P. sardiniensis*. It differs from the latter only by the slightly less reflexed antennal club in the male and the dark pilosity on ventral body surface and femora; also, the setae of the lateral margin of the elytra appear slightly longer and finer (dark in both species).

**Etymology**. The new species is named after the Sardinian region Gallura where the species occurs (adjective in nominative case).

**Variation**. Maximum body length: 11.5–14.4 mm, elytral length: 5.9–7.5 mm, maximum width: 5.7–7.8 mm. Some specimens with dark brown pronotum and elytra.

**Remarks**. Dietz et al^22^. referred to this species as *Pachypus* sp2a.

### *Pachypus baroniensis* sp. n

urn: lsid: zoobank.org: act: B4363B33-5279-4C19-8108-55A9CF6B75E7.

**Type material examined**. *(ID based on mzl-USCOs)*: Holotype: ♂ “X-DA2581 Italy SARDEGNA (OT) San Teodoro, Cala d’Ambra 32 T 557486E 4513314 N, 2 m, 9–15.VI.2010 G. Carpaneto” (ZFMK). Paratypes: 1 ♂ “X-DA2577 Italy SARDEGNA (OT) San Teodoro, Cala d’Ambra 32 T 557486E 4513314 N, 2 m, 9–15.VI.2010 G. Carpaneto” (ZFMK), 1 ♂ “X-DA2578 Italy SARDEGNA (OT) San Teodoro, Cala d’Ambra 32 T 557486E 4513314 N, 2 m, 9–15.VI.2010 G. Carpaneto” (ZFMK), 1 ♂ “X-DA2579 Italy SARDEGNA (OT) San Teodoro, Cala d’Ambra 32 T 557486E 4513314 N, 2 m, 9–15.VI.2010 G. Carpaneto” (ZFMK), 1 ♂ “X-DA2580 Italy SARDEGNA (OT) San Teodoro, Cala d’Ambra 32 T 557486E 4513314 N, 2 m, 9–15.VI.2010 G. Carpaneto” (ZFMK).

**Additional material examined**. *(ID based on IUMG*): see Supplement File 1.

**Description of holotype**: Maximum body length: 14.3 mm, elytral length: 7.1 mm, maximum width: 7.1 mm.

**Color**: Head and pronotum black, shiny, elytra black, with dull tomentum, humeri weakly shiny; ventral surface black to dark brown, shiny. Dorsal surface glabrous except on densely and erectly setose frons; ventral surface with dense and long brown setae which are often lighter at apex; abdomen and pygidium black, sparsely punctate and setose.

**Head**: Clypeus semicircular, surface strongly concave, surface densely finely punctate, glabrous. Antenna dark brown, antennal club moderately long, almost straight, club length as long as remaining antennomeres combined. Eyes small, ratio maximal diameter/interocular width: 0.5. Ocular canthus wide and moderately long, slightly evenly narrowed towards apex, apex rounded, densely punctate and setose; 1/3 of maximal ocular diameter; ocular canthus lower than frons and separated from it by a supraocular carina. Area behind frontoclypeal suture deeply impressed, behind this impression with a long, weakly curved frontal carina, which is evenly raised; frons otherwise coarsely and densely punctate and covered with long erect black setae.

**Elytra** elongate, subtriangular, strongly narrowed apically; surface with fine striae, intervals flat, with very sparse indistinct punctures and glabrous, even intervals distinctly wider than odd ones. Epipleural margin present until apical rounding of elytra, lateral setae short or long (subequal to metatarsal claw length), black, at apex brown. Ratio of length of metepisternum/metacoxa: 1/1.

**Legs**: Setae on metafemur black, at apex brown, dense. Metatibia evenly widened towards apex; ratio maximal width/maximal length at dorsal margin: 1/1.13. Meso- and metatarsomeres elongate, weakly widened posteriorly, impunctate dorsally, sparsely to densely setose ventrally, circular in cross section, metatarsomere 1 distinctly longer than following tarsomere.

Aedeagus: Figures 8O and 9O. Habitus: Fig. 13E, F.

**Diagnosis**. *Pachypus baroniensis* sp. n. is very similar to *P. gallurensis*. Both holotypes differ in a couple of candidate diagnostic characters, such as the lack of dorsal punctation on metatarsomere 1, the shorter metatibia and the more elongate anterior face of the labrum in *Pachypus baroniensis* sp. n. However, it is unclear how stable these characters really are, as we have observed them to be variable in other species with more sampling.

**Etymology**. The new species is named after the Sardinian region Baronie where the species occurs (adjective in nominative case).

**Variation**. Maximum body length: 14.3–14.4 mm, elytral length: 7.1–7.5 mm, maximum width: 7.1–7.8 mm. Sometimes parts of the pronotum shimmering slightly lighter black reddish.

**Remarks**. Dietz et al^22^. referred to this species as *Pachypus* sp2b.

### *Pachypus occidentalis* sp. n

LSID urn: lsid: zoobank.org: act:26BFA380-4006-441B-B162-DBD25A92559A.

**Type material examined**. *(ID based on mzl-USCOs)*: Holotype: ♂ “X-DA2742 Italy Sardinia: Piscinas (dune), 55 m 39°31’33,4’’N; 08°28’14,9’’E 7.−9.VII.2011” (ZFMK). Paratypes: 1 ♂ “834183 Pachypus candidae Italy, Sardinia (W coast): Camping (Scioppadrosciu) 2 km W of Irgutosu, 62 m, 39°31’31,0’’N, 08°28’20,3’’E, 8.−10.vi.2008, leg. Ahrens & Fabrizi” (ZFMK), 1 ♂ “X-DA2588 Italy Sardegna: Camping Vignola Mare 41°07’37,05’’N, 09°04’23,52’’E 2010 S. Fabrizi & D. Ahrens” (ZFMK), 1 ♂ “X-DA2589 Italy Sardegna: Camping Vignola Mare 41°07’37,05’’N, 09°04’23,52’’E 2010 S. Fabrizi & D. Ahrens” (ZFMK), 1 ♂ “X-DA2594 Italy Sardegna: Camping Vignola Mare 41°07’37,05’’N, 09°04’23,52’’E 2010 S. Fabrizi & D. Ahrens” (ZFMK), 1 ♂ “X-DA2595 Italy Sardegna: Camping Vignola Mare 41°07’37,05’’N, 09°04’23,52’’E 2010 S. Fabrizi & D. Ahrens” (ZFMK), 1 ♂ “X-DA2597 Italy Sardegna: Camping Vignola Mare 41°07’37,05’’N, 09°04’23,52’’E 2010 S. Fabrizi & D. Ahrens” (ZFMK), 1 ♂ “X-DA2598 Italy Sardegna: Camping Vignola Mare 41°07’37,05’’N, 09°04’23,52’’E 2010 S. Fabrizi & D. Ahrens” (ZFMK), 1 ♂ “X-DA2606 Italy Sardegna: Camping Vignola Mare 41°07’37,05’’N, 09°04’23,52’’E 2010 S. Fabrizi & D. Ahrens” (ZFMK), 1 ♂ “X-DA2626 Italy Sardegna: Valledoria 40.93700 N, 8.812998E 2010 S. Fabrizi & D. Ahrens” (ZFMK), 1 ♂ “X-DA2632 Italy Sardegna: Valledoria 40.93700 N, 8.812998E 2010 S. Fabrizi & D. Ahrens” (ZFMK), 1 ♂ “X-DA2636 Italy Sardegna: Valledoria 40.93700 N, 8.812998E 2010 S. Fabrizi & D. Ahrens” (ZFMK), 1 ♂ “X-DA2639 Italy Sardegna: Valledoria 40.93700 N, 8.812998E 2010 S. Fabrizi & D. Ahrens” (ZFMK), 1 ♂ “X-DA2656 Italy Sardinia: Portixeddu, 0-42m 39°26’29,8’’N; 08°25’22,3’’E 9.−11.VII.2011 S. Fabrizi & D. Ahrens” (ZFMK), 1 ♂ “X-DA2656 Italy Sardinia: Portixeddu, 0-42m 39°26’29,8’’N; 08°25’22,3’’E 9.−11.VII.2011 S. Fabrizi & D. Ahrens” (ZFMK), 1 ♂ “X-DA2657 Italy Sardinia: Portixeddu, 0-42m 39°26’29,8’’N; 08°25’22,3’’E 9.−11.VII.2011 S. Fabrizi & D. Ahrens” (ZFMK), 1 ♂ “X-DA2658 Italy Sardinia: Bosa Marina, 16 m 40°16’31,5’’N; 08°29’05,3’’E 2.VII.2011 S. Fabrizi & D. Ahrens” (ZFMK), 1 ♂ “X-DA2659 Italy Sardinia: Bosa Marina, 16 m 40°16’31,5’’N; 08°29’05,3’’E 2.VII.2011 S. Fabrizi & D. Ahrens” (ZFMK), 1 ♂ “X-DA2709 Italy Sardinia: dunes between Torre dei Corsari and Pistis, 40 m 39°41’20,0’’N; 08°27’25,4’’E 5–7.VII.2011 S. Fabrizi & D. Ahrens” (ZFMK), 1 ♂ “X-DA2719 Italy Sardinia: dunes between Torre dei Corsari and Pistis, 40 m 39°41’20,0’’N; 08°27’25,4’’E 5–7.VII.2011 S. Fabrizi & D. Ahrens” (ZFMK), 1 ♂ “X-DA2739 Italy Sardinia: Piscinas (dune), 55 m 39°31’33,4’’N; 08°28’14,9’’E 7.−9.VII.2011” (ZFMK), 1 ♂ “X-DA2740 Italy Sardinia: Piscinas (dune), 55 m 39°31’33,4’’N; 08°28’14,9’’E 7.−9.VII.2011” (ZFMK), 1 ♂ “X-DA2776 Italy Sardinia: Is Arenas, 15 m 28.VI.−4.VII.2011” (ZFMK), 1 ♂ “X-DA2778 Italy Sardinia: Is Arenas, 15 m 28.VI.−4.VII.2011” (ZFMK), 1 ♂ “X-DA2779 Italy Sardinia: Is Arenas, 15 m 28.VI.−4.VII.2011” (ZFMK), 1 ♂ “X-DA2784 Italy Sardinia: dunes between Torre dei Corsari and Pistis, 40 m 39°41’20,0’’N; 08°27’25,4’’E 5–7.VII.2011 S. Fabrizi & D. Ahrens” (ZFMK), 1 ♂ “X-DA2794 Italy Sardinia: dunes between Torre dei Corsari and Pistis, 40 m 39°41’20,0’’N; 08°27’25,4’’E 5–7.VII.2011 S. Fabrizi & D. Ahrens” (ZFMK), 1 ♂ “X-DA2810 Italy Sardinia: Is Arenas, 15 m 28.VI.−4.VII.2011 S. Fabrizi & D. Ahrens” (ZFMK), 1 ♂ “X-DA4239 - Italy, Sardinia: Gutturu Mannu (Assemini), 26.vi.2014, leg. D. Cillo & E. Bazzato” (ZFMK).

Paratypes *(ID based on IUMG*): see Supplement File 1.

**Description of holotype**: Maximum body length: 16.1 mm, elytral length: 7.9 mm, maximum width: 7.3 mm.

**Color**: Head reddish brown, pronotum dark brown with the lateral margins lighter, shiny, elytra reddish brown, their apex slightly darker, moderately shiny, with weak tomentum, humeri strongly shiny; ventral surface reddish brown, shiny. Dorsal surface glabrous except on densely and erectly setose frons; ventral surface with dense and long yellow setae; abdomen and pygidium light brown, pygidium in middle sparsely punctate and setose, otherwise finely densely punctate and with long setae.

**Head**: Clypeus semicircular, surface strongly concave, surface densely finely punctate, glabrous. Antenna completely yellow, antennal club short, almost straight, club length distinctly shorter than remaining antennomeres combined. Eyes small, ratio maximal diameter/interocular width: 0.48. Ocular canthus wide and moderately long, slightly evenly narrowed towards apex, apex rounded, densely punctate and setose; 1/3 of maximal ocular diameter; ocular canthus lower than frons and separated from it by a supraocular carina. Area behind frontoclypeal suture deeply impressed, behind this impression with a long, weakly curved frontal carina, which is evenly raised; frons otherwise coarsely and densely punctate and covered with long, erect, brown setae.

**Elytra **elongate, subtriangular, moderately narrowed apically; surface with fine striae, intervals flat, with very sparse indistinct punctures and glabrous, even intervals distinctly wider than odd ones. Epipleural margin present until apical rounding of elytra, lateral setae mostly long (subequal to metatarsal claw length), yellow. Ratio of length of metepisternum/metacoxa: 1/1.

**Legs**: Setae on metafemur yellow, dense. Metatibia evenly widened towards apex; ratio maximal width/maximal length at dorsal margin: 1/1.19; mesal face entirely densely covered with granulose punctures each bearing a long seta. Meso- and metatarsomeres short and robust, moderately elongate and posteriorly distinctly widened, partly sparsely punctate and with setae dorsally, sparsely to densely setose ventrally, circular in cross section, metatarsomere 1 distinctly longer than following tarsomere.

Aedeagus: Figures 8P and 9P. Habitus: Fig. 13G, H.

**Diagnosis**. *Pachypus occidentalis* sp. n. is very similar to *P. cornutus*. Both species share the densely setose mesal face of the metatibia. *Pachypus occidentalis* sp. n. differs from the latter by the slightly more elongate body. However, this might not apply to all specimens as most external morphological shape traits vary considerably.

**Etymology**. The name of the new species is derived from the Latin word *occidentalis* (west) referring to the westernmost occurrence on the island (adjective in nominative case).

**Variation**. Maximum body length: 11.8–16.1 mm, elytral length: 6.0–7.9 mm, maximum width: 5.4–7.3 mm. The color of elytra may vary from reddish brown (with generally indistinct apical darkening or margin which can extend over half of apical elytra) to dark brown, never completely black; specimens with dark brown elytra without darker apical area also occur. Exceptionally the dark spot is also circumscribed more sharply (in co-occurrence zones with *P. cornutus*; DA2598 from Vignola Mare, Sardinia). Female unknown.

**Remarks**. Eberle et al.^5^and Dietz et al^22^. referred to this species as *Pachypus* sp3.

### *Pachypus pelegrinus* sp. n

LSID urn: lsid: zoobank.org: act:3246FA46-EA2B-4042-AA14-2BCF319AA249.

**Type material examined**. *(ID based on mzl-USCOs)*: Holotype: ♂ “X-DA3355 - Italy, Sardegna: Torre Bari env. (Fiume Manna) Camping Marina, 39°50’08.9’’N, 09°40’49.9’’E, 26.6., 6.7.−9.7.2012, leg. D. Ahrens & S. Fabrizi” (ZFMK). Paratypes: 1 ♂ “836880 Pachypus candidae Italy, Sardinia: S Barusia (5km S of Marina di Tertenia), 39°36’41,3’’N, 9°39’11,1’’E, 9 m, 7.2009, leg. S. Fabrizi” (ZFMK), 1 ♂ “DNA voucher BMNH 837887/Italy: Est-Sardegna: 2 km S Santa Maria Navarese, 15.−18.vii.2001, lg. D. Ahrens” (ZFMK), 1 ♂ “DNA voucher BMNH 837890/Italy: Est-Sardegna: 2 km S Santa Maria Navarese, 15.−18.vii.2001, lg. D. Ahrens” (ZFMK), 1 ♂ “X-DA3354 - Italy, Sardegna: Torre Bari env. (Fiume Manna) Camping Marina, 39°50’08.9’’N, 09°40’49.9’’E, 26.6., 6.7.−9.7.2012, leg. D. Ahrens & S. Fabrizi” (ZFMK), 1 ♂ “X-DA3356 - Italy, Sardegna: Torre Bari env. (Fiume Manna) Camping Marina, 39°50’08.9’’N, 09°40’49.9’’E, 26.6., 6.7.−9.7.2012, leg. D. Ahrens & S. Fabrizi” (ZFMK), 1 ♂ “X-DA3373 - Italy, Sardegna: Lido delle Rose (2 km S. Santa Maria Navarese), 39°58’09.1’’N, 09°41’08.3’’E, 22.−26.6.2012, leg. D. Ahrens & S. Fabrizi” (ZFMK), 1 ♂ “X-DA3374 - Italy, Sardegna: Lido delle Rose (2 km S. Santa Maria Navarese), 39°58’09.1’’N, 09°41’08.3’’E, 22.−26.6.2012, leg. D. Ahrens & S. Fabrizi” (ZFMK), 1 ♂ “X-DA3375 - Italy, Sardegna: Lido delle Rose (2 km S. Santa Maria Navarese), 39°58’09.1’’N, 09°41’08.3’’E, 22.−26.6.2012, leg. D. Ahrens & S. Fabrizi” (ZFMK), 1 ♂ “X-DA3415 - Italy, Sardegna: Torre Chia (Camping), 38°53’57.5’’N, 08°53’10.0’’E, 28.6.−1.7.2012, leg. D. Ahrens & S. Fabrizi” (ZFMK), 1 ♂ “X-DA3416 - Italy, Sardegna: Torre Chia (Camping), 38°53’57.5’’N, 08°53’10.0’’E, 28.6.−1.7.2012, leg. D. Ahrens & S. Fabrizi” (ZFMK), 1 ♂ “X-DA3419 - Italy, Sardegna: Torre Chia (Camping), 38°53’57.5’’N, 08°53’10.0’’E, 28.6.−1.7.2012, leg. D. Ahrens & S. Fabrizi” (ZFMK), 1 ♂ “X-DA3422 - Italy, Sardegna: Torre Chia (Camping), 38°53’57.5’’N, 08°53’10.0’’E, 28.6.−1.7.2012, leg. D. Ahrens & S. Fabrizi” (ZFMK), 1 ♂ “X-DA4229 Italy, Sardinia: Sinnai (CA); S. Gregorio, vii.2013, D. Sechi” (ZFMK), 1 ♂ “X-DA4230 Italy, Sardinia: Sinnai (CA); S. Gregorio, 19.VI.2014, D. Cillo & E. Bazzato” (ZFMK), 1 ♂ “X-DA4231 Italy, Sardinia: Sinnai (CA); S. Gregorio, 19.VI.2014, D. Cillo & E. Bazzato” (ZFMK), 1 ♂ “X-DA4238 - Italy, Sardinia: Gutturu Mannu (Assemini), 26.vi.2014, leg. D. Cillo & E. Bazzato” (ZFMK), 1 ♂ “X-DA4272 - Italy, Sardegna: S. Margherita di Pula, 15.VI.2013, leg. Alamanni” (ZFMK).

Paratypes *(ID based on IUMG*): see Supplement File 1.

**Description of holotype**: Maximum body length: 17.0 mm, elytral length: 8.8 mm, maximum width: 8.5 mm.

**Color**: Head and pronotum dark reddish brown, pronotal disc slightly darker, shiny, elytra reddish brown, their apex with a large, elongate and almost distinctly circumscribed dark spot, dull, with tomentum, humeri shiny; ventral surface reddish brown, shiny. Dorsal surface glabrous except on densely and erectly setose frons; ventral surface with dense and long yellow setae; abdomen and pygidium light brown, pygidium finely densely punctate and with long setae.

**Head**: Clypeus semicircular, surface strongly concave, surface densely finely punctate, glabrous. Antenna completely yellow, antennal club moderately short, almost straight, club length as long as remaining antennomeres combined. Eyes small, ratio maximal diameter/interocular width: 0.5. Ocular canthus moderately long, slightly evenly narrowed towards apex, apex rounded, densely punctate and setose; 1/3 of maximal ocular diameter; ocular canthus lower than frons and separated from it by a supraocular carina. Area behind frontoclypeal suture deeply impressed, behind this impression with a long, weakly curved frontal carina, which is sharp and distinctly evenly raised; frons otherwise coarsely and densely punctate and covered with long, erect, brown setae of which some anterior ones are partly abraded and present only as short stubs of setae.

**Elytra** elongate, subtriangular, strongly narrowed apically; surface with fine striae, intervals flat, with sparse, indistinct punctures and glabrous, even intervals distinctly wider than odd ones. Epipleural margin present until apical rounding of elytra, lateral setae very long (longer than metatarsal claw length), yellow. Ratio of length of metepisternum/metacoxa: 1/1.

**Legs**: Setae on metafemur yellow, dense. Metatibia evenly widened towards apex; ratio maximal width/maximal length at dorsal margin: 1/1.2; dorsoapical half of mesal face smooth and impunctate, otherwise densely covered with robust, partly granulose punctures each bearing a long seta. Meso- and metatarsomeres elongate but posteriorly distinctly widened, impunctate and glabrous dorsally, sparsely to densely setose ventrally, circular in cross section, metatarsomere 1 distinctly longer than following tarsomere.

Aedeagus: Figures 8N and 9N. Habitus: Fig. 13C, D.

**Diagnosis**. *Pachypus pelegrinus* sp. n. is similar to *P. cornutus*. Both types share the reddish elytra with the dark apical spot. However, *P. cornutus* exists also in an entirely black form, which is so far not known for *P. pelegrinus* sp. n. *Pachypus pelegrinus* sp. n. differs additionally from *P. cornutus* by the slightly longer antennal club, the uneven distribution of the punctures on the mesal face of the metatibia, as well as the more elongate metatarsomeres.

**Etymology**. The name of the new species is derived from the Latin word *pelegrinus* (wanderer) referring to the widely dispersed occurrence of the species in Sardinia (adjective in nominative case).

**Variation**. Maximum body length: 9.7–17.0 mm, elytral length: 5.1–8.8 mm, maximum width: 4.6–8.5 mm. Some specimens are darker brown, especially the elytra, in one the dark brown elytra have a yellow lateral margin, others are entirely black, however, they all have yellow antennae. However, the ventral pilosity in black specimens is dark brown. In specimens with red-brown elytra, the apical dark spot is always well defined. Female unknown.

**Remarks**. Dietz et al^22^. referred to this species as *Pachypus* sp4.

### *Pachypus cornutus* (Olivier, 1789)

*Melolontha cornuta* Olivier, 1789: no. 5: 20^17^.

*Pachypus cornutus*: Erichson 1840: 34^18^.

**Type material examined. ***(ID based on mzl-USCOs)*: Neotype (here designated): ♂ “X-DA4259 - France, Estuary env. of river Liamone, 4 km S Sagone (daytime), 42°05.143’N, 009°04.125’E [sic!], 29.vi.−3.vii.2014, leg. Ahrens & Fabrizi leg.” (ZFMK).

**Additional material examined**. *(ID based on mzl-USCOs)*: 1 ♂ “X-DA2586 Italy Sardegna: Camping Vignola Mare 41°07’37,05’’N, 09°04’23,52’’E 2010 S. Fabrizi & D. Ahrens” (ZFMK), 1 ♂ “X-DA4240 - Italy, Sardinia: Palau, Porto Pollo, 4.VII.2014” (ZFMK), 1 ♂ “X-DA4241 - Italy, Sardinia: Palau, Porto Pollo, 4.VII.2014” (ZFMK), 1 ♂ “X-DA4242 - Italy, Sardinia: Palau, Porto Pollo, 4.VII.2014” (ZFMK), 1 ♂ “X-DA4248 - France, Corsica: L’Ostriconi (daytime), 42°39.264’N, 009°04.125’E, 5.−11.vii.2014, leg. Ahrens & Fabrizi leg.” (ZFMK), 1 ♂ “X-DA4249 - France, Corsica: L’Ostriconi (daytime), 42°39.264’N, 009°04.125’E, 5.−11.vii.2014, leg. Ahrens & Fabrizi leg.” (ZFMK), 1 ♂ “X-DA4250 - France, Camping “U Sole Marinu”, Farinole env., river Albine (daytime), 42°42.845’N, 009°20.012’E, 7.vii.2014, leg. Ahrens & Fabrizi leg.” (ZFMK), 1 ♂ “X-DA4251 - France, Camping “U Sole Marinu”, Farinole env., river Albine (daytime), 42°42.845’N, 009°20.012’E, 7.vii.2014, leg. Ahrens & Fabrizi leg.” (ZFMK), 1 ♂ “X-DA4252 - France, Casteddu d’Araghju (vistor parking place of the castle); 5 km NW Porto Vecchio (daytime), 41°38.515’N, 009°15.973’E, 24.−26.vi.2014, leg. Ahrens & Fabrizi leg.” (ZFMK), 1 ♂ “X-DA4253 - France, Casteddu d’Araghju (vistor parking place of the castle); 5 km NW Porto Vecchio (daytime), 41°38.515’N, 009°15.973’E, 24.−26.vi.2014, leg. Ahrens & Fabrizi leg.” (ZFMK), 1 ♂ “X-DA4254 - France, Casteddu d’Araghju (vistor parking place of the castle); 5 km NW Porto Vecchio (daytime), 41°38.515’N, 009°15.973’E, 24.−26.vi.2014, leg. Ahrens & Fabrizi leg.” (ZFMK), 1 ♂ “X-DA4255 - France, Casteddu d’Araghju (vistor parking place of the castle); 5 km NW Porto Vecchio (daytime), 41°38.515’N, 009°15.973’E, 24.−26.vi.2014, leg. Ahrens & Fabrizi leg.” (ZFMK), 1 ♂ “X-DA4256 - France, Casteddu d’Araghju (vistor parking place of the castle); 5 km NW Porto Vecchio (daytime), 41°38.515’N, 009°15.973’E, 24.−26.vi.2014, leg. Ahrens & Fabrizi leg.” (ZFMK), 1 ♂ “X-DA4257 - France, Estuary env. of river Liamone, 4 km S Sagone (daytime), 42°05.143’N, 009°04.125’E [sic!], 29.vi.−3.vii.2014, leg. Ahrens & Fabrizi leg.” (ZFMK), 1 ♂ “X-DA4258 - France, Estuary env. of river Liamone, 4 km S Sagone (daytime), 42°05.143’N, 009°04.125’E [sic!], 29.vi.−3.vii.2014, leg. Ahrens & Fabrizi leg.” (ZFMK), 1 ♂ “X-DA4260 - France, Estuary env. of river Liamone, 4 km S Sagone (daytime), 42°05.143’N, 009°04.125’E [sic!], 29.vi.−3.vii.2014, leg. Ahrens & Fabrizi leg.” (ZFMK), 1 ♂ “X-DA4261 - France, Estuary env. of river Liamone, 4 km S Sagone (daytime), 42°05.143’N, 009°04.125’E [sic!], 29.vi.−3.vii.2014, leg. Ahrens & Fabrizi leg.” (ZFMK), 1 ♂ “X-DA4262 - France, Estuary env. of river Liamone, 4 km S Sagone (daytime), 42°05.143’N, 009°04.125’E [sic!], 29.vi.−3.vii.2014, leg. Ahrens & Fabrizi leg.” (ZFMK), 1 ♂ “X-DA4263 - France, Camping Villata (10 km N Porto Vecchio) (daytime), 41°39.565’N, 009°22.366’E, 21.−27.vi.2014, leg. Ahrens & Fabrizi leg.” (ZFMK), 1 ♂ “X-DA4264 - France, Camping Villata (10 km N Porto Vecchio) (daytime), 41°39.565’N, 009°22.366’E, 21.−27.vi.2014, leg. Ahrens & Fabrizi leg.” (ZFMK), 1 ♂ “X-DA4265 - France, Camping Villata (10 km N Porto Vecchio) (daytime), 41°39.565’N, 009°22.366’E, 21.−27.vi.2014, leg. Ahrens & Fabrizi leg.” (ZFMK), 1 ♂ “X-DA4266 - France, Camping Villata (10 km N Porto Vecchio) (daytime), 41°39.565’N, 009°22.366’E, 21.−27.vi.2014, leg. Ahrens & Fabrizi leg.” (ZFMK), 1 ♂ “X-DA4267 - France, Camping Villata (10 km N Porto Vecchio) (daytime), 41°39.565’N, 009°22.366’E, 21.−27.vi.2014, leg. Ahrens & Fabrizi leg.” (ZFMK).

**Additional material examined**. *(ID based on IUMG*): see Supplement File 1.

**Redescription of neotype**: Maximum body length: 11.6 mm, elytral length: 6.1 mm, maximum width: 6.4 mm.

**Color**: Head and pronotum dark brown, some parts of the latter lighter, shiny, elytra reddish brown, their apex with a large, elongate but indistinctly circumscribed dark spot, dull, with tomentum, humeri shiny; ventral surface reddish brown, shiny. Dorsal surface glabrous except on densely and erectly setose frons; ventral surface with dense and long yellow setae; abdomen and pygidium light brown, pygidium finely densely punctate and with long setae.

**Head**: Clypeus semicircular, surface strongly concave, surface densely finely punctate, glabrous. Antenna completely yellow, antennal club short, almost straight, club length a little shorter than remaining antennomeres combined. Eyes small, ratio maximal diameter/interocular width: 0.56. Ocular canthus short, slightly evenly narrowed towards apex, apex rounded, densely punctate and setose; 1/4 of maximal ocular diameter; ocular canthus lower than frons and separated from it by a supraocular carina. Area behind frontoclypeal suture deeply impressed, behind this impression with a long, weakly curved frontal carina, which is weakly evenly raised; frons otherwise coarsely and densely punctate and covered with long erect yellow setae which are all partly abraded and present only by short trunks of setae.

**Elytra** elongate, subtriangular, strongly narrowed apically; surface with fine striae, intervals flat, with sparse, indistinct punctures and glabrous, even intervals distinctly wider than odd ones. Epipleural margin present until apical rounding of elytra, lateral setae very long (longer than metatarsal claw length), yellow. Ratio of length of metepisternum/metacoxa: 1/1.05.

**Legs**: Setae on metafemur yellow, dense. Metatibia evenly widened towards apex; ratio maximal width/maximal length at dorsal margin: 1/1.27; mesal face entirely densely covered with granulose punctures each bearing a long seta. Meso- and metatarsomeres short and robust, less elongate and posteriorly distinctly widened, partly sparsely punctate and with setae dorsally, sparsely to densely setose ventrally, circular in cross section, metatarsomere 1 distinctly longer than following tarsomere.

Aedeagus: Figures 8J and 9J. Habitus: Fig. 12C, D.

**Diagnosis**. *Pachypus cornutus* is similar to *P. excavatus*. Both share a color polymorphism in the elytra being either brown or entirely black. However, *P. cornutus* differs from *P. excavatus* by always present dark apical, more or less extended and distinct spot on the apex of the elytra. Furthermore, the metatarsomeres are more robust and distinctly more widened apically and the punctures on the mesal face of metatibia are more evenly dense and granular, compared to *P. excavatus*.

**Variation**. The color of elytra may vary from reddish brown (with well-defined dark apical spot or margin) to black, pronotum dark brown to black, antenna always yellow even in black specimens.

**Remarks**. Eberle et al.^5^ referred to this species as *Pachypus *sp1 and sp5, but Dietz et al^22^. recognized it as one entity (sp5). A neotype of *Pachypus cornutus* Olivier, 1789 had to be designated here, as the species was originally described from Corsica and Calabria^17^, where two different species are known to occur. The designation of a neotype was necessary as no original type material could be retrieved in any collection after extensive search, and the status of the species had to be fixed to be unambiguous, since based on the images of the original description the species cannot be distinguished as they strongly vary in color. The originally depicted specimen (plate N. IX, subfigure 74a)^17^ shows brown elytra with a dark apex as well as an almost entirely dark pronotum, which is consistent with both the species occurring in Calabria (*P. candidae*) and in Corsica (this species).

The GPS coordinates on some specimens’ labels including the neotype are incorrect: 42°05.143’N, 009°04.125’E [sic!] – the corrected coordinates of the collection locality are: 42.087°N, 8.734°E.

### *Pachypus franginii* sp. n

LSID urn: lsid: zoobank.org: act:22EC51AD-64FA-4C4C-A991-0850BE71792E.

**Type material examined**. *(ID based on mzl-USCOs)*: Holotype: ♂ “X-DA4080 - Italy, Elba, Portoferraio, loc. Norsi, 03.07.2013, leg. Forbicioni” (ZFMK). Paratypes: 1 ♂ “X-DA4081 - Italy, Elba, Portoferraio, loc. Norsi, 03.07.2013, leg. Forbicioni” (ZFMK), 1 ♂ “X-DA4077/Italia Toscana Isola d’Elba, Norsi 42.7700, 10.3461 10.VII.2013 lg. L. Forbicioni//MU.04//Coll. M. Uliana”/PP0352” (ZFMK), 1 ♂ “X-DA4075/Italia Toscana Isola d’Elba, Norsi 42.7700, 10.3461 10.VII.2013 lg. L. Forbicioni//MU.02//Coll. M. Uliana”/PP0353” (ZFMK), 1 ♂ “X-DA4078/Italia Toscana Isola d’Elba, Norsi 42.7700, 10.3461 10.VII.2013 lg. L. Forbicioni//MU.05//Coll. M. Uliana/PP0359” (ZFMK), 1 ♂ “X-DA4076/Italia Toscana Isola d’Elba, Norsi 42.7700, 10.3461 4.VII.2013 lg. L. Forbicioni//MU.03//Coll. M. Uliana/PP0362” (ZFMK), 1 ♂ “X-DA4079/Italia Toscana Isola d’Elba, Norsi 42.7700, 10.3461 10.VII.2013 lg. L. Forbicioni//MU.06//Coll. M. Uliana”/PP0368” (ZFMK).

Paratypes *(ID based on IUMG*): see Supplement File 1.

**Description of holotype**: Maximum body length: 13.0 mm, elytral length: 6.9 mm, maximum width: 6.6 mm.

**Color**: Head and pronotum black, shiny, elytra black, with dull tomentum, humeri weakly shiny; ventral surface black, shiny. Dorsal surface glabrous except on densely and erectly setose frons; ventral surface with dense and long, brown and partly yellowish setae; abdomen and pygidium black, sparsely punctate and setose.

**Head**: Clypeus semicircular, surface strongly concave, surface densely finely punctate, glabrous. Antenna completely black, antennal club long, straight, club length distinctly longer than remaining antennomeres combined. Eyes small, ratio maximal diameter/interocular width: 0.5. Ocular canthus wide and moderately long, slightly evenly narrowed towards apex, apex rounded, densely punctate and setose; 1/3 of maximal ocular diameter; ocular canthus lower than frons and separated from it by a supraocular carina. Area behind frontoclypeal suture deeply impressed, behind this impression with a long, weakly curved frontal carina, which is weakly raised and medially narrowly interrupted; frons otherwise coarsely and densely punctate and covered with long, erect, dark brown setae.

**Elytra** elongate, subtriangular, strongly narrowed apically; surface with fine striae, intervals flat, with very sparse indistinct punctures and glabrous, even intervals distinctly wider than odd ones. Epipleural margin present until apical rounding of elytra, lateral setae apically shorter (shorter than metatarsal claw length), black. Ratio of length of metepisternum/metacoxa: 1/0.9.

**Legs**: Setae on metafemur black but partly also yellowish brown, dense. Metatibia evenly widened towards apex; ratio maximal width/maximal length at dorsal margin: 1/1.4. Meso- and metatarsomeres elongate, weakly widened posteriorly, metatarsomere 1 sparsely punctate dorsally, sparsely to densely setose ventrally, circular in cross section, metatarsomere 1 distinctly longer than following tarsomere.

Aedeagus: Figures 8B and 9B. Habitus: Fig. 11C, D.

**Diagnosis**. *Pachypus franginii* sp. n. is very similar to *P. sulcis* sp. n. It differs from the latter only by the slightly more robust metatibia, the punctate metatarsomere 1, the less reflexed antennal club of the male, and the darker ventral pilosity. From *P. excavatus*, *Pachypus franginii* sp. n. differs by the black antennal club, the unevenly punctate mesal face of metatibia, and the punctate metatarsomere 1.

Variation. Maximum body length: 13.0–15.1 mm, elytral length: 6.9–7.9 mm, maximum width: 6.6–7.8 mm. Body uniformly black, sometimes elytra partly dark brown, in a few cases elytra brown or reddish brown with a dark, well-defined apical spot (similar to that in *P. cornutus*). Female unknown.

**Etymology**. The new species is named in memory of Giuliano Frangini, the naturalist.

who discovered the population at the type locality and provided the first specimens available to us (noun in genitive singular).

**Remarks**. Eberle et al.^5^ and Dietz et al.^22^ referred to this species as *Pachypus* sp6. It is likely that an earlier record of *P. candidae* from Elba^43^ refers to this species.

### Key to species – male sex

(* - species with strong integumental color polymorphism; i.e., dorsal surface/elytra can be brown or black)

1 Mesal face of metatibia evenly and densely punctate, with long setae in the punctures.............2

- Mesal face of metatibia partly impunctate and smooth, without long setae.............. 3

2 Antennal club long, distinctly longer than remaining antennomeres combined. Elytra with color polymorphism, reddish brown with a dark apical spot or entirely black. Frontal carina distinct. Metatarsi more elongate............................................................................. P. cornutus*

- Antennal club short, distinctly shorter than remaining antennomeres combined. Elytra without color polymorphism, reddish brown to dark brown without a distinctly dark apical spot, never entirely black. Frontal carina indistinct. Metatarsi more robust. ***P. occidentalis* sp. n.**

3 Species from northern Africa (i.e., northern Tunisia, Algeria). ......... P. demoflysi*

- Species from Sicily. ........................................................................................ ***P. caesus***

- Species from Elba............................................................................ *P. franginii *sp. n.*

- Species from Apennine peninsula. .............................................................................4

- Species from Sardinia. ................................................................................................... 5

4 Metatibia narrower, ratio maximal width/ maximal length at dorsal margin: 1/1.6. Species from central Tyrrhenian Italy. .......................................................................... P. excavatus*

- Metatibia wider, ratio maximal width/ maximal length at dorsal margin: 1/1.2. Species from southern Apennine (Calabria and Basilicata)........................................ ***P. candidae***

5 Body almost entirely uniformly black or dark brown. ................................................. 6

- Elytra with color polymorphism, reddish brown with a dark apical stain or margin or completely dark brown to black. Antennal club always yellow. ................. ***P. pelegrinus *sp. n.**

6 Pilosity of ventral surface blackish. .............................................................................. 7

- Pilosity of ventral surface pale, white or yellow. ........................................................ 8

7 Metatibia shorter, without punctures on metatarsi. ............... ***P. baroniensis *sp. n.**

- Metatibia slightly longer, with a few punctures on metatarsi. ***P. gallurensis *sp. n.**

8 Parameres weakly curved in apical quarter (lateral view)............... ***P. sardiniensis***

- Parameres more strongly curved in apical quarter (lateral view)................................

................................***P. impressus, P. sulcis *sp. n.,* P. matzaccara *sp. n.**

## Discussion

Based on the thorough integrative taxonomic analysis by Dietz et al.^22^(Fig. 2), we provided the formal taxonomic diagnosis of the previously delineated species resulting from an almost two-decades-long research project on *Pachypus* beetles. While the peculiar biology of *Pachypus* had attracted attention of previous workers to assess their taxonomy^14,15^ and ecology^11,12,44,45^, its taxonomy has been poorly understood.

After first attempts to use molecular traits^5^ in order to shed light on the taxonomy of this genus, our results (in combination with those of Dietz et al.^22^) helped to understand why it has been so difficult to resolve the puzzling taxonomy of the species of *Pachypus*.

### Species delimitation, museomics and taxonomy

The morphometric analysis of four outline traits and of various body measurements revealed extensive overlap between the species (Figs. 5 and 6). While early attempts of using morphometrics^11,39^ looked promising to resolve the species taxonomy, we were able to show that an increased sampling blurs apparent distinctiveness^5,22^. The revealed introgression between different species, especially in the clade around *P. excavatus* (clade B1^22^), might have contributed to that, although it has so far been considered to occur only rarely^22^.

The limited dispersal capacity of females and the resulting poor connectivity between the populations of each species also led to a considerable differentiation between many populations^5^, which was detected also by our morphometric analyses (Fig. 7). Additionally, we observed remarkable body size variation, at least in males, in which large specimens of some species had almost twice the size of smaller individuals.

The color polymorphism, which mainly affects the elytra, has not been found in all species of the genus. In the past, many formally named *Pachypus* forms had been established based on color only^34^. Some of them are, however, nomenclatorially not valid (see above). In females, we were unable to observe such a polymorphism, as females are almost always reddish brown, showing little expression of particular body pigments in adaptation to their prevailingly subterranean lifestyle.

In this study, we successfully sequenced the almost 200-year-old lectotype of *Pachypus impressus* and used it to establish the identity of the three cryptic species lineages formerly classified as *Pachypus melonii*^22^. The latter species turned out to be a synonym of *P. impressus*. The ML trees based on both ends of COI (Fig. 4) showed the lectotype of *P. impressus* to have almost identical nucleotide sequences as specimen DA3973, both being nested in a clade with specimens exclusively originating from the type locality of *P. melonii*. Although the COI data of the lectotype suggested its geographic origin being the same as that of the holotype of *P. melonii*, the incomplete mzl-USCO data of the lectotype resulted in considerable branch lengths for the specimen in both USCO trees (Figs S1, 2), which can lead to additional but likely spurious splitting in species delimitation analysis. Despite the benefit of type genomics^46^ and the potential progress that comes with it, our results made clear that the evaluation of such museomics data requires a high-quality data backbone (in terms of data completeness), in which data from type specimens can be adequately placed and interpreted. Without this, these data may not be usable and can possibly be misleading. Given the limited numbers of attempts possible with the small amount of available DNA, sequencing strategies might be oriented towards already available data sets considering potential issues of data compatibility. Dietz et al.^47^ have shown that target enrichment approaches might come with limitations in this regard since available data are determined by the a priori bait design. This is even more valid for RAD sequencing^24^. In our case, the additional availability of complete COI data of the sequenced type specimen has been highly useful and informative. Our data has perfectly shown that COI sequences, despite their limitations for species delimitation, were highly indicative for the identity of the populations and metapopulations in *Pachypus*^5,22^. However, the extraction of COI data from target enrichment data has not always been successful, as we obtained sufficiently complete COI data for only about half of the specimens (Fig. 4; Supplementary file 7).

**Fig. 14:**
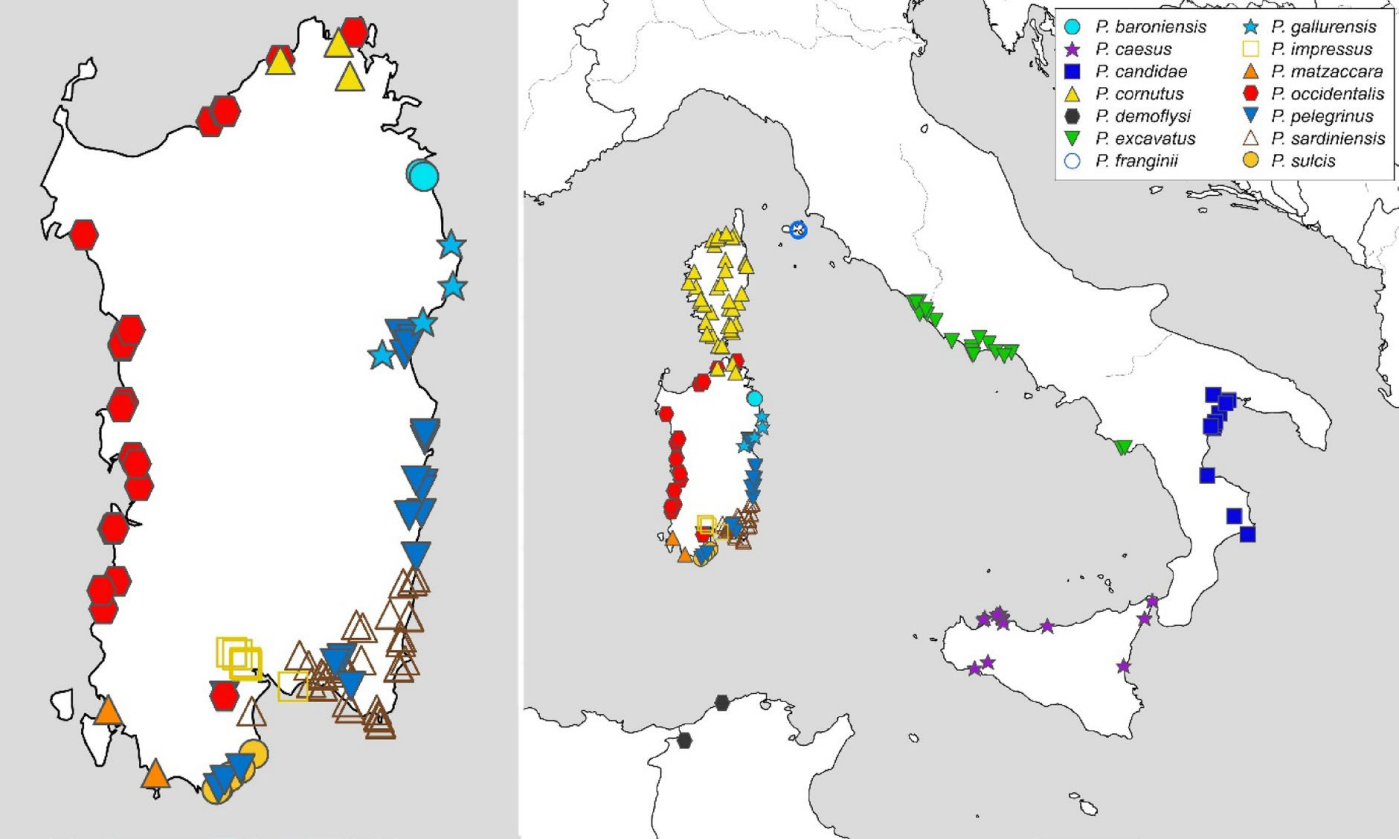
Currently known distribution of the species of *Pachypus* as inferred by the specimens examined here and identified with certainty. Populations with yet uncertain species assignment are not shown.

**Fig. 15:**
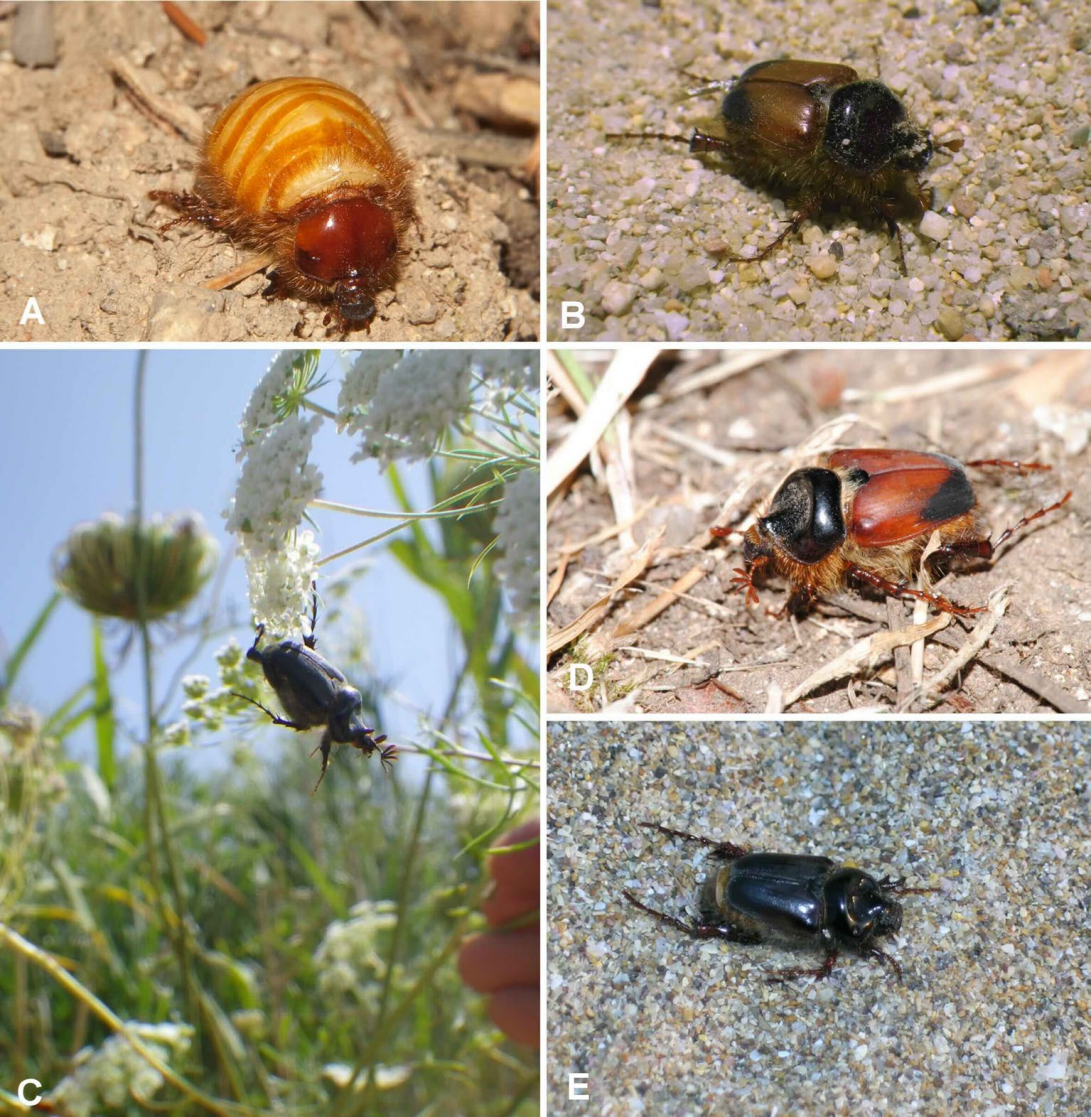
Habitus images of alive female (**A**) and male (**B**-**E**) specimens of *Pachypus pelegrinus* (**A**,** B**), *P. impressus* (**C**), *P. cornutus* (**D**), *P. occidentalis* (**E**).

**Fig. 16:**
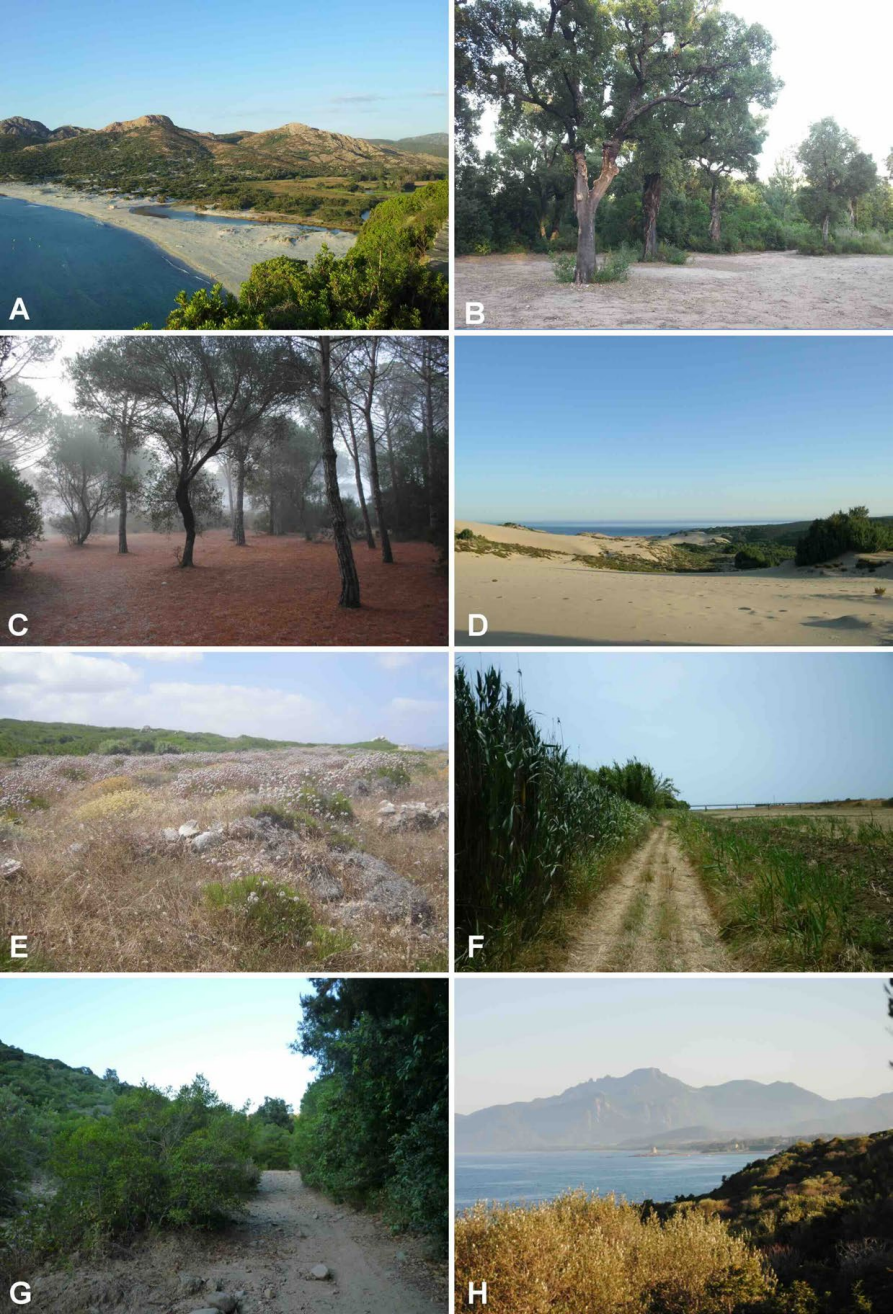
Photographs of the habitat of *Pachypus cornutus* (A – coastal habitats close to L’Ostriconi, Corsica, B – Casteddu d’Araghju, Corsica; E – Vignola Mare, Sardinia, here co-occurrence with *P. occidentalis*), *P. gallurensis* (C – pine tree forst at Cala Ginepro, Sardinia), *P. occidentalis* (D – dunes of Piscinas, Sardinia), *P. impressus* (F – river plains close to Assemini, type locality of *P. melonii* and *P. impressus*), *P. pelegrinus (*G – Sinnai, San Gregorio, Sardinia, H - Torre Bari, Sardinia).

For the interpretation of the integrative taxonomy analysis^22^ it was highly informative that several species of *Pachypus* co-occur syntopically, such as *P. sulcis* and *P. pelegrinus*, *P. sardiniensis* and *P. pelegrinus*, *P. cornutus* and *P. occidentalis* (Fig. 14). With the exception of one case, in which the closely related *P. occidentalis* and *P. pelegrinus* co-occurred, species boundaries were preserved and no increased introgression occurred – proof of concept for the hypothesized species entities. In some of these cases, we observed some morphological features to become intermediate, which otherwise in unmixed populations were quite distinctive, such as coloration, the length of the antennal club, or the thickness of metatarsomeres, which made the identification in such locations often very difficult (Figs 15, 16). Interestingly, the genomic data did not show many instances of introgression^22^, but the number of analyzed specimens was also fairly low.

Regarding the resolved taxonomy, it has become evident how powerful the use of physical name bearing specimens is: species hypotheses continue to remain testable with the exploration of additional evidence. All this would not have been possible with illustrations or images used as type specimens^48^. Fixing a lectotype of *P. impressus* and analyzing its DNA was crucial for resolving the identity of this complex of cryptic species, which have been revealed to be phylogenetically old^22^. In the same way it was necessary to fix a neotype for *Pachypus cornutus*, most of all to clarify its ambiguous locus typicus (see above), given that no original specimens of the type series were retraceable. The case of *Pachypus* highlights once more the importance of physical specimen typification including neotypes.

### Species natural history, ecology, and distribution

The intensive study of the evolution of the species of *Pachypus*^22^ allowed us to draw several conclusions about the biology of the species: despite active dispersal by males, female philopatry determines the patterns of infraspecific genetic variation. Dietz et al.^22^ showed that even flightless female specimens of *Pachypus* had been able to disperse from Sardinia to Africa and/or Sicily during sea regressions in the Messinian Salinity Crisis, and also from Corsica to Sardinia in the Pleistocene. However, considerable relief energy, exemplified by steep mountains, and the lack of deeper soil strata in lowlands may represent significant dispersal barriers for females. This could explain why eastern and southern Sardinia harbor more species than the western part of the island. Also, the strong isolation of some presumably rather small populations must have had an accelerating impact on their genetic differentiation and ultimately speciation.

Especially in inland areas we likely do not yet have a complete picture of the distribution of the species. While Crovetti^11^ indicated several deep inland occurrences of *Pachypus*, particularly for Corsica and Sardinia, we found that collecting in the interior of the islands was much less successful, which is why most of our sampling for the molecular study was from larger coastal populations^5,22^. In this context, the specimens retrieved from BOLD database, which have been identified here as *P. cornutus* (Fig. 4), were very useful. The records of the latter were extremely interesting and also important for the understanding of the ecology of *Pachypus* species, because all their collection sites in central-eastern Corsica lie above 900 m elevation. Moreover, their COI data are well differentiated from so far explored populations, which might indicate either autochthonous populations at this elevation or genetically well-differentiated eastern populations (so far, we have studied no USCO data or 5’ COI barcodes from lowlands along the eastern coast of Corsica). Arnone and Sparacio^12^ have reported *P. caesus* from up to 800 m above sea level.

The interesting but well-known patterns of phenology^11^ have been confirmed here: all species have an emergence in summer from June to August, except for *P. caesus* which occurs in autumn from September to November. In contrast to that, the patterns of daily activity have never been properly addressed or investigated. Only Arnone and Sparacio^12^ indicated between the lines that most *Pachypus caesus* beetles (occurring in Sicily only) were captured during the day, and only some from dusk to dawn, including the whole night, being sometimes attracted by light sources. In fact, during our extensive periods of fieldwork we have observed that especially during very hot periods in Sardinia specimens of the same species tended to fly either at dawn and the entire night until the early morning (during hot days), while on colder days they were found only during the day. On the northern Apennine mainland (Lazio) and the northern Islands (e.g. Corsica, Elba), the species were found only during daytime. These obviously flexible activity patterns are even more striking as most related pleurostict scarabs are known to have very well established and evolutionarily fixed daily activity pattern window at species or lineage level, either being exclusively night active or day active^49–51^.

### Habitats and conservation

Most collection sites of *Pachypus* species are situated near areas of Quaternary sedimentation, often in vicinity to larger dunes and rivers. Since males fly quite well, we do not know to what extent collection sites reflect their true habitat, and especially in which habitats they reproduce. Here they are supposed to be most vulnerable. In contrast to males, females are found much more rarely (see also^11^ and are much more time-consuming to find. As both sexes do not feed at the adult stage, adult specimens cannot be collected at food resources, which are often aggregation sites for conspecific insects.

For almost all species and all areas we noted that the species of *Pachypus* can cope quite well with human impact, as long as the soil remains intact and no pesticides are used. Camping places close to a beach have been places where we collected *Pachypus* species in vast abundances, but we found *Pachypus* specimens also in residential areas, and *P. caesus* is present in city parks of Palermo^12^. However, our studies revealed some species to have quite small ranges, and also that genetic variation is quite high among the various populations, which also means that conservation units might be different from species boundaries in this case, possibly at metapopulation level. However, more knowledge on population size and population connectivity is needed for profound and consistent conclusions in this respect.

### Systematic placement of the genus

So far, *Pachypus* is placed as the only genus within the tribe Pachypodiini Erichson, 1840. The original vernacular name (available according Art. 11.7.2^32^) was first used in latinized form by Erichson^52^(as Pachypoda) and has since been currently generally accepted as Pachypodini^20,53^. Some authors, however, referred to it as separate subfamily^9,11^, or even as own family^13–15^. Pachypodini has been placed within Melolonthinae in recent classification schemes^20,29,53^. Melolonthinae, as currently defined^29,30,53^, was revealed to be para- or polyphyletic in several recent molecular phylogenies^6–8,49,54–56^. In these previous analyses the position of *Pachypus* was not stable and always rather isolated. Dietz et al.^8^ revealed *Pachypus* to be, within pleurostict Scarabaeidae, the sister lineage of a clade comprising Dynastinae and Rutelinae, Cetoniinae, Melolonthinae (sensu stricto) as well as Hopliini. While the phylogenetic placement of many scarab lineages is still unknown or uncertain^7,56^, some of these have in the meantime been placed in separate subfamilies (e.g., Aclopinae, Phaenomeridinae, Lichniinae, Oncerinae, Podolasiinae^30^, yet often without a sound phylogenetic basis. Given the phylogenetically very isolated position of *Pachypus*, it is rather certain that it is not part of Melolonthinae (sensu stricto, i.e. the monophyletic lineage around Melolonthini), and thus has to be considered a separate subfamily (Pachypodinae Erichson, 1840) as well.

## Materials and methods

### Morphological examination of specimens

The morphological terminology and examination methods used for measurements, specimen dissection, and genital preparation follow Ahrens^57^. Measurements always refer to the maximum extension of each trait (e.g., maximum body length includes the pygidium if it extends beyond the elytral apex). In contrast to the morphometric measurements (Fig. 1), elytral length in the descriptions was measured from the visible base of the scutellum to the elytral apex, following our established standards^57^. Label data of the specimens examined are cited verbatim in quotation marks, multiple labels are separated by a “/”. Male genitalia were dissected from all specimens and glued to a small, pointed card attached to the specimen. For newly described and redescribed species, male genitalia were photographed in lateral and dorsal views using a stereomicroscope Leica M125 with a Leica DC420C digital camera. The images were combined with Automontage software as implemented in the Leica Application Suite v 4.5 to obtain an entirely focused image. Habitus images of type specimens were taken in dorsal and lateral views with a VHX Keyence Digital Microscope using the ‘3D Image Stitching’ approach. Figure plates were built and digitally edited with Artweaver 5.0.

Abbreviations used in the text for collection depositories are as follows:

CMUC collection Marco Uliana, Codevigo, Italy;

HNHM Hungarian Natural History Museum, Budapest, Hungary;

MNHN Museum National d’Histoire Naturelle, Paris, France;

MSNV Museo di Storia Naturale Giancarlo Ligabue, Venezia, Italy;

ZFMK Museum Koenig Bonn, Leibniz Institute of Biodiversity Change;

ZMHB Museum für Naturkunde, Berlin, Germany;

ZMUK Zoologisches Museum, Universität Kiel, Germany.

### Genome sequencing of historical museum specimen

Mzl-USCO data of the lectotype of *Pachypus impressus* were generated using low-coverage whole genome sequencing. Eberle et al.^24^ proposed metazoan-level universal single-copy orthologs (mzl-USCOs) as markers for species delimitation. Mzl-USCOs have been evaluated using multiple data-generation and assembly strategies, combined with different species delimitation algorithms, across several study systems and organisms^22,26,47,58^. USCOs are defined as genes that are present and single-copy in at least 90% of a given lineage’s genomes^23^. Mzl-USCOs include 900–1,000 genes and are applicable to all metazoans^24,26,47^. Dietz et al^47^. extensively tested their empirical properties and showed that mzl-USCOs provide a representative sample of the protein-coding gene set of an organism, being distributed evenly over the genome. They are suitable to construct highly reliable phylogenies on higher systematic levels^26,47,58^ and are a very useful marker set for species-level taxonomy.

DNA of the lectotype was extracted using a morphologically non-destructive protocol, by separating the pronotum and abdomen from the pterothorax to allow the extraction buffer to reach as much muscle tissue of the body as possible. DNA extracted with the Qiagen blood and tissue kit (Qiagen, Hilden, Germany) was sheared with a Bioruptor PICO sonicator (Diagenode), until the fragments had a peak size of 300–400 bp. The NEBNext Ultra II DNA library prep kit (NEB) was used to generate Illumina libraries (end-prep, adapter ligation), combined with NEBNext 96 unique dual index primers (NEB). Paired-end Illumina (2 × 150 bp) sequencing was done by Macrogen Europe and yielded an output of 8–14 Gbp per specimen, as we aimed for a genome coverage of 5–10x – with an estimated genome size of 1.3 Gbp. DNA sequences of the target genes were identified and extracted as follows: in a first step, which was already performed for the earlier analyses^22^, we used Orthograph v0.7.1 with its default settings^59^ to extract DNA sequences orthologous to the target genes, i.e. the 978 mzl-USCOs^24,26^, from a transcriptome dataset of *Pachypus*sp^8^. Use of Orthograph required downloading the official gene sets (OGS) of all species included in the Metazoa OrthoDB v. 9 dataset^60^ and their corresponding hidden Markov models (HMMs) and information files from the BUSCO website (https://busco-archive.ezlab.org/v3/)^25^ and storing them in a SQLite database. In a second step, we used the DNA sequences of the mzl-USCOs extracted from the *Pachypus* transcriptome to map the raw reads obtained by low-coverage genome sequencing using the mapping software bwa v. 2.1^61^ with its default settings, except that we set the minimum seed length to 30. We then inferred diploid consensus sequences (i.e., a separate sequence for each mzl-USCO locus, with heterozygous sites represented by ambiguity codes) using samtools v. 1.10^62^ and bcftools v. 1.10.2 (https://github.com/samtools/bcftools)^63^ with default settings. We did the same for the raw reads of the transcriptome itself to determine its taxonomic position. As this approach recovers only sequences that were directly mapped to the coding reference sequences, introns and other non-coding regions were removed automatically. These sequences were then combined with homologous sequences of 171 *Pachypus* individuals for which they had already been obtained by targeted enrichment^22^. Since bwa had already aligned the DNA sequences to the *Pachypus* transcriptome reference sequence, no further DNA alignment steps were necessary for the subsequent phylogenomic analyses.

### Data extraction of COI 5’ data

To enhance applied DNA-based studies such as metabarcoding, we extracted the sequences of the 5’ COI standard DNA barcoding fragment from the raw reads of our target enrichment data^22^, from the newly sequenced lectotype of *P. impressus*, and the transcriptome sequenced earlier^8^ and compared their resulting ML tree topology to that of the 3’ COI data^5^. For this extraction, we used the software MitoGeneExtractor 1.9.3^64^. After initial trials with the complete extracted dataset, we decided to proceed further only with specimens achieving at least 45% nucleotide completeness, since otherwise the tree topology could be compromised. Based on the extracted 5’ COI data, we also performed BLAST searches in existing barcode reference libraries such as BOLD (v3) and NCBI. Sequences retrieved from these searches, that matched any *Pachypus* species, were incorporated into our subsequent phylogenetic analysis.

### Phylogenetic analysis

COI nucleotide sequences were aligned using MAFFT^65^(https://mafft.cbrc.jp/alignment/server/). Phylogenetic trees from COI data were inferred using PhyML^66^ via the web interface (http://www.atgc-montpellier.fr/phyml/) and automatic model selection^67^.

To determine the phylogenetic position of the lectotype of *P. impressus*, we analyzed the novel data together with the mzl-USCO dataset^22^. For this purpose, the mzl-USCO multiple nucleotide sequence alignments including all individuals were concatenated using a custom Perl script^58^. The resulting supermatrix was then used to infer a maximum likelihood phylogeny using IQ-TREE v. 2.1.2^68^. The datasets were first partitioned by gene, and the optimal combination of model and partitioning scheme was obtained with ModelFinder, using the IQ-TREE option -m MFP+MERGE^69,70^. Branch support was assessed by ultrafast bootstrapping^71^ from 1,000 replicates. For the same dataset, we also conducted a coalescent-based phylogenetic analysis with ASTRAL v. 5.6.1^72^. For this, we first constructed phylogenetic trees for each gene using IQ-TREE and determined the optimal model using ModelFinder as described above. These gene trees were then used as input to construct a coalescent-based tree with ASTRAL.

### Morphometric analysis

To investigate species’ morphology, we performed a morphometric analysis and considerably expanded the sampling from Eberle et al.^5^ and Dietz et al.^22^ including a total of 1,852 specimens. We used five datasets: one of “traditional morphometrics” including eight linear distance measurements of the body (Fig. 1, Supplementary file 2) and four of shape traits (outlines of aedeagus, clypeus, elytron and pronotum; Fig. 1). Distance measurements were taken directly from the specimen using an ocular grid on a stereomicroscope. Body parts were measured with both endpoints in focus at the same time to ensure horizontal orientation. For outline analysis, images of each body part were captured with a Nikon digital camera DXM1200 (pronotum, elytra) or a Leica digital camera DFC 420, mounted on the Leica stereomicroscope SM-125 (aedeagus, clypeus).

Size is the main source of variation in most morphometric measurement datasets^73–75^ and can confound species-specific shape variation. Also, conspecific specimens of *Pachypus* vary considerably in size. Size is thus not suitable to discriminate between *Pachypus* species. Therefore, we employed variables that exclusively represent shape. Following Eberle et al.^5^, we used the Burnaby-Back-projection method with the isometric size vector^74^ to extract shape parameters from linear measurements. Shape traits were superimposed on the outlines by Generalized Procrustes analyses. For this purpose, images of each body part were captured with a Nikon digital camera DXM1200 (pronotum, elytra) or a Leica digital camera DFC 420, mounted on the Leica stereo-microscope SM-125 (aedeagus, clypeus). Objects were positioned during imaging with the outline in focus to ensure standardized orientation and 100 equidistant semilandmarks were digitized along the selected outline (Fig. 1) in tpsDig (v. 2.17)^76^. Generalized Procrustes analyses were performed using the R package geomorph (v. 3.0.0)^77^. For closed curves (elytra, clypeus) all but the first landmark were defined as sliding landmarks (semilandmarks). For open curves (parameres, pronotum) all but the first and last landmarks were defined as sliding landmarks. Procrustes distance was used to optimize positions of the sliding landmarks^78^ and shapes were aligned by principal axes^79,80^.

### Species delimitation and identification

Given the cryptic nature of the species revealed by previous works^5,22^, species boundaries were primarily inferred based on the set of 978 mzl-USCO genes^22^. Subsequently, identification of non-sequenced specimens was based on the *i*ntegrative analysis of *U*SCOs and *m*orphological evidence including *g*eographic occurrence patterns of lineages inferred from USCO genes (abbreviated: identification based on IUMG). We used wider geographical perimeters around the known occurrence of a species (e.g. Sicily, Calabria, Corsica, or Central Italy), but not for Sardinian species. In cases of doubt due to syntopic occurrence of two or more species and lack of morphological distinctiveness, we refrained from a conclusive identification of the specimen (see Discussion for information and justification of this conservative approach). The distribution maps of the *Pachypus* species were generated using SimpleMappr^81^. 

### Nomenclatural acts

The electronic edition of this article conforms to the requirements of the amended International Code of Zoological Nomenclature^32^, and hence the new names contained herein are available under that Code from the electronic edition of this article. This published work and the nomenclatural acts it contains have been registered in ZooBank (https://zoobank.org/), the online registration system for the ICZN. The LSID for this publication is: https://zoobank.org/urn:lsid:zoobank.org:pub:34F3F1E1-2A40-4450-95B3-0336E6E2CF52. The electronic edition of this paper was published in a journal with an ISSN, has been archived, and is available from PubMed Central.

## Supplementary Information

Below is the link to the electronic supplementary material.

Supplementary Material 1

Supplementary Material 2

Supplementary Material 3

Supplementary Material 4

Supplementary Material 5

Supplementary Material 6

Supplementary Material 7

Supplementary Material 8

Supplementary Material 9

Supplementary Material 10

Supplementary Material 11

## Data Availability

The type series and all other examined specimens are deposited in the indicated collections (see Material and Methods) and are publicly available. The new COI and USCO nucleotide sequences generated in this study are deposited in GenBank (https://www.ncbi.nlm.nih.gov/genbank/). The GenBank accession numbers of all new nucleotide sequences generated and used in this study are presented in Supplementary Table S1. The distribution data are available on GBIF (www.gbif.org) under the project *An integrative taxonomy of cryptic Pachypus chafers.* (https://doi.org/10.15468/xb63s3). Other raw data (e.g., primary images, measurements, tps files of morphometric data, etc.) are available upon reasonable request to the corresponding author.
