# Supplementary material for: Integrative taxonomy of cryptic Pachypus chafers using museomics, morphometrics, barcoding, and genomic DNA analysis (Coleoptera: Scarabaeidae: Pachypodinae)
Source: Sci Rep. 2026 May 20;16:15710. doi: 10.1038/s41598-026-47761-7 (PMC13190838; doi:10.1038/s41598-026-47761-7)
Supplement: Supplementary file 9 — Supplementary Material 9 [file 41598_2026_47761_MOESM9_ESM.pdf]

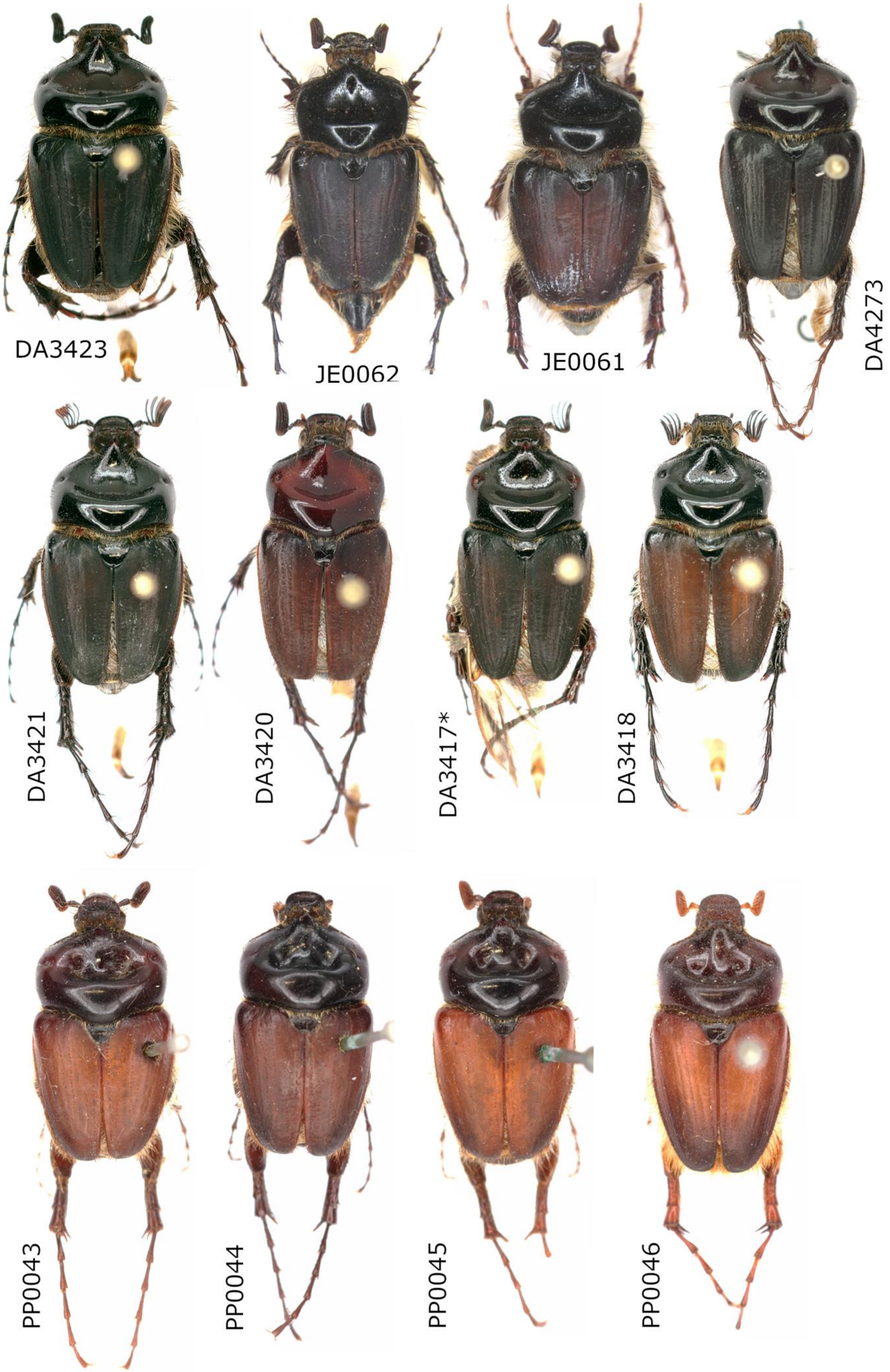
DA3423
JE0062
JE0061
DA4273
DA3421
DA3420
DA3417*
DA3418
PP0043
PP0044
PP0045
PP0046

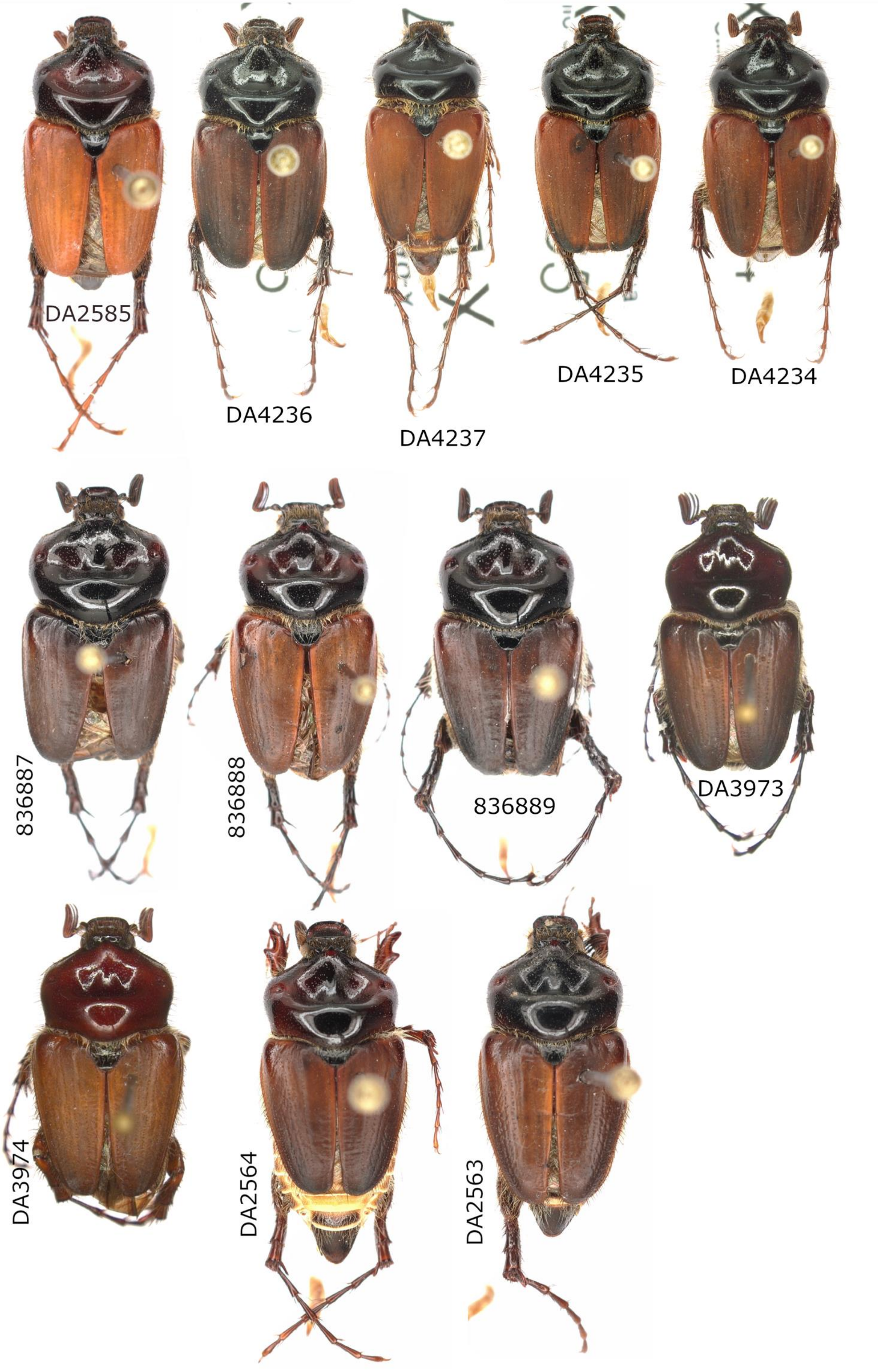
DA2585
DA4236
DA4237
DA4235
DA4234
836887
836888
836889
DA3973
DA3974
DA2564
DA2563

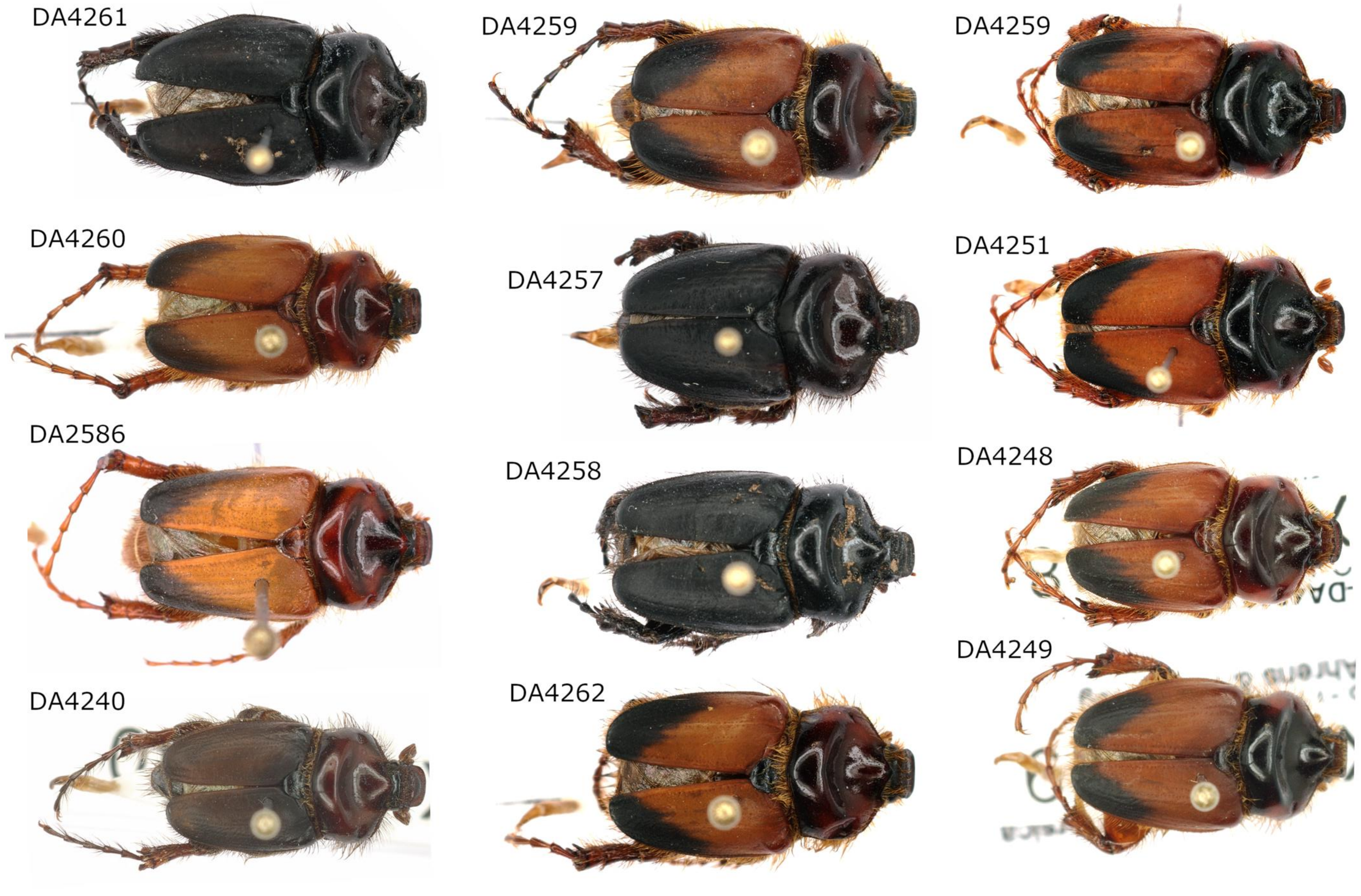
DA4261
DA4259
DA4259
DA4260
DA4257
DA4251
DA2586
DA4258
DA4248
DA4240
DA4262
DA4249

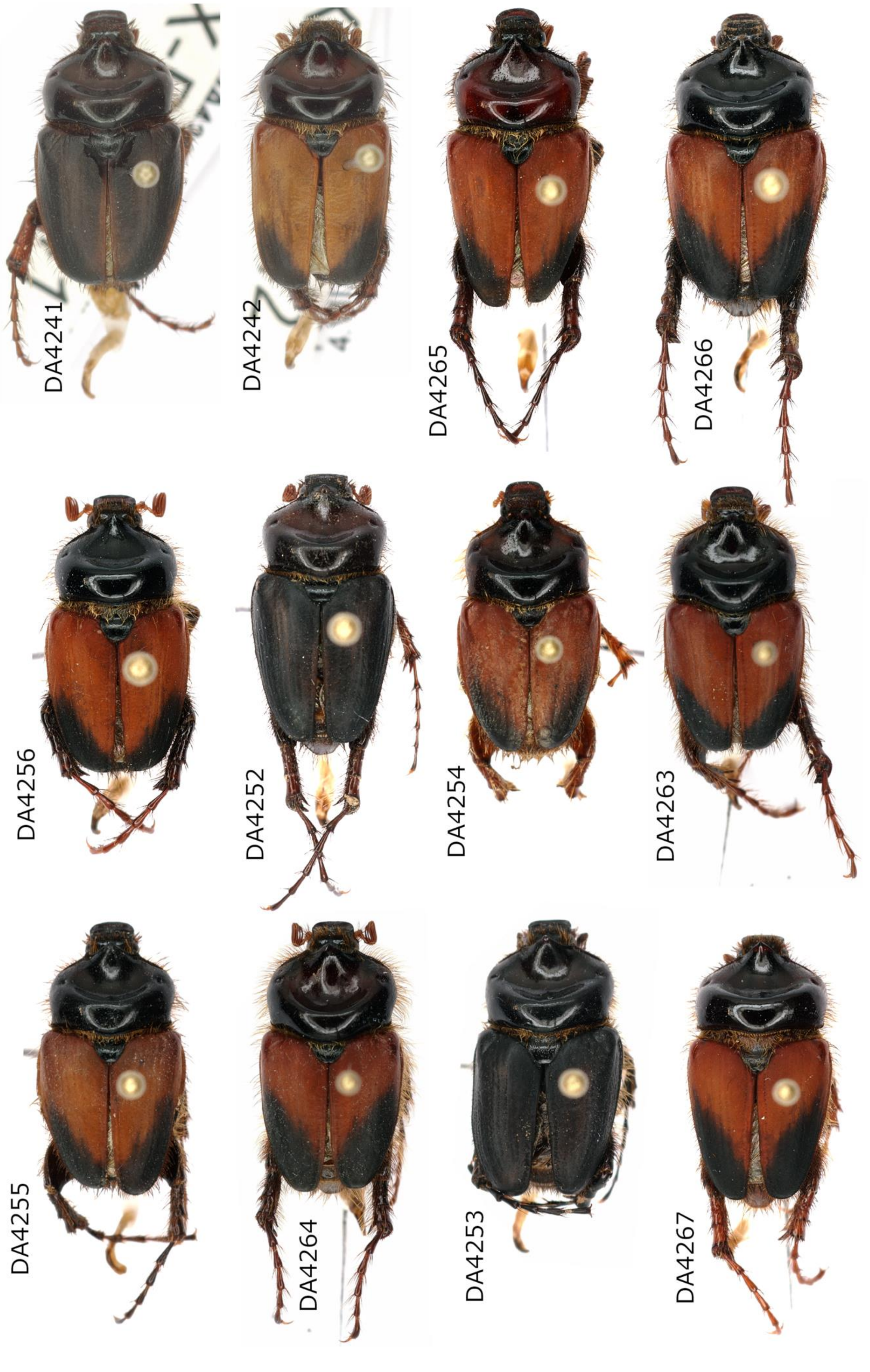

DA4241
DA4242
DA4265
DA4266
DA4256
DA4252
DA4254
DA4263
DA4255
DA4264
DA4253
DA4267

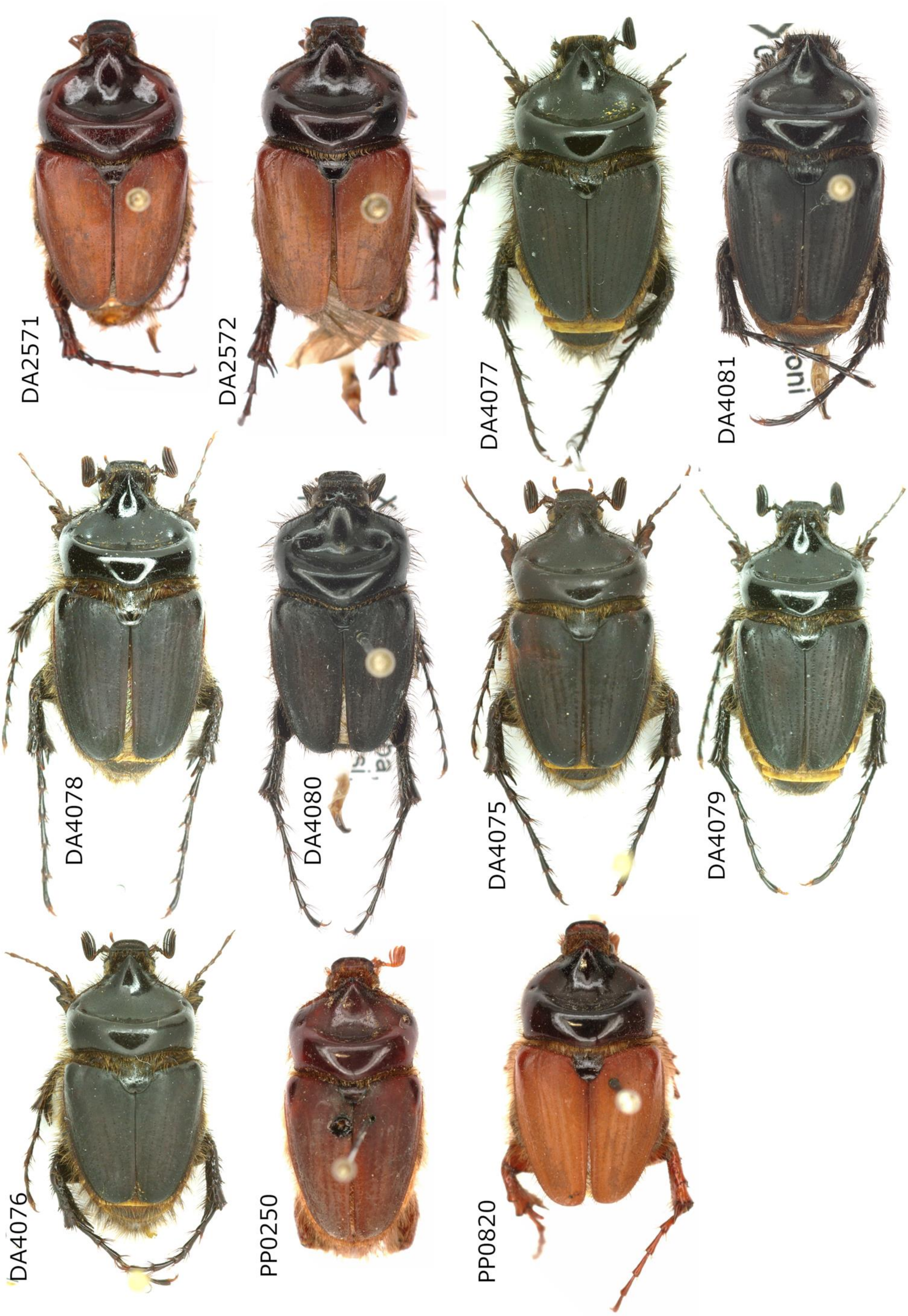
DA2571
DA2572
DA4077
DA4081
DA4078
DA4080
DA4075
DA4079
DA4076
PP0250
PP0820

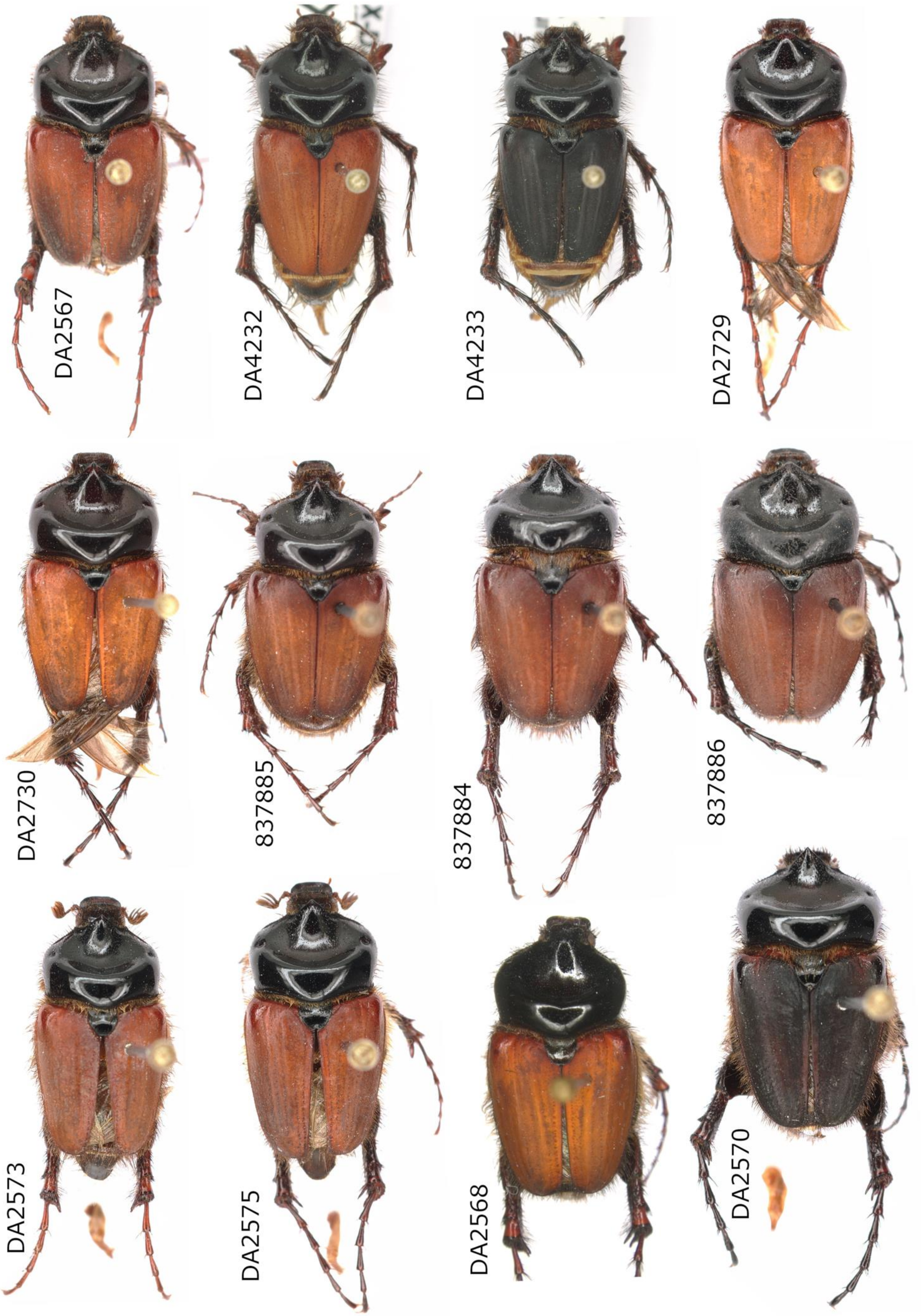
DA2567
DA4232
DA4233
DA2729
DA2730
837885
837884
837886
DA2573
DA2575
DA2568
DA2570

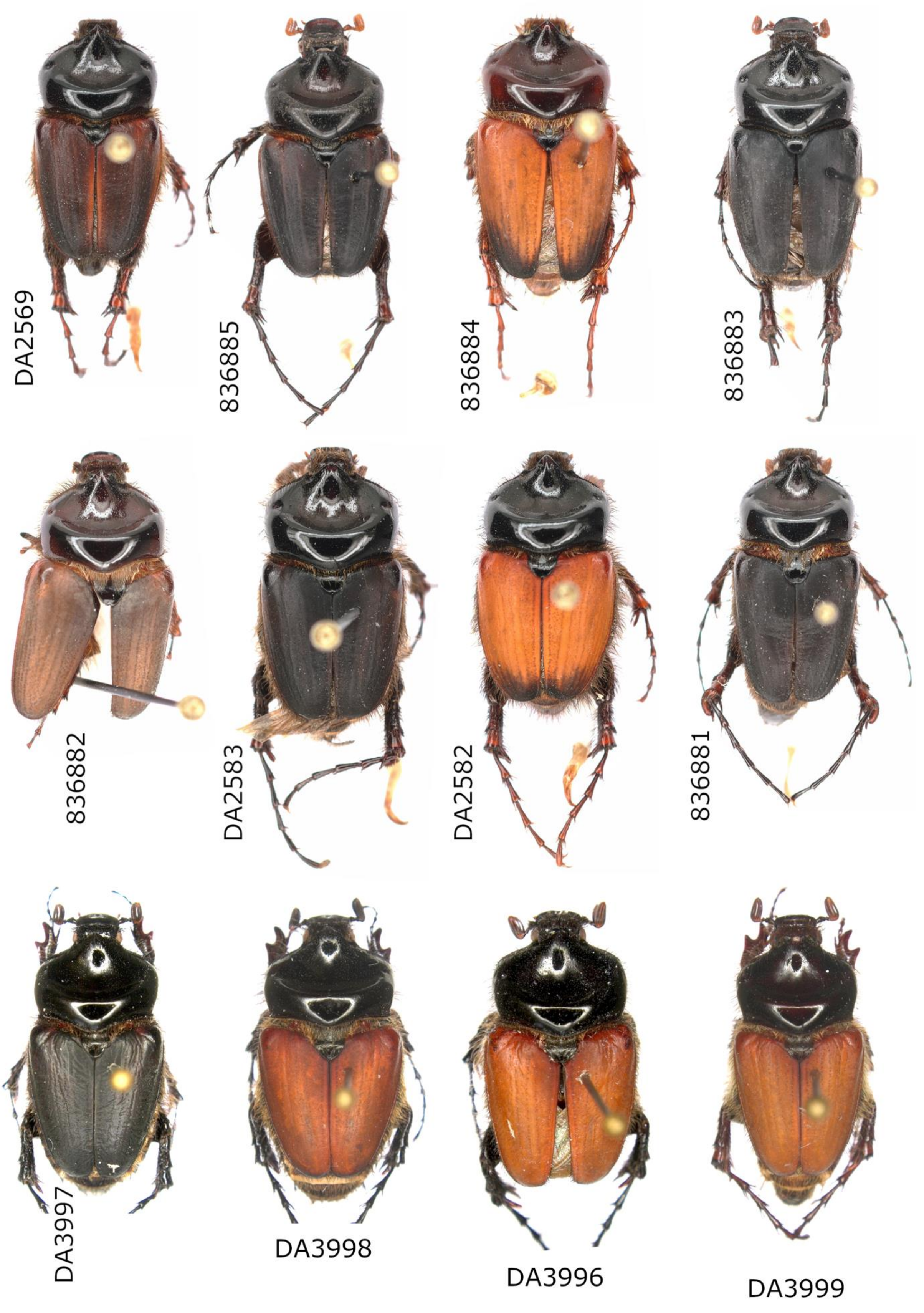
DA2569
836885
836884
836883
836882
DA2583
DA2582
836881
DA3997
DA3998
DA3996
DA3999

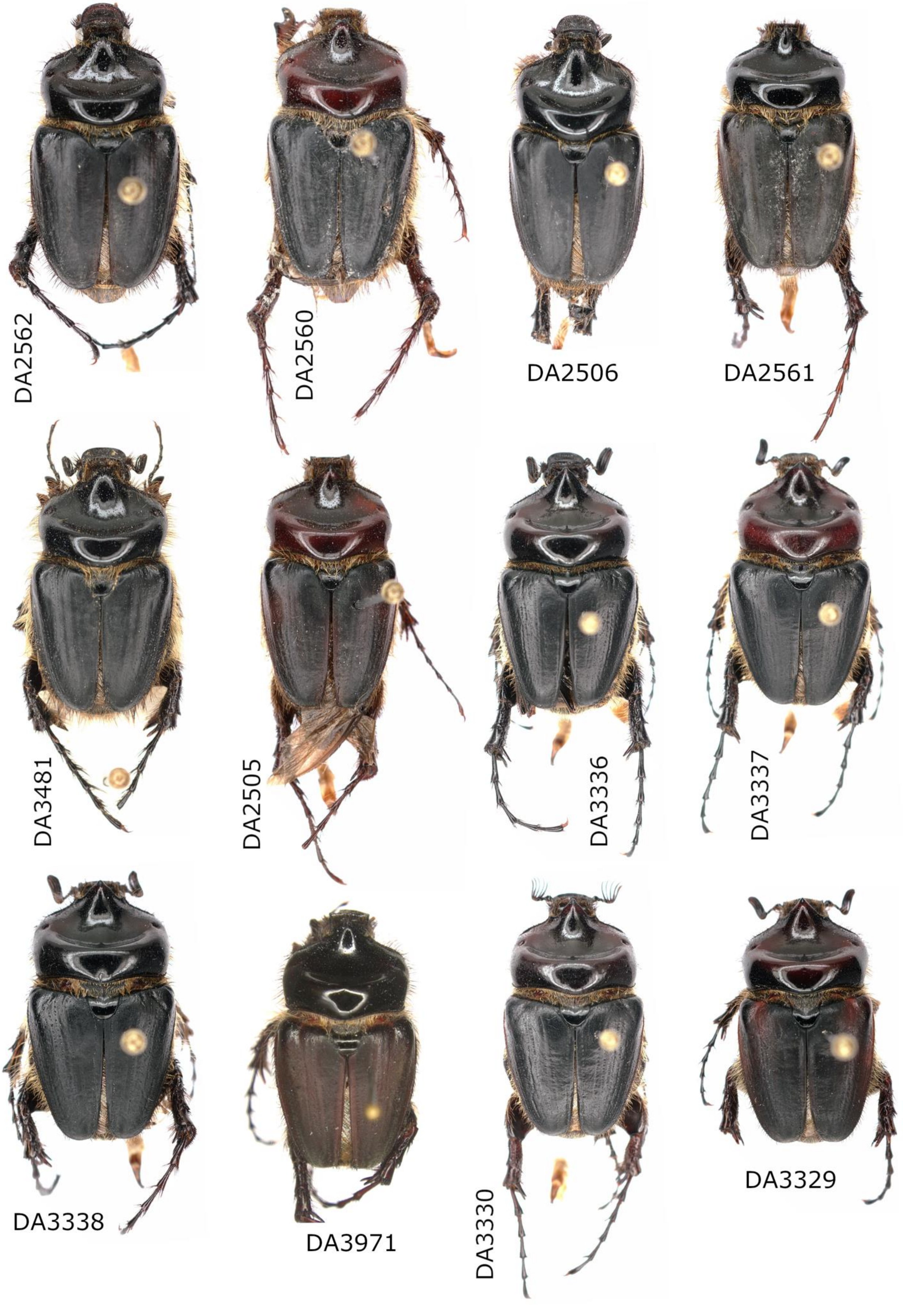

DA2562
DA2560
DA2506
DA2561
DA3481
DA2505
DA3336
DA3337
DA3338
DA3971
DA3330
DA3329

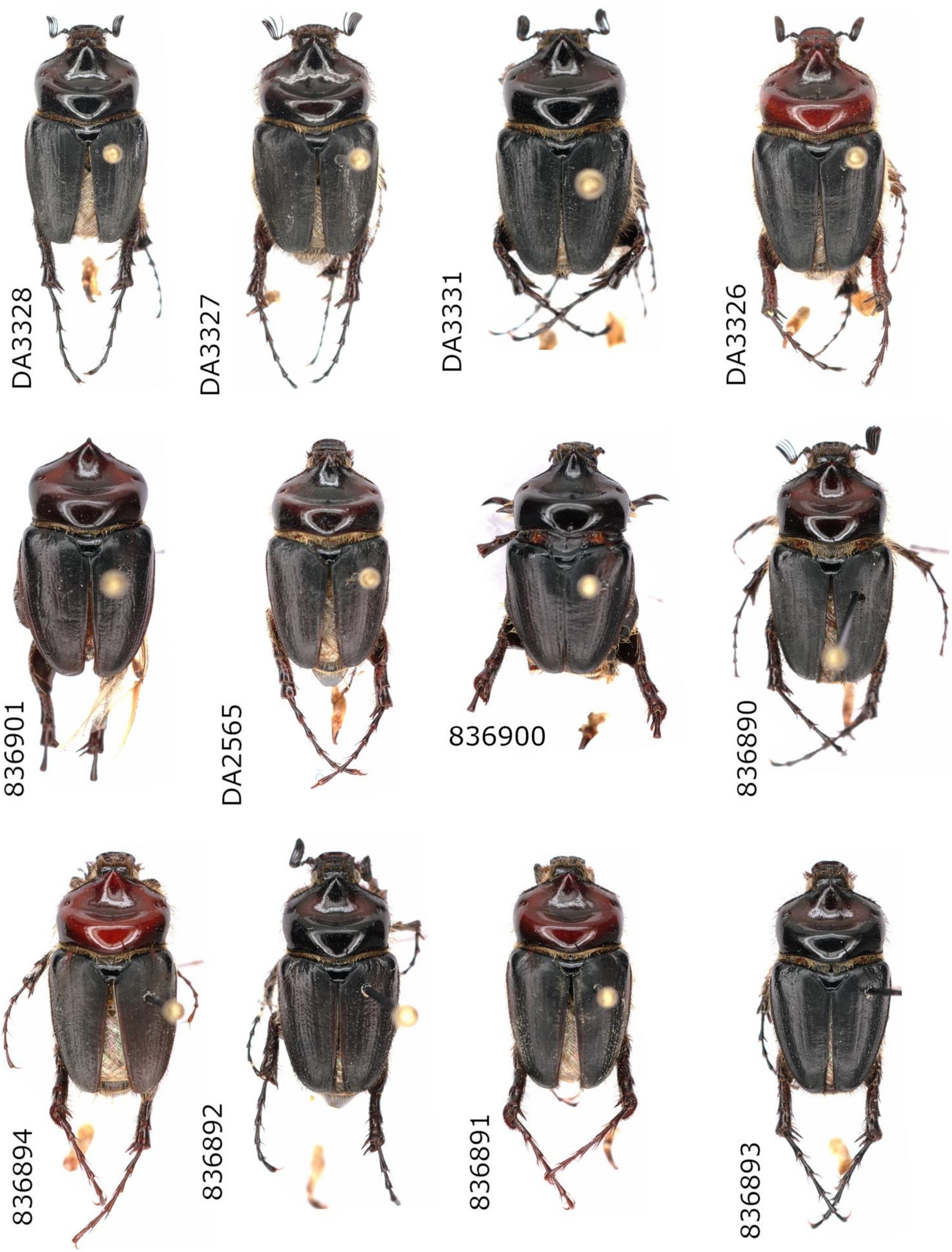

DA3328
DA3327
DA3331
DA3326
836901
DA2565
836900
836890
836894
836892
836891
836893

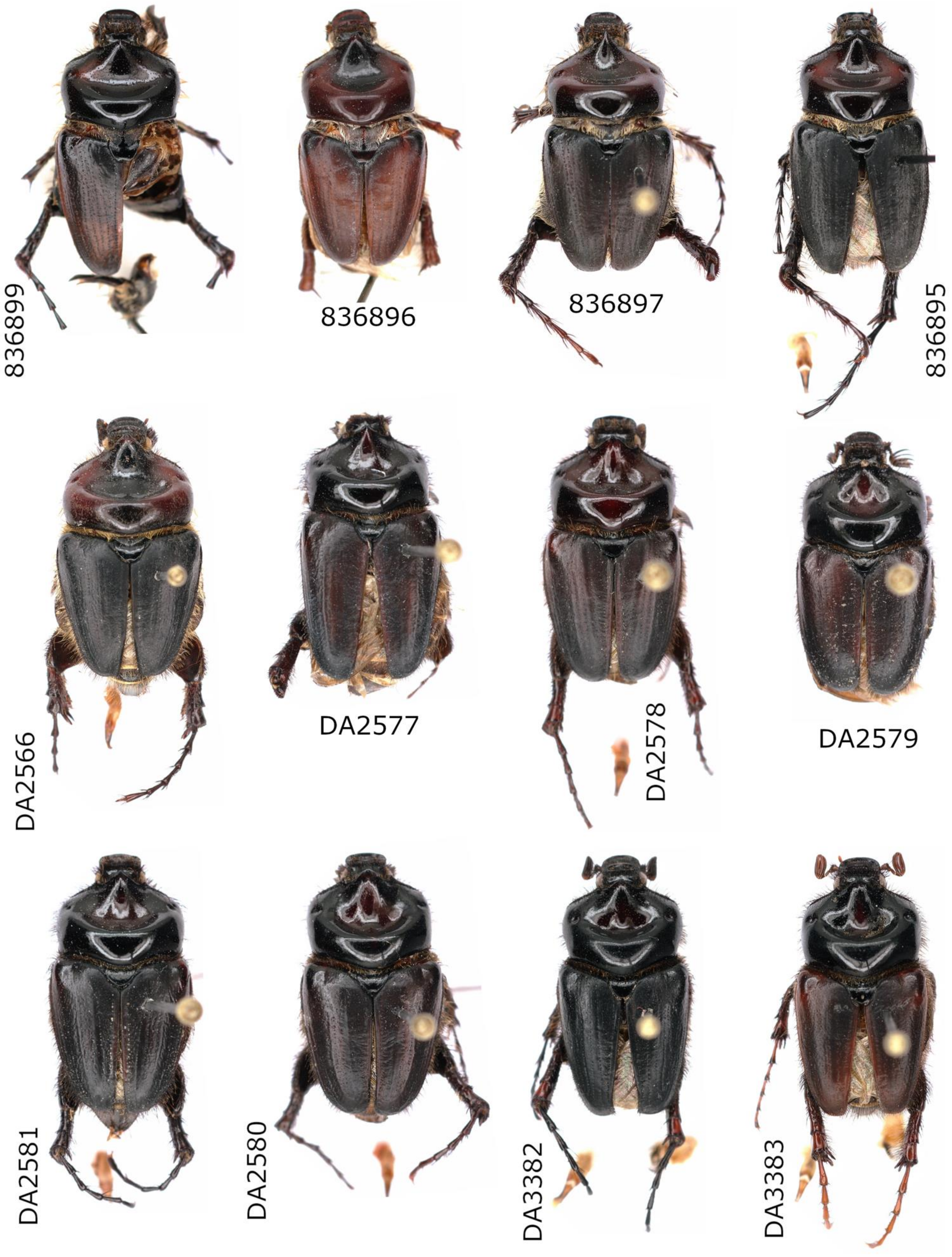
836899
836896
836897
836895
DA2566
DA2577
DA2578
DA2579
DA2581
DA2580
DA3382
DA3383

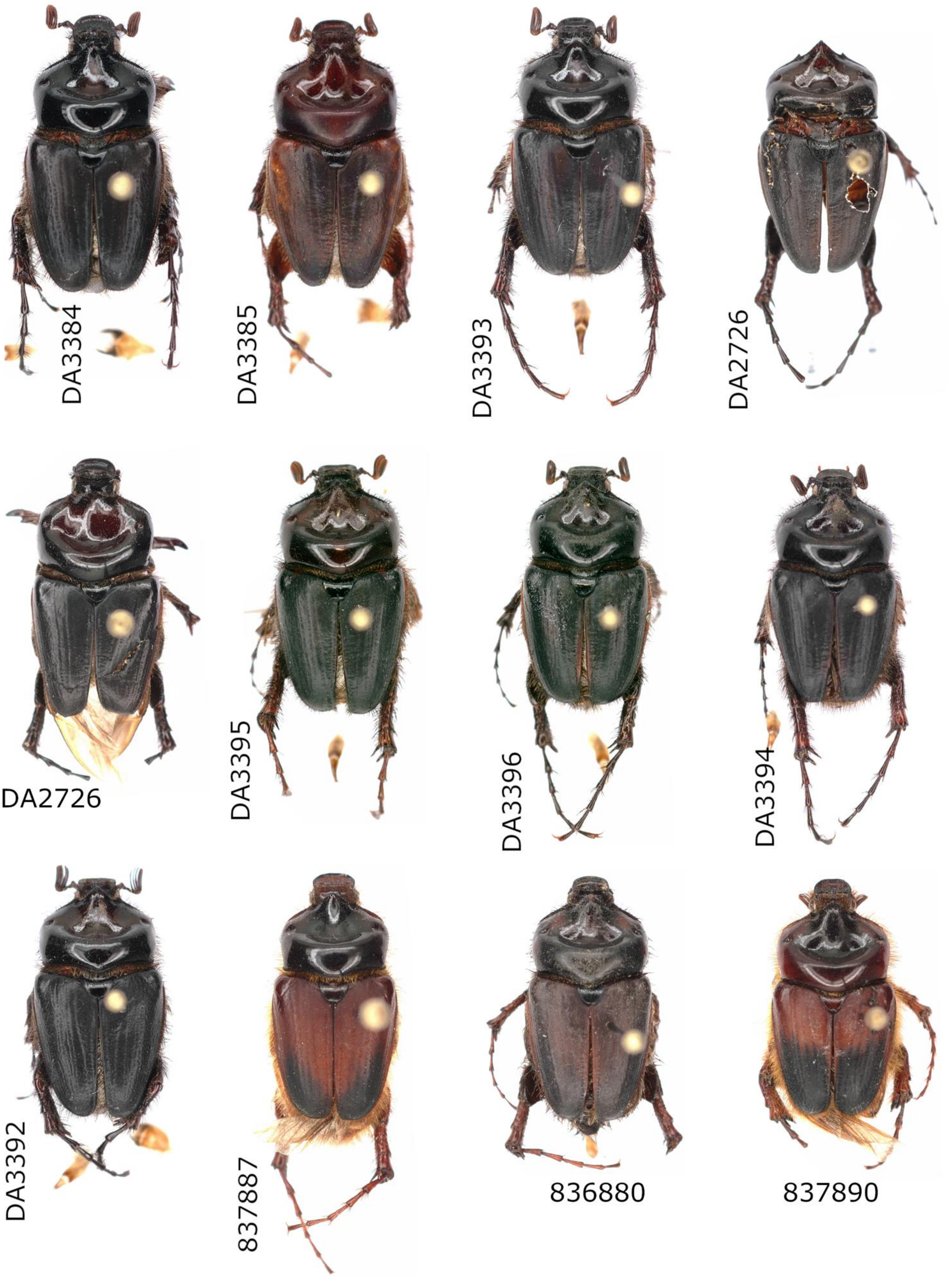
DA3384
DA3385
DA3393
DA2726
DA2726
DA3395
DA3396
DA3394
DA3392
837887
836880
837890

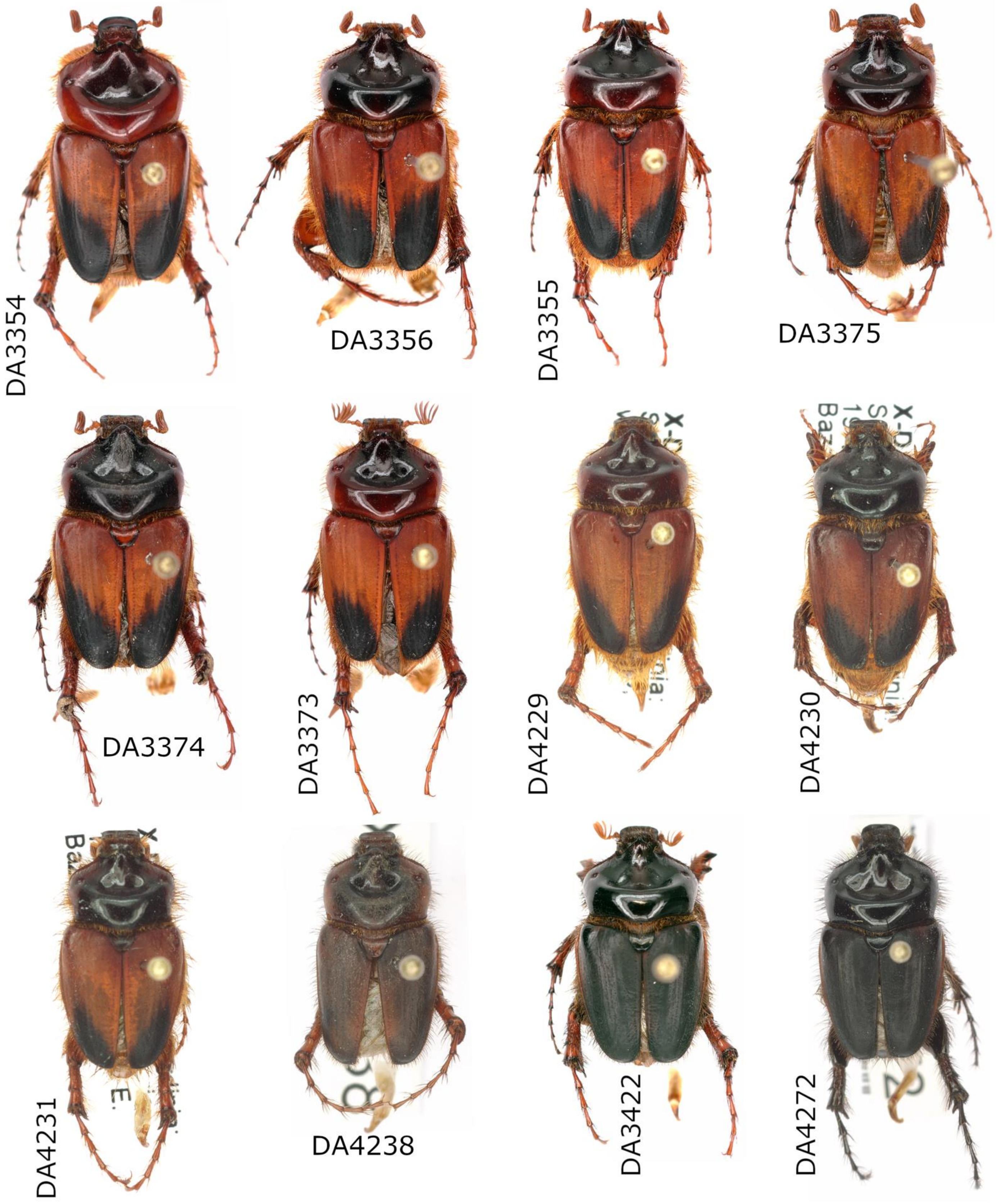

DA3354
DA3356
DA3355
DA3375
DA3374
DA3373
DA4229
DA4230
DA4231
DA4238
DA3422
DA4272

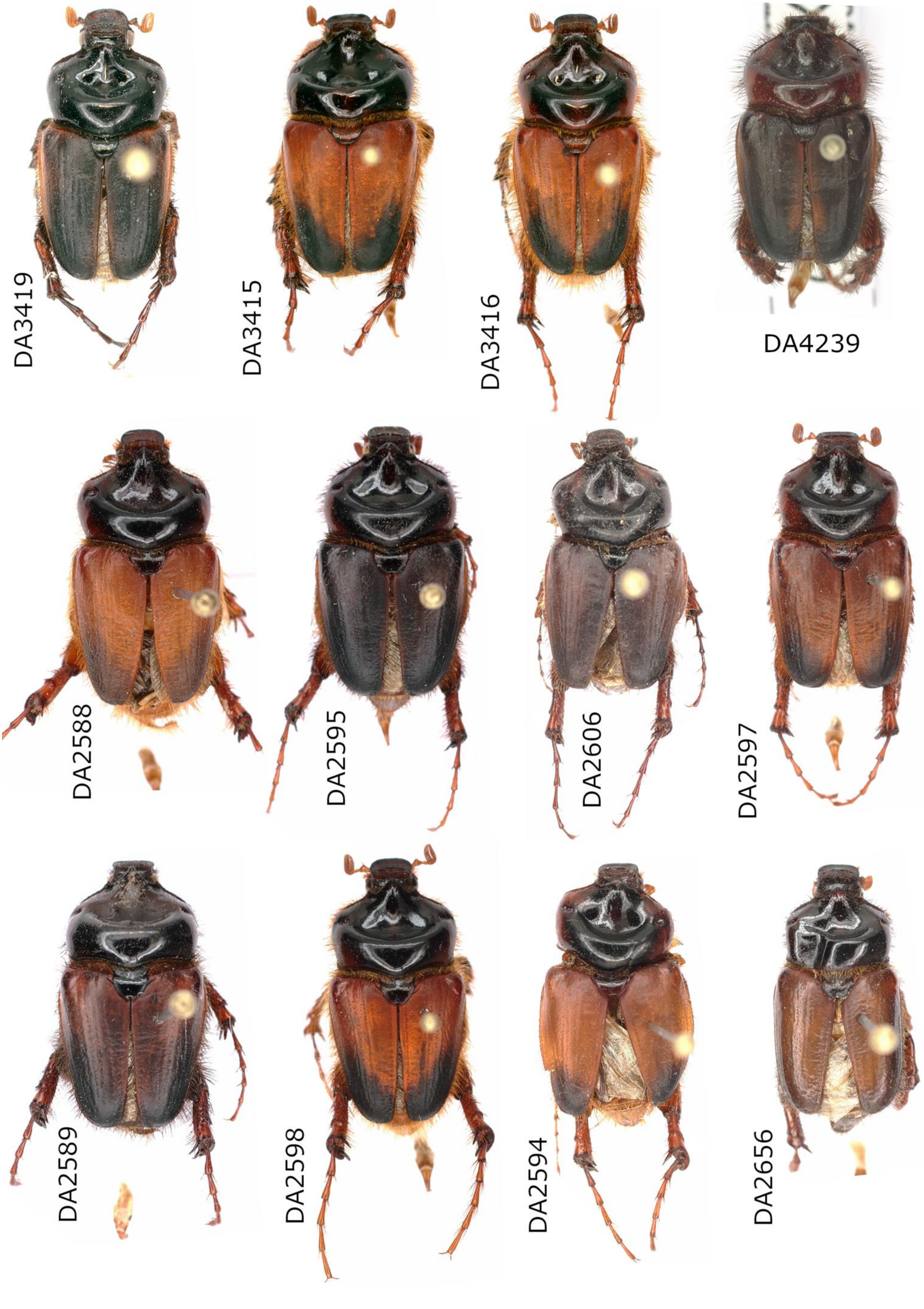
DA3419
DA3415
DA3416
DA4239
DA2588
DA2595
DA2606
DA2597
DA2589
DA2598
DA2594
DA2656

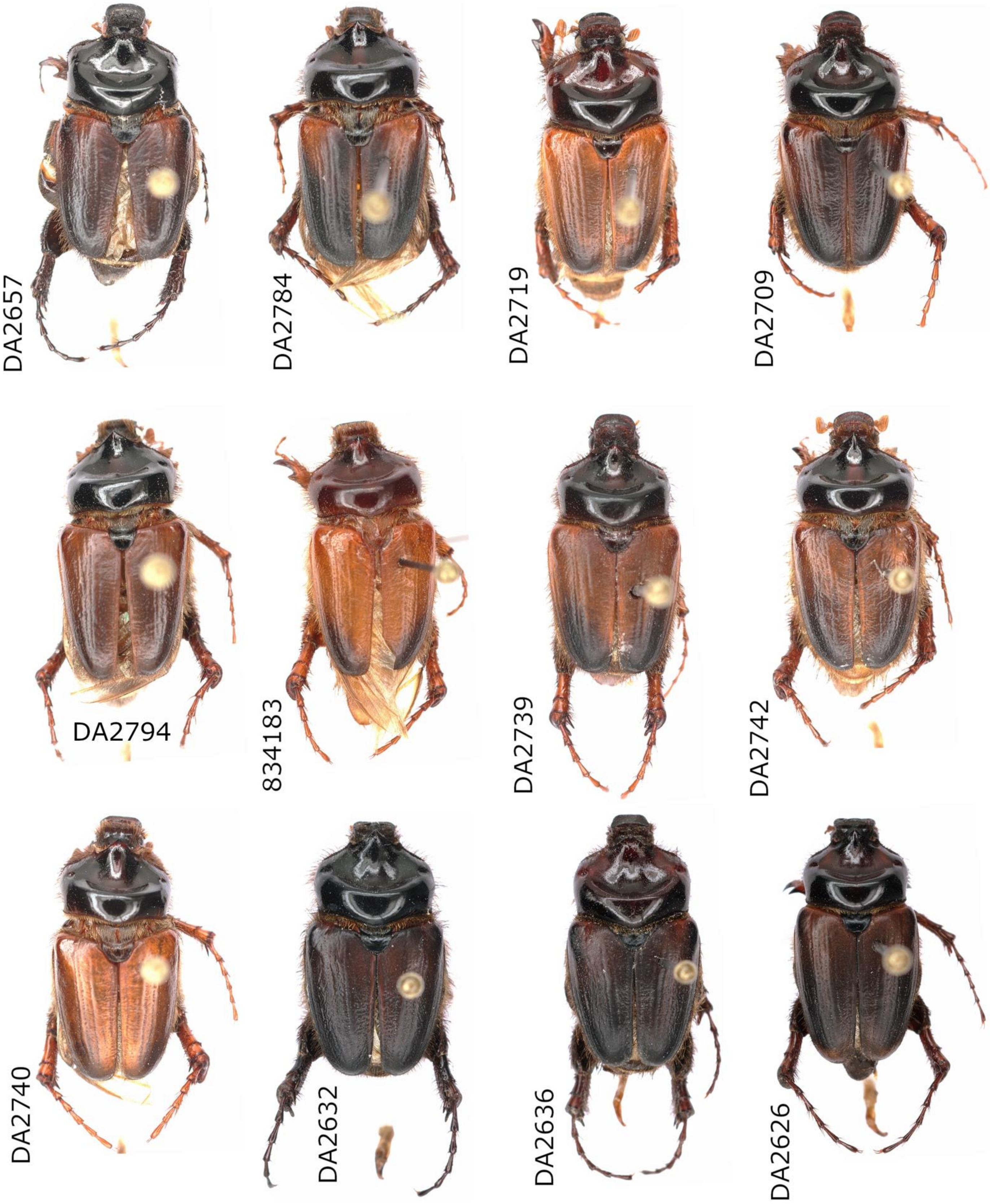
DA2657
DA2784
DA2719
DA2709
DA2794
834183
DA2739
DA2742
DA2740
DA2632
DA2636
DA2626

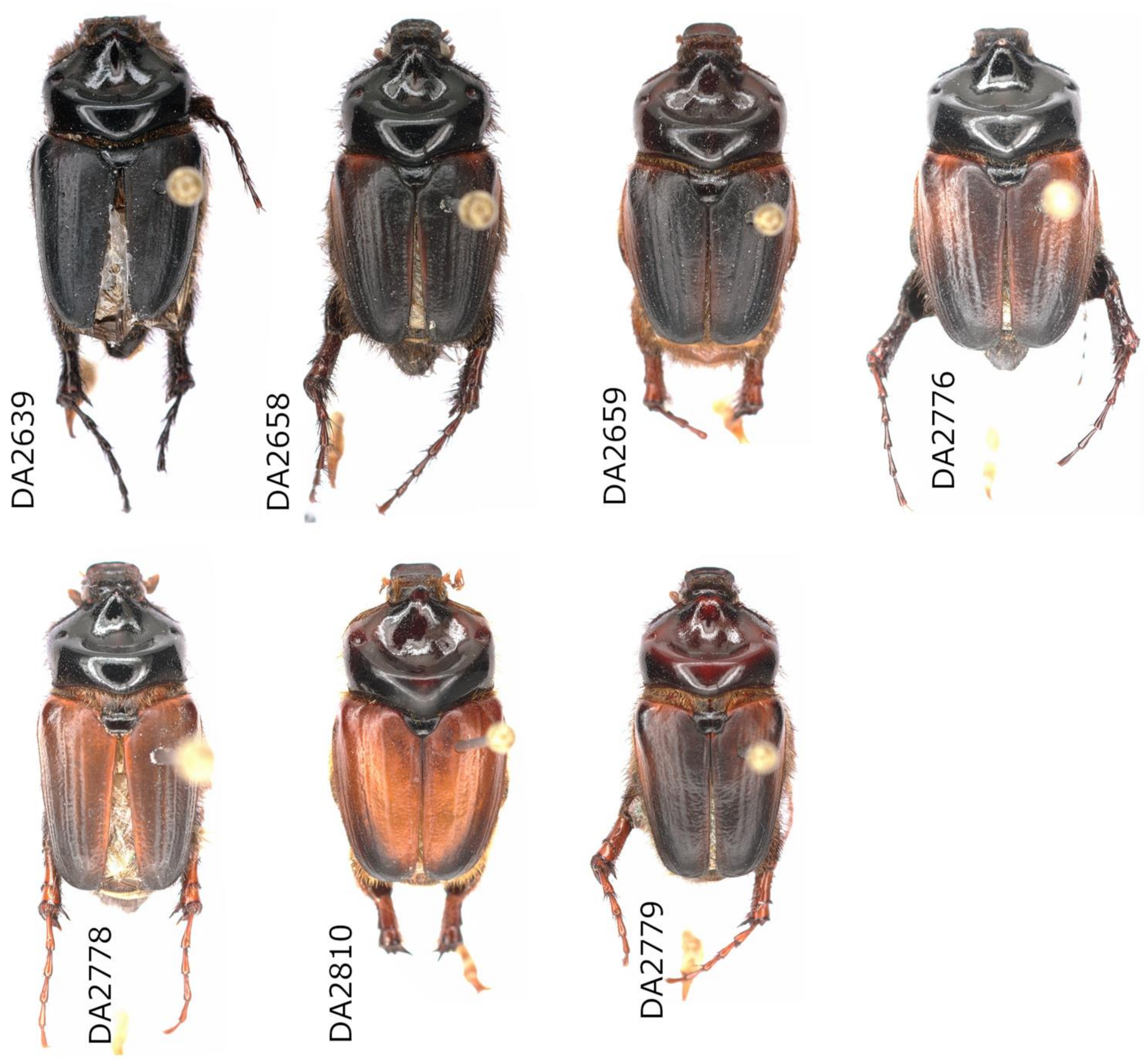

DA2639
DA2658
DA2659
DA2776
DA2778
DA2810
DA2779
