# Supplementary figures and images for: Integrative taxonomy of cryptic Pachypus chafers using museomics, morphometrics, barcoding, and genomic DNA analysis (Coleoptera: Scarabaeidae: Pachypodinae)
Source: Sci Rep. 2026 May 20;16:15710. doi: 10.1038/s41598-026-47761-7 (PMC13190838; doi:10.1038/s41598-026-47761-7)

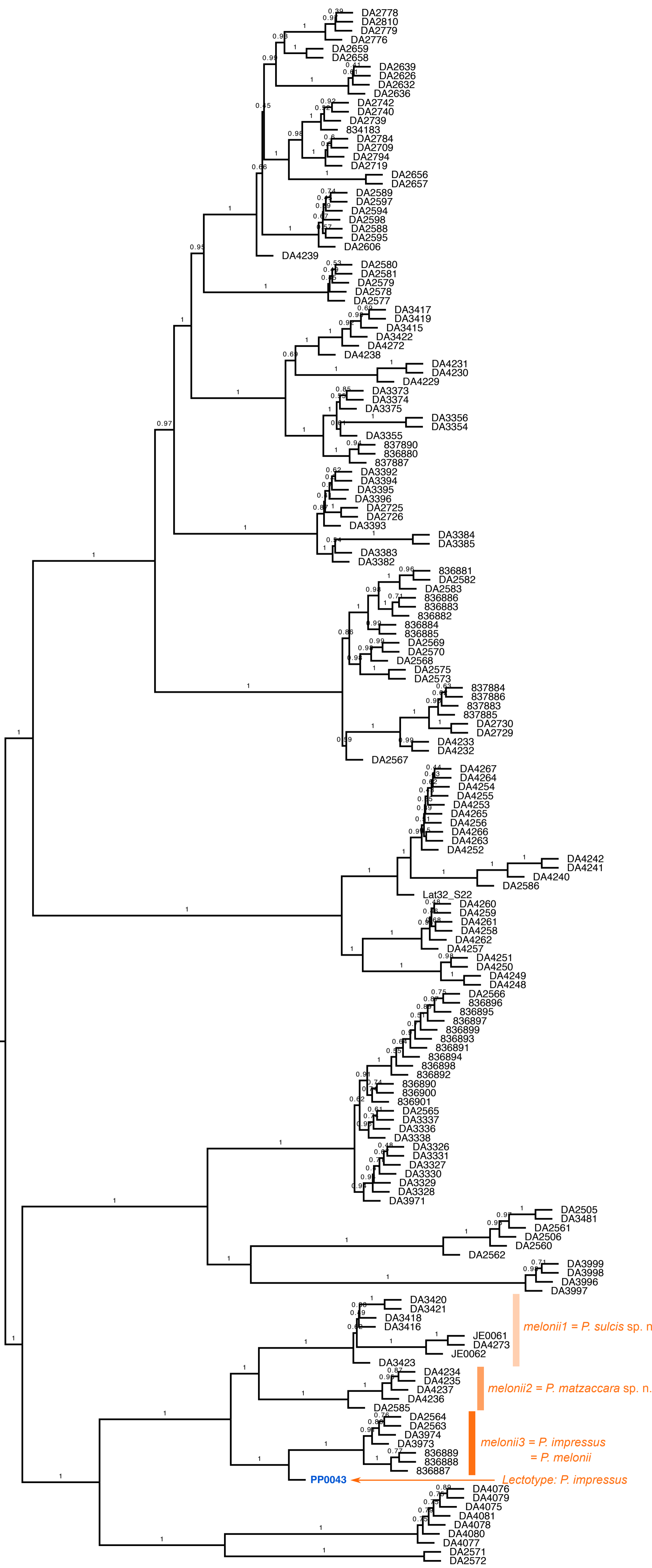

Supplement: Supplementary file 10 — Supplementary Material 10 [file 41598_2026_47761_MOESM10_ESM.pdf]
