# Supplementary material for: Integrative taxonomy of cryptic Pachypus chafers using museomics, morphometrics, barcoding, and genomic DNA analysis (Coleoptera: Scarabaeidae: Pachypodinae)
Source: Sci Rep. 2026 May 20;16:15710. doi: 10.1038/s41598-026-47761-7 (PMC13190838; doi:10.1038/s41598-026-47761-7)
Supplement: Supplementary file 11 — Supplementary Material 11 [file 41598_2026_47761_MOESM11_ESM.pdf]

Supplement File 1

Material and methods

Abbreviations used in the text for collection depositories are as follows:

CALC	Coll. A. Lecis, Cagliari, Italy;
CAMM	Coll. A. Marata, Monte San Giusto, Macerata, Italy;
CCAU	Coll. C. Ancona, Ussana, Italy;
CDCC	Coll. D. Cillo, Cagliari, Italy;
CEBQ	Coll. E. Bazzato, Quartu S.E., Italy;
CFAQ	Coll. F. Alamanni, Quartucciu, Italy;
CGSG	Coll. G. Sabatinelli, Geneva, Switzerland;
CISP	Coll. I. Sparacio, Palermo, Italy;
CLFP	Coll. L. Forbicioni, Portoferraio, Italy;
CJMH	Coll. J. Matějíček, Hradec Králové, Czech Republic;
CMAC	Coll. M.G. Atzori, Cagliari, Italy;
CMBB	Coll. M. Bastianini, Bagno di Gavorrano, Italy;
CMUC	Coll. M. Uliana, Codevigo, Italy;
CSNC	Coll. S. Nappini, Castiglione della Pescaia, Italy;
CVGS	Coll. V. Gallerati, San Pietro in Casale, Italy;
HNHM	Hungarian Natural History Museum, Budapest, Hungary;
ISNB	Royal Belgian Institute of Natural Sciences Brussels, Belgium;
MHNG	Muséum d'Histoire Naturelle Geneva, Switzerland;
MSNM	Museo Civico di Storia Naturale, Milano, Italy;
MSNV	Museo di Storia Naturale Giancarlo Ligabue, Venezia, Italy;
NMPC	National Museum Prague, Czech Republic;
ZFMK	Museum Koenig Bonn, Leibniz Institute of Biodiversity Change;
ZMHB	Museum für Naturkunde, Berlin, Germany;
ZMUK	Zoologisches Museum, Universität Kiel.

Results

Further examined material for the species:

***Pachypus caesus* Erichson, 1840**

Additional material examined. (identification based on IUMG)**:** 1 ♂ "Sicilien A. Botteher Berlin / Favo 18.10 04 Vitale/ PP0002" (ZFMK), 1 ♂ "Sicilien A. Botteher Berlin / Favo 18.10 04 Vitale/ PP0003" (ZFMK), 1 ♂ "Pachypus caesus Er. det. L. Haberäcker München / Sicilia Reitter / 2990 / 1 ♂ / PP0004" (ZFMK), 1 ♂ "1 ♂ / Sicilia/ PP0005" (ZFMK), 1 ♂ "caesus Er. Sicile / Sig. R. Oberthür Eing. Nr. 4, 1956/ PP0006" (ZFMK), 1 ♂ "caesus v. impressus / Messine / 9/57, ex coll. R. Oberthür/ PP0012" (ZFMK), 1 ♂ "caesus v. impressus / Sicile / Stef Perez/ PP0013" (ZFMK), 1 ♂ "P. impressus G. / Sicilien / Material der Coll. Moser definitiv nicht von Erichson untersucht/ PP0047"

(ZMHB), 1 ♂ "P. caesus Er. Sizilien / Sizilien; Pachypus caesus; Erichson, 1840 Ex. Coll. Missionshaus Steyl/ PP0256" (ZFMK), 1 ♂ "Sizilien; Pachypus caesus; Erichson, 1840 Ex. Coll. Missionshaus Steyl/ PP0257" (ZFMK), 1 ♂ "Sicilia - Italia (ME) Fraz. Croce 16-X-04 leg. F. Camino/ PP0467" (CDCC), 1 ♂ "Pachipus caesus / Sicilia - PA Parco della Favorita 8-X-96 leg. I. Sparacio/ PP0468" (CDCC), 1 ♂ "P. caesus / Palermo Favorita 30.IX.1973 leg. V. Aliquò/ PP0469" (CDCC), 1 ♂ "Sicilia (PA) M.te Pellegrino - F. Tana d'Ercole 20-X-10 leg. C. Muscarella/ PP0470" (CDCC), 1 ♂ "Palermo Favorita 18.X.73 leg. B. Massa / ex coll. N. Cabitta/ PP0472" (CDCC), 1 ♂ "Pachipus caesus / Favorita (PA) 30-IX-73 leg. Alirvo / ex coll. N. Cabitta/ PP0473" (CDCC), 1 ♂ "Sicilia Favorita E. Ragusa / Pachypus caesus/ PP0812" (CJMH), 1 ♂ "Sicilia Favorita E. Ragusa 10/ PP0813" (CJMH), 1 ♂ "Sicile 74 / E. Ragusa/ Coll. R. I. Sc. N. B. Italie / 9895/ PP0902" (ISNB), 1 ♂ "Sicile 74 / E. Ragusa / Coll. R. I. Sc. N. B. Italie / 9895/ PP0903" (ISNB), 1 ♂ "Sicile 74 / E. Ragusa / Coll. R. I. Sc. N. B. Italie / 9895/ PP0904" (ISNB), 1 ♂ "Sicile 74 / E. Ragusa/ Coll. R. I. Sc. N. B. Italie / M.R. Belg. / 9895/ PP0905" (ISNB), 1 ♂ "Sicile 74 / E. Ragusa / Coll. R. I. Sc. N. B. Italie / 9895/ PP0906" (ISNB), 1 ♂ "Sicile 74/ E. Ragusa / Coll. R. I. Sc. N. B. Italie / 9895/ PP0907" (ISNB), 1 ♂ "Sicile 74 / E. Ragusa / Coll. R. I. Sc. N. B. Italie / 9895/ PP0908" (ISNB), 1 ♂ "Sicile 74 / Coll. R. I. Sc. N. B. Italie / 9895/ PP0910" (ISNB), 1 ♂ "P. id. var. caesus Erichs Sicilie / Coll. R. I. Sc. N. B. Italie / M.R. Belg. / Coll. J. Thomson / 9895 / 41/ PP0912" (ISNB), 1 ♂ "Sicile 74 / Coll. R. I. Sc. N. B. Italie / 9895/ PP0915" (ISNB), 1 ♂ "Sicile 74 / Coll. R. I. Sc. N. B. Italie / 9895/ PP0916" (ISNB), 1 ♂ "Sicile 74 / Coll. R. I. Sc. N. B. Italie / M.R. Belg. / 9895/ PP0917" (ISNB), 1 ♂ "Sicilia / Coll. R. I. Sc. N. B. Italie / ex coll. Sirguey Le Moult vend.:/ PP0919" (ISNB), 1 ♂ "Messina / Coll. R. I. Sc. N. B. Italie / ex coll. Sirguey Le Moult vend.:/ PP0921" (ISNB), 1 ♂ "Sicile / Coll. R. I. Sc. N. B. Italie / ex coll. R.P. David ex coll. Pères Jésuites (Le Moult vendit)/ PP0922" (ISNB), 1 ♂ "Pach. caesus Sicilia Mondello 4/10 Füge / Coll. R. I. Sc. N. B. Italie / Coll. P.J. Roelofs R.I.Sc.N.B. I.G. 18.856 / 720/ PP0923" (ISNB), 1 ♂ "Pachypus caesus Er. / Sicilien / Coll. R. I. Sc. N. B. Italie / J.J. Gillet det., vend.: R.M.H.N. Belg. 10.640/ PP0924" (ISNB), 1 ♂ "caesus Er. / Sicilia Reitter. / Coll. R. I. Sc. N. B. Italie/ PP0925" (ISNB), 1 ♂ "Coll. R. I. Sc. N. B. Italie Sicilia Palermo coll. de Bonneuil Le Moult vendit/ PP0926" (ISNB), 1 ♂ "Sicilia F. Vitale/ PP1019" (MHNG), 1 ♂ "Sicilia Palermo/ PP1020" (MHNG), 1 ♂ "excavata Fab cornuta Oliv. Tar Atra Nel tvsn. An Sicile/ PP1022" (MHNG), 1 ♂ "impressus Er. / Sicili / Coll. Maerky/ PP1023" (MHNG), 1 ♂ "Sicili / Coll. Maerky/ PP1024" (MHNG), 1 ♂ "Sicili / Coll. Maerky/ PP1025" (MHNG), 1 ♂ "Sicili / Coll. Maerky/ PP1026" (MHNG), 1 ♂ "caesus Er. / Pachypus caesus Er. det. Petrovitz / La Favorita Sicile / Coll. Petrovitz/ PP1027" (MHNG), 1 ♂ "Pachypus caesus Er. det. Petrovitz / Sicilia La Favorita 9/10 Füge / Coll. Petrovitz/ PP1028" (MHNG), 1 ♂ "Pachypus caesus Er. det. Petrovitz / La Favorita Sicile / Coll. Petrovitz/ PP1029" (MHNG), 1 ♂ "Pachypus caesus Er. det. Petrovitz / Sicili / Coll. Petrovitz/ PP1030" (MHNG), 1 ♂ "Pachypus caesus Er. det. Petrovitz / Sicilien / Coll. Petrovitz/ PP1031" (MHNG), 1 ♂ "Sicilia (PA) Parco della Favorita 4.X.1998 leg. I. Sparacio/ PP1032" (CISP), 1 ♂ "Sicilia (PA) Parco della Favorita 4.X.1998 leg. I. Sparacio/ PP1033" (CISP), 1 ♂ "Sicilia (PA) Parco della Favorita 11.X.1998 leg. I. Sparacio/ PP1034" (CISP), 1 ♂ "Sicilia (PA) Parco della Favorita 8.X.1978 leg. I. Sparacio/ PP1035" (CISP), 1 ♂ "Sicilia (PA) Parco della Favorita 8.X.1978 leg. I. Sparacio/ PP1036" (CISP), 1 ♂ "Sicilia Parco della Favorita (PA) 4.XI.1979 leg. I. Sparacio/ PP1037"

(CISP), 1 ♂ "Sicilia (PA) Sferracavallo 30.IX.1979 leg. I. Sparacio/ PP1038" (CISP), 1 ♂ "Sicilia (PA) Sferracavallo 5.X.1979 leg. I. Sparacio/ PP1039" (CISP), 1 ♂ "Sicilia (PA) Cinisi 3.X.2001 leg. I. Sparacio/ PP1040" (CISP), 1 ♂ "Sicilia (PA) Cinisi 3.X.2001 leg. I. Sparacio/ PP1041" (CISP), 1 ♂ "Sicilia (PA) Terrasini X.1999 leg. F. Vitale/ PP1042" (CISP), 1 ♂ "Sicilia (PA) Terrasini X.1999 leg. F. Vitale/ PP1043" (CISP), 1 ♂ "Sicilia (PA) Terrasini X.1999 leg. F. Vitale/ PP1044" (CISP), 1 ♂ "Sicilia (PA) Terrasini X.1999 leg. F. Vitale/ PP1045" (CISP), 1 ♂ "Sicilia (PA) Terrasini X.1999 leg. F. Vitale/ PP1046" (CISP), 1 ♂ "Sicilia Cefalù (PA) 3.X.1999 leg. I. Sparacio/ PP1047" (CISP), 1 ♂ "Sicilia (PA) Cefalù 27.X.2003 leg. I. Sparacio/ PP1048" (CISP), 1 ♂ "Sicilia (PA) Terrasini X.1999 leg. F. Vitale/ PP1049" (CISP), 1 ♂ "Sicilia (PA) Terrasini X.1999 leg. F. Vitale/ PP1050" (CISP), 1 ♂ "Sicilia (PA) Terrasini X.1999 leg. F. Vitale/ PP1051" (CISP).
Incorrect locality label: 1 ♂ “Syria Reitter/ Pachypus caesus Er./ Pachypus project Bazzato et al. PP0001” (ZFMK).

***Pachypus candidae* Petagna, 1787**

Additional material examined. (identification based on IUMG)**:** 1 ♂ "DNA voucher BMNH 837893/ Policoro (alla luce)(MT), 20.vii.1980, leg. P. Crovato" (ZFMK), 1 ♂ "DNA voucher BMNH 837894/ Policoro (MT) in volo, 7.8.95, leg. F. Izzillo" (ZFMK), 1 ♂ "DA4405 Italy, Basilicata, Policoro, 13.viii.2013, Cirelli leg." (ZFMK), 1 ♂ "DA4406 Italy, Basilicata, Policoro, 13.viii.2013, Cirelli leg." (ZFMK), 1 ♂ "DA4407 Italy, Basilicata, Policoro, 13.viii.2013, Cirelli leg." (ZFMK), 1 ♂ "Pachypus candidae / Puglie (TA) Ginosa 28 VII 84 leg. Callegari/ PP0067" (MSNV), 1 ♂ "Pachypus candidae / Puglie (TA) Ginosa 28 VII 84 leg. Callegari/ PP0068" (MSNV), 1 ♂ "Pachypus candidae / Puglie (TA) Ginosa 28 VII 84 leg. Callegari/ PP0069" (MSNV), 1 ♂ "Pachypus candidae / Puglie (TA) Ginosa 28 VII 84 leg. Callegari/ PP0070" (MSNV), 1 ♂ "Pachypus candidae / Puglie (TA) Ginosa 28 VII 84 leg. Callegari/ PP0071" (MSNV), 1 ♂ "Pachypus candidae / Puglie (TA) Ginosa 28 VII 84 leg. Callegari/ PP0072" (MSNV), 1 ♂ "Pachypus candidae / Puglie (TA) Ginosa 28 VII 84 leg. Callegari/ PP0073" (MSNV), 1 ♂ "Pachypus candidae / Puglie (TA) Ginosa 28 VII 84 leg. Callegari/ PP0074" (MSNV), 1 ♂ "Pachypus candidae / Puglie (TA) Ginosa 28 VII 84 leg. Callegari/ PP0075" (MSNV), 1 ♂ "Pachypus candidae / Puglie (TA) Ginosa 28 VII 84 leg. Callegari/ PP0076" (MSNV), 1 ♂ "Pachypus candidae / Puglie (TA) Ginosa 28 VII 84 leg. Callegari/ PP0077" (MSNV), 1 ♂ "Pachypus candidae / Puglie (TA) Ginosa 28 VII 84 leg. Callegari/ PP0078" (MSNV), 1 ♂ "Puglie (TA) Ginosa 28 VII 84 leg. Callegari / PP0272" (MSNV), 1 ♂ "Puglie (TA) Ginosa 28 VII 84 leg. Callegari / PP0273" (MSNV), 1 ♂ "Puglie (TA) Ginosa 28 VII 84 leg. Callegari / PP0274" (MSNV), 1 ♂ "Puglie (TA) Ginosa 28 VII 84 leg. Callegari / PP0275" (MSNV), 1 ♂ "Puglie (TA) Ginosa 28 VII 84 leg. Callegari / PP0276" (MSNV), 1 ♂ "Calabria (Crotone) Isola Capo Rizzuto - Capo Piccolo VIII.'03 leg. S. Beretta / PP0294" (CMUC), 1 ♂ "Calabria (Crotone) Isola Capo Rizzuto - Capo Piccolo VIII.'03 leg. S. Beretta / PP0295" (CMUC), 1 ♂ "Calabria (Crotone) Isola Capo Rizzuto - Capo Piccolo VIII.'03 leg. S. Beretta / PP0296" (CMUC), 1 ♂ "Calabria Santa Severina (KR) 350 m 5.VIII.'03 leg. S. Beretta / PP0297" (CMUC), 1 ♂ "Basilicata (MT) Scanzano Jonico, Lido di III madonna, 28-VI-2005 leg. I. e G. Zappi / PP0298" (CMUC), 1 ♂ "Italia Basilicata (MT) Metaponto 7-10 VII 2016 40.3710,

16.8439 leg. V. Gallerati / PP0299" (CMUC), 1 ♂ "Italia Basilicata (MT) Metaponto 7-10 VII 2016 40.3710, 16.8439 leg. V. Gallerati / PP0300" (CMUC), 1 ♂ "Italia Basilicata (MT) Metaponto 7-10 VII 2016 40.3710, 16.8439 leg. V. Gallerati / PP0301" (CMUC), 1 ♂ "Italia Basilicata (MT) Metaponto 7-10 VII 2016 40.3710, 16.8439 leg. V. Gallerati / PP0302" (CMUC), 1 ♂ "Italia Puglia Castellaneta Marina 25.VII.1991 leg. Perugia / PP0303" (CMUC), 1 ♂ "Italia Puglia Castellaneta Marina 25.VII.1991 leg. Perugia / PP0304" (CMUC), 1 ♂ "Italia - Basilicata (PT) Lido di Scanzano - 3.VI.1987 P. Scaramozzino leg./ PP0815" (CJMH), 1 ♂ "IT - Basilicata - (MT) Scanzano Ionico, Lido terzo Madonna, 28.VI.2005 leg. I. e G. Zappi/ PP0895" (CGSG), 1 ♂ "IT - Basilicata - (MT) Scanzano Ionico, Lido terzo Madonna, 28.VI.2005 leg. I. e G. Zappi/ PP0896" (CGSG), 1 ♂ "IT - Basilicata - (MT) Policoro, Lido di Torremozza 30.VI.2005 leg. I. e G. Zappi/ PP0897" (CGSG), 1 ♂ "IT - Puglie - (TA) Ginosa, VII.1963 leg. Callegari/ PP0898" (CGSG), 1 ♂ "Basilicata (MT) Policoro alla luce 20.VII.1990 leg. P. Crovato/ PP1066" (CISP), 1 ♂ "Basilicata (MT) Policoro alla luce 20.VII.1990 leg. P. Crovato/ PP1067" (CISP), 1 ♂ "Basilicata (MT) Policoro alla luce 20.VII.1990 leg. P. Crovato/ PP1068" (CISP), 1 ♂ "Basilicata (MT) Policoro alla luce 20.VII.1990 leg. P. Crovato/ PP1069" (CISP), 1 ♂ "Basilicata (MT) Policoro alla luce 20.VII.1990 leg. P. Crovato/ PP1070" (CISP), 1 ♂ "Basilicata (MT) Policoro 5.VII.1989 leg. P. Crovato/ PP1071" (CISP), 1 ♂ "Basilicata (MT) Policoro 5.VII.1989 leg. P. Crovato/ PP1072" (CISP), 1 ♂ "Litorale ionico Marina Schiavonia 15-7-67 leg. Bonometto / ex. coll. L. Bonometto/ PP1113" (MSNV).

***Pachypus demoflysi* Normand, 1936**

Additional material examined. (identification based on IUMG)**:** 1 ♂ "X-DA3995 Tunisia Gov. Beja Cap Serrat, 50km E. Tabarka 17.VI.2013 G. Sabatinelli" (ZFMK), 1 ♂ "Aïn - Draham Schaedelin / ex Musæo Lefevre 1894 / 9/57, ex coll. R. Oberthür / PP0030" (ZFMK), 1 ♂ "Pachypus demoflysi Normand, 1936 det. G. Sabatinelli, 2015 / Tunisia, Gov. Bizerte Cap Serrat, 37°12' N 9°14' E 17.VI.2013, leg. G. Sabatinelli/ PP0821" (CGSG), 1 ♂ "Tunisia, Gov. Bizerte Cap Serrat, 37°12' N 9°14' E 23.VI.2014, leg. G. Sabatinelli/ PP0822" (CGSG), 1 ♂ "Tunisia, Gov. Bizerte Cap Serrat, 37°12' N 9°14' E 10-11.VI.2015, leg. G. Sabatinelli/ PP0823" (CGSG), 1 ♂ "Tunisia, Gov. Bizerte Cap Serrat, 37°12' N 9°14' E 21.VI.2013, leg. G. Sabatinelli/ PP0824" (CGSG), 1 ♂ "Tunisia, Gov. Bizerte Cap Serrat, 37°12' N 9°14' E 17.VI.2013, leg. G. Sabatinelli/ PP0825" (CGSG), 1 ♂ "Tunisia, Gov. Bizerte Cap Serrat, 37°12' N 9°14' E 10-11.VI.2015, leg. G. Sabatinelli/ PP0826" (CGSG), 1 ♂ "Pachypus demoflysi Normand, 1936 det. G. Sabatinelli, 2015 / Tunisia, Gov. Bizerte Cap Serrat, 37°12' N 9°14' E 17.VI.2013, leg. G. Sabatinelli/ PP0827" (CGSG), 1 ♂ "Pachypus demoflysi Normand, 1936 det. G. Sabatinelli, 2015 / Tunisia, Gov. Bizerte Cap Serrat, 37°12' N 9°14' E 17.VI.2013, leg. G. Sabatinelli/ PP0828" (CGSG), 1 ♂ "Pachypus demoflysi Normand, 1936 det. G. Sabatinelli, 2015 / Tunisia, Gov. Bizerte Cap Serrat, 37°12' N 9°14' E 10-11.VI.2015, leg. G. Sabatinelli/ PP0829" (CGSG), 1 ♂ "Pachypus demoflysi Normand, 1936 det. G. Sabatinelli, 2015 / Tunisia, Gov. Bizerte Cap Serrat, 37°12' N 9°14' E 10-11.VI.2015, leg. G. Sabatinelli/ PP0830" (CGSG), 1 ♂ "Pachypus demoflysi Normand, 1936 det. G. Sabatinelli, 2015 / Tunisia, Gov. Bizerte Cap Serrat, 37°12' N 9°14' E 23.VI.2014, leg. G.

Sabatinelli/ PP0831" (CGSG), 1 ♂ "Pachypus demoflysi Normand, 1936 det. G. Sabatinelli, 2015 / Tunisia, Gov. Bizerte Cap Serrat, 37°12' N 9°14' E 10-11.VI.2015, leg. G. Sabatinelli/ PP0832" (CGSG), 1 ♂ "Tunisia, Gov. Bizerte Cap Serrat, 37°12' N 9°14' E 23.VI.2014, leg. G. Sabatinelli/ PP0833" (CGSG), 1 ♂ "Pachypus demoflysi Normand, 1936 det. G. Sabatinelli, 2015 / Tunisia, Gov. Bizerte Cap Serrat, 37°12' N 9°14' E 10-11.VI.2015, leg. G. Sabatinelli/ PP0834" (CGSG), 1 ♂ "Pachypus demoflysi Normand, 1936 det. G. Sabatinelli, 2015 / Tunisia, Gov. Bizerte Cap Serrat, 37°12' N 9°14' E 10-11.VI.2015, leg. G. Sabatinelli/ PP0835" (CGSG), 1 ♂ "Tunisia, Gov. Bizerte Cap Serrat, 37°12' N 9°14' E 23.VI.2014, leg. G. Sabatinelli/ PP0836" (CGSG), 1 ♂ "Tunisia, Gov. Bizerte Cap Serrat, 37°12' N 9°14' E 23.VI.2014, leg. G. Sabatinelli/ PP0837" (CGSG), 1 ♂ "Tunisia, Gov. Bizerte Cap Serrat, 37°12' N 9°14' E 23.VI.2014, leg. G. Sabatinelli/ PP0838" (CGSG), 1 ♂ "Tunisia, Gov. Bizerte Cap Serrat, 37°12' N 9°14' E 10-11.VI.2015, leg. G. Sabatinelli/ PP0839" (CGSG), 1 ♂ "Pachypus demoflysi Normand, 1936 det. G. Sabatinelli, 2015 / Tunisia, Gov. Bizerte Cap Serrat, 37°12' N 9°14' E 23.VI.2014, leg. G. Sabatinelli/ PP0840" (CGSG), 1 ♂ "Tunisia, Gov. Bizerte Cap Serrat, 37°12' N 9°14' E 10-11.VI.2015, leg. G. Sabatinelli/ PP0841" (CGSG), 1 ♂ "Tunisia, Gov. Bizerte Cap Serrat, 37°12' N 9°14' E 17.VI.2013, leg. G. Sabatinelli/ PP0842" (CGSG), 1 ♂ "Tunisia, Gov. Bizerte Cap Serrat, 37°12' N 9°14' E 10-11.VI.2015, leg. G. Sabatinelli/ PP0843" (CGSG), 1 ♂ "Tunisia, Gov. Bizerte Cap Serrat, 37°12' N 9°14' E 21.VI.2013, leg. G. Sabatinelli/ PP0844" (CGSG), 1 ♂ "Tunisia, Gov. Bizerte Cap Serrat, 37°12' N 9°14' E 10-11.VI.2015, leg. G. Sabatinelli/ PP0845" (CGSG), 1 ♂ "Tunisia, Gov. Bizerte Cap Serrat, 37°12' N 9°14' E 10-11.VI.2015, leg. G. Sabatinelli/ PP0846" (CGSG), 1 ♂ "Tunisia, Gov. Bizerte Cap Serrat, 37°12' N 9°14' E 23.VI.2014, leg. G. Sabatinelli/ PP0847" (CGSG), 1 ♂ "Pachypus demoflysi Normand, 1936 det. G. Sabatinelli, 2015 / Tunisia, Gov. Bizerte Cap Serrat, 37°12' N 9°14' E 10-11.VI.2015, leg. G. Sabatinelli/ PP0848" (CGSG), 1 ♂ "Pachypus demoflysi Normand, 1936 det. G. Sabatinelli, 2015 / GSPA20 / Tunisia, Gov. Bizerte Cap Serrat, 37°12' N 9°14' E 17.VI.2013, leg. G. Sabatinelli/ PP0849" (CGSG), 1 ♂ "Pachypus demoflysi Normand, 1936 det. G. Sabatinelli, 2015 / Tunisia, Gov. Bizerte Cap Serrat, 37°12' N 9°14' E 17.VI.2013, leg. G. Sabatinelli/ PP0850" (CGSG), 1 ♂ "Pachypus demoflysi Normand, 1936 det. G. Sabatinelli, 2015 / Tunisia, Gov. Bizerte Cap Serrat, 37°12' N 9°14' E 2.VII.2014, leg. G. Sabatinelli/ PP0851" (CGSG), 1 ♂ "Pachypus demoflysi Normand, 1936 det. G. Sabatinelli, 2015 / Tunisia, Gov. Bizerte Cap Serrat, 37°12' N 9°14' E 17.VI.2013, leg. G. Sabatinelli/ PP0852" (CGSG), 1 ♂ "Pachypus demoflysi Normand, 1936 det. G. Sabatinelli, 2015 / Tunisia, Gov. Bizerte Cap Serrat, 37°12' N 9°14' E 10-11.VI.2015, leg. G. Sabatinelli/ PP0853" (CGSG), 1 ♂ "Tunisia, Gov. Bizerte Cap Serrat, 37°12' N 9°14' E 10-11.VI.2015, leg. G. Sabatinelli/ PP0854" (CGSG), 1 ♂ "Tunisia, Gov. Bizerte Cap Serrat, 37°12' N 9°14' E 17.VI.2013, leg. G. Sabatinelli/ PP0855" (CGSG), 1 ♂ "Tunisia, Gov. Bizerte Cap Serrat, 37°12' N 9°14' E 17.VI.2013, leg. G. Sabatinelli/ PP0856" (CGSG), 1 ♂ "Tunisia, Gov. Bizerte Cap Serrat, 37°12' N 9°14' E 17.VI.2013, leg. G. Sabatinelli/ PP0857" (CGSG), 1 ♂ "Tunisia, Gov. Bizerte Cap Serrat, 37°12' N 9°14' E 10-11.VI.2015, leg. G. Sabatinelli/ PP0858" (CGSG), 1 ♂ "Tunisia, Gov. Bizerte Cap Serrat, 37°12' N 9°14' E 17.VI.2013, leg. G. Sabatinelli/ PP0859" (CGSG), 1 ♂ "Pachypus candidae / Alg. / Coll. R. I. Sc. N. B. Algerie / R. Mus. Hist. Nat. Belg. I. G. 12.595/ PP0957" (ISNB), 1 ♂ "candidae = cornutus / Gerageles Algerie / Coll. R. I. Sc. N. B. Algerie / Détermin. E. Branake/ PP0958" (ISNB).

***Pachypus excavatus* Fabricius, 1792**

Additional material examined (identification based on RADSeq data, unpublished data)**.** 1 ♂ "X-DA4605 Italy Salerno: Palinuro 1.VI.2017 M. D'Amato" (ZFMK).
Additional material examined. (identification based on IUMG)**:** 1 ♂ "DNA voucher BMNH 836902/ Italia: Lazio: Castel di Guido, vi.2000, leg. D. Ahrens" (ZFMK), 1 ♂ "DNA voucher BMNH 837882/ Italia: Lazio: Castel di Guido, vi.2000, leg. D. Ahrens" (ZFMK), 1 ♂ "X-DA4604 Italy Lazio, Torre Astura 6.xiii.2012 D. Ahrens" (ZFMK), 1 ♂ "X-DA4606 Italy Lazio, Torre Astura 20.VII.2016 D. Ahrens & S. Fabrizi" (ZFMK), 1 ♂ "X-DA4606 Italy Lazio, Torre Astura 20.VII.2016 D. Ahrens & S. Fabrizi" (ZFMK), 1 ♂ "X-DA4644 Italy Lazio: Sughereta S. Vito 10.VIII.2013 G. Carpaneto" (ZFMK), 1 ♂ "X-DA4645 Italy Lazio: Sughereta S. Vito 9.VIII.2013 G. Carpaneto" (ZFMK), 1 ♂ "X-DA4646 Italy Lazio: Sughereta S. Vito 10.VIII.2013 G. Carpaneto" (ZFMK), 1 ♂ "X-DA4647 Italy Lazio: Sughereta S. Vito 11.VIII.2013 G. Carpaneto" (ZFMK), 1 ♂ "X-DA4648 Italy Lazio: Sughereta S. Vito 10.VIII.2013 G. Carpaneto" (ZFMK), 1 ♂ "X-DA4649 Italy Lazio: Sughereta di Pomezia 26.VI.2013 F. Turchetti" (ZFMK), 1 ♂ "X-DA4650 Italy Lazio: Sughereta di Pomezia 26.VI.2013 F. Turchetti" (ZFMK), 1 ♂ "Lazio RM Castel Fusano Loc. Piscina torta / 07.2017 Patacchiola/ PP0102" (CCAU), 1 ♂ "Lazio RM Castel Fusano Loc. Piscina torta / 27.07.2017 Patacchiola/ PP0103" (CCAU), 1 ♂ "Lazio RM Castel Fusano Loc. Piscina torta / 27.07.2017 Patacchiola/ PP0104" (CCAU), 1 ♂ "Lazio RM Castel Fusano Loc. Piscina torta / 27.07.2017 Patacchiola/ PP0105" (CCAU), 1 ♂ "Lazio Lido di Maccavese / 14.07.2015 coll. Patacchiola/ PP0106" (CCAU), 1 ♂ "Lazio Castel Fusano Piscina torta / 29.07.2014 coll. Patacchiola/ PP0107" (CCAU), 1 ♂ "Lazio Castel Fusano Piscina torta / 29.07.2014 coll. Patacchiola/ PP0108" (CCAU), 1 ♂ "Lazio Castel Fusano Piscina torta / 29.07.2014 coll. Patacchiola/ PP0109" (CCAU), 1 ♂ "Lazio RM ten. di Castel Fusano Loc. Piscina torta / 29.06.2014 coll. Patacchiola/ PP0110" (CCAU), 1 ♂ "Pachypus candidae (Petagna, 1786) Det. Uliana, 2015 / Lazio Sperlonga VII.67 Clementi / PP0305" (CMUC), 1 ♂ "Lazio - Roma Maccarese 10.VII.1985 lg. M. Gigli/ PP0475" (CDCC), 1 ♂ "Lazio - Roma Ostia 13-VII.1987 leg. M. Gigli/ PP0476" (CDCC), 1 ♂ "P. excavatus / Lazio - RO Maccarese VII.2012 leg. Patacchiola/ PP0477" (CDCC), 1 ♂ "Lazio Lido Maccavese / 14.07.2015 coll. Patacchiola/ PP0478" (CDCC), 1 ♂ "Lazio Lido Maccavese / 14.07.2015 coll. Patacchiola/ PP0479" (CDCC), 1 ♂ "Lazio Castel Fusano Piscina Torta / 29.07.2015 coll. Patacchiola/ PP0480" (CDCC), 1 ♂ "Lazio Castel di Guido loc. Maccavese / 14.07.2015 coll. Patacchiola/ PP0481" (CDCC), 1 ♂ "Lazio Castel di Guido loc. Maccavese / 14.07.2015 coll. Patacchiola/ PP0482" (CDCC), 1 ♂ "Lazio Castel di Guido loc. Maccavese / 14.07.2015 coll. Patacchiola/ PP0483" (CDCC), 1 ♂ "Lazio Castel di Guido tenuto Loc. [1 ♂ Lat] 6.07.2015 Patacchiola coll. Patacchiola/ PP0488" (CDCC), 1 ♂ "Lazio Castel Fusano Piscina Torta / 29.07.2015 coll. Patacchiola/ PP0489" (CDCC), 1 ♂ "Italia, Lazio RM ten. di Castel Fusano Loc. Piscina torta 07-2018 Patacchiola D/ PP0816" (CJMH), 1 ♂ "IT - Lazio - (LT) Sperlonga, 14.VI.1972, leg. G. Gobbi/ PP0879" (CGSG), 1 ♂ "IT - Lazio - (LT) Sperlonga, 14.VI.1972, leg. G. Gobbi/ PP0880" (CGSG), 1 ♂ "IT - Lazio - (LT) Sperlonga, 14.VI.1972, leg. G. Gobbi/ PP0881" (CGSG), 1 ♂ "IT - Lazio - (LT) Sperlonga, 14.VI.1972, leg. G. Gobbi/ PP0882" (CGSG), 1 ♂ "IT - Lazio - (LT) Sperlonga, 14.VI.1972, leg. G. Gobbi/ PP0883" (CGSG), 1 ♂ "IT -

Lazio - (LT) Sperlonga, 14.VI.1972, leg. G. Gobbi/ PP0884" (CGSG), 1 ♂ "IT - Lazio - (RM) dint. cimitero Maccarese, 30.VI.2018, leg. E. Pacieri/ PP0888" (CGSG), 1 ♂ "IT - Lazio - (RM) dint. cimitero Maccarese, 30.VI.2018, leg. E. Pacieri/ PP0889" (CGSG), 1 ♂ "IT - Lazio - (RM) dint. cimitero Maccarese, 30.VI.2018, leg. E. Pacieri/ PP0890" (CGSG), 1 ♂ "Pachypus cand. v. Erichsoni / Maccarese 10.VII.910 Luigioni - Lazio / Coll. R. I. Sc. N. B. Italie/ PP0941" (ISNB), 1 ♂ "P. candidae a. caesicolor determ. Luigioni m. / caesicolor / Lazio Maccarese Luig. / Coll. Petrovitz/ PP1007" (MHNG), 1 ♂ "Maccarese 10.VII.910 Luigioni - Lazio / Coll. Petrovitz/ PP1012" (MHNG), 1 ♂ "P. candidae determ. Luigioni / Lazio Maccarese Luig. / Coll. Petrovitz/ PP1015" (MHNG), 1 ♂ "Pachypus caesus Er. det. Petrovitz / Maccarese 10.VII.910 Luigioni - Lazio / Coll. Petrovitz/ PP1021" (MHNG), 1 ♂ "Pachypus candidae Pet. / S. Felice Circeo (LT) 1.10/07/1989 leg. E. Ratti/ PP1079" (MSNV), 1 ♂ "S. Felice Circeo (LT) 1.10/07/1989 leg. E. Ratti/ PP1080" (MSNV), 1 ♂ "S. Felice Circeo (LT) 1.10/07/1989 leg. E. Ratti/ PP1081" (MSNV), 1 ♂ "S. Felice Circeo (LT) 1.10/07/1989 leg. E. Ratti/ PP1082" (MSNV), 1 ♂ "S. Felice Circeo (LT) 1.10/07/1989 leg. E. Ratti/ PP1083" (MSNV), 1 ♂ "S. Felice Circeo (LT) 1.10/07/1989 leg. E. Ratti/ PP1091" (MSNV), 1 ♂ "S. Felice Circeo (LT) 1.10/07/1989 leg. E. Ratti/ PP1092" (MSNV), 1 ♂ "S. Felice Circeo (LT) 1.10/07/1989 leg. E. Ratti/ PP1093" (MSNV), 1 ♂ "P. candidae a. caesicolor determ. Luigioni m. / Lazio Maccarese Luig. 11.VII.99/ PP1118" (CISP), 1 ♂ "Maccarese 10.VII.910 Luigioni - Lazio/ PP1119" (CISP), 1 ♂ "Pachypus caesus / Maccarese 10.VII.910 Luigioni - Lazio/ PP1120" (CISP), 1 ♂ "Maccarese 10.VII.910 Luigioni - Lazio/ PP1121" (CISP), 1 ♂ "Roma Acilia Castel. VII.935/ PP1122" (CISP), 1 ♂ "Maccarese Luig. VI.927/ PP1123" (CISP), 1 ♂ "Maccarese 10.VII.910 Luigioni - Lazio/ PP1124" (CISP), 1 ♂ "Maccarese 10.VII.910 Luigioni - Lazio/ PP1125" (CISP), 1 ♂ "Maccarese 10.VII.910 Luigioni - Lazio/ PP1126" (CISP), 1 ♂ "Maccarese 10.VII.910 Luigioni - Lazio/ PP1127" (CISP), 1 ♂ "DNA voucher BMNH 837891/ Palinuro - SA, Foce Mingardo, 28.7.1980, leg. Bonometto" (ZFMK), 1 ♂ "DNA voucher BMNH 837892/ Foce Mingardo Rdo Palinuro - SA, 30.7.1980, leg. Bonometto" (ZFMK), 1 ♂ "PP1099/ Palinuro - SA Foce Mingardo 20.7.1980 Bonometto leg." (MSNV), 1 ♂ "PP1128/ Palinuro (SA) 1.8.1980 leg. Bonometto" (MSNV), 1 ♂ "PP1129/ Palinuro - SA Foce Mingardo 28.7.1980 Bonometto leg." (MSNV), 1 ♂ "PP1130/ Palinuro (SA) 1.8.1980 leg. Bonometto" (MSNV), 1 ♂ "PP1131/ Foci Mingardo Palinuro SA 30.7.1980 Bonometto leg." (MSNV), 1 ♂ "PP1132/ Palinuro - SA Foce Mingardo 25.7.1980 leg. Bonometto" (MSNV).

***Pachypus impressus* Erichson, 1840**

Type material examined. (identification based on IUMG**):** Paratypes (*P. melonii*): 1 ♂ "Paratypus Pachypus melonii Sparacio, 2008 / Italia - Sardegna Assemini - CA 21.6.07 leg. D. Cillo/ PP0728" (CDCC), 1 ♂ "Paratypus Pachypus melonii Sparacio, 2008 / Italia - Sardegna Assemini - CA 21.6.07 leg. D. Cillo/ PP0729" (CDCC), 1 ♂ "Paratypus Pachypus melonii Sparacio, 2008 / Italia - Sardegna Assemini - CA 21.6.07 leg. D. Cillo/ PP0730" (CDCC), 1 ♂ "Paratypus Pachypus melonii Sparacio, 2008 / Italia - Sardegna Assemini - CA 21.6.07 leg. D. Cillo/ PP0731" (CDCC), 1 ♂ "Paratypus Pachypus melonii Sparacio, 2008 / Italia - Sardegna Assemini - CA 21.6.07 leg. D. Cillo/ PP0732" (CDCC), 1 ♂ "Paratypus Pachypus melonii Sparacio, 2008 / Italia - Sardegna Assemini - CA 21.6.07 leg. D. Cillo/ PP0733" (CDCC), 1 ♂ "Paratypus Pachypus melonii Sparacio,

2008 / Italia - Sardegna Assemini - CA 21.6.07 leg. D. Cillo/ PP0734" (CDCC), 1 ♂ "Paratypus Pachypus melonii Sparacio, 2008 / Italia - Sardegna Assemini - CA 21.6.07 leg. D. Cillo/ PP0735" (CDCC), 1 ♂ "Paratypus Pachypus melonii Sparacio, 2008 / Italia - Sardegna Assemini - CA 21.6.07 leg. D. Cillo/ PP0736" (CDCC), 1 ♂ "Paratypus Pachypus melonii Sparacio, 2008 / Italia - Sardegna Assemini - CA 21.6.07 leg. D. Cillo/ PP0737" (CDCC), 1 ♂ "Paratypus Pachypus melonii Sparacio, 2008 / Italia - Sardegna Assemini - CA 29.6.07 leg. D. Cillo/ PP0738" (CDCC), 1 ♂ "Paratypus Pachypus melonii Sparacio, 2008 / Italia - Sardegna Assemini - CA 16.6.07 leg. D. Cillo/ PP0739" (CDCC), 1 ♂ "Paratypus Pachypus melonii Sparacio, 2008 / Italia - Sardegna Assemini - CA 16.6.07 leg. D. Cillo/ PP0740" (CDCC), 1 ♂ "Paratypus Pachypus melonii Sparacio, 2008 / Italia - Sardegna Assemini - CA 16.6.07 leg. D. Cillo/ PP0741" (CDCC), 1 ♂ "Paratypus Pachypus melonii Sparacio, 2008 / Italia - Sardegna Assemini - CA 16.6.07 leg. D. Cillo/ PP0742" (CDCC), 1 ♂ "Paratypus Pachypus melonii Sparacio, 2008 / Italia - Sardegna Assemini - CA 16.6.07 leg. D. Cillo/ PP0743" (CDCC), 1 ♂ "Paratypus Pachypus melonii Sparacio, 2008 / Italia - Sardegna Assemini - CA 16.6.07 leg. D. Cillo/ PP0744" (CDCC), 1 ♂ "Paratypus Pachypus melonii Sparacio, 2008 / Italia - Sardegna Assemini - CA 16.6.07 leg. D. Cillo/ PP0745" (CDCC), 1 ♂ "Paratypus Pachypus melonii Sparacio, 2008 / Italia - Sardegna Assemini - CA 16.6.07 leg. D. Cillo/ PP0746" (CDCC), 1 ♂ "Paratypus Pachypus melonii Sparacio, 2008 / Sardegna (CA) Assemini, Rio Flumini Mannu 30.VI.1996 C. Meloni/ PP1053" (CISP), 1 ♂ "Paratypus Pachypus melonii Sparacio, 2008 / Sardegna (CA) Assemini Rio Flumini Mannu 30.6.1996 C. Meloni/ PP1054" (CISP), 1 ♂ "Paratypus Pachypus melonii Sparacio, 2008 / Sardegna (CA) Assemini Rio Flumini Mannu 30.VI.1996 legit C. Meloni/ PP1055" (CISP), 1 ♂ "Paratypus Pachypus melonii Sparacio, 2008 / Sardegna (CA) Villaspeciosa dintorni / 23.VI.1976 legit Meloni C./ PP1056" (CISP), 1 ♂ "Paratypus Pachypus melonii Sparacio, 2008 / Sardegna (CA) Villaspeciosa dintorni / 23.VI.1976 legit Meloni C./ PP1057" (CISP), 1 ♂ "Paratypus Pachypus melonii Sparacio, 2008 / Sardegna (CA) Villaspeciosa loc. Fraighèddas / 23.VI.1976 m 30-40 s.l.m. leg. C. Meloni/ PP1058" (CISP), 1 ♂ "Paratypus Pachypus melonii Sparacio, 2008 / Sardegna (CA) Assemini Rio Cixerri 8.VI.1996 leg. D. Sechi/ PP1059" (CISP), 1 ♂ "Paratypus Pachypus melonii Sparacio, 2008 / Sardegna (CA) Assemini Rio Cixerri 8.VI.1996 leg. D. Sechi/ PP1060" (CISP), 1 ♂ "Paratypus Pachypus melonii Sparacio, 2008 / Sardegna (CA) Assemini Rio Cixerri 8.VI.1996 leg. D. Sechi/ PP1061" (CISP), 1 ♂ "Paratypus Pachypus melonii Sparacio, 2008 / Sardegna (CA) Assemini Rio Cixerri 8.VI.1996 leg. D. Sechi/ PP1062" (CISP), 1 ♂ "Paratypus Pachypus melonii Sparacio, 2008 / Sardegna (CA) Assemini Rio Cixerri 8.VI.1996 leg. D. Sechi/ PP1063" (CISP), 1 ♂ "Paratypus Pachypus melonii Sparacio, 2008 / Sardegna (CA) Assemini Rio Cixerri 8.VI.1996 leg. D. Sechi/ PP1064" (CISP), 1 ♂ "Paratypus Pachypus melonii Sparacio, 2008 / Sardegna - (CA) Assémini - Flúmini Mannu / 30.VI.1996 legit Meloni C. / / PP1065" (CISP), 1 ♂ "Paratypus Pachypus melonii Sparacio, 2008 / Italia - Sardegna Assemini - CA 21.6.2007 leg. D. Cillo/ PP0465" (CDCC).

Additional material examined. (identification based on IUMG**):** 1 ♂ "Pachypus melonii / Sardegna Assemini VI.2015 leg. D. Cillo/ PP0093" (CEBQ), 1 ♂ "Pachypus melonii / Sardegna Assemini - CA 6.2013 leg. E. Bazzato/ PP0094" (CEBQ), 1 ♂ "Pachypus melonii / Sardegna Assemini - CA VI.2013 D. Cillo leg./ PP0095" (CEBQ), 1 ♂ "Pachypus melonii / Italy - Sardegna Assemini (Cagliari) 23.VI.2013 leg. D. Cillo/

PP0096" (CEBQ), 1 ♂ "Pachypus melonii 1 ♂ Decimomannu (CA), Riu Flumini Mannu, 30.VI.2017 su veget. erbacea/ PP0141" (CCAU), 1 ♂ "Pachypus melonii 1 ♂ Decimomannu (CA), Riu Flumini Mannu, 30.VI.2017 su veget. erbacea/ PP0142" (CCAU), 1 ♂ "Pachypus melonii 1 ♂ Decimomannu (CA), Riu Flumini Mannu, 30.VI.2017 su vegetazione erbacea/ PP0143" (CCAU), 1 ♂ "Pachypus meloni 1 ♂ Decimomannu-Assemini (CA), Riu Flumini Mannu, 01.VII.2013 leg. Ancona su vegetazione/ PP0144" (CCAU), 1 ♂ "Pachypus meloni 1 ♂ Decimomannu-Assemini (CA), Riu Flumini Mannu, 01.VII.2013 leg. Ancona su vegetazione/ PP0145" (CCAU), 1 ♂ "Pachypus meloni 1 ♂ Decimomannu-Assemini (CA), Riu Flumini Mannu, 01.VII.2013 leg. Ancona su vegetazione/ PP0146" (CCAU), 1 ♂ "Pachypus meloni 1 ♂ Assemini (CA), Riu Flumini Mannu, 03.VII.2014/ PP0147" (CCAU), 1 ♂ "Sardegna Assemini (CA) VI.2013 D. Cillo leg. / A. Lecis/ PP0180" (CALC), 1 ♂ "Sardegna Assemini (CA) VI.2013 D. Cillo leg. / A. Lecis/ PP0181" (CALC), 1 ♂ "Assemini Cagliari 24-6-2012 leg. D. Cillo / A. Lecis/ PP0186" (CALC), 1 ♂ "Assemini CA - 24-6-12 D. Cillo / A. Lecis/ PP0187" (CALC), 1 ♂ "Italy - Sardegna Cagliari Assemini 13-6-2010 leg. D. Cillo / A. Lecis/ PP0188" (CALC), 1 ♂ "Cagliari Assemini 12-7-2014 / A. Lecis/ PP0189" (CALC), 1 ♂ "Sardinia Assemini (CA) Rio Cixerri 8.VI.1996 leg. D. Sechi/ PP0199" (CALC), 1 ♂ "Sardegna Assemini (CA) Flumini Mannu 17-VI-2015 leg. M.G. Atzori - D. Cillo/ PP0240" (CMAC), 1 ♂ "Sardegna Assemini (CA) Flumini Mannu 17-VI-2015 leg. M.G. Atzori - D. Cillo/ PP0241" (CMAC), 1 ♂ "Sardegna Assemini (CA) Flumini Mannu 17-VI-2015 leg. M.G. Atzori - D. Cillo/ PP0242" (CMAC), 1 ♂ "Sardegna Assemini (CA) Flumini Mannu 17-VI-2015 leg. M.G. Atzori - D. Cillo/ PP0243" (CMAC), 1 ♂ "Sardegna Assemini (CA) Flumini Mannu 17-VI-2015 leg. M.G. Atzori - D. Cillo/ PP0244" (CMAC), 1 ♂ "Sardegna Assemini (CA) Flumini Mannu 17-VI-2015 leg. M.G. Atzori - D. Cillo/ PP0245" (CMAC), 1 ♂ "Pachypus melonii Sparacio, 2008 / Italy - Sardinia Assemini (Cagliari) 18.VI.2009 leg. P. Leo/ PP0246" (CMAC), 1 ♂ "Italy - Sardegna Cagliari Assemini 13.6.2010 leg. D. Cillo/ PP0247" (CMAC), 1 ♂ "Sardinia Assemini (CA) 8.VI.1996 Rio Cixerri leg. D. Sechi / PP0328" (CMUC), 1 ♂ "Sardegna Assemini- VII-1939 U. Lostia / PP0329" (CMUC), 1 ♂ "Italy - Sardegna Cagliari Assemini leg. D. Cillo VI.2012/ PP0456" (CDCC), 1 ♂ "Assemini Rio Cixerri 12-VI-2009 leg. C. Meloni / PP0457" (CDCC), 1 ♂ "Italy - Sardegna Assemini (Cagliari) VI.2012 leg. D. Cillo/ PP0459" (CDCC), 1 ♂ "Italy - Sardegna Assemini (Cagliari) VI.2012 leg. D. Cillo/ PP0460" (CDCC), 1 ♂ "Italy - Sardegna Assemini (Cagliari) VI.2012 leg. D. Cillo/ PP0461" (CDCC), 1 ♂ "Italy - Sardegna Assemini (Cagliari) 26.VI.2013 leg. D. Cillo/ PP0462" (CDCC), 1 ♂ "Italy - Sardegna Assemini (Cagliari) 23.VI.2013 leg. D. Cillo/ PP0463" (CDCC), 1 ♂ "Italy - Sardegna Assemini (Cagliari) 23.VI.2013 leg. D. Cillo/ PP0464" (CDCC), 1 ♂ "Sardegna - CA Assemini VI.2012 leg. D. Cillo/ PP0466" (CDCC), 1 ♂ "Italy - Sardegna Cagliari Assemini leg. D. Cillo 16.6.2010/ PP0517" (CDCC), 1 ♂ "Italy - Sardegna Cagliari Assemini leg. D. Cillo 16.6.2010/ PP0518" (CDCC), 1 ♂ "Italy - Sardegna Cagliari Assemini leg. D. Cillo 16.6.2010/ PP0519" (CDCC), 1 ♂ "Italy - Sardegna Assemini (Cagliari) 23-VI-2013 leg. D. Cillo/ PP0520" (CDCC), 1 ♂ "Italy - Sardegna Cagliari Assemini leg. D. Cillo 15.06.2012/ PP0521" (CDCC), 1 ♂ "Italy - Sardegna Cagliari Assemini leg. D. Cillo 13.6.2010 Flumini Mannu/ PP0522" (CDCC), 1 ♂ "Italy - Sardegna Cagliari Assemini leg. D. Cillo 16.6.2010/ PP0523" (CDCC), 1 ♂ "Italy - Sardegna Cagliari Assemini leg. D. Cillo 16.6.2010/ PP0524" (CDCC), 1 ♂ "Italy - Sardegna Cagliari Assemini leg. D. Cillo 16.6.2010/

PP0525" (CDCC), 1 ♂ "Italy - Sardegna Cagliari Assemini leg. D. Cillo 16.6.2010/ PP0526" (CDCC), 1 ♂ "Italy - Sardegna Cagliari Assemini leg. D. Cillo 16.6.2010/ PP0527" (CDCC), 1 ♂ "Italy - Sardegna Cagliari Assemini leg. D. Cillo 16.6.2010/ PP0528" (CDCC), 1 ♂ "Italy - Sardegna Cagliari Assemini leg. D. Cillo 16.6.2010 Flum. Mannu/ PP0529" (CDCC), 1 ♂ "Italy - Sardegna Cagliari Assemini leg. D. Cillo 16.6.2010/ PP0530" (CDCC), 1 ♂ "Italy - Sardegna Cagliari Assemini leg. D. Cillo 16.6.2010/ PP0531" (CDCC), 1 ♂ "Italy - Sardegna Cagliari Assemini leg. D. Cillo 16.6.2010/ PP0532" (CDCC), 1 ♂ "Italy - Sardegna Cagliari Assemini leg. D. Cillo 16.6.2010/ PP0533" (CDCC), 1 ♂ "Italy - Sardegna Cagliari Assemini leg. D. Cillo 16.6.2010/ PP0534" (CDCC), 1 ♂ "Italy - Sardegna Cagliari Assemini leg. D. Cillo 13.6.2010/ PP0535" (CDCC), 1 ♂ "Italy - Sardegna Cagliari Assemini leg. D. Cillo 13.6.2010/ PP0536" (CDCC), 1 ♂ "Italy - Sardegna Cagliari Assemini leg. D. Cillo 13.6.2010/ PP0537" (CDCC), 1 ♂ "Italy - Sardegna Cagliari Assemini leg. D. Cillo 13.6.2010/ PP0538" (CDCC), 1 ♂ "Italy - Sardegna Cagliari Assemini leg. D. Cillo 13.6.2010/ PP0539" (CDCC), 1 ♂ "Italy - Sardegna Cagliari Assemini leg. D. Cillo 13.6.2010/ PP0540" (CDCC), 1 ♂ "Italy - Sardegna Cagliari Assemini leg. D. Cillo 13.6.2010/ PP0541" (CDCC), 1 ♂ "Italy - Sardegna Cagliari Assemini leg. D. Cillo 13.6.2010/ PP0542" (CDCC), 1 ♂ "Italy - Sardegna Cagliari Assemini leg. D. Cillo 13.6.2010/ PP0543" (CDCC), 1 ♂ "Italy - Sardegna Cagliari Assemini leg. D. Cillo 13.6.2010/ PP0544" (CDCC), 1 ♂ "Italy - Sardegna Cagliari Assemini leg. D. Cillo 13.6.2010/ PP0545" (CDCC), 1 ♂ "Italy - Sardegna Cagliari Assemini leg. D. Cillo 13.6.2010/ PP0546" (CDCC), 1 ♂ "Italy - Sardegna Cagliari Assemini leg. D. Cillo 13.6.2010/ PP0547" (CDCC), 1 ♂ "Italy - Sardegna Cagliari Assemini leg. D. Cillo 13.6.2010/ PP0548" (CDCC), 1 ♂ "Italy - Sardegna Cagliari Assemini leg. D. Cillo 13.6.2010/ PP0549" (CDCC), 1 ♂ "Italy - Sardegna Cagliari Assemini leg. D. Cillo 13.6.2010/ PP0550" (CDCC), 1 ♂ "Italy - Sardegna Cagliari Assemini leg. D. Cillo 13.6.2010 Flumini Mannu/ PP0551" (CDCC), 1 ♂ "Italy - Sardegna Cagliari Assemini leg. D. Cillo 13.6.2010/ PP0552" (CDCC), 1 ♂ "Italy - Sardegna Cagliari Assemini leg. D. Cillo 13.6.2010/ PP0553" (CDCC), 1 ♂ "Italy - Sardegna Cagliari Assemini leg. D. Cillo 13.6.2010/ PP0554" (CDCC), 1 ♂ "Italy - Sardegna Cagliari Assemini leg. D. Cillo 04.07.2011/ PP0558" (CDCC), 1 ♂ "Italy - Sardegna Cagliari Assemini leg. D. Cillo 04.07.2011/ PP0559" (CDCC), 1 ♂ "Italy - Sardegna Cagliari Assemini leg. D. Cillo 04.07.2011/ PP0560" (CDCC), 1 ♂ "Italy - Sardegna Cagliari Assemini leg. D. Cillo 04.07.2011/ PP0561" (CDCC), 1 ♂ "Italy - Sardegna Cagliari Assemini leg. D. Cillo 04.07.2011/ PP0562" (CDCC), 1 ♂ "Italy - Sardegna Cagliari Assemini leg. D. Cillo 04.07.2011/ PP0563" (CDCC), 1 ♂ "Italy - Sardegna Cagliari Assemini leg. D. Cillo 04.07.2011/ PP0564" (CDCC), 1 ♂ "Italy - Sardegna Cagliari Assemini leg. D. Cillo 04.07.2011/ PP0565" (CDCC), 1 ♂ "Italy - Sardegna Cagliari Assemini leg. D. Cillo 04.07.2011/ PP0566" (CDCC), 1 ♂ "Italy - Sardegna Cagliari Assemini leg. D. Cillo 04.07.2011/ PP0567" (CDCC), 1 ♂ "Italy - Sardegna Cagliari Assemini leg. D. Cillo 15.VI.2012/ PP0568" (CDCC), 1 ♂ "Italy - Sardegna Cagliari Assemini leg. D. Cillo 15.VI.2012/ PP0569" (CDCC), 1 ♂ "Italy - Sardegna Cagliari Assemini leg. D. Cillo 15.VI.2012/ PP0575" (CDCC), 1 ♂ "Italy - Sardegna Cagliari Assemini leg. D. Cillo 15.VI.2012 Flumini Mannu/ PP0576" (CDCC), 1 ♂ "Italy - Sardegna Cagliari Assemini leg. D. Cillo 15.VI.2012/ PP0577" (CDCC), 1 ♂ "Italy - Sardegna Cagliari Assemini leg. D. Cillo VI.2012/ PP0578" (CDCC), 1 ♂ "Italy - Sardegna Cagliari Assemini leg. D. Cillo

VI.2012/ PP0579" (CDCC), 1 ♂ "Italy - Sardegna Assemini (Cagliari) 26.VI.2013 leg. D. Cillo/ PP0580" (CDCC), 1 ♂ "Italy - Sardegna Assemini (Cagliari) 26.VI.2013 leg. D. Cillo/ PP0581" (CDCC), 1 ♂ "Italy - Sardegna Assemini (Cagliari) 26.VI.2013 leg. D. Cillo/ PP0582" (CDCC), 1 ♂ "Italy - Sardegna Assemini (Cagliari) 26.VI.2013 leg. D. Cillo/ PP0583" (CDCC), 1 ♂ "Italy - Sardegna Assemini (Cagliari) 26.VI.2013 leg. D. Cillo/ PP0584" (CDCC), 1 ♂ "Italy - Sardegna Assemini (Cagliari) 23.VI.2013 leg. D. Cillo/ PP0585" (CDCC), 1 ♂ "Italy - Sardegna Assemini (Cagliari) 23.VI.2013 leg. D. Cillo/ PP0586" (CDCC), 1 ♂ "Italy - Sardegna Assemini (Cagliari) 23.VI.2013 leg. D. Cillo/ PP0587" (CDCC), 1 ♂ "Italy - Sardegna Assemini (Cagliari) 26.VI.2013 leg. D. Cillo/ PP0588" (CDCC), 1 ♂ "Italy - Sardegna Assemini (Cagliari) 26.VI.2013 leg. D. Cillo/ PP0589" (CDCC), 1 ♂ "Italy - Sardegna Assemini (Cagliari) 26.VI.2013 leg. D. Cillo/ PP0590" (CDCC), 1 ♂ "Italy - Sardegna Assemini (Cagliari) 26.VI.2013 leg. D. Cillo/ PP0616" (CDCC), 1 ♂ "Italy - Sardegna Assemini (Cagliari) 23.VI.2013 leg. D. Cillo/ PP0617" (CDCC), 1 ♂ "Italy - Sardegna Cagliari Assemini leg. D. Cillo 15.VI.2012/ PP0618" (CDCC), 1 ♂ "Italy - Sardegna Assemini (Cagliari) 23.VI.2013 leg. D. Cillo/ PP0619" (CDCC), 1 ♂ "Italy - Sardegna Cagliari Assemini leg. D. Cillo 04.07.2011/ PP0622" (CDCC), 1 ♂ "Italy - Sardegna Cagliari Assemini leg. D. Cillo 13.6.2010/ PP0623" (CDCC), 1 ♂ "IT - Sardegna - (CA) Cagliari, Poetto 16.VI.1969, leg. Stefani/ PP0869" (CGSG), 1 ♂ "Cagliari (dintorni) loc. Poetto 16.VI.1960 R. Stefani/ PP0870" (CGSG).

Pachypus sulcis **sp. n.**

Type material examined. (ID based on IUMG): 1 ♂ "X-DA3424b Italy Sardegna: Torre Chia (Camping) 38°53'57.5''N, 08°53'10.0''E 28.6.-1.7.2012 D. Ahrens & S. Fabrizi" (ZFMK), 1 ♂ "X-DA3425a Italy Sardegna: Torre Chia (Camping) 38°53'57.5''N, 08°53'10.0''E 28.vi.-1.vii.2012 D. Ahrens & S. Fabrizi" (ZFMK), 1 ♂ "X-DA3425b Italy Sardegna: Torre Chia (Camping) 38°53'57.5''N, 08°53'10.0''E 28.vi.-1.vii.2012 D. Ahrens & S. Fabrizi" (ZFMK), 1 ♂ "X-DA3425c Italy Sardegna: Torre Chia (Camping) 38°53'57.5''N, 08°53'10.0''E 28.vi.-1.vii.2012 D. Ahrens & S. Fabrizi" (ZFMK), 1 ♂ "X-DA3425d Italy Sardegna: Torre Chia (Camping) 38°53'57.5''N, 08°53'10.0''E 28.vi.-1.vii.2012 D. Ahrens & S. Fabrizi" (ZFMK), 1 ♂ "X-DA3425e Italy Sardegna: Torre Chia (Camping) 38°53'57.5''N, 08°53'10.0''E 28.vi.-1.vii.2012 D. Ahrens & S. Fabrizi" (ZFMK), 1 ♂ "X-DA3425f Italy Sardegna: Torre Chia (Camping) 38°53'57.5''N, 08°53'10.0''E 28.vi.-1.vii.2012 D. Ahrens & S. Fabrizi" (ZFMK), 1 ♂ "X-DA3425g Italy Sardegna: Torre Chia (Camping) 38°53'57.5''N, 08°53'10.0''E 28.vi.-1.vii.2012 D. Ahrens & S. Fabrizi" (ZFMK), 1 ♂ "X-DA3425h Italy Sardegna: Torre Chia (Camping) 38°53'57.5''N, 08°53'10.0''E 28.vi.-1.vii.2012 D. Ahrens & S. Fabrizi" (ZFMK), 1 ♂ "X-DA3425i Italy Sardegna: Torre Chia (Camping) 38°53'57.5''N, 08°53'10.0''E 28.vi.-1.vii.2012 D. Ahrens & S. Fabrizi" (ZFMK), 1 ♂ "X-DA3425j Italy Sardegna: Torre Chia (Camping) 38°53'57.5''N, 08°53'10.0''E 28.vi.-1.vii.2012 D. Ahrens & S. Fabrizi" (ZFMK), 1 ♂ "X-DA4652 Italy Sardinia: S. Margherita 20.vi.2015 leg. F. Alamanni" (ZFMK), 1 ♂ "X-DA4654 Italy Sardinia: S. Margherita 20.vi.2015 leg. F. Alamanni" (ZFMK), 1 ♂ "X-DA4655 Italy Sardinia: S. Margherita 20.vi.2015 leg. F. Alamanni" (ZFMK), 1 ♂ "I-Sardinia (CA) Chia dint. 19/05/2001 leg. D. Sechi/ PP1052" (CISP), 1 ♂ "S. Margherita

di Pula (CA) 8-8-1983 / A. Lecis/ PP0197” (CALC), 1 ♂ "S. Margherita di Pula (CA) 5-6-1994 / A. Lecis/ PP0198" (CALC), 1 ♂ "Sardegna (CA) Santa Margherita di Pula 13-VII-1986 leg. D. Atzori/ PP0248" (CMAC), 1 ♂ "Italy - Sardegna Pula (Cagliari) Santa Margherita 22-VI-2013 leg. F. Alamanni/ PP0379” (CDCC), 1 ♂ "Italy - Sardegna Pula (Cagliari) Santa Margherita 22-VI-2013 leg. F. Alamanni/ PP0380” (CDCC), 1 ♂ "Italy - Sardegna Pula (Cagliari) Santa Margherita 22-VI-2013 leg. F. Alamanni/ PP0381” (CDCC), 1 ♂ "Italy - Sardegna Pula (Cagliari) Santa Margherita 22-VI-2013 leg. F. Alamanni/ PP0382” (CDCC), 1 ♂ "Italy - Sardegna Pula (Cagliari) Santa Margherita 20-VI-2013 leg. F. Alamanni/ PP0383” (CDCC), 1 ♂ "Italy - Sardegna Pula (Cagliari) Santa Margherita 20-VI-2013 leg. F. Alamanni/ PP0384” (CDCC), 1 ♂ "Italy - Sardegna Pula (Cagliari) Santa Margherita 20-VI-2013 leg. F. Alamanni/ PP0385” (CDCC), 1 ♂ "Italy - Sardegna Pula (Cagliari) Santa Margherita 20-VI-2013 leg. F. Alamanni/ PP0386” (CDCC), 1 ♂ "Italy - Sardegna Pula (Cagliari) Santa Margherita 22-VI-2013 leg. F. Alamanni/ PP0387” (CDCC), 1 ♂ "Italy - Sardegna Pula (Cagliari) Santa Margherita 22-VI-2013 leg. F. Alamanni/ PP0388” (CDCC), 1 ♂ "Italy - Sardegna Pula (Cagliari) Santa Margherita 22-VI-2013 leg. F. Alamanni/ PP0389” (CDCC), 1 ♂ "Italy - Sardegna Pula (Cagliari) Santa Margherita 22-VI-2013 leg. F. Alamanni/ PP0390” (CDCC), 1 ♂ "Italy - Sardegna Pula (Cagliari) Santa Margherita 22-VI-2013 leg. F. Alamanni/ PP0391” (CDCC), 1 ♂ "Italy - Sardegna Pula (Cagliari) Santa Margherita 20-VI-2013 leg. F. Alamanni/ PP0392” (CDCC), 1 ♂ "Italy - Sardegna Pula (Cagliari) Santa Margherita 20-VI-2013 leg. F. Alamanni/ PP0393” (CDCC), 1 ♂ "Italy - Sardegna Pula (Cagliari) Santa Margherita 20-VI-2013 leg. F. Alamanni/ PP0394” (CDCC), 1 ♂ "Italy - Sardegna Pula (Cagliari) Santa Margherita 20-VI-2013 leg. F. Alamanni/ PP0395” (CDCC), 1 ♂ "Italy - Sardegna Pula (Cagliari) Santa Margherita 20-VI-2013 leg. F. Alamanni/ PP0396” (CDCC), 1 ♂ "Italy - Sardegna Pula (Cagliari) Santa Margherita 20-VI-2013 leg. F. Alamanni/ PP0397” (CDCC), 1 ♂ "Italy - Sardegna Pula (Cagliari) Santa Margherita 20-VI-2013 leg. F. Alamanni/ PP0398” (CDCC), 1 ♂ "Italy - Sardegna Pula (Cagliari) Santa Margherita 20-VI-2013 leg. F. Alamanni/ PP0399” (CDCC), 1 ♂ "Italy - Sardegna Pula (Cagliari) Santa Margherita 20-VI-2013 leg. F. Alamanni/ PP0400” (CDCC), 1 ♂ "Italy - Sardegna Pula (Cagliari) Santa Margherita 20-VI-2013 leg. F. Alamanni/ PP0401” (CDCC), 1 ♂ "Sardegna Pula - CA VI.2013 D. Cillo leg./ PP0402” (CDCC), 1 ♂ "P. melonii sensu Ahrens / Italy - Sardegna Pula (Cagliari) Santa Margherita 20-VI-2013 leg. F. Alamanni/ PP0403” (CDCC), 1 ♂ "Italy - Sardegna Pula (Cagliari) Santa Margherita VI.2013 leg. F. Alamanni/ PP0404” (CDCC), 1 ♂ "Italy - Sardegna Pula (Cagliari) Santa Margherita VI.2013 leg. F. Alamanni/ PP0405” (CDCC), 1 ♂ "Sardegna Chia (CA) 10.VI.2013 leg. E. Bazzato/ PP0406” (CDCC), 1 ♂ "Italy - Sardegna Pula (CA) Santa Margherita VI.2013 leg. F. Alamanni/ PP0407” (CDCC), 1 ♂ "Italy - Sardegna Pula (CA) Santa Margherita VI.2013 leg. F. Alamanni/ PP0408” (CDCC), 1 ♂ "Italy - Sardegna Pula (CA) Santa Margherita VI.2013 leg. F. Alamanni/ PP0409” (CDCC), 1 ♂ "Italy - Sardegna Pula (CA) Santa Margherita VI.2013 leg. F. Alamanni/ PP0410” (CDCC), 1 ♂ "Italy - Sardegna Pula (CA) Santa Margherita VI.2013 leg. F. Alamanni/ PP0411” (CDCC), 1 ♂ "Italy - Sardegna Pula (CA) Santa Margherita VI.2013 leg. F. Alamanni/ PP0412” (CDCC), 1 ♂ "Italy - Sardegna Pula (CA) Santa Margherita VI.2013 leg. F. Alamanni/ PP0413” (CDCC), 1 ♂ "Italy - Sardegna Pula (CA) Santa Margherita VI.2013 leg. F. Alamanni/ PP0414” (CDCC), 1 ♂ "Sardegna Chia - 13-VI.2010 leg. R. Rattu/ PP0415” (CDCC), 1 ♂ "Italy - Sardegna Pula (Cagliari) Santa

Margherita VI.2013 leg. F. Alamanni/ PP0416” (CDCC), 1 ♂ "Italy - Sardegna Pula (Cagliari) Santa Margherita VI.2013 leg. F. Alamanni/ PP0417” (CDCC), 1 ♂ "Italy - Sardegna Pula (Cagliari) Santa Margherita VI.2013 leg. F. Alamanni/ PP0418” (CDCC), 1 ♂ "Italy - Sardegna Pula (Cagliari) Santa Margherita VI.2013 leg. F. Alamanni/ PP0419” (CDCC), 1 ♂ "Italy - Sardegna Pula (Cagliari) Santa Margherita VI.2013 leg. F. Alamanni/ PP0420” (CDCC), 1 ♂ "P. melonii sensu Ahrens / Sardegna (CA) Chia 10.VI.1990 leg. Fancello/ PP0471” (CDCC).

***Pachypus matzaccara* sp. n.**

Type material examined. (ID based on IUMG): Paratypes: 1 ♂ "Sardegna Matzaccara Carb. Igles 2.VII.2013 lg. Fancello/ PP0432” (CDCC), 1 ♂ "Sardegna (CA) Matzaccara VI.2013 leg. Fancello/ PP0433” (CDCC), 1 ♂ "Sardegna (CA) Matzaccara VI.2013 leg. Fancello/ PP0434” (CDCC), 1 ♂ "Sardegna (CA) Matzaccara VI.2013 leg. Fancello/ PP0435” (CDCC), 1 ♂ "Sardegna Matzaccara (CI) 24.VI.2014 leg. D. Cillo/ PP0443” (CDCC), 1 ♂ "Sardegna Matzaccara (CI) 24.VI.2014 leg. D. Cillo/ PP0444” (CDCC), 1 ♂ "Sardegna Matzaccara (CI) 24.VI.2014 leg. D. Cillo/ PP0445” (CDCC), 1 ♂ "Sardegna Matzaccara (CI) 24.VI.2014 leg. D. Cillo/ PP0446” (CDCC), 1 ♂ "Sardegna Matzaccara (CI) 24.VI.2014 leg. D. Cillo/ PP0447” (CDCC), 1 ♂ "Sardegna Matzaccara (CI) 24.VI.2014 leg. D. Cillo/ PP0448” (CDCC), 1 ♂ "Sardegna Matzaccara (CI) 24.VI.2014 leg. D. Cillo/ PP0449” (CDCC), 1 ♂ "Sardegna Matzaccara (CI) 24.VI.2014 leg. D. Cillo/ PP0450” (CDCC).

***Pachypus sardiniensis* Guerlach, Bazzato & Cillo, 2018**

Type material examined. (ID based on IUMG): Paratypes: 1 ♂ "Paratypus Pachypus sardiniensis n. sp. G. Guerlach, E. Bazzato & D. Cillo / Sardegna Flumini - Quartu S.E. 6.04 leg. E. Bazzato/ PP0080" (CEBQ), 1 ♂ "Paratypus Pachypus sardiniensis n. sp. G. Guerlach, E. Bazzato & D. Cillo / Sardegna Flumini - Quartu S.E. 6.04 leg. E. Bazzato/ PP0081" (CEBQ), 1 ♂ "Paratypus Pachypus sardiniensis n. sp. G. Guerlach, E. Bazzato & D. Cillo / Sardegna Flumini - Quartu S.E. 6.04 leg. E. Bazzato/ PP0082" (CEBQ), 1 ♂ "Paratypus Pachypus sardiniensis n. sp. G. Guerlach, E. Bazzato & D. Cillo / Sardegna Flumini - Quartu S.E. 6.04 leg. E. Bazzato/ PP0083" (CEBQ), 1 ♂ "Paratypus Pachypus sardiniensis n. sp. G. Guerlach, E. Bazzato & D. Cillo / Sardegna Flumini - Quartu S.E. 6.04 leg. E. Bazzato/ PP0084" (CEBQ), 1 ♂ "Paratypus Pachypus sardiniensis n. sp. G. Guerlach, E. Bazzato & D. Cillo / Sardegna Quartu S.E. 7.04 leg. E. Bazzato/ PP0085" (CEBQ), 1 ♂ "Paratypus Pachypus sardiniensis n. sp. G. Guerlach, E. Bazzato & D. Cillo / Sardegna Quartu S.E. 7.04 leg. E. Bazzato/ PP0086" (CEBQ), 1 ♂ "Paratypus Pachypus sardiniensis n. sp. G. Guerlach, E. Bazzato & D. Cillo / Sardegna Quartu S.E. 7.04 leg. E. Bazzato/ PP0087" (CEBQ), 1 ♂ "Paratypus Pachypus sardiniensis n. sp. G. Guerlach, E. Bazzato & D. Cillo / Sardegna Quartu S.E. 7.04 leg. E. Bazzato/ PP0088" (CEBQ), 1 ♂ "Paratypus Pachypus sardiniensis n. sp. G. Guerlach, E. Bazzato & D. Cillo / Sardegna Quartu S.E. 7.04 leg. E. Bazzato/ PP0089" (CEBQ), 1 ♂ "Paratypus Pachypus sardiniensis n. sp. G. Guerlach, E. Bazzato & D. Cillo / Sardegna Quartu S.E.

7.04 leg. E. Bazzato/ PP0090" (CEBQ), 1 ♂ "Paratypus Pachypus sardiniensis n. sp. G. Guerlach, E. Bazzato & D. Cillo / Sardegna Quartu S.E. 7.04 leg. E. Bazzato/ PP0091" (CEBQ), 1 ♂ "Paratypus Pachypus sardiniensis n. sp. G. Guerlach, E. Bazzato & D. Cillo / Sardegna Quartu S.E. 7.04 leg. E. Bazzato/ PP0092" (CEBQ), 1 ♂ "Paratypus Pachypus sardiniensis n. sp. G. Guerlach, E. Bazzato & D. Cillo / Italy - Sardegna Maracalagonis Torre delle Stelle 17.VII.2009 leg. D. Cillo/ PP0490" (CDCC), 1 ♂ "Paratypus Pachypus sardiniensis n. sp. G. Guerlach, E. Bazzato & D. Cillo / Italy - Sardegna Maracalagonis Torre delle Stelle 17.VII.2009 leg. D. Cillo/ PP0491" (CDCC), 1 ♂ "Paratypus / Sardegna Frutti d'Oro (CA) 5.VI.1995 leg. D. Cillo/ PP0492" (CDCC), 1 ♂" Pachypus sardiniensis / Sardegna San Vito VI.2011 leg. C. Onnis/ PP0496" (CDCC), 1 ♂ "Paratypus Pachypus sardiniensis n. sp. G. Guerlach, E. Bazzato & D. Cillo / Sardegna Quartu S.E. Foxi - VII.2009 leg. E. Bazzato/ PP0497" (CDCC), 1 ♂ "Paratypus Pachypus sardiniensis n. sp. G. Guerlach, E. Bazzato & D. Cillo / Italy - Sardegna Quartucciu (Cagliari) S. Isidoro leg. D. Cillo 6.2010/ PP0555" (CDCC), 1 ♂ "Paratypus Pachypus sardiniensis n. sp. G. Guerlach, E. Bazzato & D. Cillo / Italy - Sardegna Burcei (Cagliari) Riu Ollastu 11.6.2010 leg. D. Cillo/ PP0556" (CDCC), 1 ♂ "Paratypus Pachypus sardiniensis n. sp. G. Guerlach, E. Bazzato & D. Cillo / Italy - Sardegna Burcei (Cagliari) Riu Ollastu 11.6.2010 leg. D. Cillo/ PP0557" (CDCC), 1 ♂ "Paratypus Pachypus sardiniensis n. sp. G. Guerlach, E. Bazzato & D. Cillo / Italy - Sardegna Maracalagonis (Cagliari) Villaggio dei Gigli 15.VI.2009 leg. M.G. Atzori/ PP0596" (CDCC), 1 ♂ "Paratypus Pachypus sardiniensis n. sp. G. Guerlach, E. Bazzato & D. Cillo / Italy - Sardegna Maracalagonis (Cagliari) Villaggio dei Gigli 15.VI.2009 leg. M.G. Atzori/ PP0597" (CDCC), 1 ♂ "Paratypus Pachypus sardiniensis n. sp. G. Guerlach, E. Bazzato & D. Cillo / Italy - Sardegna Maracalagonis (Cagliari) Villaggio dei Gigli 15.VI.2009 leg. M.G. Atzori/ PP0598" (CDCC), 1 ♂ "Paratypus Pachypus sardiniensis n. sp. G. Guerlach, E. Bazzato & D. Cillo / Italy - Sardegna Maracalagonis (Cagliari) Villaggio dei Gigli 15.VI.2009 leg. M.G. Atzori/ PP0599" (CDCC), 1 ♂ "Paratypus Pachypus sardiniensis n. sp. G. Guerlach, E. Bazzato & D. Cillo / Italy - Sardegna Maracalagonis (Cagliari) Villaggio dei Gigli 15.VI.2009 leg. M.G. Atzori/ PP0600" (CDCC), 1 ♂ "Paratypus Pachypus sardiniensis n. sp. G. Guerlach, E. Bazzato & D. Cillo / Italy - Sardegna Maracalagonis Torre delle Stelle 15.VI.2012 leg. D. Cillo/ PP0601" (CDCC), 1 ♂ "Paratypus Pachypus sardiniensis n. sp. G. Guerlach, E. Bazzato & D. Cillo / Italy - Sardegna Maracalagonis Torre delle Stelle 15.VI.2012 leg. D. Cillo/ PP0602" (CDCC), 1 ♂ "Paratypus Pachypus sardiniensis n. sp. G. Guerlach, E. Bazzato & D. Cillo / Italy - Sardegna Maracalagonis Torre delle Stelle 15.VI.2012 leg. D. Cillo/ PP0603" (CDCC), 1 ♂ "Paratypus Pachypus sardiniensis n. sp. G. Guerlach, E. Bazzato & D. Cillo / Italy - Sardegna Maracalagonis Torre delle Stelle 15.VI.2012 leg. D. Cillo/ PP0604" (CDCC), 1 ♂ "Paratypus Pachypus sardiniensis n. sp. G. Guerlach, E. Bazzato & D. Cillo / Italy - Sardegna Maracalagonis Torre delle Stelle 15.VI.2012 leg. D. Cillo/ PP0605" (CDCC), 1 ♂ "Paratypus Pachypus sardiniensis n. sp. G. Guerlach, E. Bazzato & D. Cillo / Italy - Sardegna Maracalagonis Torre delle Stelle 15.VI.2012 leg. D. Cillo/ PP0606" (CDCC), 1 ♂ "Paratypus Pachypus sardiniensis n. sp. G. Guerlach, E. Bazzato & D. Cillo / Italy - Sardegna Maracalagonis Torre delle Stelle 15.VI.2012 leg. D. Cillo/ PP0607" (CDCC), 1 ♂ "Paratypus Pachypus sardiniensis n. sp. G. Guerlach, E. Bazzato & D. Cillo / Italy - Sardegna Maracalagonis Torre delle Stelle 03.VII.2012 leg. D. Cillo/ PP0608" (CDCC), 1 ♂ "Paratypus Pachypus sardiniensis n. sp. G. Guerlach, E.

Bazzato & D. Cillo / Italy - Sardegna Maracalagonis Torre delle Stelle 03.VII.2012 leg. D. Cillo/ PP0609" (CDCC), 1 ♂ "Paratypus Pachypus sardiniensis n. sp. G. Guerlach, E. Bazzato & D. Cillo / Italy - Sardegna Maracalagonis Torre delle Stelle 03.VII.2012 leg. D. Cillo/ PP0610" (CDCC), 1 ♂ "Paratypus Pachypus sardiniensis n. sp. G. Guerlach, E. Bazzato & D. Cillo / Italy - Sardegna Burcei (Cagliari) Riu Ollastu 11.6.2010 leg. D. Cillo/ PP0620" (CDCC), 1 ♂ "Paratypus Pachypus sardiniensis n. sp. G. Guerlach, E. Bazzato & D. Cillo / Italy - Sardegna Quartucciu (Cagliari) S. Isidoro leg. D. Cillo 29.6.2010/ PP0621" (CDCC), 1 ♂ "Paratypus Pachypus sardiniensis n. sp. G. Guerlach, E. Bazzato & D. Cillo / Italy - Sardegna Maracalagonis (Cagliari) Villaggio dei Gigli 15.VI.2009 leg. M.G. Atzori/ PP0624" (CDCC), 1 ♂ "Paratypus Pachypus sardiniensis n. sp. G. Guerlach, E. Bazzato & D. Cillo / Italy - Sardegna Maracalagonis Torre delle Stelle 15.VI.2012 leg. D. Cillo/ PP0625" (CDCC), 1 ♂ "Paratypus Pachypus sardiniensis n. sp. G. Guerlach, E. Bazzato & D. Cillo / Italy - Sardegna Maracalagonis Torre delle Stelle 15.VI.2012 leg. D. Cillo/ PP0626" (CDCC), 1 ♂ "Paratypus Pachypus sardiniensis n. sp. G. Guerlach, E. Bazzato & D. Cillo / Italy - Sardegna Maracalagonis Torre delle Stelle 03.VII.2012 leg. D. Cillo/ PP0642" (CDCC), 1 ♂ "Paratypus Pachypus sardiniensis n. sp. G. Guerlach, E. Bazzato & D. Cillo / Italy - Sardegna Maracalagonis Torre delle Stelle 03.VII.2012 leg. D. Cillo/ PP0643" (CDCC), 1 ♂ "Paratypus Pachypus sardiniensis n. sp. G. Guerlach, E. Bazzato & D. Cillo / Italy - Sardegna Maracalagonis Torre delle Stelle 04.VII.2012 leg. D. Cillo/ PP0644" (CDCC), 1 ♂ "Paratypus Pachypus sardiniensis n. sp. G. Guerlach, E. Bazzato & D. Cillo / Italy - Sardegna Maracalagonis Torre delle Stelle 04.VII.2012 leg. D. Cillo/ PP0645" (CDCC), 1 ♂ "Paratypus Pachypus sardiniensis n. sp. G. Guerlach, E. Bazzato & D. Cillo / Italy - Sardegna Maracalagonis Torre delle Stelle 03.VII.2012 leg. D. Cillo/ PP0646" (CDCC), 1 ♂ "Paratypus Pachypus sardiniensis n. sp. G. Guerlach, E. Bazzato & D. Cillo / Italy - Sardegna Maracalagonis Torre delle Stelle 03.VII.2012 leg. D. Cillo/ PP0647" (CDCC), 1 ♂ "Paratypus Pachypus sardiniensis n. sp. G. Guerlach, E. Bazzato & D. Cillo / Italy - Sardegna Maracalagonis Torre delle Stelle 03.VII.2012 leg. D. Cillo/ PP0648" (CDCC), 1 ♂ "Paratypus Pachypus sardiniensis n. sp. G. Guerlach, E. Bazzato & D. Cillo / Italy - Sardegna Maracalagonis Torre delle Stelle 03.VII.2012 leg. D. Cillo/ PP0649" (CDCC), 1 ♂ "Paratypus Pachypus sardiniensis n. sp. G. Guerlach, E. Bazzato & D. Cillo / Italy - Sardegna Maracalagonis Torre delle Stelle 15.VI.2012 leg. D. Cillo/ PP0659" (CDCC), 1 ♂ "Paratypus Pachypus sardiniensis n. sp. G. Guerlach, E. Bazzato & D. Cillo / Sardegna S. Pietro Parad. CA 19.07.07 leg. D. Cillo/ PP0706" (CDCC), 1 ♂ "Paratypus Pachypus sardiniensis n. sp. G. Guerlach, E. Bazzato & D. Cillo / Sardegna S. Pietro Parad. CA 18.07.07 leg. D. Cillo/ PP0707" (CDCC), 1 ♂ "Paratypus Pachypus sardiniensis n. sp. G. Guerlach, E. Bazzato & D. Cillo / Italia - Sardegna Quartu S.E. 6.07 leg. D. Cillo/ PP0708" (CDCC), 1 ♂ "Paratypus Pachypus sardiniensis n. sp. G. Guerlach, E. Bazzato & D. Cillo / Italia - Sardegna Quartu S.E. 6.07 leg. D. Cillo/ PP0709" (CDCC), 1 ♂ "Paratypus Pachypus sardiniensis n. sp. G. Guerlach, E. Bazzato & D. Cillo / Italia - Sardegna Quartu S.E. 6.07 leg. D. Cillo/ PP0710" (CDCC), 1 ♂ "Paratypus Pachypus sardiniensis n. sp. G. Guerlach, E. Bazzato & D. Cillo / Italia - Sardegna Sinnai - CA 6.07 leg. D. Cillo/ PP0711" (CDCC), 1 ♂ "Paratypus Pachypus sardiniensis n. sp. G. Guerlach, E. Bazzato & D. Cillo / Italia - Sardegna Sinnai - CA 6.07 leg. D. Cillo/ PP0712" (CDCC), 1 ♂ "Paratypus Pachypus sardiniensis n. sp. G. Guerlach, E. Bazzato & D. Cillo / Italia - Sardegna S. Isidoro - CA 25.6.07 leg. D. Cillo/ PP0713" (CDCC), 1 ♂ "Paratypus

Pachypus sardiniensis n. sp. G. Guerlach, E. Bazzato & D. Cillo / Italia - Sardegna S. Isidoro - CA 25.6.07 leg. D. Cillo/ PP0714" (CDCC), 1 ♂ "Paratypus Pachypus sardiniensis n. sp. G. Guerlach, E. Bazzato & D. Cillo / Sardegna Flumini D.Q. 6.04 leg. D. Cillo/ PP0715" (CDCC), 1 ♂ "Paratypus Pachypus sardiniensis n. sp. G. Guerlach, E. Bazzato & D. Cillo / Sardegna Flumini D.Q. 6.04 leg. D. Cillo/ PP0716" (CDCC), 1 ♂ "Paratypus Pachypus sardiniensis n. sp. G. Guerlach, E. Bazzato & D. Cillo / Sardegna Flumini D.Q. 6.04 leg. D. Cillo/ PP0717" (CDCC), 1 ♂ "Paratypus Pachypus sardiniensis n. sp. G. Guerlach, E. Bazzato & D. Cillo / Italia - Sardegna S. Isidoro CA - 26.6.08 leg. D. Cillo/ PP0718" (CDCC), 1 ♂ "Paratypus Pachypus sardiniensis n. sp. G. Guerlach, E. Bazzato & D. Cillo / Italia - Sardegna S. Isidoro - CA 26.6.08 leg. D. Cillo/ PP0719" (CDCC), 1 ♂ "Paratypus Pachypus sardiniensis n. sp. G. Guerlach, E. Bazzato & D. Cillo / Italia - Sardegna S. Isidoro CA - 26.6.08 leg. D. Cillo/ PP0720" (CDCC), 1 ♂ "Paratypus Pachypus sardiniensis n. sp. G. Guerlach, E. Bazzato & D. Cillo / Italia - Sardegna S. Isidoro - CA 28.6.08 leg. D. Cillo/ PP0721" (CDCC), 1 ♂ "Paratypus Pachypus sardiniensis n. sp. G. Guerlach, E. Bazzato & D. Cillo / Italia - Sardegna S. Isidoro - CA 28.6.08 leg. D. Cillo/ PP0722" (CDCC), 1 ♂ "Paratypus Pachypus sardiniensis n. sp. G. Guerlach, E. Bazzato & D. Cillo / Italia - Sardegna S. Isidoro - CA 6.7.08 leg. D. Cillo/ PP0723" (CDCC), 1 ♂ "Paratypus Pachypus sardiniensis n. sp. G. Guerlach, E. Bazzato & D. Cillo / Italia - Sardegna S. Isidoro - CA 6.7.08 leg. D. Cillo/ PP0724" (CDCC), 1 ♂ "Paratypus Pachypus sardiniensis n. sp. G. Guerlach, E. Bazzato & D. Cillo / Italia - Sardegna S. Isidoro - CA 25.6.07 leg. D. Cillo/ PP0725" (CDCC), 1 ♂ "Paratypus Pachypus sardiniensis n. sp. G. Guerlach, E. Bazzato & D. Cillo / Italia - Sardegna Sinnai - CA 6.07 leg. D. Cillo/ PP0726" (CDCC), 1 ♂ "Paratypus Pachypus sardiniensis n. sp. G. Guerlach, E. Bazzato & D. Cillo / Italy - Sardegna Rio Ollastu 6.07 leg. D. Cillo/ PP0727" (CDCC), 1 ♂ "Paratypus Pachypus sardiniensis n. sp. G. Guerlach, E. Bazzato & D. Cillo / Sardegna Flumini Quartu S.E. 24.6.01 leg. D. Cillo/ PP0751" (CDCC), 1 ♂ "Paratypus Pachypus sardiniensis n. sp. G. Guerlach, E. Bazzato & D. Cillo / Sardegna Flumini Quartu S.E. 24.6.01 leg. D. Cillo/ PP0752" (CDCC), 1 ♂ "Paratypus Pachypus sardiniensis n. sp. G. Guerlach, E. Bazzato & D. Cillo / Sardegna Flumini D.Q. 6.04 leg. D. Cillo/ PP0753" (CDCC), 1 ♂ "Paratypus Pachypus sardiniensis n. sp. G. Guerlach, E. Bazzato & D. Cillo / Sardegna Flumini D.Q. 6.04 leg. D. Cillo/ PP0754" (CDCC), 1 ♂ "Paratypus Pachypus sardiniensis n. sp. G. Guerlach, E. Bazzato & D. Cillo / Sardegna Flumini D.Q. 6.04 leg. D. Cillo/ PP0755" (CDCC), 1 ♂ "Paratypus Pachypus sardiniensis n. sp. G. Guerlach, E. Bazzato & D. Cillo / Sardegna Flumini D.Q. 6.04 leg. D. Cillo/ PP0756" (CDCC), 1 ♂ "Paratypus Pachypus sardiniensis n. sp. G. Guerlach, E. Bazzato & D. Cillo / Sardegna Flumini D.Q. 6.04 leg. D. Cillo/ PP0757" (CDCC), 1 ♂ "Paratypus Pachypus sardiniensis n. sp. G. Guerlach, E. Bazzato & D. Cillo / Sardegna Flumini D.Q. 6.04 leg. D. Cillo/ PP0758" (CDCC), 1 ♂ "Paratypus Pachypus sardiniensis n. sp. G. Guerlach, E. Bazzato & D. Cillo / Sardegna Flumini D.Q. 6.04 leg. D. Cillo/ PP0759" (CDCC), 1 ♂ "Paratypus Pachypus sardiniensis n. sp. G. Guerlach, E. Bazzato & D. Cillo / Sardegna Flumini D.Q. 6.04 leg. D. Cillo/ PP0760" (CDCC), 1 ♂ "Paratypus Pachypus sardiniensis n. sp. G. Guerlach, E. Bazzato & D. Cillo / Sardegna Flumini D.Q. 6.04 leg. D. Cillo/ PP0761" (CDCC), 1 ♂ "Paratypus Pachypus sardiniensis n. sp. G. Guerlach, E. Bazzato & D. Cillo / Sardegna Flumini D.Q. 6.04 leg. D. Cillo/ PP0762" (CDCC), 1 ♂ "Paratypus Pachypus sardiniensis n. sp. G. Guerlach, E. Bazzato & D. Cillo / Sardegna Flumini

D.Q. 6.04 leg. D. Cillo/ PP0763" (CDCC), 1 ♂ "Paratypus Pachypus sardiniensis n. sp. G. Guerlach, E. Bazzato & D. Cillo / Sardegna Flumini D.Q. 6.03 leg. D. Cillo/ PP0764" (CDCC), 1 ♂ "Paratypus Pachypus sardiniensis n. sp. G. Guerlach, E. Bazzato & D. Cillo / Sardegna Flumini D.Q. 7.04 leg. D. Cillo/ PP0765" (CDCC), 1 ♂ "Paratypus Pachypus sardiniensis n. sp. G. Guerlach, E. Bazzato & D. Cillo / Sardegna Flumini D.Q. 7.04 leg. D. Cillo/ PP0766" (CDCC), 1 ♂ "Paratypus Pachypus sardiniensis n. sp. G. Guerlach, E. Bazzato & D. Cillo / Sardegna Quartu S.E. Flumini 17.6.98 leg. D. Cillo/ PP0767" (CDCC), 1 ♂ "Paratypus Pachypus sardiniensis n. sp. G. Guerlach, E. Bazzato & D. Cillo / Sardegna Geremeas 5.03 leg. D. Cillo/ PP0768" (CDCC), 1 ♂ "Paratypus Pachypus sardiniensis n. sp. G. Guerlach, E. Bazzato & D. Cillo / Sardegna Geremeas 6.03 leg. D. Cillo/ PP0769" (CDCC), 1 ♂ "Paratypus Pachypus sardiniensis n. sp. G. Guerlach, E. Bazzato & D. Cillo / Sardegna Geremeas 6.03 leg. D. Cillo/ PP0770" (CDCC), 1 ♂ "Paratypus Pachypus sardiniensis n. sp. G. Guerlach, E. Bazzato & D. Cillo / Sardegna Geremeas 6.03 leg. D. Cillo/ PP0771" (CDCC), 1 ♂ "Paratypus Pachypus sardiniensis n. sp. G. Guerlach, E. Bazzato & D. Cillo / Sardegna Cast. Quirra 29.6.08 leg. D. Cillo/ PP0772" (CDCC), 1 ♂ "Paratypus Pachypus sardiniensis n. sp. G. Guerlach, E. Bazzato & D. Cillo / Sardegna Cast. Quirra 29.6.08 leg. D. Cillo/ PP0773" (CDCC), 1 ♂ "Paratypus Pachypus sardiniensis n. sp. G. Guerlach, E. Bazzato & D. Cillo / Sardegna Cast. Quirra 29.6.08 leg. D. Cillo/ PP0774" (CDCC), 1 ♂ "Paratypus Pachypus sardiniensis n. sp. G. Guerlach, E. Bazzato & D. Cillo / Sardegna Cast. Quirra 29.6.08 leg. D. Cillo/ PP0775" (CDCC), 1 ♂ "Paratypus Pachypus sardiniensis n. sp. G. Guerlach, E. Bazzato & D. Cillo / Sardegna T. delle Stelle 14.6.86 leg. D. Cillo/ PP0778" (CDCC), 1 ♂ "Paratypus Pachypus sardiniensis n. sp. G. Guerlach, E. Bazzato & D. Cillo / Sardegna Flumini D.Q. 6.03 leg. D. Cillo/ PP0779" (CDCC), 1 ♂ "Paratypus Pachypus sardiniensis n. sp. G. Guerlach, E. Bazzato & D. Cillo / Sardegna Flumini D.Q. 6.04 leg. D. Cillo/ PP0780" (CDCC), 1 ♂ "Paratypus Pachypus sardiniensis n. sp. G. Guerlach, E. Bazzato & D. Cillo / Sardegna Flumini D.Q. 6.04 leg. D. Cillo/ PP0781" (CDCC), 1 ♂ "Paratypus Pachypus sardiniensis n. sp. G. Guerlach, E. Bazzato & D. Cillo / Sardegna Flumini Quartu S.E. 24.6.01 leg. D. Cillo/ PP0782" (CDCC), 1 ♂ "Paratypus Pachypus sardiniensis n. sp. G. Guerlach, E. Bazzato & D. Cillo / Sardegna Geremeas 5.6.02 leg. D. Cillo/ PP0783" (CDCC), 1 ♂ "Paratypus Pachypus sardiniensis n. sp. G. Guerlach, E. Bazzato & D. Cillo / Sardegna T. delle Stelle 14.6.86 leg. D. Cillo/ PP0784" (CDCC), 1 ♂ "Paratypus Pachypus sardiniensis n. sp. G. Guerlach, E. Bazzato & D. Cillo / S. Isidoro Quartucciu 25.5.95 leg. D. Cillo/ PP0785" (CDCC), 1 ♂ "Paratypus Pachypus sardiniensis n. sp. G. Guerlach, E. Bazzato & D. Cillo / S. Isidoro Quartucciu 25.5.95 leg. D. Cillo/ PP0786" (CDCC), 1 ♂ "Paratypus Pachypus sardiniensis n. sp. G. Guerlach, E. Bazzato & D. Cillo / Sardegna S. Isidoro - CA 30.6.09 leg. E. Bazzato/ PP0787" (CDCC), 1 ♂ "Paratypus Pachypus sardiniensis n. sp. G. Guerlach, E. Bazzato & D. Cillo / Italia - Sardegna S. Isidoro - CA 30.6.09 leg. D. Cillo/ PP0788" (CDCC), 1 ♂ "Paratypus Pachypus sardiniensis n. sp. G. Guerlach, E. Bazzato & D. Cillo / Italia - Sardegna S. Isidoro - CA 3.7.09 leg. D. Cillo/ PP0789" (CDCC), 1 ♂ "Paratypus Pachypus sardiniensis n. sp. G. Guerlach, E. Bazzato & D. Cillo / Italia - Sardegna S. Isidoro - CA 7.09 leg. D. Cillo/ PP0790" (CDCC), 1 ♂ "Paratypus Pachypus sardiniensis n. sp. G. Guerlach, E. Bazzato & D. Cillo / Italia - Sardegna San Gregorio 28.5.09 leg. D. Cillo/ PP0791" (CDCC), 1 ♂ "Paratypus Pachypus sardiniensis n. sp. G. Guerlach, E.

Bazzato & D. Cillo / Sardegna Maracalagonis Torre delle Stelle VII.2012 leg. D. Cillo/ PP0792" (CDCC), 1 ♂ "Paratypus Pachypus sardiniensis n. sp. G. Guerlach, E. Bazzato & D. Cillo / Sardegna Maracalagonis Torre delle Stelle 3.VII.2012 D. Cillo leg./ PP0793" (CDCC), 1 ♂ "Paratypus Pachypus sardiniensis n. sp. G. Guerlach, E. Bazzato & D. Cillo / Sardegna Murta Sterria 17.VI.05/ PP0794" (CDCC), 1 ♂ "Paratypus Pachypus sardiniensis n. sp. G. Guerlach, E. Bazzato & D. Cillo / Sardegna Murta Sterria 17.VI.05/ PP0795" (CDCC), 1 ♂ "Paratypus Pachypus sardiniensis n. sp. G. Guerlach, E. Bazzato & D. Cillo / Italy - Sardegna Mara - CA Burranca 5.6.93 leg. D. Cillo/ PP0796" (CDCC), 1 ♂ "Paratypus Pachypus sardiniensis n. sp. G. Guerlach, E. Bazzato & D. Cillo / Italy - Sardegna Mara - CA Burranca 5.6.93 leg. D. Cillo/ PP0797" (CDCC), 1 ♂ "Paratypus Pachypus sardiniensis n. sp. G. Guerlach, E. Bazzato & D. Cillo / Italia - Sardegna Burcei - CA Rio Ollastu 28.5.09 leg. D. Cillo/ PP0798" (CDCC), 1 ♂ "Paratypus Pachypus sardiniensis n. sp. G. Guerlach, E. Bazzato & D. Cillo / Italia - Sardegna Burcei - CA Rio Ollastu 28.5.09 leg. D. Cillo/ PP0799" (CDCC), 1 ♂ "Paratypus Pachypus sardiniensis n. sp. G. Guerlach, E. Bazzato & D. Cillo / Italia - Sardegna S. Isidoro - CA 3.7.09 leg. D. Cillo/ PP0800" (CDCC), 1 ♂ "Paratypus Pachypus sardiniensis n. sp. G. Guerlach, E. Bazzato & D. Cillo / Italia - Sardegna S. Isidoro - CA 3.7.09 leg. D. Cillo/ PP0801" (CDCC), 1 ♂ "Paratypus Pachypus sardiniensis n. sp. G. Guerlach, E. Bazzato & D. Cillo / Italia - Sardegna S. Isidoro - CA 3.7.09 leg. D. Cillo/ PP0802" (CDCC), 1 ♂ "Paratypus Pachypus sardiniensis n. sp. G. Guerlach, E. Bazzato & D. Cillo / Italia - Sardegna Torre del. Stelle Maracalagonis CA - 17.7.09 leg. D. Cillo/ PP0803" (CDCC), 1 ♂ "Paratypus Pachypus sardiniensis n. sp. G. Guerlach, E. Bazzato & D. Cillo / Italia - Sardegna Torre del. Stelle Maracalagonis CA - 17.VII.2009 leg. D. Cillo/ PP0804" (CDCC), 1 ♂ "Paratypus Pachypus sardiniensis n. sp. G. Guerlach, E. Bazzato & D. Cillo / Italia - Sardegna Torre del. Stelle Maracalagonis CA - 17.VII.2009 leg. D. Cillo/ PP0805" (CDCC), 1 ♂ "Paratypus Pachypus sardiniensis n. sp. G. Guerlach, E. Bazzato & D. Cillo / Italia - Sardegna Torre del. Stelle Maracalagonis CA - 17.7.09 leg. D. Cillo/ PP0806" (CDCC), 1 ♂ "Paratypus Pachypus sardiniensis n. sp. G. Guerlach, E. Bazzato & D. Cillo / Italia - Sardegna Torre del. Stelle Maracalagonis CA - 17.7.09 leg. D. Cillo/ PP0807" (CDCC).

Additional material examined. (identification based on IUMG)**:** 1 ♂ "X-DA3968 Italia Sardinia: Marcalagonis (CA) Vill. dei Gigli 15.VI.2009 leg. M.G. Atzori" (ZFMK), 1 ♂ "X-DA3970 Italia Sardinia: Marcalagonis (CA) Vill. dei Gigli 15.VI.2009 leg. M.G. Atzori" (ZFMK), 1 ♂ "X-DA3972 - Italia, Sardinia: Torre delle Stelle, 4.vii.2012, leg. D. Cillo" (ZFMK), 1 ♂ "X-DA3332b Italy Sardegna: Villasimius (Camping Spiaggia del Riso) 29°07'23.1'N [sic!; correct: 39°07'23.1''N], 09°30'40.6"E 27.VI.2012 D. Ahrens & S. Fabrizi" (ZFMK), 1 ♂ "X-DA3332c Italy Sardegna: Villasimius (Camping Spiaggia del Riso) 29°07'23.1'N [sic!; correct: 39°07'23.1''N], 09°30'40.6"E 27.VI.2012 D. Ahrens & S. Fabrizi" (ZFMK), 1 ♂ "X-DA3332d Italy Sardegna: Villasimius (Camping Spiaggia del Riso) 29°07'23.1'N [sic!; correct: 39°07'23.1''N], 09°30'40.6"E 27.VI.2012 D. Ahrens & S. Fabrizi" (ZFMK), 1 ♂ "X-DA3332e Italy Sardegna: Villasimius (Camping Spiaggia del Riso) 29°07'23.1'N [sic!; correct: 39°07'23.1''N], 09°30'40.6"E 27.VI.2012 D. Ahrens & S. Fabrizi" (ZFMK), 1 ♂ "X-DA3332f Italy Sardegna: Villasimius (Camping Spiaggia del Riso) 29°07'23.1'N [sic!; correct: 39°07'23.1''N], 09°30'40.6"E 27.VI.2012 D. Ahrens & S. Fabrizi" (ZFMK), 1 ♂ "X-DA3332g Italy Sardegna: Villasimius (Camping Spiaggia del Riso) 29°07'23.1'N [sic!; correct: 39°07'23.1''N], 09°30'40.6"E 27.VI.2012 D. Ahrens &

S. Fabrizi" (ZFMK), 1 ♂ "X-DA3332h Italy Sardegna: Villasimius (Camping Spiaggia del Riso) 29°07'23.1'N [sic!; correct: 39°07'23.1''N], 09°30'40.6''E 27.VI.2012 D. Ahrens & S. Fabrizi" (ZFMK), 1 ♂ "X-DA3332i Italy Sardegna: Villasimius (Camping Spiaggia del Riso) 29°07'23.1'N [sic!; correct: 39°07'23.1''N], 09°30'40.6''E 27.VI.2012 D. Ahrens & S. Fabrizi" (ZFMK), 1 ♂ "X-DA3332j Italy Sardegna: Villasimius (Camping Spiaggia del Riso) 29°07'23.1'N [sic!; correct: 39°07'23.1''N], 09°30'40.6''E 27.VI.2012 D. Ahrens & S. Fabrizi" (ZFMK), 1 ♂ "X-DA3332k Italy Sardegna: Villasimius (Camping Spiaggia del Riso) 29°07'23.1'N [sic!; correct: 39°07'23.1''N], 09°30'40.6''E 27.VI.2012 D. Ahrens & S. Fabrizi" (ZFMK), 1 ♂ "X-DA3332l Italy Sardegna: Villasimius (Camping Spiaggia del Riso) 29°07'23.1'N [sic!; correct: 39°07'23.1''N], 09°30'40.6''E 27.VI.2012 D. Ahrens & S. Fabrizi" (ZFMK), 1 ♂ "X-DA3332m Italy Sardegna: Villasimius (Camping Spiaggia del Riso) 29°07'23.1'N [sic!; correct: 39°07'23.1''N], 09°30'40.6''E 27.VI.2012 D. Ahrens & S. Fabrizi" (ZFMK), 1 ♂ "X-DA3332n Italy Sardegna: Villasimius (Camping Spiaggia del Riso) 29°07'23.1'N [sic!; correct: 39°07'23.1''N], 09°30'40.6''E 27.VI.2012 D. Ahrens & S. Fabrizi" (ZFMK), 1 ♂ "X-DA3332o Italy Sardegna: Villasimius (Camping Spiaggia del Riso) 29°07'23.1'N [sic!; correct: 39°07'23.1''N], 09°30'40.6''E 27.VI.2012 D. Ahrens & S. Fabrizi" (ZFMK), 1 ♂ "X-DA3332p Italy Sardegna: Villasimius (Camping Spiaggia del Riso) 29°07'23.1'N [sic!; correct: 39°07'23.1''N], 09°30'40.6''E 27.VI.2012 D. Ahrens & S. Fabrizi" (ZFMK), 1 ♂ "X-DA3332q Italy Sardegna: Villasimius (Camping Spiaggia del Riso) 29°07'23.1'N [sic!; correct: 39°07'23.1''N], 09°30'40.6''E 27.VI.2012 D. Ahrens & S. Fabrizi" (ZFMK), 1 ♂ "X-DA3332r Italy Sardegna: Villasimius (Camping Spiaggia del Riso) 29°07'23.1'N [sic!; correct: 39°07'23.1''N], 09°30'40.6''E 27.VI.2012 D. Ahrens & S. Fabrizi" (ZFMK), 1 ♂ "X-DA3333b Italy Sardegna: Villasimius (Camping Spiaggia del Riso) 29°07'23.1'N [sic!; correct: 39°07'23.1''N], 09°30'40.6''E 27.VI.2012 D. Ahrens & S. Fabrizi" (ZFMK), 1 ♂ "X-DA3333c Italy Sardegna: Villasimius (Camping Spiaggia del Riso) 29°07'23.1'N [sic!; correct: 39°07'23.1''N], 09°30'40.6''E 27.VI.2012 D. Ahrens & S. Fabrizi" (ZFMK), 1 ♂ "X-DA3333d Italy Sardegna: Villasimius (Camping Spiaggia del Riso) 29°07'23.1'N [sic!; correct: 39°07'23.1''N], 09°30'40.6''E 27.VI.2012 D. Ahrens & S. Fabrizi" (ZFMK), 1 ♂ "X-DA3333e Italy Sardegna: Villasimius (Camping Spiaggia del Riso) 29°07'23.1'N [sic!; correct: 39°07'23.1''N], 09°30'40.6''E 27.VI.2012 D. Ahrens & S. Fabrizi" (ZFMK), 1 ♂ "X-DA3333f Italy Sardegna: Villasimius (Camping Spiaggia del Riso) 29°07'23.1'N [sic!; correct: 39°07'23.1''N], 09°30'40.6''E 27.VI.2012 D. Ahrens & S. Fabrizi" (ZFMK), 1 ♂ "X-DA3333g Italy Sardegna: Villasimius (Camping Spiaggia del Riso) 29°07'23.1'N [sic!; correct: 39°07'23.1''N], 09°30'40.6''E 27.VI.2012 D. Ahrens & S. Fabrizi" (ZFMK), 1 ♂ "X-DA3333h Italy Sardegna: Villasimius (Camping Spiaggia del Riso) 29°07'23.1'N [sic!; correct: 39°07'23.1''N], 09°30'40.6''E 27.VI.2012 D. Ahrens & S. Fabrizi" (ZFMK), 1 ♂ "X-DA3333i Italy Sardegna: Villasimius (Camping Spiaggia del Riso) 29°07'23.1'N [sic!; correct: 39°07'23.1''N], 09°30'40.6''E 27.VI.2012 D. Ahrens & S. Fabrizi" (ZFMK), 1 ♂ "X-DA3333j Italy Sardegna: Villasimius (Camping Spiaggia del Riso) 29°07'23.1'N [sic!; correct: 39°07'23.1''N], 09°30'40.6''E 27.VI.2012 D. Ahrens & S. Fabrizi" (ZFMK), 1 ♂ "X-DA3333k Italy Sardegna: Villasimius (Camping Spiaggia del Riso) 29°07'23.1'N [sic!; correct: 39°07'23.1''N], 09°30'40.6''E 27.VI.2012 D. Ahrens & S. Fabrizi" (ZFMK), 1 ♂ "X-DA3333l Italy Sardegna: Villasimius (Camping Spiaggia del Riso) 29°07'23.1'N [sic!; correct: 39°07'23.1''N], 09°30'40.6''E 27.VI.2012 D. Ahrens & S. Fabrizi" (ZFMK), 1 ♂ "X-DA3333m Italy Sardegna: Villasimius (Camping Spiaggia del Riso) 29°07'23.1'N [sic!; correct: 39°07'23.1''N], 09°30'40.6''E 27.VI.2012 D. Ahrens &

S. Fabrizi" (ZFMK), 1 ♂ "X-DA3333n Italy Sardegna: Villasimius (Camping Spiaggia del Riso) 29°07’23.1’N [sic!; correct: 39°07’23.1”N], 09°30'40.6"E 27.VI.2012 D. Ahrens & S. Fabrizi" (ZFMK), 1 ♂ "X-DA3333o Italy Sardegna: Villasimius (Camping Spiaggia del Riso) 29°07’23.1’N [sic!; correct: 39°07’23.1”N], 09°30'40.6"E 27.VI.2012 D. Ahrens & S. Fabrizi" (ZFMK), 1 ♂ "X-DA3333p Italy Sardegna: Villasimius (Camping Spiaggia del Riso) 29°07’23.1’N [sic!; correct: 39°07’23.1”N], 09°30'40.6"E 27.VI.2012 D. Ahrens & S. Fabrizi" (ZFMK), 1 ♂ "X-DA3333q Italy Sardegna: Villasimius (Camping Spiaggia del Riso) 29°07’23.1’N [sic!; correct: 39°07’23.1”N], 09°30'40.6"E 27.VI.2012 D. Ahrens & S. Fabrizi" (ZFMK), 1 ♂ "X-DA3333r Italy Sardegna: Villasimius (Camping Spiaggia del Riso) 29°07’23.1’N [sic!; correct: 39°07’23.1”N], 09°30'40.6"E 27.VI.2012 D. Ahrens & S. Fabrizi" (ZFMK), 1 ♂ "X-DA3333s Italy Sardegna: Villasimius (Camping Spiaggia del Riso) 29°07’23.1’N [sic!; correct: 39°07’23.1”N], 09°30'40.6"E 27.VI.2012 D. Ahrens & S. Fabrizi" (ZFMK), 1 ♂ "X-DA3333t Italy Sardegna: Villasimius (Camping Spiaggia del Riso) 29°07’23.1’N [sic!; correct: 39°07’23.1”N], 09°30'40.6"E 27.VI.2012 D. Ahrens & S. Fabrizi" (ZFMK), 1 ♂ "X-DA3339A Italy Sardegna: Villasimius (Camping Spiaggia del Riso) 29°07’23.1’N [sic!; correct: 39°07’23.1”N], 09°30'40.6"E 27.6.2012 D. Ahrens & S. Fabrizi" (ZFMK), 1 ♂ "X-DA3339B Italy Sardegna: Villasimius (Camping Spiaggia del Riso) 29°07’23.1’N [sic!; correct: 39°07’23.1”N], 09°30'40.6"E 27.6.2012 D. Ahrens & S. Fabrizi" (ZFMK), 1 ♂ "X-DA3339C Italy Sardegna: Villasimius (Camping Spiaggia del Riso) 29°07’23.1’N [sic!; correct: 39°07’23.1”N], 09°30'40.6"E 27.6.2012 D. Ahrens & S. Fabrizi" (ZFMK), 1 ♂ "X-DA3339D Italy Sardegna: Villasimius (Camping Spiaggia del Riso) 29°07’23.1’N [sic!; correct: 39°07’23.1”N], 09°30'40.6"E 27.6.2012 D. Ahrens & S. Fabrizi" (ZFMK), 1 ♂ "X-DA3339E Italy Sardegna: Villasimius (Camping Spiaggia del Riso) 29°07’23.1’N [sic!; correct: 39°07’23.1”N], 09°30'40.6"E 27.6.2012 D. Ahrens & S. Fabrizi" (ZFMK), 1 ♂ "X-DA3339F Italy Sardegna: Villasimius (Camping Spiaggia del Riso) 29°07’23.1’N [sic!; correct: 39°07’23.1”N], 09°30'40.6"E 27.6.2012 D. Ahrens & S. Fabrizi" (ZFMK), 1 ♂ "X-DA3339G Italy Sardegna: Villasimius (Camping Spiaggia del Riso) 29°07’23.1’N [sic!; correct: 39°07’23.1”N], 09°30'40.6"E 27.6.2012 D. Ahrens & S. Fabrizi" (ZFMK), 1 ♂ "X-DA3339H Italy Sardegna: Villasimius (Camping Spiaggia del Riso) 29°07’23.1’N [sic!; correct: 39°07’23.1”N], 09°30'40.6"E 27.6.2012 D. Ahrens & S. Fabrizi" (ZFMK), 1 ♂ "X-DA3339i Italy Sardegna: Villasimius (Camping Spiaggia del Riso) 29°07’23.1’N [sic!; correct: 39°07’23.1”N], 09°30'40.6"E 27.6.2012 D. Ahrens & S. Fabrizi" (ZFMK), 1 ♂ "X-DA3339J Italy Sardegna: Villasimius (Camping Spiaggia del Riso) 29°07’23.1’N [sic!; correct: 39°07’23.1”N], 09°30'40.6"E 27.6.2012 D. Ahrens & S. Fabrizi" (ZFMK), 1 ♂ "X-DA3339K Italy Sardegna: Villasimius (Camping Spiaggia del Riso) 29°07’23.1’N [sic!; correct: 39°07’23.1”N], 09°30'40.6"E 27.6.2012 D. Ahrens & S. Fabrizi" (ZFMK), 1 ♂ "X-DA3339L Italy Sardegna: Villasimius (Camping Spiaggia del Riso) 29°07’23.1’N [sic!; correct: 39°07’23.1”N], 09°30'40.6"E 27.6.2012 D. Ahrens & S. Fabrizi" (ZFMK), 1 ♂ "X-DA3339M Italy Sardegna: Villasimius (Camping Spiaggia del Riso) 29°07’23.1’N [sic!; correct: 39°07’23.1”N], 09°30'40.6"E 27.6.2012 D. Ahrens & S. Fabrizi" (ZFMK), 1 ♂ "X-DA3339N Italy Sardegna: Villasimius (Camping Spiaggia del Riso) 29°07’23.1’N [sic!; correct: 39°07’23.1”N], 09°30'40.6"E 27.6.2012 D. Ahrens & S. Fabrizi" (ZFMK), 1 ♂ "X-DA3339O Italy Sardegna: Villasimius (Camping Spiaggia del Riso) 29°07’23.1’N [sic!; correct: 39°07’23.1”N], 09°30'40.6"E 27.6.2012 D. Ahrens & S. Fabrizi" (ZFMK), 1 ♂ "X-DA3339P Italy Sardegna: Villasimius (Camping Spiaggia del Riso) 29°07’23.1’N [sic!; correct: 39°07’23.1”N], 09°30'40.6"E 27.6.2012 D. Ahrens & S.

Fabrizi" (ZFMK), 1 ♂ "X-DA3339Q Italy Sardegna: Villasimius (Camping Spiaggia del Riso) 29°07’23.1’N [sic!; correct: 39°07’23.1”N], 09°30'40.6"E 27.6.2012 D. Ahrens & S. Fabrizi" (ZFMK), 1 ♂ "X-DA3339R Italy Sardegna: Villasimius (Camping Spiaggia del Riso) 29°07’23.1’N [sic!; correct: 39°07’23.1”N], 09°30'40.6"E 27.6.2012 D. Ahrens & S. Fabrizi" (ZFMK), 1 ♂ "X-DA3339S Italy Sardegna: Villasimius (Camping Spiaggia del Riso) 29°07’23.1’N [sic!; correct: 39°07’23.1”N], 09°30'40.6"E 27.6.2012 D. Ahrens & S. Fabrizi" (ZFMK), 1 ♂ "X-DA3339t Italy Sardegna: Villasimius (Camping Spiaggia del Riso) 29°07’23.1’N [sic!; correct: 39°07’23.1”N], 09°30'40.6"E 27.6.2012 D. Ahrens & S. Fabrizi" (ZFMK), 1 ♂ "X-DA3339U Italy Sardegna: Villasimius (Camping Spiaggia del Riso) 29°07’23.1’N [sic!; correct: 39°07’23.1”N], 09°30'40.6"E 27.6.2012 D. Ahrens & S. Fabrizi" (ZFMK), 1 ♂ "X-DA3339V Italy Sardegna: Villasimius (Camping Spiaggia del Riso) 29°07’23.1’N [sic!; correct: 39°07’23.1”N], 09°30'40.6"E 27.6.2012 D. Ahrens & S. Fabrizi" (ZFMK), 15 ♂♂ "X-DA3340 Italy Sardegna: Villasimius (Camping Spiaggia del Riso) 29°07’23.1’N [sic!; correct: 39°07’23.1”N], 09°30'40.6"E 27.6.2012 D. Ahrens & S. Fabrizi" (ZFMK), 1 ♂ "X-DA3341A Italy Sardegna: Villasimius (Camping Spiaggia del Riso) 29°07’23.1’N [sic!; correct: 39°07’23.1”N], 09°30'40.6"E 27.6.2012 D. Ahrens & S. Fabrizi" (ZFMK), 1 ♂ "X-DA3341B Italy Sardegna: Villasimius (Camping Spiaggia del Riso) 29°07’23.1’N [sic!; correct: 39°07’23.1”N], 09°30'40.6"E 27.6.2012 D. Ahrens & S. Fabrizi" (ZFMK), 1 ♂ "X-DA3341C Italy Sardegna: Villasimius (Camping Spiaggia del Riso) 29°07’23.1’N [sic!; correct: 39°07’23.1”N], 09°30'40.6"E 27.6.2012 D. Ahrens & S. Fabrizi" (ZFMK), 1 ♂ "X-DA3341D Italy Sardegna: Villasimius (Camping Spiaggia del Riso) 29°07’23.1’N [sic!; correct: 39°07’23.1”N], 09°30'40.6"E 27.6.2012 D. Ahrens & S. Fabrizi" (ZFMK), 1 ♂ "X-DA3341E Italy Sardegna: Villasimius (Camping Spiaggia del Riso) 29°07’23.1’N [sic!; correct: 39°07’23.1”N], 09°30'40.6"E 27.6.2012 D. Ahrens & S. Fabrizi" (ZFMK), 1 ♂ "X-DA3341F Italy Sardegna: Villasimius (Camping Spiaggia del Riso) 29°07’23.1’N [sic!; correct: 39°07’23.1”N], 09°30'40.6"E 27.6.2012 D. Ahrens & S. Fabrizi" (ZFMK), 1 ♂ "X-DA3341G Italy Sardegna: Villasimius (Camping Spiaggia del Riso) 29°07’23.1’N [sic!; correct: 39°07’23.1”N], 09°30'40.6"E 27.6.2012 D. Ahrens & S. Fabrizi" (ZFMK), 1 ♂ "X-DA3341H Italy Sardegna: Villasimius (Camping Spiaggia del Riso) 29°07’23.1’N [sic!; correct: 39°07’23.1”N], 09°30'40.6"E 27.6.2012 D. Ahrens & S. Fabrizi" (ZFMK), 1 ♂ "X-DA3341i Italy Sardegna: Villasimius (Camping Spiaggia del Riso) 29°07’23.1’N [sic!; correct: 39°07’23.1”N], 09°30'40.6"E 27.6.2012 D. Ahrens & S. Fabrizi" (ZFMK), 1 ♂ "X-DA3341J Italy Sardegna: Villasimius (Camping Spiaggia del Riso) 29°07’23.1’N [sic!; correct: 39°07’23.1”N], 09°30'40.6"E 27.6.2012 D. Ahrens & S. Fabrizi" (ZFMK), 1 ♂ "X-DA3341K Italy Sardegna: Villasimius (Camping Spiaggia del Riso) 29°07’23.1’N [sic!; correct: 39°07’23.1”N], 09°30'40.6"E 27.6.2012 D. Ahrens & S. Fabrizi" (ZFMK), 1 ♂ "X-DA3341L Italy Sardegna: Villasimius (Camping Spiaggia del Riso) 29°07’23.1’N [sic!; correct: 39°07’23.1”N], 09°30'40.6"E 27.6.2012 D. Ahrens & S. Fabrizi" (ZFMK), 1 ♂ "X-DA3348 Italy Sardegna: Costa Rei, Piscina Rei env.: Camping "le Dune" 39°16'29.7"N, 09°35'08.2"E 2.7.-4.7.2012 D. Ahrens & S. Fabrizi" (ZFMK), 1 ♂ "X-DA3349 Italy Sardegna: Costa Rei, Piscina Rei env.: Camping "le Dune" 39°16'29.7"N, 09°35'08.2"E 2.7.-4.7.2012 D. Ahrens & S. Fabrizi" (ZFMK), 1 ♂ "X-DA3350 Italy Sardegna: Costa Rei, Piscina Rei env.: Camping "le Dune" 39°16'29.7"N, 09°35'08.2"E 2.7.-4.7.2012 D. Ahrens & S. Fabrizi" (ZFMK), 1 ♂ "X-DA3351 Italy Sardegna: Costa Rei, Piscina Rei env.: Camping "le Dune" 39°16'29.7"N, 09°35'08.2"E 2.7.-4.7.2012 D. Ahrens & S. Fabrizi" (ZFMK), 1 ♂ "X-DA3352 Italy Sardegna: Costa

Rei, Piscina Rei env.: Camping "le Dune" 39°16'29.7"N, 09°35'08.2"E 2.7.-4.7.2012 D. Ahrens & S. Fabrizi" (ZFMK), 1 ♂ "X-DA3353 Italy Sardegna: Costa Rei, Piscina Rei env.: Camping "le Dune" 39°16'29.7"N, 09°35'08.2"E 2.7.-4.7.2012 D. Ahrens & S. Fabrizi" (ZFMK), 1 ♂ "X-DA3969 Italia Sardinia: Marcalagonis (CA) Vill. dei Gigli 15.VI.2009 leg. M.G. Atzori" (ZFMK), 1 ♂ "X-DA4274 Italy Sardegna: Muravera (CA) 5.v.2013 C. Onnis" (ZFMK), 1 ♂ "X-DA4276 Italy Sardegna: Maracalagonis, Torre delle Stelle VII.2013 D. Cillo" (ZFMK), 1 ♂ "X-DA4651 Italy Sardinia: Capo Carbonaria, Loc. Cava Usai (Villasimius, CA) VII.2015" (ZFMK), 1 ♂ "Pachypus sardiniensis Villasimius (CA), Cordolino Blu 18.XI.2017 (VII.2017) + alla luce svuotamento autunnale lampade ristorante da Max/ PP0111" (CCAU), 1 ♂ "Pachypus sardiniensis 1 ♂ Villasimius (CA), Cordolino Blu 18.XI.2017 (VII.2017) + alla luce/ PP0112" (CCAU), 1 ♂ "Pachypus sardiniensis 1 ♂ Villasimius (CA), Cordolino Blu 18.XI.2017 (VII.2017) + alla luce svuotamento autunnale lampade ristorante da Max/ PP0113" (CCAU), 1 ♂ "Pachypus sardiniensis G. B. C., 2013 1 ♂ Villasimius (CA), Pineta bordo strada 06.VII.2014 leg. Ancona C./ PP0114" (CCAU), 1 ♂ "Pachypus sardiniensis G B C, 2013. 1 ♂ Villasimius (CA) Capo Carbonara, Cava Usai 01.VI.2013 I. Ancona/ PP0115" (CCAU), 1 ♂ "Pachypus sardiniensis G B C, 2013. 1 ♂ Villasimius (CA) Cava Usai 01.VI.2013 I. Ancona/ PP0116" (CCAU), 1 ♂ "Pachypus sardiniensis G B C, 2013 Villasimius (CA) Capo Carbonara, Cava Usai 01.VI.2013 I. Ancona/ PP0117" (CCAU), 1 ♂ "Pachypus sardiniensis G B C, 2013 Villasimius (CA) Cava Usai 9.VI.2013/ PP0118" (CCAU), 1 ♂ "Pachypus sardiniensis G. B. C., 2013 1 ♂ Villasimius (CA), Capo Carbonara 22.V.2013/ PP0119" (CCAU), 1 ♂ "Pachypus sardiniensis G. B. C., 2013 1 ♂ Villasimius (CA), Capo Carbonara 22.V.2013/ PP0120" (CCAU), 1 ♂ "Pachypus sardiniensis G. B. C., 2013 1 ♂ Villasimius (CA), Capo Carbonara 14.V.2013/ PP0121" (CCAU), 1 ♂ "Colostrai Muravera CA 1-6-1997 appeso ai fiori / A. Lecis/ PP0190" (CALC), 1 ♂ "Sardegna Castiadas (CA) Piscina Rei 15-VI-1996 leg. D. Atzori/ PP0234" (CMAC), 1 ♂ "Sardegna Castiadas (CA) Piscina Rei 15-VI-1996 leg. D. Atzori/ PP0235" (CMAC), 1 ♂ "Sardegna Muravera (CA) loc. Colostrai 26-VI-1988 leg. M.G. Atzori/ PP0236" (CMAC), 1 ♂ "Sardegna Muravera (CA) Porto Pirastu (Capo Ferrato) 24-VI-2012 leg. M.G. Atzori/ PP0237" (CMAC), 1 ♂ "Sardegna Villasimius (CA) 3-VI-1984 leg. M.G. Atzori/ PP0239" (CMAC), 1 ♂ "Villasimius (Cagliari) 14-VI-1962 leg. F. Cassola/ PP0866" (CGSG), 1 ♂ "Sardegna orientale Muravera, Costa Rei 20.VI.2014 leg. R. Rattu/ PP0876" (CGSG), 1 ♂ "Sardegna (CA) Muravera: Torre Salinas 1/4.7.2003 legit I. Sparacio/ PP1073" (CISP), 1 ♂ "Sardegna (CA) Muravera: Torre Salinas 1/4.7.2003 legit I. Sparacio/ PP1074" (CISP), 1 ♂ "Sardegna (CA) Muravera: Torre Salinas 1/4.7.2003 legit I. Sparacio/ PP1075" (CISP), 1 ♂ "Sardegna (CA) Muravera: Torre Salinas 1/4.7.2003 legit I. Sparacio/ PP1076" (CISP), 1 ♂ "Sardegna (CA) Muravera: Torre Salinas 1/4.7.2003 legit I. Sparacio/ PP1077" (CISP), 1 ♂ "Sardegna (CA) Muravera: Torre Salinas 1/4.7.2003 legit I. Sparacio/ PP1078" (CISP), 1 ♂ "Sardegna Foce Flumendosa 15.VI.84 leg. Canestrelli P./ PP1100" (MSNV), 1 ♂ "Villasimius (CA), Capo Carbonara, VIII.2018 leg. C. Ancona, al suolo/ PP1142" (CCAU), 1 ♂ "Villasimius (CA), Capo Carbonara, VIII.2018 leg. C. Ancona, al suolo/ PP1143" (CCAU), 1 ♂ "Villasimius (CA), Capo Carbonara, VIII.2018 leg. C. Ancona, al suolo/ PP1144" (CCAU), 1 ♂ "Villasimius (CA), Capo Carbonara, 27.V.2018 leg. C. Ancona/ PP1145" (CCAU), 1 ♂ "Villasimius (CA), Capo Carbonara, 27.V.2018 leg. C. Ancona/ PP1146" (CCAU), 1 ♂ "Villasimius (CA), Capo Carbonara, VII.2018 leg. C. Ancona, al suolo/ PP1147" (CCAU), 1 ♂

"Villasimius (CA), Capo Carbonara, 27.V.2018 leg. C. Ancona/ PP1148" (CCAU), 1 ♂ "Villasimius (CA), Capo Carbonara, cava Usai e Stagno Notteri, 20.VI.2017 leg. C. Ancona, al suolo/ PP1149" (CCAU), 1 ♂ "Villasimius (CA), Capo Carbonara, cava Usai e Stagno Notteri, 20.VI.2017 leg. C. Ancona, al suolo/ PP1150" (CCAU), 1 ♂ "Villasimius (CA), Capo Carbonara, cava Usai e Stagno Notteri, 20.VI.2017 leg. C. Ancona, al suolo/ PP1151" (CCAU), 1 ♂ "Maracalagonis Villaggio dei Gigli - CA 15-6-09 leg. M.G. Atzori / A. Lecis/ PP0182" (CALC), 1 ♂ "Maracalagonis Villaggio dei Gigli - CA 15-6-09 leg. M.G. Atzori / A. Lecis/ PP0183" (CALC), 1 ♂ "Maracalagonis Villaggio dei Gigli - CA 15-6-09 leg. M.G. Atzori / A. Lecis/ PP0184" (CALC), 1 ♂ "Villaputzu (CA) Quirra Cala Murtas sulle tamerici retro spiaggia / 17/7/2016 A. Lecis D. Piras G.C. Piras L. Troia / A. Lecis/ PP0191" (CALC), 1 ♂ "Villaputzu (CA) Quirra Cala Murtas sulle tamerici retro spiaggia / 17/7/2016 A. Lecis D. Piras G.C. Piras L. Troia / A. Lecis/ PP0192" (CALC), 1 ♂ "Sardegna (OG-NU) Isola Amm. Arzana Porto Sa Paglia 17-VI-2018 leg. A. Murgioni/ PP0204" (CMAC), 1 ♂ "Sardegna (OG-NU) Isola Amm. Arzana Porto Sa Paglia 17-VI-2018 leg. A. Murgioni/ PP0205" (CMAC), 1 ♂ "Sardegna (OG-NU) Isola Amm. Arzana Porto Sa Paglia 17-VI-2018 leg. A. Murgioni/ PP0206" (CMAC), 1 ♂ "Sardegna (OG-NU) Isola Amm. Arzana Porto Sa Paglia 17-VI-2018 leg. A. Murgioni/ PP0207" (CMAC), 1 ♂ "Sardegna (CA) Maracalagonis Villaggio dei Gigli 20-VI-2009 leg. D. Atzori/ PP0221" (CMAC), 1 ♂ "Sardegna (CA) Maracalagonis Villaggio dei Gigli 20-VI-2009 leg. D. Atzori/ PP0222" (CMAC), 1 ♂ "Sardegna (CA) Maracalagonis Villaggio dei Gigli 20-VI-2009 leg. D. Atzori/ PP0223" (CMAC), 1 ♂ "Sardegna (CA) Maracalagonis Villaggio dei Gigli 15-VI-2009 leg. L. Atzori/ PP0224" (CMAC), 1 ♂ "Sardegna (CA) Maracalagonis Villaggio dei Gigli 15-VI-2009 leg. L. Atzori/ PP0225" (CMAC), 1 ♂ "Sardegna (CA) Maracalagonis Villaggio dei Gigli 10-VI-2009 leg. L. Atzori/ PP0226" (CMAC), 1 ♂ "Sardegna (CA) Maracalagonis Villaggio dei Gigli 10-VI-2009 leg. L. Atzori/ PP0227" (CMAC), 1 ♂ "Sardegna (CA) Maracalagonis Villaggio dei Gigli 14-VI-2009 leg. D. Atzori/ PP0228" (CMAC), 1 ♂ "Sardegna (CA) Maracalagonis Villaggio dei Gigli 02-VI-2008 leg. D. Atzori/ PP0229" (CMAC), 1 ♂ "Sardegna (CA) Maracalagonis Villaggio dei Gigli 06-VII-2008 leg. L. Atzori/ PP0230" (CMAC), 1 ♂ "Sardegna (CA) Maracalagonis Villaggio dei Gigli 27-VI-2012 leg. D. Atzori/ PP0231" (CMAC), 1 ♂ "Sardegna (CA) Maracalagonis Villaggio dei Gigli 27-VI-2012 leg. D. Atzori/ PP0232" (CMAC), 1 ♂ "Sardegna (CA) Maracalagonis Villaggio dei Gigli 27-VI-2012 leg. D. Atzori/ PP0233" (CMAC), 1 ♂ "Sardegna Sinnai (CA) loc. San Basilio S.S. 125 km 24 / 10-VI-2012 leg. A. Murgioni/ PP0238" (CMAC), 1 ♂ "Sardegna (CA) Maracalagonis Villaggio dei Gigli 13.VI.2015 leg. D. Atzori / PP0306" (CMUC), 1 ♂ "Sardegna (CA) Maracalagonis Villaggio dei Gigli 13.VI.2015 leg. D. Atzori / PP0307" (CMUC), 1 ♂ "Sardegna (CA) Maracalagonis Villaggio dei Gigli 10.VII.2015 leg. D. Atzori / Coll. M. Uliana/ PP0308" (CMUC), 1 ♂ "Sardegna Burcei, Rio Ollastu 11.VI.2015 leg. R. Rattu / Coll. M. Uliana/ PP0309" (CMUC), 1 ♂ "Sardegna Burcei, Rio Ollastu 11.VI.2015 leg. R. Rattu / Coll. M. Uliana/ PP0310" (CMUC), 1 ♂ "Sardegna Burcei, Rio Ollastu 11.VI.2015 leg. R. Rattu / Coll. M. Uliana/ PP0311" (CMUC), 1 ♂ "Pachypus sardiniensis Guerlach, Bazzato & Cillo, 2013 det. Uliana, 2015 / Sardegna - Dint. Cagliari Golfo di Carbonara VI.1962 leg. F. Tassi / Coll. M. Uliana/ PP0315" (MSNV), 1 ♂ "Pachypus sardiniensis / Sardegna Maracalagonis 12.VI.2013 leg. E. Bazzato/ PP0451" (CDCC), 1 ♂ "Pachypus sardiniensis / Sardegna Maracalagonis 12.VI.2013 leg. E. Bazzato/ PP0452" (CDCC), 1 ♂ "Pachypus sardiniensis / Sardegna

Maracalagonis 12.VI.2013 leg. E. Bazzato/ PP0453” (CDCC), 1 ♂ "Pachypus sardiniensis / Sardegna Muravera CA 4.VI.2013 leg. C. Onnis/ PP0454” (CDCC), 1 ♂ "Sardegna Mt 7 Fratelli (CA) 8-6-03 leg. A. Spiga / Coll. Spiga/ PP0498” (CDCC), 1 ♂ "Paratypus Pachypus sardiniensis n. sp. G. Guerlach, E. Bazzato & D. Cillo / Italy - Sardegna Maracalagonis Torre delle Stelle 15.VI.2012 leg. D. Cillo/ PP0626” (CDCC), 1 ♂ "Sardegna sud-orientale S. Vito, Rio Ollastu 15.VI.2015, leg. R. Rattu/ PP0860” (CGSG), 1 ♂ "Sardegna sud-orientale S. Vito, Rio Ollastu 15.VI.2015, leg. R. Rattu/ PP0861” (CGSG), 1 ♂ "Sardegna sud-orientale S. Vito, Rio Ollastu 15.VI.2015, leg. R. Rattu/ PP0862” (CGSG), 1 ♂ "Sardegna sud-orient. Burcei, Rio Ollastu 15-17.VI.2014, leg. R. Rattu/ PP0863” (CGSG), 1 ♂ "Sardegna merid. Burcei, Rio Ollastu 24.V.2014, leg. R. Rattu/ PP0864” (CGSG), 1 ♂ "Sardegna sud-orientale S. Vito, Rio Ollastu 15.VI.2015, leg. R. Rattu/ PP0865” (CGSG), 1 ♂ "Sardegna sud-orient. Burcei, Rio Ollastu 15-17.VI.2014, leg. R. Rattu/ PP0867” (CGSG), 1 ♂ "Sardegna sud-orient. Burcei, Rio Ollastu 15-17.VI.2014, leg. R. Rattu/ PP0872” (CGSG), 1 ♂ "Sardegna sud-orient. Burcei, Rio Ollastu 15-17.VI.2014, leg. R. Rattu/ PP0874” (CGSG), 1 ♂ "Sardegna sud-orient. Burcei, Rio Ollastu 15-17.VI.2014, leg. R. Rattu/ PP0877” (CGSG), 1 ♂ "Sardegna merid. Burcei, Rio Ollastu 24.V.2014, leg. R. Rattu/ PP0885” (CGSG), 1 ♂ "Sardegna sud-orient. Quartucciu, Sa Madalena 2.VI.2014, leg. R. Rattu/ PP0887” (CGSG).

Specimens not included in morphometric analysis: (ID based on IUMG): Paratypes: 1 ♂ “ITALY – Sardegna Maracalagonis Torre delle Stelle 15.VI.2012 Leg. D. Cillo” (CDCC).

***Pachypus gallurensis* sp. n.**

Type material examined. <u>(identification based on IUMG</u>**):** Paratypes: 1 ♂ "X-DA3386 Italy Sardegna: Caletta di Osalla (10 km S Orosei), on sand 40°19'48.2"N, 09°40'31.6"E 20.6.2012 D. Ahrens & S. Fabrizi" (ZFMK), 1 ♂ "X-DA3387 Italy Sardegna: Caletta di Osalla (10 km S Orosei), on sand 40°19'48.2"N, 09°40'31.6"E 20.6.2012 D. Ahrens & S. Fabrizi" (ZFMK), 1 ♂ "X-DA3397a Italy Sardegna: Cala Ginepro (Cala Liberotto), N. Orosei 40°26'29.1"N, 09°47'42.6"E (Pachypus, lehmiger Boden) 17.-21.6.2012 D. Ahrens & S. Fabrizi" (ZFMK), 1 ♂ "X-DA3397b Italy Sardegna: Cala Ginepro (Cala Liberotto), N. Orosei 40°26'29.1"N, 09°47'42.6"E (Pachypus, lehmiger Boden) 17.-21.6.2012 D. Ahrens & S. Fabrizi" (ZFMK), 1 ♂ "X-DA3398a Italy Sardegna: Cala Ginepro (Cala Liberotto), N. Orosei 40°26'29.1"N, 09°47'42.6"E (Pachypus, lehmiger Boden) 17.-21.6.2012 D. Ahrens & S. Fabrizi" (ZFMK), 1 ♂ "X-DA3398b Italy Sardegna: Cala Ginepro (Cala Liberotto), N. Orosei 40°26'29.1"N, 09°47'42.6"E (Pachypus, lehmiger Boden) 17.-21.6.2012 D. Ahrens & S. Fabrizi" (ZFMK), 1 ♂ "Sardegna Dorgali (NU) Scala 'e Surtana 28-V-2016 leg. M.G. Atzori/ PP0200” (CMAC), 1 ♂ "Sardegna Dorgali (NU) Scala 'e Surtana 28-V-2016 leg. M.G. Atzori/ PP0201” (CMAC), 1 ♂ "Sardegna Dorgali (NU) Caletta di Osalla 26-VII-1983 leg. D. Atzori/ PP0202” (CMAC), 1 ♂ "Sardegna Dorgali (NU) Caletta di Osalla 26-VII-1983 leg. D. Atzori/ PP0203” (CMAC).

***Pachypus baroniensis* sp. n.**

Type material examined. (identification based on IUMG)**:** Paratypes: 1 ♂ "X-DA4635 Italy Sardinia: San Teodoro, Cala d'Ambra 21-29.VI.2013 G. Carpaneto" (ZFMK), 1 ♂ "X-DA4636 Italy Sardinia: San Teodoro, Cala d'Ambra 21-29.VI.2013 G. Carpaneto" (ZFMK), 1 ♂ "X-DA4637 Italy Sardinia: San Teodoro, Cala d'Ambra 21-29.VI.2013 G. Carpaneto" (ZFMK), 1 ♂ "X-DA4638 Italy Sardinia: San Teodoro, Cala d'Ambra 21-29.VI.2013 G. Carpaneto" (ZFMK), 1 ♂ "X-DA4639 Italy Sardinia: San Teodoro, Cala d'Ambra 21-29.VI.2013 G. Carpaneto" (ZFMK), 1 ♂ "X-DA4640 Italy Sardinia: San Teodoro, Cala d'Ambra 21-29.VI.2013 G. Carpaneto" (ZFMK), 1 ♂ "X-DA4641 Italy Sardinia: San Teodoro, Cala d'Ambra 21-29.VI.2013 G. Carpaneto" (ZFMK), 1 ♂ "X-DA4642 Italy Sardinia: San Teodoro, Cala d'Ambra 21-29.VI.2013 G. Carpaneto" (ZFMK), 1 ♂ "X-DA4643 Italy Sardinia: San Teodoro, Cala d'Ambra 21-29.VI.2013 G. Carpaneto" (ZFMK).
Specimens not included in morphometric analysis (ID based on IUMG): Paratypes: 2 ♂♂ “S. Teodoro (SS) 20 VI 1994 leg. G. Franzini” (CMUC).

***Pachypus occidentalis* sp. n.**

Type material examined. (identification based on IUMG)**: Paratypes:** 1 ♂ "X-DA2609 Italy Sardegna: Valledoria 40.93700N, 8.812998E 2010 S. Fabrizi & D. Ahrens" (ZFMK), 1 ♂ "X-DA2610 Italy Sardegna: Valledoria 40.93700N, 8.812998E 2010 S. Fabrizi & D. Ahrens" (ZFMK), 1 ♂ "X-DA2611 Italy Sardegna: Valledoria 40.93700N, 8.812998E 2010 S. Fabrizi & D. Ahrens" (ZFMK), 1 ♂ "X-DA2612 Italy Sardegna: Valledoria 40.93700N, 8.812998E 2010 S. Fabrizi & D. Ahrens" (ZFMK), 1 ♂ "X-DA2613 Italy Sardegna: Valledoria 40.93700N, 8.812998E 2010 S. Fabrizi & D. Ahrens" (ZFMK), 1 ♂ "X-DA2614 Italy Sardegna: Valledoria 40.93700N, 8.812998E 2010 S. Fabrizi & D. Ahrens" (ZFMK), 1 ♂ "X-DA2615 Italy Sardegna: Valledoria 40.93700N, 8.812998E 2010 S. Fabrizi & D. Ahrens" (ZFMK), 1 ♂ "X-DA2616 Italy Sardegna: Valledoria 40.93700N, 8.812998E 2010 S. Fabrizi & D. Ahrens" (ZFMK), 1 ♂ "X-DA2618 Italy Sardegna: Valledoria 40.93700N, 8.812998E 2010 S. Fabrizi & D. Ahrens" (ZFMK), 1 ♂ "X-DA2619 Italy Sardegna: Valledoria 40.93700N, 8.812998E 2010 S. Fabrizi & D. Ahrens" (ZFMK), 1 ♂ "X-DA2620 Italy Sardegna: Valledoria 40.93700N, 8.812998E 2010 S. Fabrizi & D. Ahrens" (ZFMK), 1 ♂ "X-DA2621 Italy Sardegna: Valledoria 40.93700N, 8.812998E 2010 S. Fabrizi & D. Ahrens" (ZFMK), 1 ♂ "X-DA2622 Italy Sardegna: Valledoria 40.93700N, 8.812998E 2010 S. Fabrizi & D. Ahrens" (ZFMK), 1 ♂ "X-DA2623 Italy Sardegna: Valledoria 40.93700N, 8.812998E 2010 S. Fabrizi & D. Ahrens" (ZFMK), 1 ♂ "X-DA2624 Italy Sardegna: Valledoria 40.93700N, 8.812998E 2010 S. Fabrizi & D. Ahrens" (ZFMK), 1 ♂ "X-DA2625 Italy Sardegna: Valledoria 40.93700N, 8.812998E 2010 S. Fabrizi & D. Ahrens" (ZFMK), 1 ♂ "X-DA2627 Italy Sardegna: Valledoria 40.93700N, 8.812998E 2010 S. Fabrizi & D. Ahrens" (ZFMK), 1 ♂ "X-DA2630 Italy Sardegna: Valledoria 40.93700N, 8.812998E 2010 S. Fabrizi & D. Ahrens" (ZFMK), 1 ♂ "X-DA2631 Italy Sardegna: Valledoria 40.93700N, 8.812998E 2010 S. Fabrizi & D. Ahrens" (ZFMK), 1 ♂ "X-DA2633 Italy Sardegna: Valledoria 40.93700N, 8.812998E 2010 S. Fabrizi & D. Ahrens" (ZFMK), 1 ♂ "X-DA2637 Italy

Sardegna: Valledoria 40.93700N, 8.812998E 2010 S. Fabrizi & D. Ahrens" (ZFMK), 1 ♂ "X-DA2638 Italy Sardegna: Valledoria 40.93700N, 8.812998E 2010 S. Fabrizi & D. Ahrens" (ZFMK), 1 ♂ "X-DA2649 Italy Sardinia: dunes between Torre dei Corsari and Pistis, 40m 39°41'20,0"N; 08°27'25,4"E 5-7.VII.2011 S. Fabrizi & D. Ahrens" (ZFMK), 1 ♂ "X-DA2650 Italy Sardinia: dunes between Torre dei Corsari and Pistis, 40m 39°41'20,0"N; 08°27'25,4"E 5-7.VII.2011 S. Fabrizi & D. Ahrens" (ZFMK), 1 ♂ "X-DA2651 Italy Sardinia: dunes between Torre dei Corsari and Pistis, 40m 39°41'20,0"N; 08°27'25,4"E 5-7.VII.2011 S. Fabrizi & D. Ahrens" (ZFMK), 1 ♂ "X-DA2660 Italy Sardinia: Bosa Marina, 16m 40°16'31,5"N; 08°29'05,3"E 2.VII.2011 S. Fabrizi & D. Ahrens" (ZFMK), 1 ♂ "X-DA2661 Italy Sardinia: Bosa Marina, 16m 40°16'31,5"N; 08°29'05,3"E 2.VII.2011 S. Fabrizi & D. Ahrens" (ZFMK), 1 ♂ "X-DA2665 Italy Sardinia: Is Arenas, 15m 28.VI.-4.VII.2011" (ZFMK), 1 ♂ "X-DA2666 Italy Sardinia: Is Arenas, 15m 28.VI.-4.VII.2011" (ZFMK), 1 ♂ "X-DA2667 Italy Sardinia: Is Arenas, 15m 28.VI.-4.VII.2011" (ZFMK), 1 ♂ "X-DA2668 Italy Sardinia: Is Arenas, 15m 28.VI.-4.VII.2011" (ZFMK), 1 ♂ "X-DA2669 Italy Sardinia: Is Arenas, 15m 28.VI.-4.VII.2011" (ZFMK), 1 # "X-DA2670 Italy Sardinia: Is Arenas, 15m 28.VI.-4.VII.2011" (ZFMK), 1 ♂ "X-DA2671 Italy Sardinia: Is Arenas, 15m 28.VI.-4.VII.2011" (ZFMK), 1 ♂ "X-DA2672 Italy Sardinia: Is Arenas, 15m 28.VI.-4.VII.2011" (ZFMK), 1 ♂ "X-DA2673 Italy Sardinia: Is Arenas, 15m 28.VI.-4.VII.2011" (ZFMK), 1 ♂ "X-DA2674 Italy Sardinia: Is Arenas, 15m 28.VI.-4.VII.2011" (ZFMK), 1 ♂ "X-DA2675 Italy Sardinia: Is Arenas, 15m 28.VI.-4.VII.2011" (ZFMK), 1 ♂ "X-DA2676 Italy Sardinia: Is Arenas, 15m 28.VI.-4.VII.2011" (ZFMK), 1 ♂ "X-DA2677 Italy Sardinia: Is Arenas, 15m 28.VI.-4.VII.2011" (ZFMK), 1 ♂ "X-DA2688 Italy Sardinia: Portixeddu, 0-42m 39°26'29,8"N; 08°25'22,3"E 9.-11.VII.2011 S. Fabrizi & D. Ahrens" (ZFMK), 1 ♂ "X-DA2689 Italy Sardinia: Portixeddu, 0-42m 39°26'29,8"N; 08°25'22,3"E 9.-11.VII.2011 S. Fabrizi & D. Ahrens" (ZFMK), 1 ♂ "X-DA2690 Italy Sardinia: Portixeddu, 0-42m 39°26'29,8"N; 08°25'22,3"E 9.-11.VII.2011 S. Fabrizi & D. Ahrens" (ZFMK), 1 ♂ "X-DA2691 Italy Sardinia: Portixeddu, 0-42m 39°26'29,8"N; 08°25'22,3"E 9.-11.VII.2011 S. Fabrizi & D. Ahrens" (ZFMK), 1 ♂ "X-DA2699 Italy Sardinia: Piscinas (dune), 55m 39°31'33,4"N; 08°28'14,9"E 7.-9.VII.2011" (ZFMK), 1 ♂ "X-DA2700 Italy Sardinia: Piscinas (dune), 55m 39°31'33,4"N; 08°28'14,9"E 7.-9.VII.2011" (ZFMK), 1 ♂ "X-DA2701 Italy Sardinia: Piscinas (dune), 55m 39°31'33,4"N; 08°28'14,9"E 7.-9.VII.2011" (ZFMK), 1 ♂ "X-DA2702 Italy Sardinia: Piscinas (dune), 55m 39°31'33,4"N; 08°28'14,9"E 7.-9.VII.2011" (ZFMK), 1 ♂ "X-DA2703 Italy Sardinia: Piscinas (dune), 55m 39°31'33,4"N; 08°28'14,9"E 7.-9.VII.2011" (ZFMK), 1 ♂ "X-DA2704 Italy Sardinia: Piscinas (dune), 55m 39°31'33,4"N; 08°28'14,9"E 7.-9.VII.2011" (ZFMK), 1 ♂ "X-DA2707 Italy Sardinia: dunes between Torre dei Corsari and Pistis, 40m 39°41'20,0"N; 08°27'25,4"E 5-7.VII.2011 S. Fabrizi & D. Ahrens" (ZFMK), 1 ♂ "X-DA2708 Italy Sardinia: dunes between Torre dei Corsari and Pistis, 40m 39°41'20,0"N; 08°27'25,4"E 5-7.VII.2011 S. Fabrizi & D. Ahrens" (ZFMK), 1 ♂ "X-DA2715 Italy Sardinia: dunes between Torre dei Corsari and Pistis, 40m 39°41'20,0"N; 08°27'25,4"E 5-7.VII.2011 S. Fabrizi & D. Ahrens" (ZFMK), 1 ♂ "X-DA2716 Italy Sardinia: dunes between Torre dei Corsari and Pistis, 40m 39°41'20,0"N; 08°27'25,4"E 5-7.VII.2011 S. Fabrizi & D. Ahrens" (ZFMK), 1 ♂ "X-DA2717 Italy Sardinia: dunes between Torre dei Corsari and Pistis, 40m 39°41'20,0"N; 08°27'25,4"E 5-7.VII.2011 S. Fabrizi & D. Ahrens" (ZFMK), 1 ♂ "X-DA2718 Italy Sardinia: dunes between Torre dei Corsari and Pistis, 40m 39°41'20,0"N; 08°27'25,4"E 5-7.VII.2011 S. Fabrizi & D. Ahrens" (ZFMK), 1 ♂ "X-

DA2720 Italy Sardinia: dunes between Torre dei Corsari and Pistis, 40m 39°41'20,0"N; 08°27'25,4"E 5-7.VII.2011 S. Fabrizi & D. Ahrens" (ZFMK), 1 ♂ "X-DA2721 Italy Sardinia: dunes between Torre dei Corsari and Pistis, 40m 39°41'20,0"N; 08°27'25,4"E 5-7.VII.2011 S. Fabrizi & D. Ahrens" (ZFMK), 1 ♂ "X-DA2722 Italy Sardinia: dunes between Torre dei Corsari and Pistis, 40m 39°41'20,0"N; 08°27'25,4"E 5-7.VII.2011 S. Fabrizi & D. Ahrens" (ZFMK), 1 ♂ "X-DA2723 Italy Sardinia: dunes between Torre dei Corsari and Pistis, 40m 39°41'20,0"N; 08°27'25,4"E 5-7.VII.2011 S. Fabrizi & D. Ahrens" (ZFMK), 1 ♂ "X-DA2733 Italy Sardinia: Piscinas (dune), 55m 39°31'33,4"N; 08°28'14,9"E 7.-9.VII.2011" (ZFMK), 1 ♂ "X-DA2734 Italy Sardinia: Piscinas (dune), 55m 39°31'33,4"N; 08°28'14,9"E 7.-9.VII.2011" (ZFMK), 1 ♂ "X-DA2735 Italy Sardinia: Piscinas (dune), 55m 39°31'33,4"N; 08°28'14,9"E 7.-9.VII.2011" (ZFMK), 1 ♂ "X-DA2736 Italy Sardinia: Piscinas (dune), 55m 39°31'33,4"N; 08°28'14,9"E 7.-9.VII.2011" (ZFMK), 1 ♂ "X-DA2737 Italy Sardinia: Piscinas (dune), 55m 39°31'33,4"N; 08°28'14,9"E 7.-9.VII.2011" (ZFMK), 1 ♂ "X-DA2738 Italy Sardinia: Piscinas (dune), 55m 39°31'33,4"N; 08°28'14,9"E 7.-9.VII.2011" (ZFMK), 1 ♂ "X-DA2741 Italy Sardinia: Piscinas (dune), 55m 39°31'33,4"N; 08°28'14,9"E 7.-9.VII.2011" (ZFMK), 1 ♂ "X-DA2743 Italy Sardinia: Piscinas (dune), 55m 39°31'33,4"N; 08°28'14,9"E 7.-9.VII.2011" (ZFMK), 1 ♂ "X-DA2744 Italy Sardinia: Piscinas (dune), 55m 39°31'33,4"N; 08°28'14,9"E 7.-9.VII.2011" (ZFMK), 1 ♂ "X-DA2745 Italy Sardinia: Piscinas (dune), 55m 39°31'33,4"N; 08°28'14,9"E 7.-9.VII.2011" (ZFMK), 1 ♂ "X-DA2746 Italy Sardinia: Piscinas (dune), 55m 39°31'33,4"N; 08°28'14,9"E 7.-9.VII.2011" (ZFMK), 1 ♂ "X-DA2747 Italy Sardinia: Piscinas (dune), 55m 39°31'33,4"N; 08°28'14,9"E 7.-9.VII.2011" (ZFMK), 1 ♂ "X-DA2748 Italy Sardinia: Piscinas (dune), 55m 39°31'33,4"N; 08°28'14,9"E 7.-9.VII.2011" (ZFMK), 1 ♂ "X-DA2749 Italy Sardinia: Piscinas (dune), 55m 39°31'33,4"N; 08°28'14,9"E 7.-9.VII.2011" (ZFMK), 1 ♂ "X-DA2755 Italy Sardinia: Is Arenas, 15m 28.VI.-4.VII.2011" (ZFMK), 1 ♂ "X-DA2756 Italy Sardinia: Is Arenas, 15m 28.VI.-4.VII.2011" (ZFMK), 1 ♂ "X-DA2757 Italy Sardinia: Is Arenas, 15m 28.VI.-4.VII.2011" (ZFMK), 1 # "X-DA2758 Italy Sardinia: Is Arenas, 15m 28.VI.-4.VII.2011" (ZFMK), 1 ♂ "X-DA2765 Italy Sardinia: Is Arenas, 15m 28.VI.-4.VII.2011" (ZFMK), 1 ♂ "X-DA2766 Italy Sardinia: Is Arenas, 15m 28.VI.-4.VII.2011" (ZFMK), 1 ♂ "X-DA2767 Italy Sardinia: Is Arenas, 15m 28.VI.-4.VII.2011" (ZFMK), 1 ♂ "X-DA2768 Italy Sardinia: Is Arenas, 15m 28.VI.-4.VII.2011" (ZFMK), 1 ♂ "X-DA2769 Italy Sardinia: Is Arenas, 15m 28.VI.-4.VII.2011" (ZFMK), 1 ♂ "X-DA2770 Italy Sardinia: Is Arenas, 15m 28.VI.-4.VII.2011" (ZFMK), 1 ♂ "X-DA2777 Italy Sardinia: Is Arenas, 15m 28.VI.-4.VII.2011" (ZFMK), 1 ♂ "X-DA2780 Italy Sardinia: Is Arenas, 15m 28.VI.-4.VII.2011" (ZFMK), 1 ♂ "X-DA2781 Italy Sardinia: Is Arenas, 15m 28.VI.-4.VII.2011" (ZFMK), 1 ♂ "X-DA2782 Italy Sardinia: dunes between Torre dei Corsari and Pistis, 40m 39°41'20,0"N; 08°27'25,4"E 5-7.VII.2011 S. Fabrizi & D. Ahrens" (ZFMK), 1 ♂ "X-DA2783 Italy Sardinia: dunes between Torre dei Corsari and Pistis, 40m 39°41'20,0"N; 08°27'25,4"E 5-7.VII.2011 S. Fabrizi & D. Ahrens" (ZFMK), 1 ♂ "X-DA2785 Italy Sardinia: dunes between Torre dei Corsari and Pistis, 40m 39°41'20,0"N; 08°27'25,4"E 5-7.VII.2011 S. Fabrizi & D. Ahrens" (ZFMK), 1 ♂ "X-DA2786 Italy Sardinia: dunes between Torre dei Corsari and Pistis, 40m 39°41'20,0"N; 08°27'25,4"E 5-7.VII.2011 S. Fabrizi & D. Ahrens" (ZFMK), 1 ♂ "X-DA2787 Italy Sardinia: dunes between Torre dei Corsari and Pistis, 40m 39°41'20,0"N; 08°27'25,4"E 5-7.VII.2011 S. Fabrizi & D. Ahrens" (ZFMK), 1 ♂ "X-DA2789 Italy Sardinia: dunes between Torre dei Corsari and Pistis, 40m 39°41'20,0"N;

08°27'25,4"E 5-7.VII.2011 S. Fabrizi & D. Ahrens" (ZFMK), 1 ♂ "X-DA2790 Italy Sardinia: dunes between Torre dei Corsari and Pistis, 40m 39°41'20,0"N; 08°27'25,4"E 5-7.VII.2011 S. Fabrizi & D. Ahrens" (ZFMK), 1 ♂ "X-DA2791 Italy Sardinia: dunes between Torre dei Corsari and Pistis, 40m 39°41'20,0"N; 08°27'25,4"E 5-7.VII.2011 S. Fabrizi & D. Ahrens" (ZFMK), 1 ♂ "X-DA2792 Italy Sardinia: dunes between Torre dei Corsari and Pistis, 40m 39°41'20,0"N; 08°27'25,4"E 5-7.VII.2011 S. Fabrizi & D. Ahrens" (ZFMK), 1 ♂ "X-DA2793 Italy Sardinia: dunes between Torre dei Corsari and Pistis, 40m 39°41'20,0"N; 08°27'25,4"E 5-7.VII.2011 S. Fabrizi & D. Ahrens" (ZFMK), 1 ♂ "X-DA2795 Italy Sardinia: dunes between Torre dei Corsari and Pistis, 40m 39°41'20,0"N; 08°27'25,4"E 5-7.VII.2011 S. Fabrizi & D. Ahrens" (ZFMK), 1 ♂ "X-DA2796 Italy Sardinia: dunes between Torre dei Corsari and Pistis, 40m 39°41'20,0"N; 08°27'25,4"E 5-7.VII.2011 S. Fabrizi & D. Ahrens" (ZFMK), 1 ♂ "X-DA2798 Italy Sardinia: dunes between Torre dei Corsari and Pistis, 40m 39°41'20,0"N; 08°27'25,4"E 5-7.VII.2011 S. Fabrizi & D. Ahrens" (ZFMK), 1 ♂ "X-DA2799 Italy Sardinia: dunes between Torre dei Corsari and Pistis, 40m 39°41'20,0"N; 08°27'25,4"E 5-7.VII.2011 S. Fabrizi & D. Ahrens" (ZFMK), 1 ♂ "X-DA2800 Italy Sardinia: dunes between Torre dei Corsari and Pistis, 40m 39°41'20,0"N; 08°27'25,4"E 5-7.VII.2011 S. Fabrizi & D. Ahrens" (ZFMK), 1 ♂ "X-DA2801 Italy Sardinia: dunes between Torre dei Corsari and Pistis, 40m 39°41'20,0"N; 08°27'25,4"E 5-7.VII.2011 S. Fabrizi & D. Ahrens" (ZFMK), 1 ♂ "X-DA2802 Italy Sardinia: dunes between Torre dei Corsari and Pistis, 40m 39°41'20,0"N; 08°27'25,4"E 5-7.VII.2011 S. Fabrizi & D. Ahrens" (ZFMK), 1 ♂ "X-DA2803 Italy Sardinia: dunes between Torre dei Corsari and Pistis, 40m 39°41'20,0"N; 08°27'25,4"E 5-7.VII.2011 S. Fabrizi & D. Ahrens" (ZFMK), 1 ♂ "X-DA2804 Italy Sardinia: dunes between Torre dei Corsari and Pistis, 40m 39°41'20,0"N; 08°27'25,4"E 5-7.VII.2011 S. Fabrizi & D. Ahrens" (ZFMK), 1 ♂ "X-DA2805 Italy Sardinia: dunes between Torre dei Corsari and Pistis, 40m 39°41'20,0"N; 08°27'25,4"E 5-7.VII.2011 S. Fabrizi & D. Ahrens" (ZFMK), 1 ♂ "X-DA2806 Italy Sardinia: dunes between Torre dei Corsari and Pistis, 40m 39°41'20,0"N; 08°27'25,4"E 5-7.VII.2011 S. Fabrizi & D. Ahrens" (ZFMK), 1 ♂ "X-DA2807 Italy Sardinia: dunes between Torre dei Corsari and Pistis, 40m 39°41'20,0"N; 08°27'25,4"E 5-7.VII.2011 S. Fabrizi & D. Ahrens" (ZFMK), 1 ♂ "X-DA2808 Italy Sardinia: dunes between Torre dei Corsari and Pistis, 40m 39°41'20,0"N; 08°27'25,4"E 5-7.VII.2011 S. Fabrizi & D. Ahrens" (ZFMK), 1 ♂ "X-DA2811 Italy Sardinia: Is Arenas, 15m 28.VI.-4.VII.2011 S. Fabrizi & D. Ahrens" (ZFMK), 1 ♂ "X-DA2817 Italy Sardinia: dunes between Torre dei Corsari and Pistis, 40m 39°41'20,0"N; 08°27'25,4"E 5-7.VII.2011 S. Fabrizi & D. Ahrens" (ZFMK), 1 ♂ "X-DA2818 Italy Sardinia: dunes between Torre dei Corsari and Pistis, 40m 39°41'20,0"N; 08°27'25,4"E 5-7.VII.2011 S. Fabrizi & D. Ahrens" (ZFMK), 1 ♂ "X-DA2819 Italy Sardinia: dunes between Torre dei Corsari and Pistis, 40m 39°41'20,0"N; 08°27'25,4"E 5-7.VII.2011 S. Fabrizi & D. Ahrens" (ZFMK), 1 ♂ "X-DA2820 Italy Sardinia: dunes between Torre dei Corsari and Pistis, 40m 39°41'20,0"N; 08°27'25,4"E 5-7.VII.2011 S. Fabrizi & D. Ahrens" (ZFMK), 1 ♂ "X-DA2821 Italy Sardinia: dunes between Torre dei Corsari and Pistis, 40m 39°41'20,0"N; 08°27'25,4"E 5-7.VII.2011 S. Fabrizi & D. Ahrens" (ZFMK), 1 ♂ "X-DA2822 Italy Sardinia: dunes between Torre dei Corsari and Pistis, 40m 39°41'20,0"N; 08°27'25,4"E 5-7.VII.2011 S. Fabrizi & D. Ahrens" (ZFMK), 1 ♂ "X-DA2823 Italy Sardinia: dunes between Torre dei Corsari and Pistis, 40m 39°41'20,0"N; 08°27'25,4"E 5-7.VII.2011 S. Fabrizi & D. Ahrens" (ZFMK), 1 ♂ "X-DA2824 Italy Sardinia: dunes between Torre dei Corsari and Pistis, 40m

39°41'20,0"N; 08°27'25,4"E 5-7.VII.2011 S. Fabrizi & D. Ahrens" (ZFMK), 1 ♂ "X-DA2825 Italy Sardinia: dunes between Torre dei Corsari and Pistis, 40m 39°41'20,0"N; 08°27'25,4"E 5-7.VII.2011 S. Fabrizi & D. Ahrens" (ZFMK), 1 ♂ "X-DA2826 Italy Sardinia: dunes between Torre dei Corsari and Pistis, 40m 39°41'20,0"N; 08°27'25,4"E 5-7.VII.2011 S. Fabrizi & D. Ahrens" (ZFMK), 1 ♂ "X-DA2827 Italy Sardinia: dunes between Torre dei Corsari and Pistis, 40m 39°41'20,0"N; 08°27'25,4"E 5-7.VII.2011 S. Fabrizi & D. Ahrens" (ZFMK), 1 ♂ "X-DA2828 Italy Sardinia: dunes between Torre dei Corsari and Pistis, 40m 39°41'20,0"N; 08°27'25,4"E 5-7.VII.2011 S. Fabrizi & D. Ahrens" (ZFMK), 1 ♂ "X-DA2832 Italy Sardinia: dunes between Torre dei Corsari and Pistis, 40m 39°41'20,0"N; 08°27'25,4"E 5-7.VII.2011 S. Fabrizi & D. Ahrens" (ZFMK), 1 ♂ "X-DA2833 Italy Sardinia: dunes between Torre dei Corsari and Pistis, 40m 39°41'20,0"N; 08°27'25,4"E 5-7.VII.2011 S. Fabrizi & D. Ahrens" (ZFMK), 1 ♂ "X-DA2834 Italy Sardinia: dunes between Torre dei Corsari and Pistis, 40m 39°41'20,0"N; 08°27'25,4"E 5-7.VII.2011 S. Fabrizi & D. Ahrens" (ZFMK), 1 ♂ "X-DA2835 Italy Sardinia: dunes between Torre dei Corsari and Pistis, 40m 39°41'20,0"N; 08°27'25,4"E 5-7.VII.2011 S. Fabrizi & D. Ahrens" (ZFMK), 1 ♂ "X-DA2836 Italy Sardinia: dunes between Torre dei Corsari and Pistis, 40m 39°41'20,0"N; 08°27'25,4"E 5-7.VII.2011 S. Fabrizi & D. Ahrens" (ZFMK), 1 ♂ "X-DA2837 Italy Sardinia: dunes between Torre dei Corsari and Pistis, 40m 39°41'20,0"N; 08°27'25,4"E 5-7.VII.2011 S. Fabrizi & D. Ahrens" (ZFMK), 1 ♂ "X-DA2838 Italy Sardinia: dunes between Torre dei Corsari and Pistis, 40m 39°41'20,0"N; 08°27'25,4"E 5-7.VII.2011 S. Fabrizi & D. Ahrens" (ZFMK), 1 ♂ "X-DA2840 Italy Sardinia: dunes between Torre dei Corsari and Pistis, 40m 39°41'20,0"N; 08°27'25,4"E 5-7.VII.2011 S. Fabrizi & D. Ahrens" (ZFMK), 1 ♂ "X-DA2841 Italy Sardinia: dunes between Torre dei Corsari and Pistis, 40m 39°41'20,0"N; 08°27'25,4"E 5-7.VII.2011 S. Fabrizi & D. Ahrens" (ZFMK), 1 ♂ "X-DA2842 Italy Sardinia: dunes between Torre dei Corsari and Pistis, 40m 39°41'20,0"N; 08°27'25,4"E 5-7.VII.2011 S. Fabrizi & D. Ahrens" (ZFMK), 1 ♂ "X-DA2846 Italy Sardinia: Portixeddu, 0-42m 39°26'29,8"N; 08°25'22,3"E 9.-11.VII.2011 S. Fabrizi & D. Ahrens" (ZFMK), 1 ♂ "X-DA2847 Italy Sardinia: Portixeddu, 0-42m 39°26'29,8"N; 08°25'22,3"E 9.-11.VII.2011 S. Fabrizi & D. Ahrens" (ZFMK), 1 ♂ "X-DA2848 Italy Sardinia: Portixeddu, 0-42m 39°26'29,8"N; 08°25'22,3"E 9.-11.VII.2011 S. Fabrizi & D. Ahrens" (ZFMK), 1 ♂ "X-DA2849 Italy Sardinia: Portixeddu, 0-42m 39°26'29,8"N; 08°25'22,3"E 9.-11.VII.2011 S. Fabrizi & D. Ahrens" (ZFMK), 1 # "X-DA2850 Italy Sardinia: Portixeddu, 0-42m 39°26'29,8"N; 08°25'22,3"E 9.-11.VII.2011 S. Fabrizi & D. Ahrens" (ZFMK), 1 ♂ "X-DA2587 Italy Sardegna: Camping Vignola Mare 41°07'37,05"N, 09°04'23,52"E 2010 S. Fabrizi & D. Ahrens" (ZFMK), 1 ♂ "X-DA2609 Italy Sardegna: Valledoria 40.93700N, 8.812998E 2010 S. Fabrizi & D. Ahrens" (ZFMK), 1 ♂ "X-DA2615 Italy Sardegna: Valledoria 40.93700N, 8.812998E 2010 S. Fabrizi & D. Ahrens" (ZFMK), 1 ♂ "X-DA2616 Italy Sardegna: Valledoria 40.93700N, 8.812998E 2010 S. Fabrizi & D. Ahrens" (ZFMK), 1 ♂ "X-DA2625 Italy Sardegna: Valledoria 40.93700N, 8.812998E 2010 S. Fabrizi & D. Ahrens" (ZFMK), 1 ♂ "X-DA2637 Italy Sardegna: Valledoria 40.93700N, 8.812998E 2010 S. Fabrizi & D. Ahrens" (ZFMK), 1 ♂ "X-DA2638 Italy Sardegna: Valledoria 40.93700N, 8.812998E 2010 S. Fabrizi & D. Ahrens" (ZFMK), 1 ♂ "X-DA4607 Italy Sardinia: Portobello di Gallura, 0,5km E 41.130475N, 9.036141E 1.vii.2016, leg. D. Ahrens & S. Fabrizi" (ZFMK), 1 ♂ "X-DA4608 Italy Sardinia: Portobello di Gallura, 0,5km E 41.130475N, 9.036141E 1.vii.2016, leg. D. Ahrens & S. Fabrizi" (ZFMK), 1 ♂ "X-DA4609 Italy

Sardinia: Costa Paradiso 41.048514N, 8.936935E 2.vii.2016 leg. D. Ahrens & S. Fabrizi" (ZFMK), 1 ♂ "X-DA4622 Italy Sardinia: Vignola Mare, Camping "Saragosa", 1 km E 41.129623N, 9.091657E 29.vi.-1.vii.2016 leg. D. Ahrens & S. Fabrizi" (ZFMK), 1 ♂ "X-DA4623 Italy Sardinia: Vignola Mare, Camping "Saragosa", 1 km E 41.129623N, 9.091657E 29.vi.-1.vii.2016 leg. D. Ahrens & S. Fabrizi" (ZFMK), 1 ♂ "X-DA4624 Italy Sardinia: Vignola Mare, Camping "Saragosa", 1 km E 41.129623N, 9.091657E 29.vi.-1.vii.2016 leg. D. Ahrens & S. Fabrizi" (ZFMK), 1 ♂ "X-DA4626 Italy Sardinia: Vignola Mare, Camping "Saragosa", 1 km E 41.129623N, 9.091657E 29.vi.-1.vii.2016 leg. D. Ahrens & S. Fabrizi" (ZFMK), 1 ♂ "X-DA4629 Italy Sardinia: Vignola Mare, Camping "Saragosa", 1 km E 41.129623N, 9.091657E 29.vi.-1.vii.2016 leg. D. Ahrens & S. Fabrizi" (ZFMK), 1 ♂ "X-DA4630 Italy Sardinia: Vignola Mare, Camping "Saragosa", 1 km E 41.129623N, 9.091657E 29.vi.-1.vii.2016 leg. D. Ahrens & S. Fabrizi" (ZFMK), 1 ♂ "X-DA2627 Italy Sardegna: Valledoria 40.93700N, 8.812998E 2010 S. Fabrizi & D. Ahrens" (ZFMK), 1 ♂ "X-DA4634 Italy Sardinia: Vignola Mare, Camping "Saragosa", 1 km E 41.129623N, 9.091657E 29.vi.-1.vii.2016 leg. D. Ahrens & S. Fabrizi" (ZFMK), 1 ♂ "Pachypus sp. 1 ♂ Torregrande (OR) Foce Tirso Sardegna, Italia 16.VI.2018 C. Ancona leg./ PP0127" (CCAU), 1 ♂ "Pachypus sp. 1 ♂ Torregrande (OR) Foce Tirso Sardegna, Italia 16.VI.2018 C. Ancona leg./ PP0128" (CCAU), 1 ♂ "Pachypus sp. 1 ♂ Torregrande (OR) Foce Tirso Sardegna, Italia 16.VI.2018 C. Ancona leg./ PP0129" (CCAU), 1 ♂ "Pachypus sp. 1 ♂ Torregrande (OR) Foce Tirso Sardegna, Italia 16.VI.2018 C. Ancona leg./ PP0130" (CCAU), 1 ♂ "Pachypus sp. 1 ♂ Torregrande (OR) Foce Tirso Sardegna, Italia 16.VI.2018 C. Ancona leg./ PP0131" (CCAU), 1 ♂ "Pachypus sp. 1 ♂ Torregrande (OR) Foce Tirso Sardegna, Italia 16.VI.2018 C. Ancona leg./ PP0132" (CCAU), 1 ♂ "Pachypus sp. 1 ♂ Torregrande (OR) Foce Tirso Sardegna, Italia 16.VI.2018 C. Ancona leg./ PP0133" (CCAU), 1 ♂ "Pachypus sp. 1 ♂ Torregrande (OR) Foce Tirso Sardegna, Italia 16.VI.2018 C. Ancona leg./ PP0134" (CCAU), 1 ♂ "Pachypus sp. 1 ♂ Torregrande (OR) Foce Tirso Sardegna, Italia 16.VI.2018 C. Ancona leg./ PP0135" (CCAU), 1 ♂ "Pachypus sp. 1 ♂ Torregrande (OR) Foce Tirso Sardegna, Italia 16.VI.2018 C. Ancona leg./ PP0136" (CCAU), 1 ♂ "Torregrande (OR) 3.VII.2015 Alamanni Fed./ PP0179" (CFAQ), 1 ♂ "Sardegna (OR) Torre dei Corsari sand dunes 39.6848, 8.4504 18-24.6.2017 lg. M. Uliana / Coll. M. Uliana/ PP0278" (CMUC), 1 ♂ "Sardegna (OR) Torre dei Corsari sand dunes 39.6848, 8.4504 18-24.6.2017 lg. M. Uliana / PP0279" (CMUC), 1 ♂ "Sardegna (OR) Torre dei Corsari sand dunes 39.6848, 8.4504 18-24.6.2017 lg. M. Uliana / PP0280" (CMUC), 1 ♂ "Sardegna (OR) Torre dei Corsari dunes 39.6848, 8.4504 24.6.2017 h. 07-08.00 am L. M. Uliana / PP0282" (CMUC), 1 ♂ "Sardegna (OR) Torre dei Corsari dunes 39.6848, 8.4504 24.6.2017 h. 07-08.00 am L. M. Uliana / PP0283" (CMUC), 1 ♂ "Sardegna (OR) Torre dei Corsari retroduna a Juniperus, 39.6880, 8.459 UV light 21.6.2017 lg. M. Uliana / PP0284" (CMUC), 1 ♂ "Sardegna (OR) Torre dei Corsari retroduna a Juniperus, 39.6880, 8.459 UV light 21.6.2017 lg. M. Uliana / PP0285" (CMUC), 1 ♂ "Sardegna (OR) Torre dei Corsari retroduna a Juniperus, 39.6880, 8.459 UV light 21.6.2017 lg. M. Uliana / PP0286" (CMUC), 1 ♂ "Sardegna (OR) Torre dei Corsari retroduna a Juniperus, 39.6880, 8.459 UV light 21.6.2017 lg. M. Uliana / PP0287" (CMUC), 1 ♂ "Sardegna (OR) Torre dei Corsari retroduna a Juniperus, 39.6880, 8.459 UV light 21.6.2017 lg. M. Uliana / PP0288" (CMUC), 1 ♂ "Sardegna (OR) Torre dei Corsari sand dunes 39.6848, 8.4504 18-24.6.2017 lg. M. Uliana / PP0289" (CMUC), 1 ♂ "Sardegna (OR) Torre dei Corsari sand

dunes 39.6848, 8.4504 18-24.6.2017 lg. M. Uliana / PP0290" (CMUC), 1 ♂ "Sardegna (OR) Torre dei Corsari sand dunes 39.6848, 8.4504 18-24.6.2017 lg. M. Uliana / PP0291" (CMUC), 1 ♂ "Sardegna (OR) Torre dei Corsari sand dunes 39.6848, 8.4504 18-24.6.2017 lg. M. Uliana / PP0292" (CMUC), 1 ♂ "Sardegna (OR) Torre dei Corsari sand dunes 39.6848, 8.4504 18-24.6.2017 lg. M. Uliana / PP0293" (CMUC), 1 ♂ "Italia - Sardegna Alghero (SS) 27.VI.2012 leg. F. Fois/ PP0455" (CDCC), 1 ♂ "Italia - Sardegna Narbolia (Oristano) Spiaggia Is Arenas 21.VI.2011 D. Cillo leg./ PP0474" (CDCC), 1 ♂ "Italy - Sardegna Tresnuraghes (OR) Porto Alabe 27.VI.2014 leg. D. Cillo/ PP0493" (CDCC), 1 ♂ "Italy - Sardegna Tresnuraghes (OR) Porto Alabe 27.VI.2014 leg. D. Cillo/ PP0494" (CDCC), 1 ♂ "Italy - Sardegna Tresnuraghes (OR) Porto Alabe 27.VI.2014 leg. D. Cillo/ PP0495" (CDCC), 1 ♂ "Italia - Sardegna Narbolia (Oristano) Spiaggia di Is Arenas 5-8.VII.2010 leg. D. Cillo/ PP0513" (CDCC), 1 ♂ "Italia - Sardegna Tresnuraghes Porto Alabe (Oristano) 1.VII.2011 D. Cillo leg./ PP0514" (CDCC), 1 ♂ "Italia - Sardegna Tresnuraghes Porto Alabe (Oristano) 1.VII.2011 D. Cillo leg./ PP0515" (CDCC), 1 ♂ "sp. 5 / Italia - Sardegna Tresnuraghes Porto Alabe (Oristano) 1.VII.2011 D. Cillo leg./ PP0516" (CDCC), 1 ♂ "Torregrande (OR), foce Tirso, strade bianche presso Eucalipteto, m 3 s.l.m., 32S 460730.52 m E, 4416710.37 m N, 28.VI.2018 leg. C. Ancona, al suolo/ PP1140" (CCAU), 1 ♂ "Torregrande (OR), foce fiume Tirso, strada bianca retrodunale, 26.VI.2018 leg. C. Ancona, di sera al suolo/ PP1141" (CCAU), 1 ♂ "Torregrande (OR), foce Tirso, strade bianche presso Eucalipteto, m 3 s.l.m., 32S 460730.52 m E, 4416710.37 m N, 28.VI.2018 leg. C. Ancona, al suolo e in volo al tramonto/ PP1152" (CCAU), 1 ♂ "Torregrande (OR), foce Tirso, strade bianche presso Eucalipteto, m 3 s.l.m., 32S 460730.52 m E, 4416710.37 m N, 28.VI.2018 leg. C. Ancona, al suolo e in volo al tramonto/ PP1153" (CCAU), 1 ♂ "Torregrande (OR), foce Tirso, strade bianche presso Eucalipteto, m 3 s.l.m., 32S 460730.52 m E, 4416710.37 m N, 28.VI.2018 leg. C. Ancona, al suolo e in volo al tramonto/ PP1154" (CCAU), 1 ♂ "Torregrande (OR), foce Tirso, strade bianche presso Eucalipteto, m 3 s.l.m., 32S 460730.52 m E, 4416710.37 m N, 28.VI.2018 leg. C. Ancona, al suolo e in volo al tramonto/ PP1155" (CCAU), 1 ♂ "Torregrande (OR), strada bianca e retroduna, 27.VI.2018 leg. C. Ancona, al suolo/ PP1156" (CCAU), 1 ♂ "Torregrande (OR), strada bianca e retroduna, 27.VI.2018 leg. C. Ancona, al suolo/ PP1157" (CCAU), 1 ♂ "Torregrande (OR), retroduna foce Tirso, 26.VI.2018 leg. C. Ancona, al suolo/ PP1158" (CCAU), 1 ♂ "Torregrande (OR), presso eucalipteto e canneto, 27.VI.2018 leg. C. Ancona, al suolo e al tramonto/ PP1159" (CCAU), 1 ♂ "Pachypus candidae ssp. caesicolor Sardegna La Maddalena VI.1977 leg. Callegari / Coll. MSN Venezia/ PP0271" (MSNV), 1 ♂ "Sardegna (OR) Porto Alabe dunes 19.6.2017 lg. M. Uliana / PP0277" (CMUC), 1 ♂ "Sardegna (OR) S'Ena Arrubia 7.VIII.2013 lg. R. Rattu / PP0281" (CMUC), 1 ♂ "Sardegna Pabillonis Is Arenas IIII.97 / PP0317" (CMUC), 1 ♂ "Sardegna Porto Alabe 21-VI-2008 leg. A. Rattu/ PP0428" (CDCC), 1 ♂ "Italia - Sardegna Tresnuraghes Porto Alabe (Oristano) 01.VII.2011 D. Cillo leg./ PP0429" (CDCC), 1 ♂ "sp. 5 / Sardegna Porto Alabe 22-VI-2008 leg. E. Bazzato/ PP0430" (CDCC), 1 ♂ "Sardegna occidentale Arbus, Scivu 03.VI.2014, leg. R. Rattu"/ PP0871 " (CGSG). **Specimens not included in morphometric analysis** (ID based on IUMG): Paratypes: 3 ♂♂ "Badesi Mare (SS) VIII.2003 Leg. M. Franzini" (CMUC), 3 ♂♂ "I - Badesi (SS) 8-1991 leg. Franzini" (CMUC), 5 ♂♂ "I - Badesi (SS) VIII-1991 leg. Franzini" (CMUC).

***Pachypus pelegrinus* sp. n.**

Type material examined. (identification based on IUMG): 1 ♂ "DNA voucher BMNH 837889/ Italy: Est-Sardegna: 2 km S Santa Maria Navarese, 15.-18.vii.2001, lg. D. Ahrens" (ZFMK), 1 ♂ "X-DA3357 Italy Sardegna: Torre Bari env. (Fiume Manna) Camping Marina 39°50'08.9"N, 09°40'49.9"E 26.6., 6.7.-9.7.2012 D. Ahrens & S. Fabrizi" (ZFMK), 1 ♂ "X-DA3358 Italy Sardegna: Torre Bari env. (Fiume Manna) Camping Marina 39°50'08.9"N, 09°40'49.9"E 26.6., 6.7.-9.7.2012 D. Ahrens & S. Fabrizi" (ZFMK), 1 ♂ "X-DA3359 Italy Sardegna: Torre Bari env. (Fiume Manna) Camping Marina 39°50'08.9"N, 09°40'49.9"E 26.6., 6.7.-9.7.2012 D. Ahrens & S. Fabrizi" (ZFMK), 1 ♂ "X-DA3424c Italy Sardegna: Torre Chia (Camping) 38°53'57.5"N, 08°53'10.0"E 28.6.-1.7.2012 D. Ahrens & S. Fabrizi" (ZFMK), 1 ♂ "X-DA3426a Italy Sardegna: Torre Chia (Camping) 38°53'57.5"N, 08°53'10.0"E 28.vi.-1.vii.2012 D. Ahrens & S. Fabrizi" (ZFMK), 1 ♂ "X-DA3426b Italy Sardegna: Torre Chia (Camping) 38°53'57.5"N, 08°53'10.0"E 28.vi.-1.vii.2012 D. Ahrens & S. Fabrizi" (ZFMK), 1 ♂ "X-DA3426c Italy Sardegna: Torre Chia (Camping) 38°53'57.5"N, 08°53'10.0"E 28.vi.-1.vii.2012 D. Ahrens & S. Fabrizi" (ZFMK), 1 ♂ "X-DA3426d Italy Sardegna: Torre Chia (Camping) 38°53'57.5"N, 08°53'10.0"E 28.vi.-1.vii.2012 D. Ahrens & S. Fabrizi" (ZFMK), 1 ♂ "X-DA3426e Italy Sardegna: Torre Chia (Camping) 38°53'57.5"N, 08°53'10.0"E 28.vi.-1.vii.2012 D. Ahrens & S. Fabrizi" (ZFMK), 1 ♂ "X-DA3426f Italy Sardegna: Torre Chia (Camping) 38°53'57.5"N, 08°53'10.0"E 28.vi.-1.vii.2012 D. Ahrens & S. Fabrizi" (ZFMK), 1 ♂ "X-DA3426g Italy Sardegna: Torre Chia (Camping) 38°53'57.5"N, 08°53'10.0"E 28.vi.-1.vii.2012 D. Ahrens & S. Fabrizi" (ZFMK), 1 ♂ "X-DA3426h Italy Sardegna: Torre Chia (Camping) 38°53'57.5"N, 08°53'10.0"E 28.vi.-1.vii.2012 D. Ahrens & S. Fabrizi" (ZFMK), 1 ♂ "X-DA3426i Italy Sardegna: Torre Chia (Camping) 38°53'57.5"N, 08°53'10.0"E 28.vi.-1.vii.2012 D. Ahrens & S. Fabrizi" (ZFMK), 1 ♂ "X-DA3426j Italy Sardegna: Torre Chia (Camping) 38°53'57.5"N, 08°53'10.0"E 28.vi.-1.vii.2012 D. Ahrens & S. Fabrizi" (ZFMK), 1 ♂ "X-DA3426l Italy Sardegna: Torre Chia (Camping) 38°53'57.5"N, 08°53'10.0"E 28.vi.-1.vii.2012 D. Ahrens & S. Fabrizi" (ZFMK), 1 ♂ "X-DA3426m Italy Sardegna: Torre Chia (Camping) 38°53'57.5"N, 08°53'10.0"E 28.vi.-1.vii.2012 D. Ahrens & S. Fabrizi" (ZFMK), 1 ♂ "X-DA3426n Italy Sardegna: Torre Chia (Camping) 38°53'57.5"N, 08°53'10.0"E 28.vi.-1.vii.2012 D. Ahrens & S. Fabrizi" (ZFMK), 1 ♂ "X-DA3426o Italy Sardegna: Torre Chia (Camping) 38°53'57.5"N, 08°53'10.0"E 28.vi.-1.vii.2012 D. Ahrens & S. Fabrizi" (ZFMK), 1 ♂ "X-DA3426p Italy Sardegna: Torre Chia (Camping) 38°53'57.5"N, 08°53'10.0"E 28.vi.-1.vii.2012 D. Ahrens & S. Fabrizi" (ZFMK), 1 ♂ "X-DA3426q Italy Sardegna: Torre Chia (Camping) 38°53'57.5"N, 08°53'10.0"E 28.vi.-1.vii.2012 D. Ahrens & S. Fabrizi" (ZFMK), 1 ♂ "X-DA3426r Italy Sardegna: Torre Chia (Camping) 38°53'57.5"N, 08°53'10.0"E 28.vi.-1.vii.2012 D. Ahrens & S. Fabrizi" (ZFMK), 1 ♂ "X-DA4656 Italy Sardinia: S. Gregorio, Sinnai (CA) 2.vii.2017 leg. C. Ancona" (ZFMK), 1 ♂ "X-DA4657 Italy Sardinia: S. Gregorio, Sinnai (CA) 2.vii.2017 leg. C. Ancona" (ZFMK), 1 ♂ "X-DA4658 Italy Sardinia: S. Gregorio, Sinnai (CA) 2.vii.2017 leg. C. Ancona" (ZFMK), 1 ♂ "Sardegna (NU) Dorgali 4.7.1993 leg. A. Ceccon / PP0318" (CMUC), 1 ♂ "Sardegna (NU) Dorgali 4.7.1993 leg. A. Ceccon / PP0319" (CMUC), 1 ♂ "Sardegna (NU) Dorgali 4.7.1993 leg. A. Ceccon / PP0320" (CMUC), 1 ♂ "Sardegna (NU) Dorgali 4.7.1993 leg. A. Ceccon /

PP0321” (CMUC), 1 ♂ "Italy Sardegna Dorgali (NU) 5.VII.1993 leg. Ceccon Aurelio / PP0322” (CMUC), 1 ♂ "Sardegna (NU) Cala Gonone 21.VII.91 leg. Agnoli / PP0323” (CMUC), 1 ♂ "Sardegna (CA) San Gregorio (Sinnai) 4.VII.2016 leg. R. Rattu / PP0330” (CMUC), 1 ♂ "Sardinia-merid. San Gregorio (Sinnai)-R. Rattu VII.2016 / PP0331” (CMUC), 1 ♂ "Sardinia-merid. San Gregorio - (Sinnai) - R. Rattu 20.VII.2016 / PP0332” (CMUC), 1 ♂ "Sardegna (CA) Sinnai, San Gregorio 16.7.2016 leg. R. Rattu / PP0333” (CMUC), 1 ♂ "Sardegna (CA) Sinnai, loc. S. Gregorio 30.7.2014 leg. R. Rattu / PP0334” (CMUC), 1 ♂ "Pachypus candidae piero.leo1♂ tiscali.it / Italy - Sardinia Sinnai (Cagliari) San Gregorio 15.VII.2009 leg. P. Leo / PP0335” (CMUC), 1 ♂ "Sardegna (CA) S. Gregorio loc. Sinnai 20.7.2011 leg. R. Rattu / PP0336” (CMUC), 1 ♂ "Sardegna (CA) S. Gregorio loc. Sinnai 20.7.2011 leg. R. Rattu / PP0337” (CMUC), 1 ♂ "Sardegna (CA) Sinnai, S. Gregorio 13.7.2013 leg. R. Rattu / PP0338” (CMUC), 1 ♂ "Sardegna (CA) Sinnai, S. Gregorio 13.7.2013 leg. R. Rattu / PP0339” (CMUC), 1 ♂ "Sardegna Sinnai, San Gregorio 10.VII.2015 leg. R. Rattu / PP0340” (CMUC), 1 ♂ "SE Sardegna Sinnai, San Gregorio 10.VII.2015 leg. R. Rattu / PP0341” (CMUC), 1 ♂ "sp. 4 P. cornutus / Sardegna Barisardo 17-VII-1991 lg. G. Mulas/ PP0422” (CDCC), 1 ♂ "Sardegna Barisardo 18-VII-1991 leg. G. Mulas/ PP0423” (CDCC), 1 ♂ "Sardegna Barisardo 18-VII-1991 leg. G. Mulas/ PP0424” (CDCC), 1 ♂ "Sardegna Dorgali Buchi Arta 12-8-1997 leg. D. Cillo/ PP0425” (CDCC), 1 ♂ "Sardegna Dorgali Buchi Arta 12-8-1997 leg. D. Cillo/ PP0426” (CDCC), 1 ♂ "Dorgali - NU - Cala Gonone Cod. Fuili 8.96 leg. D. Cillo/ PP0502” (CDCC), 1 ♂ "Dorgali - NU - Cala Gonone Cod. Fuili 8.96 leg. D. Cillo/ PP0503” (CDCC), 1 ♂ "Dorgali - NU - Cala Gonone Cod. Fuili 8.96 leg. D. Cillo/ PP0504” (CDCC), 1 ♂ "Dorgali - NU - Cala Gonone Cod. Fuili 8.96 leg. D. Cillo/ PP0505” (CDCC), 1 ♂ "Dorgali - NU - Cala Gonone Cod. Fuili 8.96 leg. D. Cillo/ PP0506” (CDCC), 1 ♂ "Sardegna S. Gregorio 7.08 leg. E. Bazzato/ PP0507” (CEBQ), 1 ♂ "Sardegna S. Gregorio 7.08 leg. E. Bazzato/ PP0508” (CEBQ), 1 ♂ "Sardegna S. Gregorio 7.08 leg. E. Bazzato/ PP0509” (CEBQ), 1 ♂ "Sardegna S. Gregorio 7.08 leg. E. Bazzato/ PP0510” (CEBQ), 1 ♂ "Sardegna S. Gregorio 7.08 leg. E. Bazzato/ PP0511” (CEBQ), 1 ♂ "Sardegna S. Gregorio 7.08 leg. E. Bazzato/ PP0512” (CEBQ), 1 ♂ "Italia - Sardegna Corti Ois 7.09 leg. D. Cillo/ PP0570” (CDCC), 1 ♂ "Italia - Sardegna Corti Ois 7.09 leg. D. Cillo/ PP0571” (CDCC), 1 ♂ "Italy - Sardegna Sinnai (Cagliari) S. Gregorio leg. D. Cillo 30.6.2010/ PP0572” (CDCC), 1 ♂ "Italy - Sardegna Sinnai (Cagliari) Burranca 30-6.2010 leg. D. Cillo/ PP0573” (CDCC), 1 ♂ "Italy - Sardegna Sinnai (Cagliari) S. Gregorio leg. D. Cillo 14.7.2010 S'Incantu/ PP0574” (CDCC), 1 ♂ "Italy - Sardinia Sinnai (Cagliari) S'Incantu 14.VII.2009 D. Cillo/ PP0591” (CDCC), 1 ♂ "Italy - Sardinia Sinnai (Cagliari) S'Incantu 14.VII.2009 D. Cillo/ PP0592” (CDCC), 1 ♂ "Italy - Sardinia Sinnai (Cagliari) S'Incantu 14.VII.2009 D. Cillo/ PP0593” (CDCC), 1 ♂ "Italy - Sardinia Sinnai (Cagliari) S'Incantu 14.VII.2009 D. Cillo/ PP0594” (CDCC), 1 ♂ "Italy - Sardinia Sinnai (Cagliari) S'Incantu 14.VII.2009 D. Cillo/ PP0595” (CDCC), 1 ♂ "Italy - Sardegna Sinnai (Cagliari) S. Gregorio leg. D. Cillo 7.2009/ PP0611” (CDCC), 1 ♂ "Italy - Sardegna Sinnai (Cagliari) S. Gregorio leg. D. Cillo 7.2009/ PP0612” (CDCC), 1 ♂ "Italy - Sardegna Sinnai (Cagliari) S. Gregorio leg. D. Cillo 7.2009/ PP0613” (CDCC), 1 ♂ "Italy - Sardegna Sinnai (Cagliari) S. Gregorio leg. D. Cillo 7.2009/ PP0614” (CDCC), 1 ♂ "Italy - Sardegna Sinnai (Cagliari) Burranca 30.6.2010 leg. D. Cillo/ PP0615” (CDCC), 1 ♂ "Italy - Sardegna Sinnai (Cagliari) S. Gregorio leg. D. Cillo 7.2009/ PP0627” (CDCC), 1 ♂ "Italy - Sardegna Sinnai (Cagliari) S. Gregorio leg. D.

Cillo 7.2009/ PP0628” (CDCC), 1 ♂ "Italy - Sardegna Sinnai (Cagliari) S. Gregorio leg. D. Cillo 7.2009/ PP0629” (CDCC), 1 ♂ "Italy - Sardegna Sinnai (Cagliari) S. Gregorio leg. D. Cillo 7.2009/ PP0630” (CDCC), 1 ♂ "Italy - Sardegna Sinnai (Cagliari) S. Gregorio leg. D. Cillo 24.7.09/ PP0631” (CDCC), 1 ♂ "Italy - Sardegna Sinnai (Cagliari) S. Gregorio leg. D. Cillo 24.7.09/ PP0632” (CDCC), 1 ♂ "Italy - Sardegna Sinnai (Cagliari) S. Gregorio leg. D. Cillo 24.7.09/ PP0633” (CDCC), 1 ♂ "Italy - Sardegna Sinnai (Cagliari) S. Gregorio leg. D. Cillo 24.7.09/ PP0634” (CDCC), 1 ♂ "Italy – Sardegna M.ti Sette Fratelli Sa Corti leg. D. Cillo 7.2009/ PP0635” (CDCC), 1 ♂ "S. Gregorio Sinnai - CA 7.93 leg. D. Cillo/ PP0636” (CDCC), 1 ♂ "S. Gregorio Sinnai - CA 7.93 leg. D. Cillo/ PP0637” (CDCC), 1 ♂ "S. Gregorio Sinnai - CA 7.93 leg. D. Cillo/ PP0638” (CDCC), 1 ♂ "S. Gregorio Sinnai - CA 7.93 leg. D. Cillo/ PP0639” (CDCC), 1 ♂ "S. Gregorio Sinnai - CA 7.93 leg. D. Cillo/ PP0640” (CDCC), 1 ♂ "Italy - Sardegna Sinnai (Cagliari) S. Gregorio leg. D. Cillo 7.2009/ PP0641” (CDCC), 1 ♂ "Italy - Sardegna Sinnai (Cagliari) S. Gregorio leg. D. Cillo 7.2009/ PP0650” (CDCC), 1 ♂ "Italy - Sardegna Sinnai (Cagliari) S. Gregorio leg. D. Cillo 7.2009/ PP0651” (CDCC), 1 ♂ "Italy - Sardegna Sinnai (Cagliari) S. Gregorio leg. D. Cillo 7.2009/ PP0652” (CDCC), 1 ♂ "Italy - Sardegna Sinnai (Cagliari) S. Gregorio leg. D. Cillo 7.2009/ PP0653” (CDCC), 1 ♂ "Italy - Sardegna Sinnai (Cagliari) S. Gregorio leg. D. Cillo 7.2009/ PP0654” (CDCC), 1 ♂ "Italy - Sardegna Sinnai (Cagliari) S. Gregorio leg. D. Cillo 24.7.09/ PP0655” (CDCC), 1 ♂ "Italy - Sardegna Sinnai (Cagliari) S. Gregorio leg. D. Cillo 24.7.09/ PP0656” (CDCC), 1 ♂ "Italy - Sardegna Sinnai (Cagliari) S. Gregorio leg. D. Cillo 24.7.09/ PP0657” (CDCC), 1 ♂ "Italy - Sardegna Sinnai (Cagliari) S. Gregorio leg. D. Cillo 24.7.09/ PP0658” (CDCC), 1 ♂ "Italia - Sardegna Cortiois Sinnai 26.6.08 leg. D. Cillo/ PP0660” (CDCC), 1 ♂ "Italia - Sardegna S. Gregorio Sinnai 6.08 leg. D. Cillo/ PP0661” (CDCC), 1 ♂ "Italia - Sardegna S. Gregorio Sinnai 6.7.08 leg. D. Cillo/ PP0662” (CDCC), 1 ♂ "Italia - Sardegna Cortiois Sinnai 26.6.08 leg. D. Cillo/ PP0663” (CDCC), 1 ♂ "Italia - Sardegna Cortiois Sinnai 26.6.08 leg. D. Cillo/ PP0664” (CDCC), 1 ♂ "Italia - Sardegna Cortiois Sinnai 26.6.08 leg. D. Cillo/ PP0665” (CDCC), 1 ♂ "Italia - Sardegna Cortiois Sinnai 26.6.08 leg. D. Cillo/ PP0666” (CDCC), 1 ♂ "Italia - Sardegna Cortiois Sinnai 26.6.08 leg. D. Cillo/ PP0667” (CDCC), 1 ♂ "Italia - Sardegna Cortiois Sinnai 26.6.08 leg. D. Cillo/ PP0668” (CDCC), 1 ♂ "Italia - Sardegna Cortiois Sinnai 26.6.08 leg. D. Cillo/ PP0669” (CDCC), 1 ♂ "Italia - Sardegna Cortiois Sinnai 26.6.08 leg. D. Cillo/ PP0670” (CDCC), 1 ♂ "Sardegna S. Gregorio CA - 7.07 leg. D. Cillo/ PP0671” (CDCC), 1 ♂ "Sardegna S. Gregorio CA - 7.07 leg. D. Cillo/ PP0672” (CDCC), 1 ♂ "Sardegna S. Gregorio CA - 7.07 leg. D. Cillo/ PP0673” (CDCC), 1 ♂ "Sardegna S. Gregorio CA - 7.07 leg. D. Cillo/ PP0674” (CDCC), 1 ♂ "Sardegna S. Gregorio CA - 7.07 leg. D. Cillo/ PP0675” (CDCC), 1 ♂ "Sardegna S. Gregorio CA - 7.07 leg. D. Cillo/ PP0676” (CDCC), 1 ♂ "Sardegna S. Gregorio CA - 7.07 leg. D. Cillo/ PP0677” (CDCC), 1 ♂ "Sardegna S. Gregorio CA - 7.07 leg. D. Cillo/ PP0678” (CDCC), 1 ♂ "Sardegna S. Gregorio CA - 7.07 leg. D. Cillo/ PP0679” (CDCC), 1 ♂ "Sardegna S. Gregorio CA - 16.7.07 leg. D. Cillo/ PP0680” (CDCC), 1 ♂ "Sardegna S. Gregorio CA - 16.7.07 leg. D. Cillo/ PP0681” (CDCC), 1 ♂ "Italia - Sardegna S. Gregorio CA - 7.09 leg. D. Cillo/ PP0682” (CDCC), 1 ♂ "Italia - Sardegna S. Gregorio CA - 9.7.07 leg. D. Cillo/ PP0683” (CDCC), 1 ♂ "Italia - Sardegna S. Gregorio CA - 9.7.07 leg. D. Cillo/ PP0684” (CDCC), 1 ♂ "Italia - Sardegna S. Gregorio CA - 9.7.07 leg. D. Cillo/ PP0685” (CDCC), 1 ♂ "Italia - Sardegna S. Gregorio CA - 9.7.07 leg. D. Cillo/ PP0686” (CDCC), 1 ♂ "Italia -

Sardegna S. Gregorio CA - 9.7.07 leg. D. Cillo/ PP0687” (CDCC), 1 ♂ "Sardegna S. Gregorio CA - 16.7.07 leg. D. Cillo/ PP0688” (CDCC), 1 ♂ "Sardegna S. Gregorio CA - 16.7.07 leg. D. Cillo/ PP0689” (CDCC), 1 ♂ "Sardegna S. Gregorio CA - 16.7.07 leg. D. Cillo/ PP0690” (CDCC), 1 ♂ "Sardegna S. Gregorio CA - 16.7.07 leg. D. Cillo/ PP0691” (CDCC), 1 ♂ "Sardegna S. Gregorio CA - 16.7.07 leg. D. Cillo/ PP0692” (CDCC), 1 ♂ "Sardegna S. Gregorio CA - 16.7.07 leg. D. Cillo/ PP0693” (CDCC), 1 ♂ "Sardegna S. Gregorio CA - 16.7.07 leg. D. Cillo/ PP0694” (CDCC), 1 ♂ "Sardegna S. Gregorio CA - 16.7.07 leg. D. Cillo/ PP0695” (CDCC), 1 ♂ "Sardegna S. Gregorio CA - 16.7.07 leg. D. Cillo/ PP0696” (CDCC), 1 ♂ "Sardegna S. Gregorio CA - 16.7.07 leg. D. Cillo/ PP0697” (CDCC), 1 ♂ "Sardegna S. Gregorio CA - 16.7.07 leg. D. Cillo/ PP0698” (CDCC), 1 ♂ "Sardegna S. Gregorio CA - 16.7.07 leg. D. Cillo/ PP0699” (CDCC), 1 ♂ "Sardegna S. Gregorio CA - 16.7.07 leg. D. Cillo/ PP0700” (CDCC), 1 ♂ "Sardegna S. Gregorio CA - 16.7.07 leg. D. Cillo/ PP0701” (CDCC), 1 ♂ "Italia - Sardegna S. Gregorio CA - 9.7.07 leg. D. Cillo/ PP0702” (CDCC), 1 ♂ "Italia - Sardegna S. Gregorio CA - 9.7.07 leg. D. Cillo/ PP0703” (CDCC), 1 ♂ "Italia - Sardegna S. Gregorio CA - 9.7.07 leg. D. Cillo/ PP0704” (CDCC), 1 ♂ "Italia - Sardegna S. Gregorio CA - 7.09 leg. D. Cillo/ PP0705” (CDCC), 1 ♂ "Italia - Sardegna S. Gregorio CA - 7.09 leg. D. Cillo/ PP0747” (CDCC), 1 ♂ "Italia - Sardegna S. Gregorio CA - 7.09 leg. D. Cillo/ PP0748” (CDCC), 1 ♂ "Italia - Sardegna S. Gregorio CA - 7.09 leg. D. Cillo/ PP0749” (CDCC), 1 ♂ "Italia - Sardegna S. Gregorio CA - 7.09 leg. D. Cillo/ PP0750” (CDCC), 1 ♂ "San Gregorio Sinnai 7.93 leg. D. Cillo/ PP0776” (CDCC), 1 ♂ "Italia - Sardegna S. Gregorio CA - 9.7.07 leg. D. Cillo/ PP0777” (CDCC), 1 ♂ "Italia - Sardegna S. Gregorio CA - 7.09 leg. D. Cillo/ PP0808” (CDCC), 1 ♂ "Italia - Sardegna S. Gregorio CA - 7.09 leg. D. Cillo/ PP0809” (CDCC), 1 ♂ "Italia - Sardegna S. Gregorio CA - 7.09 leg. D. Cillo/ PP0810” (CDCC), 1 ♂ "Sardegna orient. Sinnai, San Gregorio 12.VII.2014 leg. R. Rattu/ PP0873” (CGSG), 1 ♂ "Sardegna merid. S. Gregorio (Sinnai) 14.VII.2015 leg. R. Rattu/ PP0875” (CGSG), 1 ♂ "Sardegna merid. S. Gregorio (Sinnai) 14.VII.2015 leg. R. Rattu/ PP0878” (CGSG), 1 ♂ "Italy - Sardinia Sinnai (Cagliari) San Gregorio 15.VII.2009 leg. P. Leo/ PP0886” (CGSG), 1 ♂ "Italy - Sardinia Sinnai (Cagliari) San Gregorio 15.VII.2009 leg. P. Leo/ PP0894” (CGSG), 1 ♂ "Marina di Gairo (NU) Cardedu 7-1990 leg. A. Lecis / A. Lecis/ PP0193” (CALC), 1 ♂ "S. Margherita di Pula - CA 10-7-2000 leg. Farris M. / A. Lecis/ PP0194” (CALC), 1 ♂” S. Gregorio Sinnai - CA 16-7-07/ PP0195” (CALC), 1 ♂ "Sardegna Baunei (NU) S. Maria Navarrese 17-VII-1977 leg. M.G. Atzori/ PP0212” (CMAC), 1 ♂ "Sardegna Baunei (NU) S. Maria Navarrese 17-VII-1977 leg. M.G. Atzori/ PP0213” (CMAC), 1 ♂ "Sardegna Baunei (NU) S. Maria Navarrese 18-VII-1977 leg. M.G. Atzori/ PP0214” (CMAC), 1 ♂ "Sardegna Cardedu (NU) Monte Ferru 24-VII-1998 leg. M.G. Atzori/ PP0216” (CMAC), 1 ♂ "Sardegna Bari Sardo (NU) loc. Planargia Pineta Tramalitza / (pomeriggio) 02-VII-2018 leg. M.G. Atzori D. Murgioni/ PP0217” (CMAC), 1 ♂ "Sardegna Bari Sardo (NU) loc. Planargia Pineta Tramalitza / (pomeriggio) 02-VII-2018 leg. M.G. Atzori D. Murgioni/ PP0218” (CMAC), 1 ♂ "Sardegna Sinnai (CA) San Gregorio 02-VII-2007 leg. D. Cillo/ PP0219” (CDCC), 1 ♂ "Sardegna Sinnai (CA) San Gregorio 02-VII-2007 leg. D. Cillo/ PP0220” (CDCC).

***Pachypus cornutus* (Olivier, 1789)**

Additional material examined. <u>(identification based on IUMG)</u>**:** 1 ♂ "X-DA2603 Italy Sardegna: Camping Vignola Mare 41°07'37,05"N, 09°04'23,52"E 2010 S. Fabrizi & D. Ahrens" (ZFMK), 1 ♂ "DA3478 France Corse Isl. 2 km S Porto-Vecchio "Stabiacciu" river, 2.-3.VI.2000, J. Hajek" (NMPC), 1 ♂ "DA3479 France Corse Isl. 2 km S Porto-Vecchio "Stabiacciu" river, 2.-3.VI.2000, J. Hajek" (NMPC), 1 ♂ "DA3480 France Corse Isl. 2 km S Porto-Vecchio "Stabiacciu" river, 2.-3.VI.2000, J. Hajek" (NMPC), 1 ♂ "X-DA4268a France Camping Villata (10 km N Porto Vecchio) (daytime) 41°39.565'N, 009°22.366'E 21.-27.VI.2014 Ahrens & Fabrizi leg." (ZFMK), 1 ♂ "X-DA4268b France Camping Villata (10 km N Porto Vecchio) (daytime) 41°39.565'N, 009°22.366'E 21.-27.VI.2014 Ahrens & Fabrizi leg." (ZFMK), 1 ♂ "X-DA4268c France Camping Villata (10 km N Porto Vecchio) (daytime) 41°39.565'N, 009°22.366'E 21.-27.VI.2014 Ahrens & Fabrizi leg." (ZFMK), 1 ♂ "X-DA4268d France Camping Villata (10 km N Porto Vecchio) (daytime) 41°39.565'N, 009°22.366'E 21.-27.VI.2014 Ahrens & Fabrizi leg." (ZFMK), 1 ♂ "X-DA4269a France Estuary env. of river Liamone, 4 km S Sagone (daytime) 42°05.143'N, 009°04.125'E [sic!] 29.VI.-3.VII.2014 Ahrens & Fabrizi leg." (ZFMK), 1 ♂ "X-DA4269b France Estuary env. of river Liamone, 4 km S Sagone (daytime) 42°05.143'N, 009°04.125'E [sic!] 29.VI.-3.VII.2014 Ahrens & Fabrizi leg." (ZFMK), 1 ♂ "X-DA4269c France Estuary env. of river Liamone, 4 km S Sagone (daytime) 42°05.143'N, 009°04.125'E [sic!] 29.VI.-3.VII.2014 Ahrens & Fabrizi leg." (ZFMK), 1 ♂ "X-DA4270a France Camping Villata (10 km N Porto Vecchio) (daytime) 41°39.565'N, 009°22.366'E 21.-27.VI.2014 Ahrens & Fabrizi leg." (ZFMK), 1 ♂ "X-DA4270b France Camping Villata (10 km N Porto Vecchio) (daytime) 41°39.565'N, 009°22.366'E 21.-27.VI.2014 Ahrens & Fabrizi leg." (ZFMK), 1 ♂ "X-DA4270c France Camping Villata (10 km N Porto Vecchio) (daytime) 41°39.565'N, 009°22.366'E 21.-27.VI.2014 Ahrens & Fabrizi leg." (ZFMK), 1 ♂ "X-DA4271 France Estuary env. of river Liamone, 4 km S Sagone (daytime) 42°05.143'N, 009°04.125'E [sic!] 29.VI.-3.VII.2014 Ahrens & Fabrizi leg." (ZFMK), 1 ♂ "caesus v. impressus / Corse / 9/57, ex coll. R. Oberthür/ PP0010" (ZFMK), 1 ♂ "caesus v. impressus / Corse / 9/57, ex coll. R. Oberthür/ PP0011" (ZFMK), 1 ♂ "candidae Petagna / Corse / 9/57, ex coll. R. Oberthür/ PP0014" (ZFMK), 1 ♂ "candidae Petagna / Corse / 9/57, ex coll. R. Oberthür/ PP0015" (ZFMK), 1 ♂ "candidae Petagna / Corse / 9/57, ex coll. R. Oberthür/ PP0016" (ZFMK), 1 ♂ "candidae Petagna / Corse / 9/57, ex coll. R. Oberthür/ PP0017" (ZFMK), 1 ♂ "candidae Petagna / Corse / 9/57, ex coll. R. Oberthür/ PP0018" (ZFMK), 1 ♂ "candidae Petagna / Corse / 9/57, ex coll. R. Oberthür/ PP0019" (ZFMK), 1 ♂ "candidae Petagna / Corse / 9/57, ex coll. R. Oberthür/ PP0020" (ZFMK), 1 ♂ "candidae Petagna / Corse / 9/57, ex coll. R. Oberthür/ PP0021" (ZFMK), 1 ♂ "candidae Petagna / Corse / 9/57, ex coll. R. Oberthür/ PP0022" (ZFMK), 1 ♂ "candidae Petagna / Corse / 9/57, ex coll. R. Oberthür/ PP0023" (ZFMK), 1 ♂ "candidae Petagna / Corse / 9/57, ex coll. R. Oberthür/ PP0024" (ZFMK), 1 ♂ "candidae Petagna / Corsica / ex coll. Missions - haus SVD Steyl (Eing. N. 3/59)/ PP0025" (ZFMK), 1 ♂ "candidae Petagna / Corsica / ex coll. Missions - haus SVD Steyl (Eing. N. 3/59)/ PP0026" (ZFMK), 1 ♂ "Pachypus candidae Pet. / candidae Petagna / Corsica / det. Dr Balthasar/ PP0027" (ZFMK), 1 ♂ "Pachypus cornutus Oliv. / Corse Mabille / 9/57, ex coll. R. Oberthür/ PP0028" (ZFMK), 1 ♂ "Corse Mabille / 9/57, ex coll. R. Oberthür/ PP0029" (ZFMK), 1 ♂ "P. cornutus Ol. / Dauerleihgabe Fuhlrott-Museum ZFMK Bonn 05/09/ PP0031" (ZFMK), 1 ♂ "cornutus Oliv. / Corse / ex Coll. Marquet via Coll. R. Oberthür ZFMK Bonn/ PP0033" (ZFMK), 1 ♂ "Corse./ PP0036" (ZFMK), 1 ♂ "Pachypus cornutus Oliv. / Corse Damry / Sig. R. Oberthür (Coll. Damry) Eing. Nr. 4,

1956/ PP0038" (ZFMK), 1 ♂ "Corse Damry / Sig. R. Oberthür (Coll. Damry) Eing. Nr. 4, 1956/ PP0039" (ZFMK), 1 ♂ "Corse Damry / Sig. R. Oberthür (Coll. Damry) Eing. Nr. 4, 1956/ PP0040" (ZFMK), 1 ♂ "Corse Damry / Sig. R. Oberthür (Coll. Damry) Eing. Nr. 4, 1956/ PP0041" (ZFMK), 1 ♂ "X-DA2590 Italy Sardegna: Camping Vignola Mare 41°07'37,05''N, 09°04'23,52''E 2010 S. Fabrizi & D. Ahrens" (ZFMK), 1 ♂ "X-DA2591 Italy Sardegna: Camping Vignola Mare 41°07'37,05''N, 09°04'23,52''E 2010 S. Fabrizi & D. Ahrens" (ZFMK), 1 ♂ "X-DA2593 Italy Sardegna: Camping Vignola Mare 41°07'37,05''N, 09°04'23,52''E 2010 S. Fabrizi & D. Ahrens" (ZFMK), 1 ♂ "X-DA2596 Italy Sardegna: Camping Vignola Mare 41°07'37,05''N, 09°04'23,52''E 2010 S. Fabrizi & D. Ahrens" (ZFMK), 1 ♂ "X-DA2599 Italy Sardegna: Camping Vignola Mare 41°07'37,05''N, 09°04'23,52''E 2010 S. Fabrizi & D. Ahrens" (ZFMK), 1 ♂ "X-DA2600 Italy Sardegna: Camping Vignola Mare 41°07'37,05''N, 09°04'23,52''E 2010 S. Fabrizi & D. Ahrens" (ZFMK), 1 ♂ "X-DA2601 Italy Sardegna: Camping Vignola Mare 41°07'37,05''N, 09°04'23,52''E 2010 S. Fabrizi & D. Ahrens" (ZFMK), 1 ♂ "X-DA2602 Italy Sardegna: Camping Vignola Mare 41°07'37,05''N, 09°04'23,52''E 2010 S. Fabrizi & D. Ahrens" (ZFMK), 1 ♂ "X-DA2603 Italy Sardegna: Camping Vignola Mare 41°07'37,05''N, 09°04'23,52''E 2010 S. Fabrizi & D. Ahrens" (ZFMK), 1 ♂ "X-DA2605 Italy Sardegna: Camping Vignola Mare 41°07'37,05''N, 09°04'23,52''E 2010 S. Fabrizi & D. Ahrens" (ZFMK), 1 ♂ "X-DA4610 Italy Sardinia: Camping "La Tortuga" Vignola Mare 41.126376N, 9.065852E 28.vi.2016 leg. D. Ahrens & S. Fabrizi" (ZFMK), 1 ♂ "X-DA4614 Italy Sardinia: Cala vall'alta (pineta) 41.165068N, 9.170645E 5.vii.2016 leg. D. Ahrens & S. Fabrizi" (ZFMK), 1 ♂ "X-DA4618 Italy Sardinia: Vignola Mare, Camping "Saragosa", 1 km E 41.129623N, 9.091657E 29.vi.-1.vii.2016 leg. D. Ahrens & S. Fabrizi" (ZFMK), 1 ♂ "X-DA4619 Italy Sardinia: Vignola Mare, Camping "Saragosa", 1 km E 41.129623N, 9.091657E 29.vi.-1.vii.2016 leg. D. Ahrens & S. Fabrizi" (ZFMK), 1 ♂ "X-DA4620 Italy Sardinia: Vignola Mare, Camping "Saragosa", 1 km E 41.129623N, 9.091657E 29.vi.-1.vii.2016 leg. D. Ahrens & S. Fabrizi" (ZFMK), 1 ♂ "X-DA4621 Italy Sardinia: Vignola Mare, Camping "Saragosa", 1 km E 41.129623N, 9.091657E 29.vi.-1.vii.2016 leg. D. Ahrens & S. Fabrizi" (ZFMK), 1 ♂ "X-DA4625 Italy Sardinia: Vignola Mare, Camping "Saragosa", 1 km E 41.129623N, 9.091657E 29.vi.-1.vii.2016 leg. D. Ahrens & S. Fabrizi" (ZFMK), 1 ♂ "X-DA6974 France: CORSICA, Moriani, 21.VI.2025 Leg. A. Marata" (ZFMK), 1 ♂ "cornutus Er.° Melol. cornuta Oliv. Excavatus Feisth. Corsica. Ramb. / Hist.-Coll (Coleoptera) Nr. 11214 Pachypus cornutus Er.° Corsica, Ramb. Zool. Mus. Berlin / PP0051" (ZMHB), 1 ♂ "Hist.-Coll (Coleoptera) Nr. 11214 Pachypus cornutus Er.* Corsica, Sardinia. Zool. Mus. Berlin/ PP0052" (ZMHB), 1 ♂ "Pachyppus cornutus Erichson / gehört möglicherweise zur historischen Serie Nr. 11214 von P. cornutus Er.°/ noctiluca * Islebia/ PP0053" (ZMHB), 1 ♂ "Corsica / Material der Coll. Moser definitiv nicht von Erichson untersucht/ PP0055" (ZMHB), 1 ♂ "Corsica / Material der Coll. Moser definitiv nicht von Erichson untersucht/ PP0056" (ZMHB), 1 ♂ "Corsica / Material der Coll. Moser definitiv nicht von Erichson untersucht/ PP0057" (ZMHB), 1 ♂ "Pachypus cornutus Oliv. / Corsica Fiuiher/ Material der Coll. Schilsky definitiv nicht von Erichson untersucht/ PP0058" (ZMHB), 1 ♂ "Corsica Ajaccio Dr. J. Schulz / Sammlungsurupming unklar/ PP0059" (ZMHB), 1 ♂ "Hampei i.l. Schauf. Corsica. / Coll. L. W. Schaufuss/ PP0062" (ZMHB), 1 ♂ "Korsika 7.75 / coll. G. Heiland Berlin Spende 2009 / P. indet./ PP0065" (ZMHB), 1 ♂ "Korsika 8.69 / coll. G. Heiland Berlin Spende 2009 / P. indet./ PP0066" (ZMHB), 1 ♂ "P. impressus Er. Corsica / Korsika; Pachypus impressus; Erichson, 1840 Ex. Coll.

Missionshaus Steyl/ PP0251" (ZFMK), 1 ♂ "P. candidus Pet. Corsica / Korsika; Pachypus candidus Muls. Ex. Coll. Missionshaus Steyl/ PP0252" (ZFMK), 1 ♂ "P. cornutus Ol. Corsica / Korsika; Pachypus cornutus; Olivier, 1789 Ex. Coll. Missionshaus Steyl/ PP0253" (ZFMK), 1 ♂ "Korsika; Pachypus cornutus; Olivier, 1789 Ex. Coll. Missionshaus Steyl/ PP0254" (ZFMK), 1 ♂ "Korsika; Pachypus cornutus; Olivier, 1789 Ex. Coll. Missionshaus Steyl/ PP0255" (ZFMK), 1 ♂ "Pachypus candidae 1 ♂ / Cargèse (Corse) - 7 - 61 / Ex coll. Maurice Fenain ZFMK Bonn/ PP0258" (ZFMK), 1 ♂ "20_Monacia 9 - 7 - 76 / Ex coll. Maurice Fenain ZFMK Bonn/ PP0259" (ZFMK), 1 ♂ "20_Palombaggia 6 - 7 -76 / Ex coll. Maurice Fenain ZFMK Bonn/ PP0260" (ZFMK), 1 ♂ "20_Palombaggia 5 - 7 -76 / Ex coll. Maurice Fenain ZFMK Bonn/ PP0261" (ZFMK), 1 ♂ "20_La Trinité de Porto Vecchio 10 - 7 -74 / Ex coll. Maurice Fenain ZFMK Bonn/ PP0262" (ZFMK), 1 ♂ "20_Palombaggia 5 - 7 -76 / Ex coll. Maurice Fenain ZFMK Bonn/ PP0263" (ZFMK), 1 ♂ "20_Palombaggia 6 - 7 -76 / Ex coll. Maurice Fenain ZFMK Bonn/ PP0264" (ZFMK), 1 ♂ "20_La Trinité de Porto Vecchio 10 - 7 -74 / Ex coll. Maurice Fenain ZFMK Bonn/ PP0265" (ZFMK), 1 ♂ "20_Palombaggia 5 - 7 -76 / Ex coll. Maurice Fenain ZFMK Bonn/ PP0266" (ZFMK), 1 ♂ "20_Palombaggia 5 - 7 -76 / Ex coll. Maurice Fenain ZFMK Bonn/ PP0267" (ZFMK), 1 ♂ "20_Palombaggia 5 - 7 -76 / Ex coll. Maurice Fenain ZFMK Bonn/ PP0268" (ZFMK), 1 ♂ "20_Palombaggia 5 - 7 -76 / Ex coll. Maurice Fenain ZFMK Bonn/ PP0269" (ZFMK), 1 ♂ "20_Palombaggia 10 - 7 -76 / Ex coll. Maurice Fenain ZFMK Bonn/ PP0270" (ZFMK), 1 ♂ "Pachypus candidae / Corsika. Dint. Porto. 6.84 lg. Busetto coll. De Giovanni A. / PP0342" (CMUC), 1 ♂ "Pachypus candidae / Francia Palavesa, Porto Vecchio Corse du sud, VII.2012/ PP0427" (CDCC), 1 ♂ "Palavesa, Porto Vecchio Corse du sud, VII.2012/ PP0436" (CDCC), 1 ♂ "Palavesa, Porto Vecchio Corse du sud, VII.2012/ PP0437" (CDCC), 1 ♂ "Palavesa, Porto Vecchio Corse du sud, VII.2012/ PP0438" (CDCC), 1 ♂ "Palavesa, Porto Vecchio Corse du sud, VII.2012/ PP0439" (CDCC), 1 ♂ "Palavesa, Porto Vecchio Corse du sud, VII.2012/ PP0440" (CDCC), 1 ♂ "NO - Korsika - 6.87 Porto Vecchio let. Kleinfeld/ PP0811" (CJMH), 1 ♂ "Corsica - Moriani Loc. Peri (2B) 40 m 14.VI.2017 leg. A. Marata/ PP0817" (CAMM), 1 ♂ "Corsica - Moriani Loc. Peri (2B) 40 m 14.VI.2017 leg. A. Marata/ PP0818" (CAMM), 1 ♂ "Corsica – CORSICA – Roccapina (2A) 4 m 13.VI.2017 leg. A. Marata" (CAMM), 1 ♂ "Corsica, Campo bro Port de Taverna (2B) 2 m 05.VII.2018 leg. A. Marata/ PP0819" (CAMM), 1 ♂ "Corse, West Coast, Travo 23.VII.1989/ PP0891" (CGSG), 1 ♂ "Corse, West Coast, Travo 23.VII.1989/ PP0892" (CGSG), 1 ♂ "F: Corse du Sud Bonifacio - Capo di Feno 20.VI.1994 - Colonnelli/ PP0899" (CGSG), 1 ♂ "F: Corse du Sud Bonifacio - Capo di Feno 20.VI.1994 - Colonnelli/ PP0900" (CGSG), 1 ♂ "FR - Corsica - St. Florent 5.VI.1975, leg. Tagliaferri/ PP0901" (CGSG), 1 ♂ "cornutus Burm. Corse / Coll. R. I. Sc. N. B. France / ex coll. R.P. David ex coll. Pères Jésuites (Le Moult vendit)/ PP0928" (ISNB), 1 ♂ "A. Janssens det., 1941: candidae Petagna. / Corse Campo dell Oro (Marais) G. Bènard. capt / Coll. R. I. Sc. N. B. France / Juin/ PP0929" (ISNB), 1 ♂ "Pachypus. L.atr. cornutus Ol. Corse. / Coll. R. I. Sc. N. B. France Corse coll. de Bonneuil Le Moult vendit/ PP0930" (ISNB), 1 ♂ "Coll. R. I. Sc. N. B. France Corse Coll. Madon/ PP0931" (ISNB), 1 ♂ "Coll. R. I. Sc. N. B. France Corse coll. P. Madon Le Moult vendit / 1 ♂ / PP0932" (ISNB), 1 ♂ "Coll. R. I. Sc. N. B. France Corse Ajaccio coll. Le Moult Juin / 1 ♂ / PP0933" (ISNB), 1 ♂ "Corse / ex coll. R.P. David ex coll. Pères Jésuites (Le Moult vendit)/ PP0934" (ISNB), 1 ♂ "Corse / Coll. R. I. Sc. N. B. France / coll. Mesmin / coll. Vitalia de Salvaza (Le Moult vendit)/ PP0935" (ISNB), 1 ♂ "Pachypus candidae Petagna / Corse. / Coll. R. I. Sc. N. B. France / coll. Mesmin / coll.

Vitalia de Salvaza (Le Moult vendit)/ PP0936" (ISNB), 1 ♂ "Pachypus candidae / Omessa Corse / Coll. R. I. Sc. N. B. France / R.I.Sc.N.B. I.G. 18.694 Coll. Steinmetz / Pachypus candidae (Pet.) D. Keith det 03/ PP0937" (ISNB), 1 ♂ "Corse / Coll. R. I. Sc. N. B. France/ PP0939" (ISNB), 1 ♂ "Pachypus cornutus Corse / Coll. R. I. Sc. N. B. France / Collection E. Candèze/ PP0940" (ISNB), 1 ♂ "Corse Campo dell Oro (Marais) G. Bènard. capt / Coll. R. I. Sc. N. B. France / Juin/ PP0942" (ISNB), 1 ♂ "Pachypus candidae var. noire / Coll. R. I. Sc. N. B. France Corse Ajaccio coll. de Bonneuil Le Moult vendit/ PP0943" (ISNB), 1 ♂ "Corse Campo dell Oro (Marais) G. Bènard. Capt / Coll. R. I. Sc. N. B. France/ PP0944" (ISNB), 1 ♂ "Corse Damry / Coll. R. I. Sc. N. B. France / M.R. Belg. / Coll. Camille Van Voixem. / 316 / 65 / PP0945" (ISNB), 1 ♂ "Corse / Coll. R. I. Sc. N. B. France / Desbrochers / Coll. de Borre / 316/ PP0947" (ISNB), 1 ♂ "Corse / Coll. R. I. Sc. N. B. France / ex coll. Sirguey Le Moult vend.:/ PP0948" (ISNB), 1 ♂ "Pachypus candidae 1 ♂ Corse / Coll. R. I. Sc. N. B. France / ex coll. Sirguey Le Moult vend.:/ PP0949" (ISNB), 1 ♂ "Pachypus impressus Er. / Corse / Coll. R. I. Sc. N. B. France/ PP0950" (ISNB), 1 ♂ "Corse / Coll. R. I. Sc. N. B. France / Sédillot/ PP0951" (ISNB), 1 ♂ "Corse Ajaccio coll. Le Moult / Juilet / Coll. R. I. Sc. N. B. France/ PP0952" (ISNB), 1 ♂ "Corse Ajaccio coll. Le Moult / Juin / Coll. R. I. Sc. N. B. France/ PP0953" (ISNB), 1 ♂ "Corse Ajaccio coll. Le Moult / Juilet / Coll. R. I. Sc. N. B. France/ PP0954" (ISNB), 1 ♂ "Corse Ajaccio coll. Le Moult / Juilet / Coll. R. I. Sc. N. B. France/ PP0955" (ISNB), 1 ♂ "Corse Ajaccio coll. Le Moult / Juin / Coll. R. I. Sc. N. B. France/ PP0956" (ISNB), 1 ♂ "Portigliano Corse Juin 1983 / Coll. R. I. Sc. N. B. ex coll J.M. Warlet I.G. 31.513/ PP0959" (ISNB), 1 ♂ "Ajaccio Corse Juin 1983 / Coll. R. I. Sc. N. B. ex coll J.M. Warlet I.G. 31.513/ PP0960" (ISNB), 1 ♂ "Petreto Bichissiano Corse 6.83 / Coll. R. I. Sc. N. B. ex coll J.M. Warlet I.G. 31.513/ PP0961" (ISNB), 1 ♂ "Portigliano Corse Juin 1983 / Coll. R. I. Sc. N. B. ex coll J.M. Warlet I.G. 31.513/ PP0962" (ISNB), 1 ♂ "Propriano Corse Juin 1983 / Coll. R. I. Sc. N. B. ex coll J.M. Warlet I.G. 31.513/ PP0963" (ISNB), 1 ♂ "Propriano Corse Juin 1983 / Coll. R. I. Sc. N. B. ex coll J.M. Warlet I.G. 31.513/ PP0964" (ISNB), 1 ♂ "Propriano Corse Juin 1983 / Coll. R. I. Sc. N. B. ex coll J.M. Warlet I.G. 31.513/ PP0965" (ISNB), 1 ♂ "Propriano Corse Juin 1983 / Coll. R. I. Sc. N. B. ex coll J.M. Warlet I.G. 31.513/ PP0966" (ISNB), 1 ♂ "Monte Rosso Corse Juin 1983 / Coll. R. I. Sc. N. B. ex coll J.M. Warlet I.G. 31.513/ PP0967" (ISNB), 1 ♂ "Propriano Corse Juin 1983 / Coll. R. I. Sc. N. B. ex coll J.M. Warlet I.G. 31.513/ PP0968" (ISNB), 1 ♂ "Pachypus candidae / Portigliano Corse Juin 1983 / Coll. R. I. Sc. N. B. ex coll J.M. Warlet I.G. 31.513/ PP0969" (ISNB), 1 ♂ "Portigliano Corse Juin 1983 / Coll. R. I. Sc. N. B. ex coll J.M. Warlet I.G. 31.513/ PP0970" (ISNB), 1 ♂ "Pitrera, Trinité Porto Vecchio 7-2000 S. Corse, France leg. E. Jiroux col. E. Jiroux / Collection A. Drumont/ PP0971" (ISNB), 1 ♂ "Vizzavona, Corse V.2001 leg. F. Drumont / Collection A. Drumont / Photo N. Mal 2015/ PP0972" (ISNB), 1 ♂ "env. St. Florent, Corse V.1997 / Collection A. Drumont/ PP0973" (ISNB), 1 ♂ "Marché de Bonifacio, Corse, France 27/VI/2007 leg. F. Drumont / Collection A. Drumont / Photo N. Mal 2015/ PP0974" (ISNB), 1 ♂ "Pitrera, Trinité Pto Vecchio, Corse 7.2000 col. E. Jiroux / Collection A. Drumont/ PP0975" (ISNB), 1 ♂ "Corse./ PP0976" (MHNG), 1 ♂ "Corse Ajaccio/ PP0977" (MHNG), 1 ♂ "Corse./ PP0978" (MHNG), 1 ♂ "Corse./ PP0979" (MHNG), 1 ♂ "Corse Ajaccio/ PP0980" (MHNG), 1 ♂ "Corse Ajaccio/ PP0981" (MHNG), 1 ♂ "Pachypus candidae Pet. / Corse/ PP0982" (MHNG), 1 ♂ "Pachypus candidae Pet. / Corse / Coll. Jacques Rappo / Coll. Jacques Rappo/ PP0984" (MHNG), 1 ♂ "Museum Paris Corse env. d'Ajaccio Vallée du Cavallo Morto G. Bernard capt E.G. Dehaut 1910 / Juin/ PP0985"

(MHNG), 1 ♂ "Museum Paris Corse env. d'Ajaccio Carobàccia G. Bernard capt E.G. Dehaut 1910 / 27 Mai 1910/ PP0986" (MHNG), 1 ♂ "Corse Cargèse 20.VI.73 leg. I. Sieber/ PP0987" (MHNG), 1 ♂ "Pachypus candidae Pet. / Corse coll. De Vauloger / Coll. Jacques Rappo / Coll. Jacques Rappo/ PP0988" (MHNG), 1 ♂ "Pachypus candidae Pet. / Corse Ajaccio coll. Le Moult / Juin / Coll. Jacques Rappo / Coll. Jacques Rappo/ PP0989" (MHNG), 1 ♂ "Pachypus candidae Pet. / Corse. / Coll. Jacques Rappo / Coll. Jacques Rappo/ PP0990" (MHNG), 1 ♂ "France Corse Ostriconi 1-9.VII.1976 Löbl/ PP0991" (MHNG), 1 ♂ "France Corse Ostriconi 1-9.VII.1976 Löbl/ PP0992" (MHNG), 1 ♂ "Pachypus cornutus var. candidae. 272. / Corse Damry / 272/ PP0993" (MHNG), 1 ♂ "Corse Ajaccio coll. Le Moult / Juilet/ PP0994" (MHNG), 1 ♂ "Corse Ajaccio coll. Le Moult / Juin/ PP0995" (MHNG), 1 ♂ "Corse Ajaccio coll. Le Moult / Juin/ PP0996" (MHNG), 1 ♂ "Corse Ajaccio coll. Le Moult / Juin/ PP0997" (MHNG), 1 ♂ "Corse./ PP0998" (MHNG), 1 ♂ "Corse Ajaccio/ PP0999" (MHNG), 1 ♂ "Corse Ajaccio/ PP1000" (MHNG), 1 ♂ "Corse Ajaccio coll. Le Moult / Juin/ PP1001" (MHNG), 1 ♂ "Corse coll. De Vauloger/ PP1002" (MHNG), 1 ♂ "Corse Ajaccio/ PP1003" (MHNG), 1 ♂ "Pachypus candidae Pet. / Corse Ajaccio / Coll. Jacques Rappo/ PP1004" (MHNG), 1 ♂ "Corse coll. De Vauloger/ PP1005" (MHNG), 1 ♂ "Corse./ PP1006" (MHNG), 1 ♂ "Corse Vizzavona coll. Le Moult/ PP1008" (MHNG), 1 ♂ "Corse Vizzavona coll. Le Moult / Juin/ PP1009" (MHNG), 1 ♂ "Corse Vizzavona coll. Le Moult / Juin/ PP1010" (MHNG), 1 ♂ "Pachypus cornutus. V70'/ Corse Damry / 209/ PP1011" (MHNG), 1 ♂ "cornutus Corsica. Srnaka. / Coll. Petrovitz/ PP1014" (MHNG), 1 ♂ "Corse Ajaccio / Coll. Petrovitz/ PP1016" (MHNG), 1 ♂ "Type. / Corse Damry/ PP1017" (MHNG), 1 ♂ "excavatus F. Corse Chiesi/ PP1018" (MHNG), 1 ♂ "Corsica (Nord) Foce Ostriconi 12.VII.1993 leg. L. Bonometto/ PP1084" (MSNV), 1 ♂ "Corsica Bonifacio dint. 21-7-83 % leg. Bonometto L./ PP1085" (MSNV), 1 ♂ "Pachypus candidae (Pet.) ssp. ? Det. E. Ratti, 1991 / Corsica Desert des Agriates spiaggia 2.VIII.1991 leg. L. Bonometto / Corsica Désert des Agriates, anse l'Acciola 2-8-1991 leg. L. Bonometto/ PP1086" (MSNV), 1 ♂ "Corsica (Nord) Desert des Agriates spiaggia 2.VIII.1991 leg. L. Bonometto / Corsica Désert des Agriates, anse l'Acciola 2-8-1991 leg. L. Bonometto/ PP1087" (MSNV), 1 ♂ "Corsica (Nord) Desert des Agriates spiaggia leg. L. Bonometto / Corsica Désert des Agriates, anse l'Acciola 20-VII-1992 leg. L. Bonometto/ PP1088" (MSNV), 1 ♂ "Corsica (Nord) Desert des Agriates spiaggia 2.VIII.1991 leg. L. Bonometto/ PP1089" (MSNV), 1 ♂ "Corsica (Nord) Désert des Agriates, Foce Ostriconi 20-6-2003 leg. L. Bonometto/ PP1090" (MSNV), 1 ♂ "Pachypus cornutus Oliv. Corsica / Corsica/ PP1094" (MSNV), 1 ♂ "Corsica Désert des Agriates, Foce Ostriconi 20-6-2003 leg. L. Bonometto / Rilievi aridi/ PP1095" (MSNV), 1 ♂ "Corsica Désert des Agriates, Foce Ostriconi 20-6-2003 leg. L. Bonometto / Pascolo/ PP1096" (MSNV), 1 ♂ "Corsica (Nord) Desert des Agriates / Baia di Acciola 13.VII.1993 leg. L. Bonometto/ PP1097" (MSNV), 1 ♂ "Corsica Désert des Agriates, Foce Ostriconi 26-6-2005 leg. L. Bonometto / Rilievi aridi/ PP1098" (MSNV), 1 ♂ "Sardegna Arzachena (SS) 06-VII-1986 leg. M.G. Atzori/ PP0215" (CMAC).

Specimens not included in morphometric analysis (ID based on IUMG): 6 ♂♂ "CORSICA 7-12.7.2019 Ghisonaccia 42.016, 9.460 Leg. V. Gallerati" (CVGS), 1 ♂ "CORSICA 9.VII.2019 Ghisonaccia 42.016, 9.460 Leg. V. Gallerati" (CVGS), 1 ♂ "CORSICA 8.VII.2019 Ghisonaccia 42.016, 9.460 Leg. V. Gallerati" (CVGS), 1 ♂ "CORSICA 10.VII.2019 Val Solenzara, Ponte Grossu 41.835, 9.320 Leg. V. Gallerati" (CVGS), 4 ♂♂ "CORSICA 11.7.2019 S of Sartene, Paddaghiu 41.534, 8.918 Leg. V.

Gallerati/” (CMUC), 3 ♂♂ “CORSICA 11.7.2019 S of Sartene, Paddaghiu 41.534, 8.918 Leg. V. Gallerati” (CVGS), 1 ♂ “CORSICA Corte, 8.VII.2019 42.286, 9.198 Leg. V. Gallerati” (CVGS), 1 ♂ “CORSICA Chisa, VII.2019 41.923, 9.260 Leg. V. Gallerati” (CVGS).

Pachypus franginii **sp. n.**

Type material examined. (identification based on IUMG): Paratypes: 1 ♂ "X-DA4668 Italy: Elba, Norsi beach 42.765231N. 10.342743E, 18.vi.2019 leg. Ahrens & Fabrizi/ X-DA4668" (ZFMK), 1 ♂ "Italia Toscana Isola d'Elba, Norsi 42.7700, 10.3461 7.VII.2013 lg. L. Forbicioni / PP0343" (CMUC), 1 ♂ "Italia Toscana Isola d'Elba, Norsi 42.7700, 10.3461 10.VII.2013 lg. L. Forbicioni / Aedeagus not dissected / PP0344" (CMUC), 1 ♂ "Italia Toscana Isola d'Elba, Norsi 42.7700, 10.3461 10.VII.2013 lg. L. Forbicioni / Aedeagus not dissected / PP0345" (CMUC), 1 ♂ "Italia Toscana Isola d'Elba, Norsi 42.7700, 10.3461 10.VII.2013 lg. L. Forbicioni / Aedeagus not dissected / PP0346" (CMUC), 1 ♂ "Italia Toscana Isola d'Elba, Norsi 42.7700, 10.3461 10.VII.2013 lg. L. Forbicioni / PP0347" (CMUC), 1 ♂ "Italia Toscana Isola d'Elba, Norsi 42.7700, 10.3461 7.VII.2013 lg. L. Forbicioni / PP0348" (CMUC), 1 ♂ "Italia Toscana 24.VI.2015 Isola d'Elba, Capoliveri, Calanchiole, Isola d'Elba leg. L. Forbicioni / PP0349" (CMUC), 1 ♂ "Italia Toscana 24.VI.2015 Isola d'Elba, Capoliveri, Calanchiole, Isola d'Elba leg. L. Forbicioni / PP0350" (CMUC), 1 ♂ "Italia Toscana 24.VI.2015 Isola d'Elba, Capoliveri, Calanchiole, Isola d'Elba leg. L. Forbicioni / PP0351" (CMUC), 1 ♂ "Italia Toscana Isola d'Elba, Norsi 42.7700, 10.3461 10.VII.2013 lg. L. Forbicioni / PP0355" (CMUC), 1 ♂ "Italia Toscana Isola d'Elba, Norsi 42.7700, 10.3461 10.VII.2013 lg. L. Forbicioni / PP0356" (CMUC), 1 ♂ "Italia Toscana Isola d'Elba, Norsi 42.7700, 10.3461 10.VII.2013 lg. L. Forbicioni / Aedeagus not dissected / PP0357" (CMUC), 1 ♂ "Italia Toscana Isola d'Elba, Norsi 42.7700, 10.3461 10.VII.2013 lg. L. Forbicioni / Aedeagus not dissected / PP0358" (CMUC), 1 ♂ "Italia Toscana Isola d'Elba, Norsi 42.7700, 10.3461 4.VII.2013 lg. L. Forbicioni / PP0360" (CMUC), 1 ♂ "Italia Toscana Isola d'Elba, Norsi 42.7700, 10.3461 4.VII.2013 lg. L. Forbicioni / PP0361" (CMUC), 1 ♂ "Italia - Toscana Isola d'Elba Capoliveri, loc. Norsi 14.6.2015 leg. L. Forbicioni / PP0363" (CMUC), 1 ♂ "Italia - Toscana Isola d'Elba Capoliveri, loc. Norsi 14.6.2015 leg. L. Forbicioni / PP0364" (CMUC), 1 ♂ "Italia - Toscana Isola d'Elba Capoliveri, loc. Norsi 14.6.2015 leg. L. Forbicioni / PP0365" (CMUC), 1 ♂ "Italia Toscana Isola d'Elba, Norsi 42.7700, 10.3461 4.VII.2013 lg. L. Forbicioni / Aedeagus not dissected / PP0366" (CMUC), 1 ♂ "Italia Toscana Isola d'Elba, Norsi 42.7700, 10.3461 4.VII.2013 lg. L. Forbicioni / Aedeagus not dissected / PP0367" (CMUC), 1 ♂ "Italia Toscana Isola d'Elba, Norsi 42.7700, 10.3461 10.VII.2013 lg. L. Forbicioni / Aedeagus not dissected / PP0369" (CMUV), 1 ♂ "Italia Toscana Isola d'Elba, Norsi 42.7700, 10.3461 7.VII.2013 lg. L. Forbicioni / PP0370" (CMUV), 1 ♂ "Italia Toscana Isola d'Elba, Norsi 42.7700, 10.3461 7.VII.2013 lg. L. Forbicioni / PP0371" (CMUC), 1 ♂ "Italia Toscana Isola d'Elba, Norsi 42.7700, 10.3461 7.VII.2013 lg. L. Forbicioni / PP0372" (CMUC), 1 ♂ "Italia Toscana Isola d'Elba, Norsi 42.7700, 10.3461 7.VII.2013 lg. L. Forbicioni / PP0373" (CMUC), 1 ♂ "Italia Toscana Isola d'Elba, Norsi 42.7700, 10.3461 7.VII.2013 lg. L. Forbicioni / PP0374" (CMUC), 1 ♂ "Italia Toscana Isola d'Elba, Norsi 42.7700, 10.3461 7.VII.2013 lg. L. Forbicioni /

PP0375" (CMUC), 1 ♂ "Italia Toscana Isola d'Elba, Norsi 42.7700, 10.3461 7.VII.2013 lg. L. Forbicioni / PP0376" (CMUC), 1 ♂ "Italia, Porto Azzurro (LI) loc. Norsi 17 VI 2010 leg. G. Frangini margine di gariga / PP0377" (CMUC), 1 ♂ "Capoliveri (LI) loc. Norsi - margine di gariga 17.VI.2010 leg. L. Forbicioni & G. Frangini / Aedeagus not dissected / PP0378" (CMUC).

Specimens not included in morphometric analysis (ID based on IUMG): Paratypes: 1 ♂ "Capoliveri Norsi 4.VII.2018 leg. L. Forbicioni " (CLFP), 5 ♂♂ "18.VI 2014 - TOSCANA Capoliveri (Li) Loc. Norsi leg. L. Forbicioni" (CLFP), 1 ♂ "Loc. Norsi Capoliveri Portoferraio (LI) 03.VI.2013 Leg: Forbicioni L." (CSNC), 1 ♂ "Loc. Norsi Capoliveri Portoferraio (LI) 03.VI.2013 Leg: Forbicioni L." (CMBB), 1 ♂ "ITALIA Toscana (LI) Portoferraio – 40 mt. 30.VI.2017 – Buraccio L. Forbicioni leg/ Case Galletti 42°46'47,6'' N 10°21'16,7''E" (CLFP), 1 ♂ "ITALIA Toscana (LI) Portoferraio/ P. Azurro, 29.VI.2017 L. Forbicioni leg/ Fosso del Buraccio 42°46'47,1''N 10°21'25,2''E" (CLFP), 1 ♂ "IT Toscana (LI) Capoliveri - Loc. Norsi 04. VII.2018 L. Forbicioni leg" (CLFP), 1 ♂ "25.VIII.2014 Capoliveri (LI) Loc. Barabarca/ leg. Antonio Pinna" (CLFP), 1 ♂ "Capoliveri Norsi 17.VI.2010 lg. G. Frangini" (CLFP).
